# Decoding the complexity of on-target integration: characterizing DNA insertions at the CRISPR-Cas9 targeted locus using nanopore sequencing

**DOI:** 10.1186/s12864-024-10050-6

**Published:** 2024-02-17

**Authors:** Juan-Juan Zhao, Xin-Yu Sun, Sai-Ning Tian, Zong-Ze Zhao, Meng-Di Yin, Mei Zhao, Feng Zhang, Si-Ang Li, Zhi-Xue Yang, Wei Wen, Tao Cheng, An Gong, Jian-Ping Zhang, Xiao-Bing Zhang

**Affiliations:** 1 State Key Laboratory of Experimental Hematology, Haihe Laboratory of Cell Ecosystem, Institute of Hematology & Blood Diseases Hospital, National Clinical Research Center for Blood Diseases, Chinese Academy of Medical Sciences & Peking Union Medical College, Tianjin, 300020 China; 2 Tianjin Institutes of Health Science, Tianjin, 301600 China; 3 Tianjin Medical University, Tianjin, China; 4 College of Computer Science and Technology, China University of Petroleum (East China), Qingdao, 266000 China

**Keywords:** CRISPR-Cas9, Gene therapy, DNA integration, Long-range PCR, Nanopore sequencing

## Abstract

**Background:**

CRISPR-Cas9 technology has advanced in vivo gene therapy for disorders like hemophilia A, notably through the successful targeted incorporation of the F8 gene into the Alb locus in hepatocytes, effectively curing this disorder in mice. However, thoroughly evaluating the safety and specificity of this therapy is essential. Our study introduces a novel methodology to analyze complex insertion sequences at the on-target edited locus, utilizing barcoded long-range PCR, CRISPR RNP-mediated deletion of unedited alleles, magnetic bead-based long amplicon enrichment, and nanopore sequencing.

**Results:**

We identified the expected F8 insertions and various fragment combinations resulting from the in vivo linearization of the double-cut plasmid donor. Notably, our research is the first to document insertions exceeding ten kbp. We also found that a small proportion of these insertions were derived from sources other than donor plasmids, including Cas9-sgRNA plasmids, genomic DNA fragments, and LINE-1 elements.

**Conclusions:**

Our study presents a robust method for analyzing the complexity of on-target editing, particularly for in vivo long insertions, where donor template integration can be challenging. This work offers a new tool for quality control in gene editing outcomes and underscores the importance of detailed characterization of edited genomic sequences. Our findings have significant implications for enhancing the safety and effectiveness of CRISPR-Cas9 gene therapy in treating various disorders, including hemophilia A.

**Supplementary Information:**

The online version contains supplementary material available at 10.1186/s12864-024-10050-6.

## Background

Recent advancements in genome engineering, facilitated by the development of engineered nucleases, have revolutionized molecular biology. Among these tools, the CRISPR (Clustered Regularly Interspaced Short Palindromic Repeats) system, particularly the CRISPR-Cas9 technology, has shown immense promise for therapeutic applications to treat or potentially cure human genetic diseases [1–3]. The CRISPR-Cas9 system, derived from a bacterial adaptive immune system, comprises a Cas9 endonuclease and a guide RNA. This guide RNA can be a combination of crRNA and tracrRNA or a single chimeric guide RNA (sgRNA). In the CRISPR system from Streptococcus pyogenes, the SpCas9-sgRNA complex induces double-stranded DNA breaks 3 base pairs (bp) upstream of a protospacer adjacent motif (PAM) identified by the NGG sequence. This DNA cleavage activates two primary cellular DNA repair pathways: nonhomologous end-joining (NHEJ) and homology-directed repair (HDR).

NHEJ, often error-prone, can lead to small insertions, deletions (indels), or substitutions at the break site. These alterations can result in frameshift mutations or the generation of premature stop codons, ultimately leading to gene inactivation [4]. On the other hand, HDR, a more precise repair mechanism, facilitates the introduction of specific genetic modifications. This is achieved by providing an exogenous donor template with the desired sequence flanked by homologous regions to the target site. Such precision allows for gene knock-in, correction, or targeted mutagenesis, thereby enabling controlled genomic alterations [5].

CRISPR-Cas9-mediated gene knockout therapies have yielded promising outcomes in clinical settings [6–8]. Ex vivo therapies, which involve extracting cells from patients, editing them in vitro using the Cas9-sgRNA system, and then reinfusing them, have been applied in treating genetic blood disorders such as β-thalassemia and sickle cell disease [7]. Advanced editing technologies like base editors and prime editors have further enabled precise nucleotide modifications in a programmable fashion [9]. In addition, in vivo CRISPR-Cas9 editing employing lipid nanoparticles has demonstrated therapeutic efficacy in patients with transthyretin amyloidosis [10].

Nonetheless, many effective gene therapies necessitate the introduction of large transgenes to restore gene function. This is particularly relevant in conditions like hemophilia A, caused by mutations in the F8 gene. In a previous study, we demonstrated the successful treatment of hemophilia A in mice by injecting CRISPR components and a double-cut donor plasmid. Our results indicated that NHEJ-mediated ectopic insertion of B domain-deleted F8 (BDDF8) into the Alb gene in just 1–2% of liver cells was sufficient to fully restore serum F8 activity [11].

While CRISPR-Cas9 has shown remarkable efficacy, concerns regarding its safety remain. Both on-target and off-target editing can lead to undesirable insertions and deletions or more extensive chromosomal rearrangements, including deletions, translocations, and inversions [12–15]. Additionally, more severe outcomes such as chromothripsis [16] and chromosome loss [17–19] have been reported. Beyond these issues, unintended integrations of exogenous sequences like genomic DNA fragments [20], plasmids [21–23], and LINE-1 retrotransposons [24] have also been documented following CRISPR-Cas9 editing. Therefore, a comprehensive assessment and vigilant monitoring of the safety profile of CRISPR-Cas9 technology are imperative for its continued clinical advancement.

Numerous methodologies have been established to evaluate the potential adverse effects of genome editing. GUIDE-seq [25], for instance, is frequently utilized to detect and quantify off-target effects, but methodologies for analyzing and quantifying large insertions resulting from CRISPR-Cas9 are still limited. While Next-Generation Sequencing (NGS) is commonly employed to analyze small indels and assess HDR and NHEJ outcomes, especially when short homology arms are involved [26], it falls short in detecting large deletions and insertions due to the limitations imposed by short read lengths. Southern blot hybridization is another technique that can be used to assess large insertions [22]; however, its application is mainly restricted to single-cell clones and lacks the capability to investigate integrations with low incidence.

Recent advancements in third-generation sequencing (3GS) technologies have opened new avenues for accurately detecting complex genomic alterations. Both the Pacific Biosciences (PacBio) single-molecule real-time sequencing (SMRT-seq) and Oxford Nanopore Technologies (ONT) sequencing have demonstrated their potential in identifying gene modifications post gene editing [23, 27–29]. In our prior work, we utilized nanopore sequencing to evaluate large deletions and formulated a bioinformatics pipeline to process the data [30]. The current study introduces a comprehensive methodology for analyzing insertion events following targeted genome editing.

By employing a combination of optimized barcoded long-range PCR, CRISPR cleavage-mediated elimination of unedited alleles, magnetic bead-based amplification of long amplicons, and nanopore sequencing, we were able to delineate the intricacies of on-target insertion events. Our investigation uncovered that the most frequent insertion was a single fragment of linearized double-cut donor plasmid in both forward and reverse orientations. Additionally, we encountered notable instances of integration involving various combinations of 2–3 pieces of F8 and the plasmid backbone. Intriguingly, a small fraction of these insertions stemmed not from the donor plasmids but from genomic DNA fragments or LINE elements. Furthermore, we detected complex insertions exceeding ten kilobases, comprising multiple segments from diverse origins. This sheds new light on the mechanisms of DNA fragment integration at sites cleaved by CRISPR in double-stranded DNA.

## Results

### Reduced incidence of large deletions following in vivo CRISPR editing in Hemophilia A mice

**Fig. 1:**
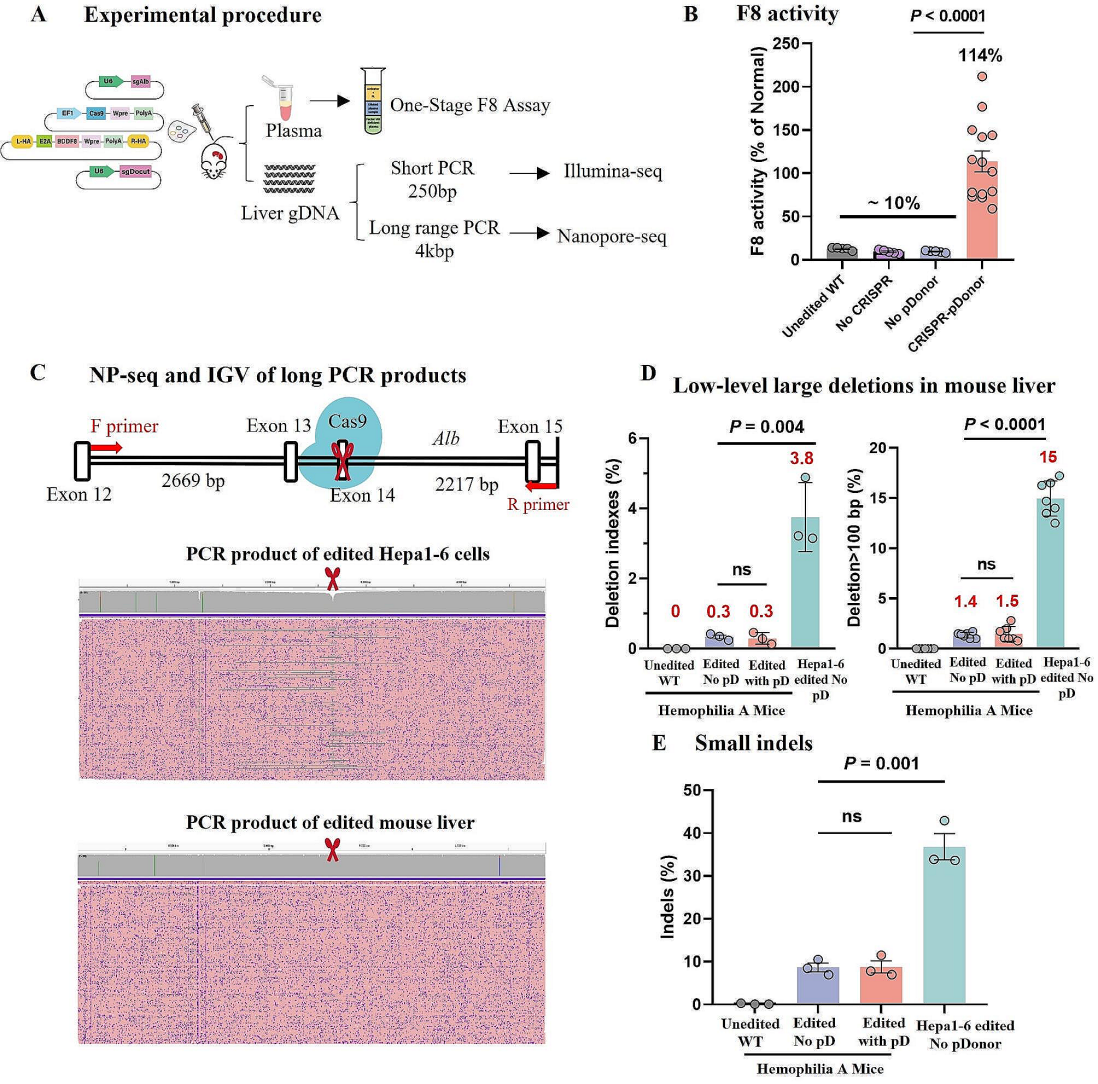
Evaluation of large deletions at the Alb target site in CRISPR and F8 donor gene-edited hemophilia A mice. (**A**) Schematic representation of the experimental workflow, involving hydrodynamic injection of editing plasmids into hemophilia A mice. Post-treatment assessments included measuring F8 activities via One-Stage F8 Assay and analyzing indels through Illumina sequencing, Crispresso2, and long-range PCR coupled with nanopore sequencing to detect potential large deletions. (**B**) Quantification of serum F8 activity three weeks post-administration in different groups: untreated mice (*n* = 5 mice), mice injected without CRISPR components (*n* = 5 mice), mice injected without pD-BDDF8-sg (*n* = 5 mice), and mice injected with both CRISPR components and pD-BDDF8-sg (*n* = 14 mice). Error bars represent mean ± SEM. (**C**) Using long-range PCR and nanopore sequencing to identify large deletions. The IGV visualization displays 200 randomly sampled reads, with purple dots marking sequencing errors and red scissors denoting the sgAlb target site. A positive control was established using Hepa1-6 cells. (**D**) Comparative analysis of large deletions in edited liver tissue from hemophilia A mice versus in vitro edited Hepa1-6 cells (*n* = 3), using deletion indexes and D100 (percentage of deletions > 100 bp). Statistical analysis was performed using unpaired two-sided Student’s t-tests. (**E**) Comparison of small indel frequencies in CRISPR-edited mice with or without pD-BDDF8-sg (*n* = 3 mice each) and in vitro edited Hepa1-6 cells (*n* = 3). Statistical evaluations were conducted using one-way ANOVA and unpaired two-sided Student’s t-tests

In developing CRISPR-based gene therapy for hemophilia A, we employed hydrodynamic injection to deliver four key genetic constructs to the hemophilia A murine model. The first construct was a Cas9-encoding plasmid (pEF1-Cas9), essential for initiating gene editing. This was followed by the double-cut donor BDDF8 plasmid (pD-E2A-BDDF8-Wpre-PolyA, abbreviated as pD-BDDF8-sg), featuring custom homology arms for precise integration. Additionally, we introduced the sgAlb (pU6-sgAlb) guide RNA targeting the albumin locus for site-specific integration. Crucially, the sgDocut plasmid (pU6-sgDocut) was also administered, specifically designed to cleave the donor plasmid, thereby enhancing the efficiency of gene insertion in the hemophilia A murine model. This approach led to integrating the BDDF8 cassette into Exon 14 of the Alb gene in hepatocytes. The integration facilitated the high-level expression of a fusion transcript, Alb-BDDF8, thanks to the E2A linker, which allowed for the concurrent translation of both Alb and BDDF8 proteins via ribosome skipping [11, 31]. This approach mirrored our previous findings [11], where an average F8 activity level of 114% was observed three weeks after vector injection (Fig. 1A, B).

Large deletions at the CRISPR-mediated gene editing target site are a concerning byproduct. Previous studies have identified these deletions in approximately 10% of ex vivo edited T cells and hematopoietic stem/progenitor cells. However, the incidence was notably lower in human induced pluripotent stem cells, suggesting cell-type specific variations in deletion frequency [32]. We utilized optimized long-range PCR coupled with nanopore sequencing to assess the prevalence of large deletions following in vivo liver editing. This approach targeted regions extending 2669 bp upstream and 2217 bp downstream of the sgAlb target site (Fig. 1C; Supplementary Table S3). To ensure precise integration, we used constructs with homologous arms of appropriate lengths on both ends. Specifically, the genomic DNA extracted from mice that were injected with the double-cut donor pD-BDDF8-sg, which includes HA190-130 homologous arms, along with pEF1-Cas9, pU6-sgAlb, and pU6-sgDocut. Utilizing our previously established GREPore-seq pipeline [30], we calculated deletion indexes and D100 (deletions exceeding 100 bp). Contrary to expectations, the deletion indexes in edited liver samples were similar to wildtype controls, at merely 0.3%. D100 was slightly elevated at 1.5% in test samples compared to unedited controls, underscoring the sensitivity of D100 over deletion indexes (Fig. 1D). We speculated that the low D100 in the liver might be attributable to its quiescent nature. This was supported by editing the actively dividing mouse hepatocyte cancer cell line Hepa1-6, where we observed a tenfold increase in deletion index and D100 compared to in vivo hepatocyte editing. Further indel analysis using NGS and CRISPResso2 excluded the possibility of discrepancies due to varying editing efficiencies, revealing indel frequencies in edited Hepa1-6 cells to be 4–5 times higher than in hemophilia A mice (Fig. 1E; Supplementary Figure S1). Upon normalizing the data by calculating the ratio of D100 to indels, we noted a reduction of over 50% in large deletions for in vivo liver editing.

These observations suggest that the relatively dormant state of hepatocytes in vivo may offer a protective mechanism against the emergence of large deletions. This highlights the criticality of accounting for cell-type specific dynamics in the progression of CRISPR-mediated gene therapy.

### Challenges in enriching amplicons with F8 inserts using gel extraction

**Fig. 2:**
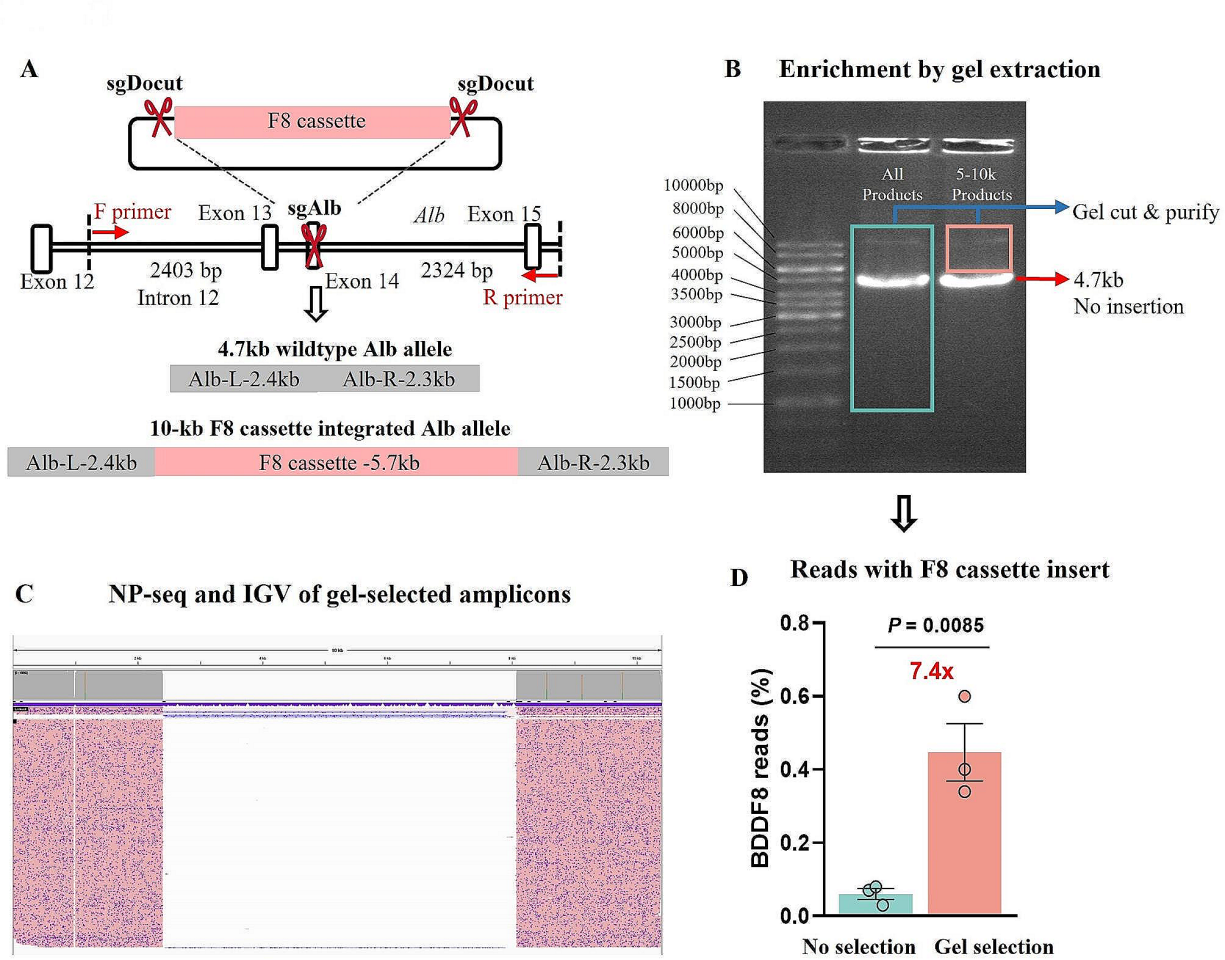
Challenges in enriching amplicons with F8 inserts via gel extraction. (**A**) Illustration of the expected PCR amplification products: approximately 4.7 kb for wild-type alleles and around 10 kb for alleles with integrated BDDF8 cassettes. (**B**) Conducting gel electrophoresis on PCR products, demarcating two distinct regions for gel extraction: amplicons greater than 1 kb and those exceeding 4.7 kb. (**C**) Nanopore sequencing (NP-seq) analysis of the gel-selected amplicons, aligned to the reference sequence of the 10 kb BDDF8-inserted Alb allele. The visualization displays a representative sample. (**D**) Quantitative assessment revealed that less than 1% of the reads demonstrate F8 insertions, even after gel extraction. Error bars represent mean ± SEM based on data from 3 mice. Statistical significance was evaluated using unpaired two-sided Student’s t-tests

Our study examined the effects of in vivo liver editing and focused on characterizing long-donor insertions. For this purpose, we employed long-range PCR to analyze genomic DNA extracted from mice. These mice had been injected with the pD-BDDF8-sg construct, which incorporates HA85-130 homologous arms, in conjunction with pEF1-Cas9, pU6-sgAlb, and pU6-sgDocut (Fig. 2A; Supplementary Table S4). Gel electrophoresis revealed a pronounced 4.7-kb band, likely representing the wild-type alleles, and a fainter band of approximately 10 kb, presumably indicative of F8 insertions (Fig. 2B). To enhance DNA yield with F8 insertions, we excised the 5-10 kb bands from the gel for subsequent nanopore sequencing. The sequencing reads were aligned to the anticipated reference sequence, showing F8 insertion at the Alb locus, and visualized using the Integrative Genomics Viewer (IGV) (Fig. 2C).

Despite employing gel extraction and nanopore sequencing as enrichment methods, the resulting data showed that only about 0.5% of the reads contained the F8 insert. This was a significant discrepancy, as most reads still represented background DNA (Fig. 2C, D). Although there was a 7-fold increase in the enrichment of F8-containing amplicons, the overall low proportion of desired amplicons poses a challenge to the efficacy and cost-effectiveness of this method. This outcome underscores the necessity for developing more efficient strategies to enrich and analyze donor-inserted amplicons in the context of CRISPR-mediated gene editing.

### Enhancing long amplicon enrichment through refined PCR design and magnetic bead selection

We explored using magnetic beads for size selection to enrich long amplicons, eliminating short DNA fragments efficiently. Optimal bead ratios between 0.35x and 0.45x were efficient (Supplementary Figure S2A). We then revisited our PCR strategy, managing to reduce the size of background PCR products from 4.8 kb to 1.6 kb. This adjustment allowed us to amplify insertions of pD-BDDF8-sg featuring 600 bp homology arms specifically. We devised primers to target regions 693 bp upstream and 889 bp downstream of the Alb target site (Supplementary Figure S2B; Supplementary Table S5), and the second PCR primer was designed to amplify a 1.38-kb region, centering on the on-target cut site (Supplementary Figure S2C).

Initial tests with magnetic bead (Magbeads) ratios ranging from 0.35x to 0.425x suggested that a 0.4x ratio was the most effective in enriching amplicons with F8 insertions (~ 7-kb). However, the difference was not statistically significant (Supplementary Figure S2D). The highest enrichment achieved was less than 5-fold. Nanopore sequencing indicated that only about 0.5% of the reads contained Alb with F8 insert following size selection using magnetic beads (Supplementary Figure S2E), suggesting that using magnetic beads to separate the ~ 7-kb product from the 1.4–1.6 kb background was not highly efficient.

**Fig. 3:**
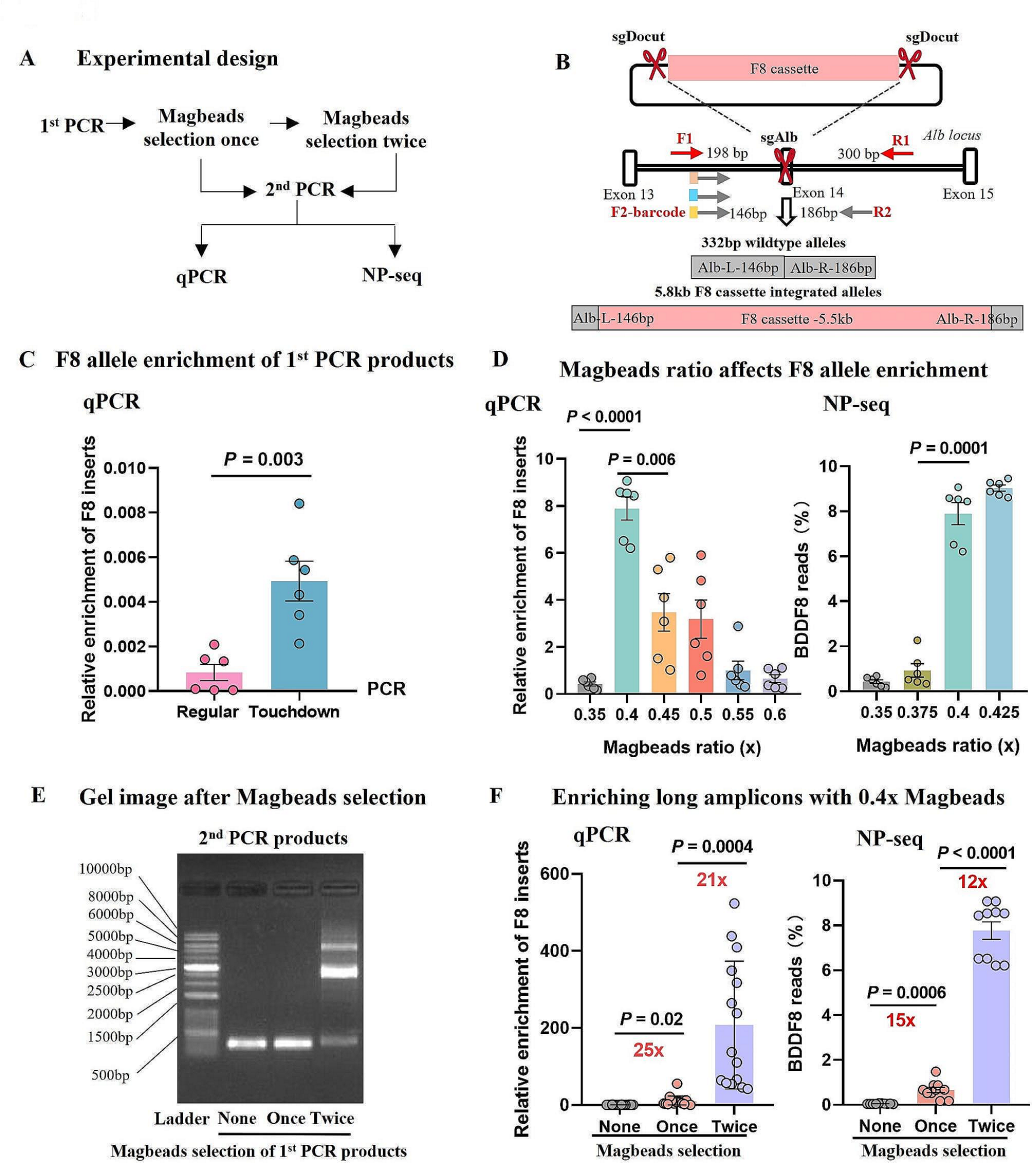
Enhanced enrichment of long amplicons via refined nested PCR and magnetic beads selection. (**A**) Outline of the experimental approach for optimizing magnetic beads selection. (**B**) Primer design targeting the generation of short and long PCR products (332 bp for wild-type alleles and approximately 5.8 kb for alleles with integrated F8 cassettes) through nested PCR. (**C**) Evaluation of F8 allele enrichment in the 1st PCR products using regular PCR and touchdown PCR, assessed by qPCR. Error bars indicate mean ± SEM, based on data from 6 mice. Paired two-sided Student’s t-tests were used for statistical analysis. (**D**) Fine-tuning of the magnetic beads ratio to optimize F8 allele enrichment. The assessment was conducted through qPCR and nanopore sequencing of the 2nd PCR products derived from magnetic beads size-selected 1st PCR products. Error bars represent mean ± SEM, based on data from 6 mice. Paired two-sided Student’s t-tests were used for statistical analysis. (**E**) Gel electrophoresis display of 2nd PCR products with and without magnetic beads size-selection applied to the 1st PCR products. (**F**) Quantitative assessment of the relative enrichment of the F8 allele in the 2nd PCR products following 0.4x magnetic beads size selection of 1st PCR products. The evaluation was performed using qPCR (*n* = 15 PCR reactions) and nanopore sequencing (*n* = 10 PCR reactions). Error bars indicate mean ± SEM. Paired two-sided Student’s t-tests were conducted for statistical analysis

To further refine the enrichment process, we focused on reducing the size of the background PCR product and optimizing the magnetic bead selection. We employed qPCR and nanopore sequencing to assess the effectiveness of these modifications (Fig. 3A). In designing the primers, we aimed to maintain a 100 bp stretch of genomic DNA from the target site to accommodate potential deletions and exclude PCR artifacts during bioinformatic analysis. The new primer set targeted regions 198 bp upstream and 300 bp downstream of the Alb cutting site (Fig. 3B; Supplementary Table S6).

We used genomic samples from mice injected with donor constructs containing HA85-0. Initial PCR amplification predominantly yielded wildtype alleles, with F8-containing amplicons accounting for approximately 0.05% of the total, as determined by qPCR (Fig. 3C). We explored optimizing PCR conditions to mitigate the preferential amplification of short amplicons. At an annealing temperature of 64 °C, a significant increase in the proportion of F8 alleles in the PCR products was observed (Supplementary Figure S4A). We also compared regular PCR with touchdown PCR (TD-PCR), which enhanced the amplification of larger target fragments [33]. Our results indicated that TD-PCR was superior to the standard method (Supplementary Figure S4B). These optimizations led to a 2-fold increase in F8-containing amplicons in the primary PCR products, prompting us to adopt these conditions for subsequent experiments.

Next, we fine-tuned the magnetic bead ratio to maximize the elimination of the 0.5-kb background and enriched the F8-integrated amplicons. Testing ratios from 0.35x to 0.60x, we found that 0.40x and 0.425x ratios were the most effective, with no significant difference (Fig. 3D).

We implemented two size-selections after the initial PCR to further enrich donor-inserted alleles. The secondary nested primers were predicted to yield a 332 bp background amplicon and products with insertions. Gel electrophoresis of the secondary PCR products revealed the background only in the control and after one 0.4x Magbeads selection of the primary PCR products. However, two size selections revealed distinct bands with insertions at ~ 2.5 kb and ~ 6 kb (Fig. 3E). qPCR analysis showed that each selection increased F8 enrichment by approximately 20-fold. Nanopore sequencing confirmed the efficacy of this approach, with a 12- to 15-fold improvement in detected insertion rates per size selection (Fig. 3F).

While this double size-selection strategy significantly improved enrichment, the background reads still exceeded 90% (Supplementary Figure S3), indicating that additional refinements may be necessary to enhance detection sensitivity further.

### Optimizing the enrichment of F8-inserted amplicons through in vitro RNP cleavage

**Fig. 4:**
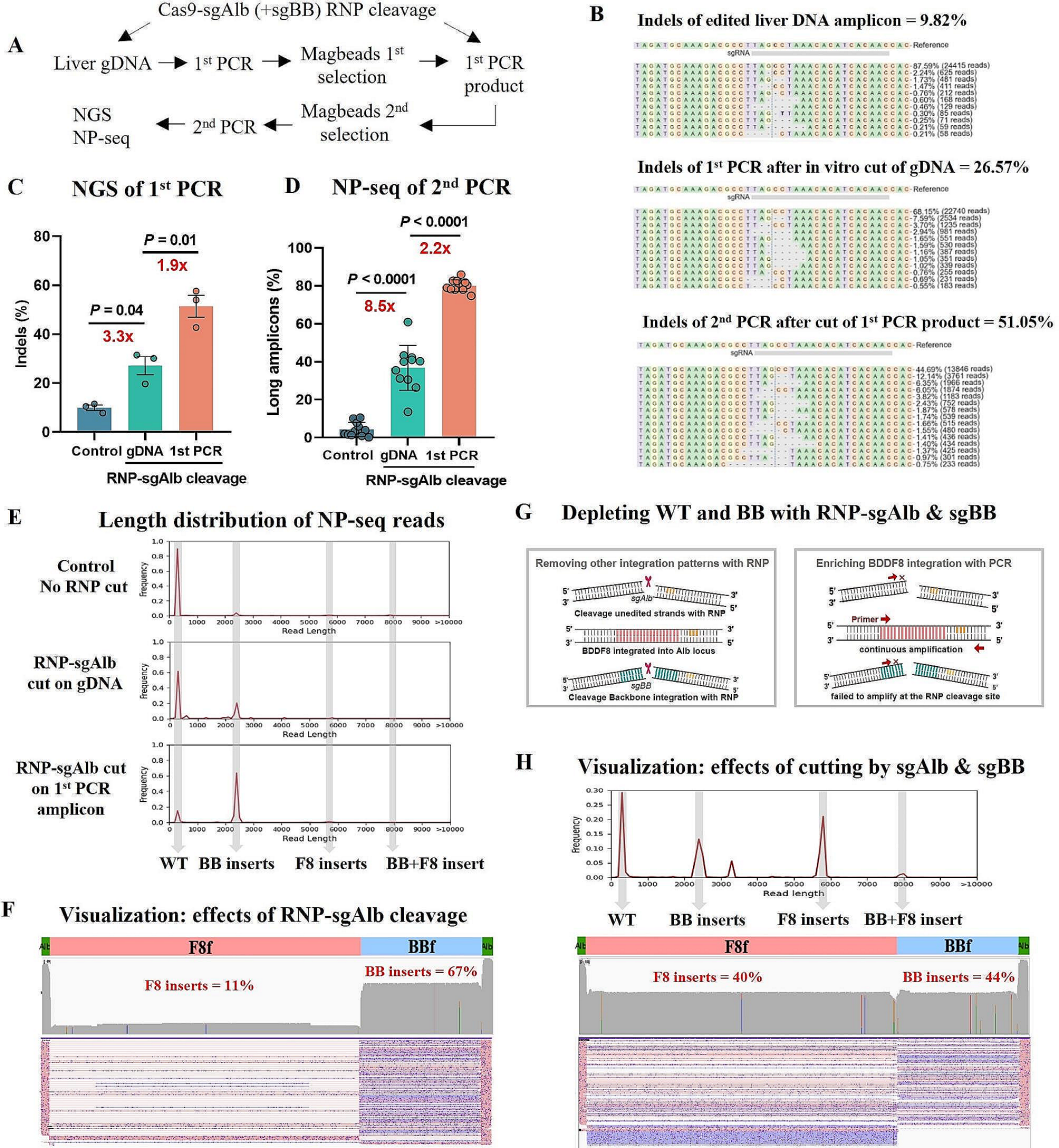
Targeted enrichment of F8-integrated amplicons via in vitro RNP cleavage. (**A**) Schematic illustrating the process for optimizing amplicon enrichment with F8 inserts through in vitro RNP cleavage. (**B** & **C**) Analysis of representative indels in genomic DNA, RNP-sgAlb-cleaved genomic DNA, or sgAlb-cleaved 1st PCR products. The assessment was performed using NGS of Alb amplicons and CRISPResso2. Error bars indicate mean ± SEM, based on data from 3 mice. Paired two-sided Student’s t-tests were used for statistical analysis. (**D**) Nanopore sequencing (NP-seq) analysis showing the percentage of long amplicons in 2nd PCR products amplified from genomic DNA, RNP-sgAlb-cleaved genomic DNA, and RNP-sgAlb-cleaved 1st PCR products. Error bars represent mean ± SEM, based on data from 11 PCR reactions. Paired two-sided Student’s t-tests were conducted. (**E**) Length distribution of 2nd PCR products amplified from genomic DNA, RNP-sgAlb-cleaved genomic DNA, and RNP-sgAlb-cleaved 1st PCR products. (**F**) Visualization of 2nd PCR products amplified from RNP-sgAlb-cleaved 1st PCR products after double size selection using a 0.4x beads ratio. Red alignments indicate reads aligning with the reference sequence, while purple alignments indicate reads aligning with the reverse complement of the reference. (**G**) Illustration of the depletion of Alb background PCR products using RNP-sgAlb and the removal of plasmid backbone inserts at the Alb site using RNP-sgBB. (**H**) Length distribution and visualization of 2nd PCR products amplified from 1st PCR products cleaved by both RNP-sgAlb and RNP-sgBB

Given that our indel analysis indicated about 95% of alleles were wildtype (Fig. 1E), we theorized that removing these unedited alleles could significantly enrich donor-inserted amplicons. To test this, we employed an in vitro ribonucleoprotein complex (RNP) comprising Cas9 protein and sgRNA targeting the same Alb locus (RNP-sgAlb). This approach aimed to deplete most wild-type alleles selectively (Fig. 4A). For this experiment, the genomic DNA used was from mice injected with pD-BDDF8-sg, which featured HA85-0 homologous arms, along with pEF1-Cas9, pU6-sgAlb, and pU6-sgDocut.

Our evaluation of genomic DNA and first PCR products as substrates for RNP-sgAlb cleavage showed average indels of 27% and 51% after cleavage, respectively (Fig. 4B, C). This observation suggested that using first PCR products as the substrate was more effective for removing unedited alleles. Consequently, we saw a marked increase in long fragment amplicons (> 2 kb), reaching up to approximately 80% in second PCR products (Fig. 4D). Additionally, the length distribution analysis showed a significant reduction in short background amplicons and an increase in longer amplicons over 2 kb (Fig. 4E).

However, when analyzing the second PCR amplicons, we encountered an unexpected issue: a predominant 2 kb fragment corresponding to the donor plasmid backbone, indicative of a bias towards shorter amplicons (Fig. 4E). Despite the linearization of the double-cut donor releasing the backbone (2.1 kb) and the F8 cassette (5.4 kb) in equal molar quantities, nanopore sequencing revealed a six-fold higher frequency of backbone insertions compared to F8 cassette insertions (Fig. 4F). This discrepancy underscored the need to minimize the amplification of backbone insertions and to develop approaches that favor the expansion of longer amplicons.

To address this, we introduced an additional RNP targeting the donor backbone (RNP-sgBB) to specifically cut and thereby reduce the frequency of backbone insertions. This step aimed to enrich F8 insertions (Fig. 4G). Following this adjustment, the length distribution map of the second amplicons and subsequent sequence analysis showed significant F8 insertions and a more balanced distribution between the backbone and F8 cassette insertions (Fig. 4H).

In summary, by incorporating in vitro RNP-sgAlb and RNP-sgBB cleavage, we successfully characterized a diverse range of gene insertion outcomes, overcoming the initial challenges of amplicon bias and enriching the dataset with relevant F8 insertions.

### Unraveling the complexity of donor plasmid integration patterns at the Alb site

In our previous study, we identified insertions from five F8 donors with various homology arm lengths (HA600-600, HA190-130, HA190-0, HA85-130, HA85-0), all flanked by Cas9-sgDocut sequences [11]. To further investigate the intricacies of DNA fragments inserted at the target site post-editing, we focused on the HA85-0 F8 donor. It is also important to note that, although plasmid delivery was used for BDDF8 in our study, AAV vectors are commonly employed in broader clinical strategies to package the BDDF8 cassette. The choice of the HA85-0 donor structure is a practical decision that balances the limitations of AAV packaging, which can be challenging with overly long homology arms and the need for effective genome editing. This analysis involved a two-step PCR procedure: initial PCR products were magnetically size selected, cleaved with RNP-sgAlb-sgBB, purified again, and subjected to a second PCR.

**Fig. 5:**
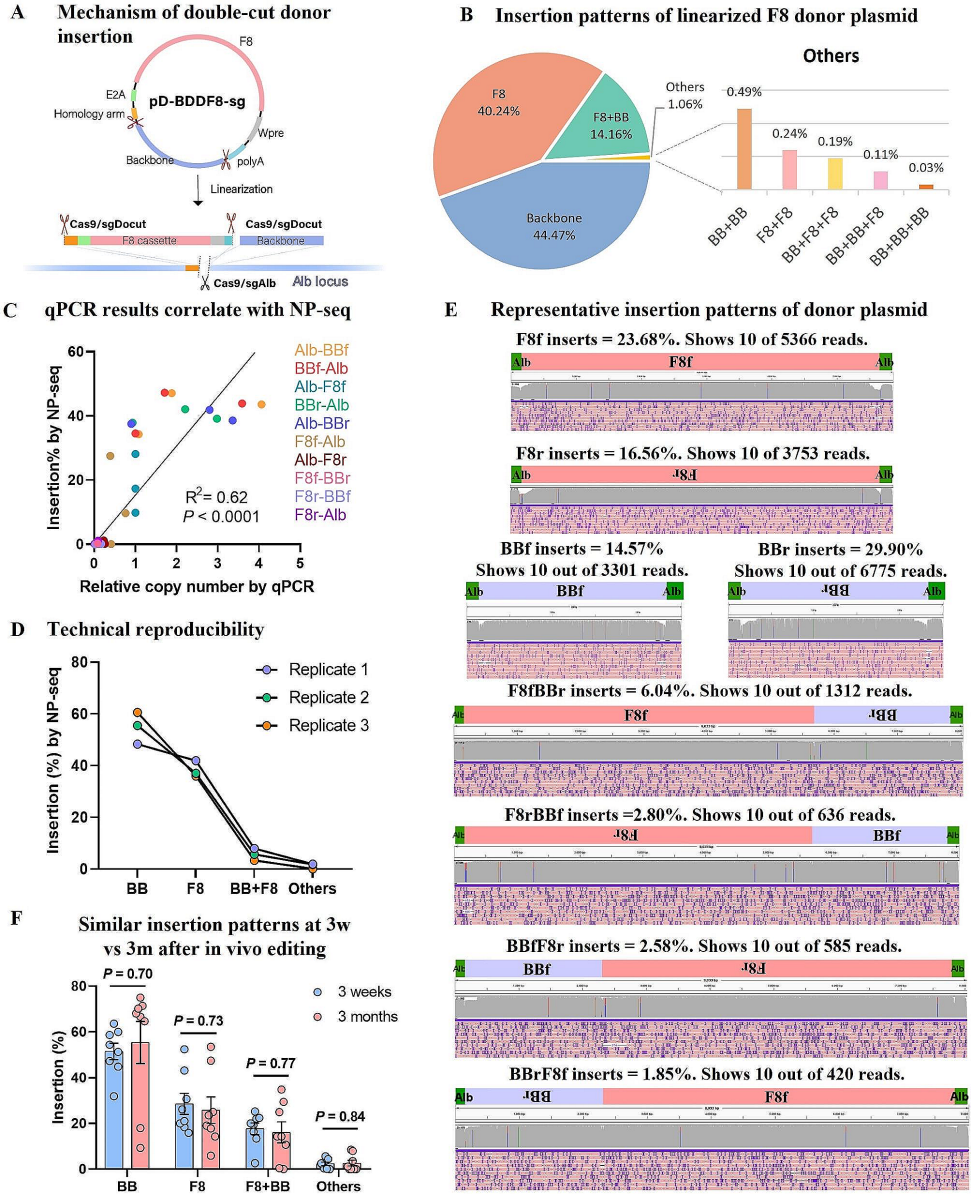
Diverse integration patterns of donor plasmid fragments at the Alb site revealed by nanopore sequencing. (**A**) Diagram illustrating how the double-cut donor plasmid is linearized into two fragments in hepatocytes, followed by their integration at the sgAlb-cleaved genome site. (**B**) Identification of various patterns of integration of the F8 gene and/or plasmid backbone at the on-target site, as determined by magnetic beads selection and nanopore sequencing. (**C**) Correlation analysis validating the proportion of different integration events determined by nanopore sequencing. The top six insertion patterns, namely F8f (forward F8), F8r (reverse F8), BBf (forward plasmid backbone), BBr (reverse plasmid backbone), F8fBBr (forward F8 with reverse plasmid backbone), and F8rBBf (reverse F8 with forward plasmid backbone), were also evaluated using qPCR analysis. This analysis focused on junction sequences between Alb, F8, and BB, employing 10 pairs of primers specifically designed for this purpose. (**D**) Technical reproducibility assessment based on three replicates of the same sample using nanopore sequencing. (**E**) Visualization of eight representative insertion patterns of the donor template. The BDDF8 cassette is abbreviated as “F8”, and the plasmid backbone as “BB”. Forward insertions are denoted as “f”, and reverse insertions as “r”. (**F**) Changes in the proportion of hepatocytes carrying different insertion patterns at 3 weeks vs. 3 months post-injection with CRISPR and BDDF8 donor plasmids. Error bars represent mean ± SEM, based on data from 8 mice. Unpaired two-sided Student’s t-tests were conducted for statistical analysis

Following vector delivery, the double-cut donor was cleaved into two components: the F8 cassette and the plasmid backbone (BB) (Fig. 5A). One or more fragments could be integrated at the double-strand break site. We analyzed integration outcomes involving single, double, or triple donor fragments. We employed the GREPore-seq bioinformatic pipeline to sort donor insertion patterns from various mouse liver samples into nine distinct categories: B (single BB integration), F (single F8 integration), BF (combination of BB and F8), BB (two BBs concatenated), FF (two F8 cassettes concatenated), BBF (concatenation of two BBs and one F8), BFF (one BB and two F8 cassettes concatenated), BBB (triple BB concatenation), and FFF (triple F8 concatenation).

To accommodate the ~ 5% sequencing errors typically associated with nanopore sequencing and the potential partial truncation of donor fragments, we expanded the interval by ± 20% based on the expected perfect insertion size during data analysis. We then scrutinized the data using grepseqs for forward backbone insertion (Bf), reverse backbone insertion (Br), forward F8 cassette insertion (F8f), and reverse F8 cassette insertion (F8r) within the specific length range of the nine categorized data sets. Subsequently, we calculated the ratios of single F8 cassette insertions and backbone integrations, which were 40.24% and 44.47%, respectively. About 38% of insertions were in the correct orientation. Within this subset of correctly oriented insertions, we investigated the occurrence of HDR events. Our junction PCR verification and computational analysis indicated that the HDR occurrence for this vector ranged between 54 and 65%.

Notably, complete donor integration represented 14.16% of reads, corresponding to total insertion events, while the rest involved combinations of two identical fragments or three-fragment integrations (Fig. 5B). Among the possible 64 three-element integration patterns, we identified 37 compound insertion patterns, although they occurred at relatively low frequencies (~ 0.01%) (Supplementary Figure S6).

To validate the proportions of each integration pattern, we designed ten primer pairs to amplify specific junctions—left Alb-BB, left Alb-F8, BB-right Alb, F8-right Alb, and F8-BB—for quantitative PCR (qPCR) analysis of the purified first PCR products. This analysis revealed that the relative copy numbers aligned with the proportions determined by nanopore sequencing (Fig. 5C; Supplementary Table S7). The correlation coefficient (R^2^ = 0.62) between qPCR results and nanopore sequencing data substantiates our claims regarding the relative proportions of each insertion pattern. For further validation of our analysis’ accuracy, we employed the same sample to perform long-range PCR, magnetic bead-based enrichment, nanopore sequencing, and analysis focusing on the four primary types of integration patterns. Consistently, we observed comparable results, confirming the robust technical reproducibility of our methods (Fig. 5D). These findings underscore the capability of nanopore sequencing to accurately evaluate the relative proportions of complex insertion patterns in gene editing experiments.

The donor plasmids in this study featured an 85 bp homology arm on the left side, potentially facilitating HDR and thus potentially leading to the elimination of the homologous sequence in forward insertions of the F8 gene. To streamline our analysis of insertion patterns, we generated reference sequences based on the assumption of donor insertion via the NHEJ repair pathway. Figure 5E provides eight representative visual alignment results in sketch form, while Supplementary Figure S5 offers more detailed visualizations. Additionally, Supplementary Figure S6 displays all other identified integration patterns. The alignment of nearly all reads with their respective grouped patterns across all cases supports the effectiveness of our proposed analysis approach, which involves combining read length assessment with the identification of unique sequences.

Our nanopore sequencing-based analysis uncovered a multitude of compound integration patterns of the donor plasmid, most of which were unintended and nonfunctional. To assess whether these insertion events could potentially harm edited hepatocytes, we harvested liver samples for PCR and sequencing at two time points: three weeks and three months post-CRISPR-F8 editing in hemophilia A mice. The rationale was that if these genomic alterations were detrimental, they might trigger cellular stress, potentially leading to cell death and a consequent reduction in occurrence. However, the comparative analysis showed no significant changes in the ratios of F8, BB, F8 + BB, and compound insertions between the two groups of edited mice (Fig. 5F). This outcome suggests that these unintended insertion patterns do not adversely affect the viability or overall health of the host cells.

In summary, our investigation revealed various unintended and nonfunctional compound integration patterns of the donor plasmid. Importantly, these insertion events did not negatively impact the edited hepatocytes, as evidenced by the consistent proportions of insertions observed in two groups of edited mice harvested at different intervals. This outcome supports the safety of CRISPR-F8 and similar in vivo gene therapies. However, it is essential to conduct further research to fully understand the long-term implications of these unintended insertions.

### Validation of BDDF8 cassette integration through long-range junction PCR and nanopore sequencing

**Fig. 6:**
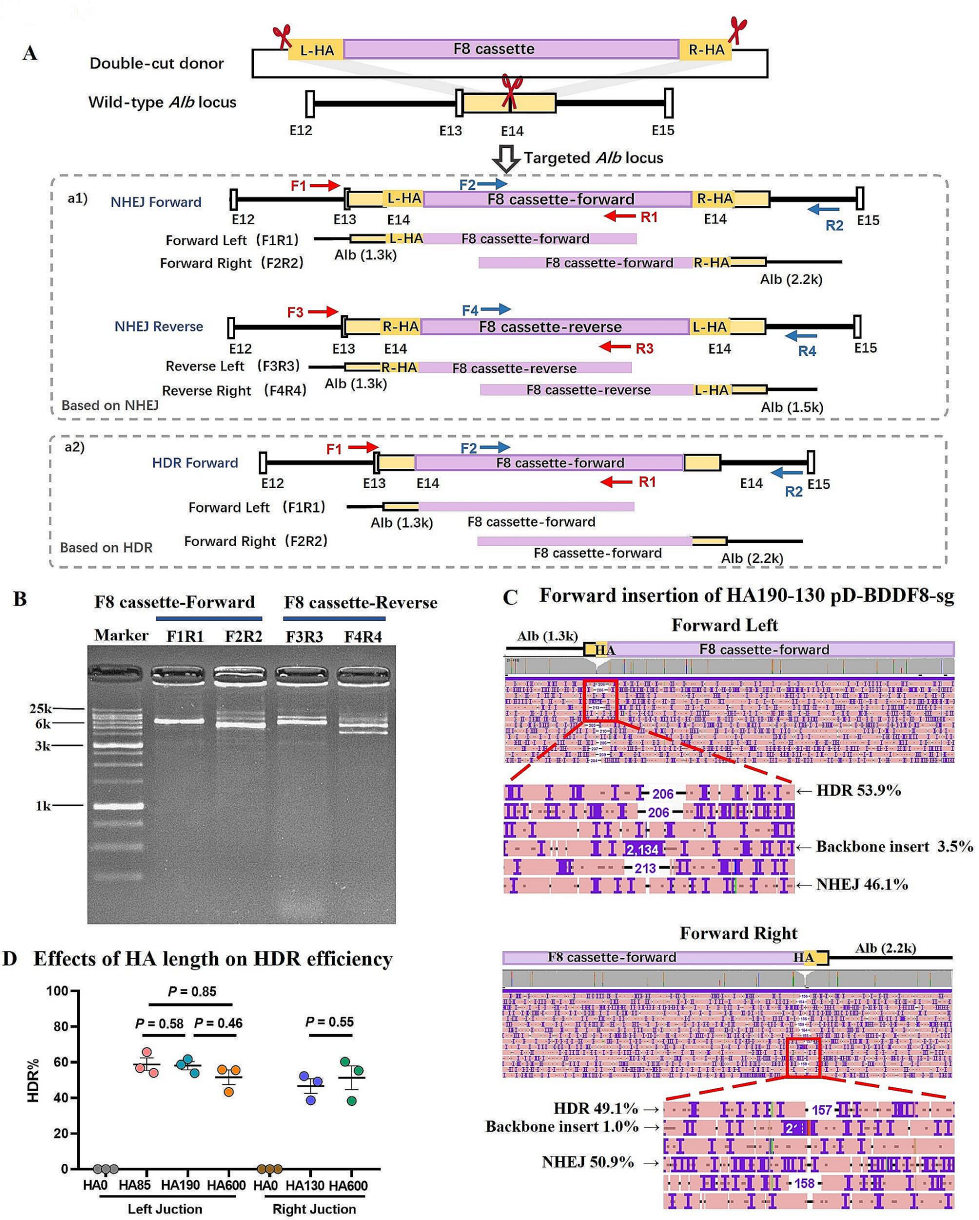
Precise evaluation of NHEJ and HDR efficiency through NP-Seq analysis of long Alb-F8 insert junction PCR products. (**A**) Diagram showing forward and reverse integrations of the BDDF8 donor carrying homology arms. Four primer pairs were strategically designed to amplify the junctions: Left F8-Forward (F1-R1), Right F8-Forward (F2-R2), Left F8-Reverse (F3-R3), and Right F8-Reverse (F4-R4). The sgAlb target site is indicated by red scissors. (**B**) Demonstration of successful amplification of junctions using the designated primers. Shown here is a representative result of long-range PCR products obtained from the liver genomic DNA of an edited mouse. The identities of these PCR products were confirmed by Nanopore sequencing. (**C**) Visualization of 15 randomly selected reads depicting forward insertion of F8 at the left junction (FL) and forward insertion at the right junction (FR) of the donor with HA190-130. Labels indicate HDR and NHEJ alleles, plasmid backbone inserts, and their respective percentages. (**D**) Effect of homology arm length on HDR efficiency. Various homology lengths, ranging from 85 to 600 bp, were assessed. Error bars represent mean ± SEM, based on data from 3 mice. Unpaired two-sided Student’s t-tests were utilized for statistical analysis

To confirm our previous findings, we PCR amplified a region spanning 5 to 6 kb across the left and right junctions, using additional primers. Considering the possibility of the double-cut F8 donor inserting in either forward or reverse orientation at the dsDNA break, we designed four primer pairs, each consisting of one primer within the F8 cassette and another outside the homology arm (Fig. 6A; Supplementary Table S8). We used five different donors with distinct homology arm lengths (HA600-600, HA190-130, HA190-0, HA85-130, HA85-0). For consistency, 400 ng of genomic DNA was used as the template in all 20 µL long-range PCR reactions. The anticipated junction PCR sizes for NHEJ knock-in were 6428 bp (forward left insert) and 6709 bp (forward right insert), and 6086 bp (reverse left insert) and 5514 bp (reverse right insert) for NHEJ-mediated insertion. Gel electrophoresis was performed to confirm the presence of the expected band sizes (Fig. 6B). To further validate the PCR products, we performed nanopore sequencing, demultiplexed the sequencing data, and aligned it with the expected direct donor insertion reference sequences using Minimap2. The aligned data were visualized using the Integrative Genomics Viewer IGV (Fig. 6C; Supplementary Figure S7) [34, 35].

Our junction PCR and nanopore sequencing data validated the integration of the F8 donor plasmid in both orientations. Additionally, they facilitated the analysis of other genomic alterations occurring during CRISPR-Cas9 editing, such as short deletions in the Alb and donor sequences and the integration of the plasmid backbone. We found that deletions exceeding 100 bp in Alb were rare, aligning with previous findings indicating infrequent substantial deletions during in vivo liver editing (Supplementary Figure S8A). However, F8 fragment insertions led to deletions over 200 bp in 1–5% of reads (Supplementary Figure S8B). Plasmid backbone integration was observed in 1–5% of reads with F8 insertion, a lower frequency than in the analysis of insertion patterns. This discrepancy may be due to backbone insertion increasing amplicon sizes from 6 kb to 8 kb, resulting in less efficient PCR amplification under conditions optimized for 6 kb products. Consequently, the preferential amplification of shorter products may have led to an underestimation of the F8 + BB insertion frequency by 5–10 fold.

Supplementary Figure S9 presents the algorithm and flowchart used to distinguish between HDR editing and NHEJ insertion. The removal of the homologous arm sequence indicated HDR editing and was categorized as an HDR event. While previous studies in cell culture systems suggested that longer homology arms increase HDR efficiency, this study found HDR editing in 40–60% of F8 forward insertion events, regardless of homology arm lengths ranging from 85 to 600 bp (Fig. 6D). These findings imply that the length of homology arms does not significantly affect HDR efficiency in the liver, underscoring the need to explore how homology arm length and other factors influence HDR efficiency in various tissues.

### Analysis of degraded double-cut donor plasmid and circular CRISPR plasmids integration at the editing site

**Fig. 7:**
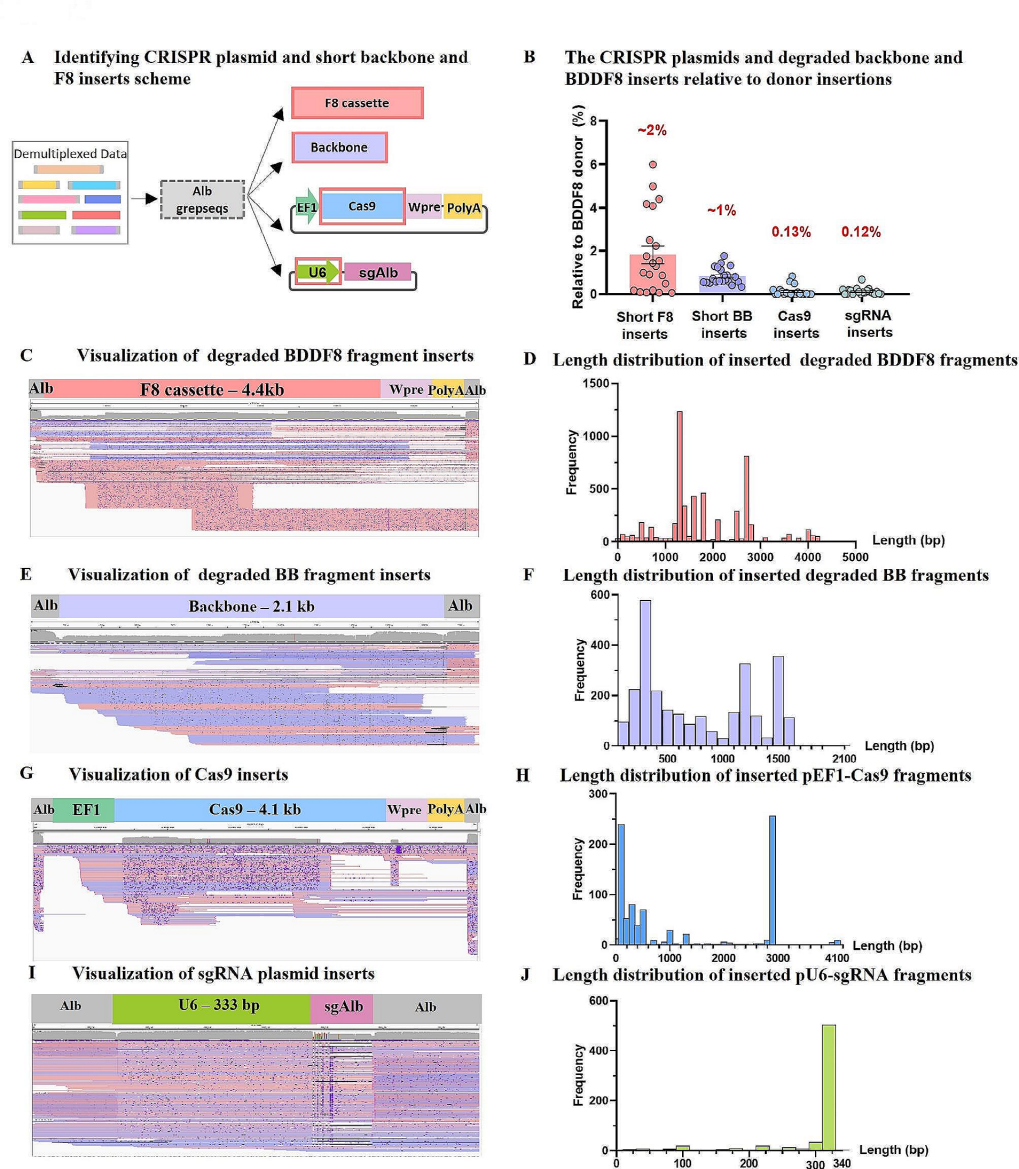
Characterization of CRISPR plasmid, short plasmid backbone, and F8 sequence insertions at the edited Alb site. (**A**) Strategy to analyze short insertions post-editing: The analysis began with grepseqs derived from regions 146 bp left and 186 bp right of the Alb cleavage site. Reads with insertions other than the F8 donor were isolated using unique sequences from Cas9 and U6, excluding full-length backbone and F8 insertions. Short plasmid backbone (defined as < 1700 bp) and F8 sequences (< 4400 bp) were then analyzed. (**B**) Insertion comparison: The frequency of reads with inserted short F8, short backbone, and CRISPR plasmids was compared to those carrying double-cut donor sequences. Error bars represent mean ± SEM, based on data from 21 mice. Paired two-sided Student’s t-tests were used. (**C**) Short F8 inserts visualization: 200 randomly selected short F8 inserts are visualized against the reference sequence of Alb gDNA flanking the entire F8 cassette sequence. (**D**) Short F8 inserts length distribution. (**E**) Short backbone inserts visualization: 200 randomly selected short backbone inserts are visualized against the reference sequence of Alb gDNA flanking the entire plasmid backbone sequence. (**F**) Short backbone inserts length distribution. (**G**) Cas9 Plasmid insertions visualization: Visualization against the reference sequence of Alb gDNA flanking the CRISPR plasmid sequence, excluding the backbone. (**H**) Cas9 sequence inserts length distribution analysis. (**I**) Visualization of inserted sgRNA plasmid fragments. (**J**) Length distribution of sgRNA plasmid sequence Inserts

Our previous studies primarily focused on the integration of relatively full-length linearized fragments of F8 and BB. However, we also observed deletions exceeding 200 bp in inserted plasmid sequences. In this analysis, we aimed to investigate the integration of degraded plasmid pieces. The samples utilized encompassed DNA from mice injected with donor constructs with various lengths of homologous arms, excluding HA600-600. Most linearized donor plasmid pieces were promptly captured at double-strand breaks (DSBs). We arbitrarily defined degraded F8 and BB as insertions with less than 80% of their original length. We first selected data by read length and then used unique 17-mer grepseqs of F8 and BB for further data selection (Fig. 7A). Visualization and insert length analysis revealed that degraded F8 and BB integration occurred at frequencies of 1–2% relative to intact fragment integration (Fig. 7B). The insert length varied among reads, but most inserts were less than half of the full F8 or BB (Fig. 7C-F).

We also examined the integration of the editing plasmids, pEF1-Cas9 and pU6-sgAlb. Previous reports indicated low efficiency in animal models of gene-editing elements integrating into the host genome [36]. The editing plasmids were not cleaved by the introduced Cas9-sgRNA. However, small residual pieces may find their way to the DSBs at the Alb locus during the degradation of circular plasmids. We observed an approximately 1000-fold lower insertion frequency for these DNA remnants (Fig. 7B). For the pEF1-Cas9, the most frequently integrated DNA lengths were less than 500 bp or around 3 kb (Fig. 7G, H). For the U6-sgRNA, 300 bp was the most commonly inserted (Fig. 7I, J).

In summary, consistent with other reports, any dsDNA elements introduced into cells may lead to unintended integration at DSBs, albeit at a low frequency of 0.1-1% compared to the predominant insertion events.

### Analysis of endogenous genomic DNA fragments capture at the Alb editing site

In addition to insertions of gene-editing elements, unintended insertions of endogenous genomic DNA fragments can occur during editing [20, 23]. We aimed to evaluate the insertion levels of these genomic DNA fragments at the Alb editing site. To do this, we developed an algorithm to align the mouse genomic sequence with our sequencing data. To minimize false positives, we filtered out matches to the Alb gene on chromosome 5 and the F8 gene on chromosome X due to their significant homology to the exogenous F8 donor sequence. We discarded aligned sequences shorter than 50 bp. For reads with multiple chromosome alignments, we selected the source chromosome with the highest identity to the reads (Supplementary Figure S10).

**Fig. 8:**
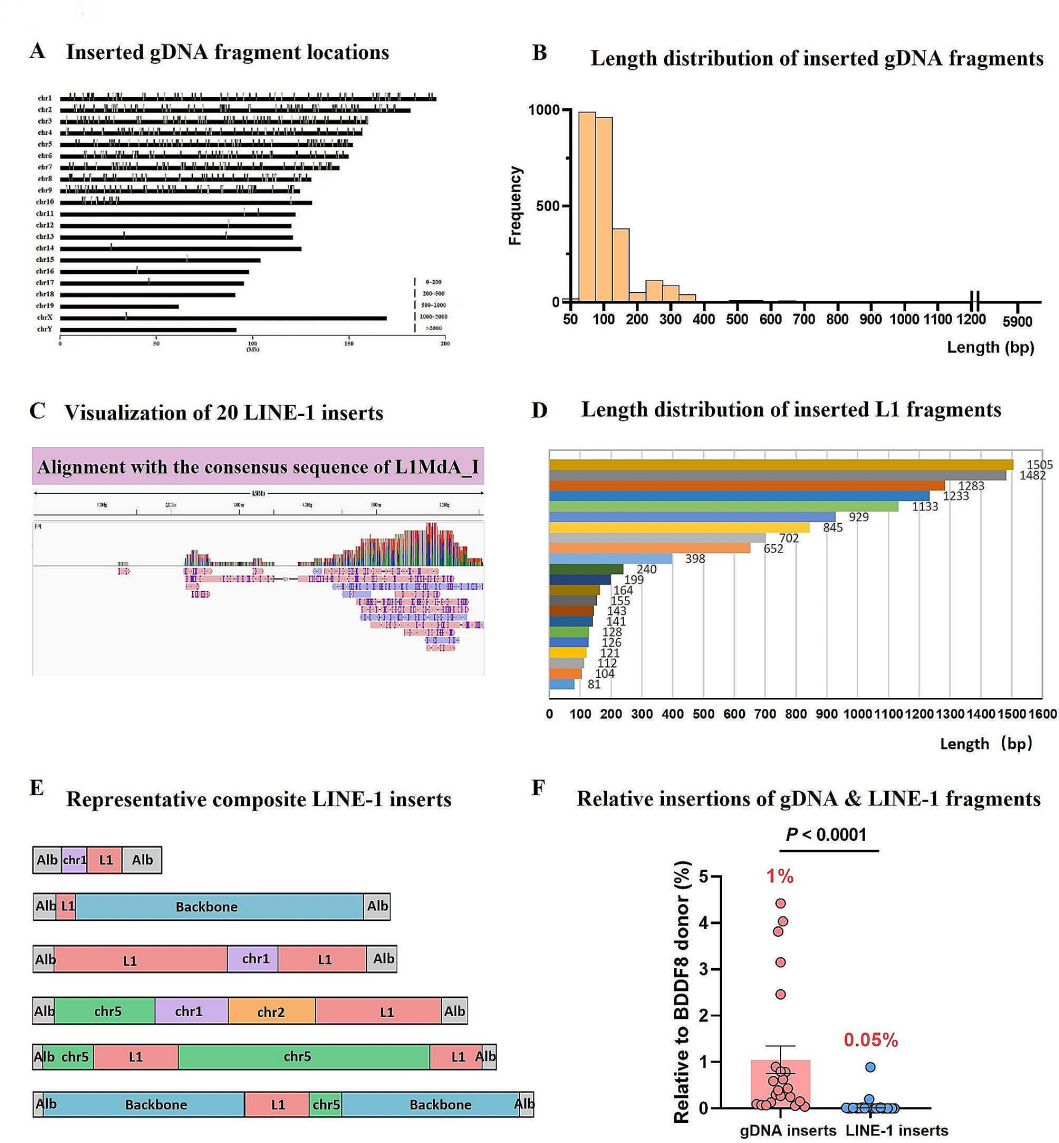
Analysis of genomic DNA and LINE-1 fragment integration at the edited Alb site. (**A**) Insertion locations of detected genomic DNA: The original sites of inserted genomic DNA sequenced in this study are represented by bars, colored according to length. (**B**) Genomic DNA insertion length distribution: The captured genomic DNA at the CRISPR-cleaved Alb site ranges from 50-400 bp, with the longest insertion measuring 5912 bp. (**C**) LINE-1 insertion visualization: 20 LINE-1 inserted sequences are displayed, with multiple tracks represented in a single line. (**D**) Length distribution of LINE-1 insertions: Analysis of 22 LINE-1 inserted sequences. (**E**) Complex insertion schematic: Representative complex insertions, including LINE-1, genomic DNA fragment, and plasmid backbone sequence, are illustrated. (**F**) Relative abundance comparison: The frequency of genomic DNA fragments and LINE-1 sequences is compared to double-cut donor inserts. Error bars represent mean ± SEM, based on data from 23 mice. Paired two-sided Student’s t-tests were used

Our analysis pipeline identified 416 unique inserts originating from genomic DNA fragments. These genomic fragments were dispersed throughout almost all mouse chromosomes (Fig. 8A; Supplementary Figure S11). Detailed sequence analysis of 50 representative genomic inserts is presented in Supplementary Figure S11. We detected various types of inserts, including single genomic DNA fragment inserts (Supplementary Figure S11A), multiple distinct genomic DNA fragment inserts (Supplementary Figure S11B), tandem inserts of backbone and genomic fragments (Supplementary Figure S11C), tandem inserts of F8 and genomic elements (Supplementary Figure S11D), and tandem inserts of backbone, F8, and genomic fragments (Supplementary Figure S11E).

Insert length analysis showed that nearly 98% of the genomic inserts were less than 400 bp (Fig. 8B), with the longest insert measuring 5912 bp. Additionally, inserted backbone tandem sequences ranged from 213 to 2171 bp, while F8 tandem sequences were 1272 and 2183 bp in length. Compared to inserting a single gDNA fragment (1%), tandem integration occurred at a 300-times lower frequency (0.003%).

### Analysis of LINE-1 sequence capture at the editing site

LINE-1 (L1) is a retrotransposon that constitutes over 20% of the human and mouse genomes, composed of two open reading frames (ORFs) - ORF1 and ORF2 [37]. Prior research has documented numerous de novo L1 insertions at multiple CRISPR-Cas9 editing sites [24]. Our study focused on the youngest LINE-1 element (L1MdA_I) as the reference sequence for our analysis (Supplementary Figure S12). Our approach for detecting LINE-1 inserts paralleled that used for genomic inserts.

From millions of sequencing reads, we detected 20 reads containing LINE-1 insertion. These included nine single LINE-1 inserts (Supplementary Figure S13A), six tandem inserts of LINE-1 and genomic fragments (Supplementary Figure S13B), three tandem inserts of backbone and LINE-1 fragments (Supplementary Figure S13C), and two tandem inserts of backbone, genomic, and LINE-1 fragments (Supplementary Figure S13D). Alignment of these 20 inserts with the L1MdA_I reference sequence showed that all were truncated (Fig. 8C). Length analysis revealed that the longest L1MdA_I insert detected measured 1505 bp (Fig. 8D). We observed complex inserts comprising backbone, genomic DNA insertions, and L1MdA_I (Fig. 8E), suggesting potential multiple insertions and rearrangements at the editing site. The ratio of LINE-1 inserts relative to donor plasmid fragments was 0.05%, mirroring the proportion of the LINE-1 sequence in the genome (Fig. 8F). Detailed sequence analysis is provided in Supplementary Figure S13.

Our results highlight the complexity of gene editing outcomes and the potential for unintended insertional events. LINE-1 retrotransposons, which represent a significant portion of the genome, adds a layer of complexity to the gene editing landscape. Previous studies have shown the ability of LINE-1 elements to insert into new genomic sites [37], and our analysis detected rare insertions of LINE-1 elements at CRISPR-Cas9 editing sites.

## Discussion

Our study provides a detailed analysis of DNA integration at the Alb target site in a hemophilia A mouse model. By integrating barcoded long-range nested PCR, magnetic bead size selection, and nanopore sequencing, we enhanced the precision of gene editing outcome analysis. In our experimental conditions, we noted the presence of full-length and multiple insertion patterns from a double-cut donor in the liver. Additionally, we observed lower frequency integrations, including partially degraded F8 cassettes and plasmid backbones (3% relative to intact insertions), CRISPR plasmid fragments (0.1%), pieces of endogenous genomic DNA (1%), and LINE-1 elements (0.05%). Notably, we detected insertions exceeding 10 kb from various sources, illustrating the intricate nature of DNA repair and integration processes. This study not only confirms the potential of CRISPR-Cas9 gene therapy for hemophilia A but also highlights the critical importance of comprehensive understanding and stringent quality control in gene editing applications.

This study represents a significant advancement in CRISPR-Cas9 research, being the first to comprehensively detail full-length insertions in an in vivo setting. This contributes significantly to our understanding of gene editing complexities in live organisms. Our initial efforts revealed the difficulty in reducing dominant background alleles and enhancing the presence of low-level edited alleles with insertions. To address the challenge of biased amplification, we concentrated on the PCR amplification of short genomic regions adjacent to the inserted donor DNA. This targeted approach allowed for more accurate quantification and analysis of editing outcomes. However, the significant size discrepancy between wild-type and modified loci led to biased amplification towards shorter amplicons, complicating data quantification. To counter this, we implemented size selection using magnetic beads and in vitro RNP cleavage, targeting unedited alleles and excessively amplified plasmid backbone insertions. This strategy effectively reduced background noise and narrowed the quantitative disparity.

Additionally, the integration of long-range PCR with nanopore sequencing shed light on the intricacy of editing outcomes involving CRISPR and extended donor templates. Diverging from prior studies that primarily used Southern blot analysis—dependent on length as the main factor [22, 38]—our study delved into gene editing outcomes at the single nucleotide level via long-read sequencing technology. This approach is particularly crucial in experiments with only about ~ 1% donor gene insertion efficiency.

Our findings extend beyond the anticipated forward F8 cassette insertions. We identified various integration events, including reverse insertions of F8 cassettes and bidirectional integrations of the plasmid backbone. These results highlight the complex dynamics of CRISPR-Cas9 editing beyond conventional expectations. Such unintended template DNA insertions via CRISPR-Cas9 have been documented across several organisms [21, 22, 39, 40]. In our design, donor plasmids could break into two parts: the F8 cassette and backbone, both susceptible to integration at the target site (Supplementary Figure S15). Thus, single-piece F8 or backbone insertions comprised over 80% of outcomes. Despite the expectation of low frequency for multiple donor insertions, we noted both F8 and backbone present in over 10% of cases, a rate significantly higher than other compound insertions. This could be due to the double-cut design and CRISPR-Cas9 cleavage and repair dynamics.

A recent study highlighted frequent concatemerization, especially head-to-tail insertions, in CRISPR editing using circular plasmids [41]. While Southern blot analysis can identify unintended insertions in individual clones, our approach offers broader insights, capturing various subtle insertion patterns within bulk populations. We also explored the editing outcomes using a circular donor plasmid with HA600-600 homology arms (Supplementary Figure S16; Supplementary Table S9). Despite attempts to enrich long PCR products, as outlined in Fig. 2B, nanopore sequencing failed to detect any F8 insertions among over 6,000 reads. This lack of detection is likely due to the preferential amplification of short background sequences (1.3 kb) without insertions. Therefore, we focused our subsequent analysis on the insertion patterns of double-cut donors with shorter homology arms.

We observed mostly complete insertions of backbone fragments and shorter F8 or backbone inserts, comprising less than 80% of full length, with an insertion ratio of 1–2% relative to the F8 donor. Notably, about 5% of these insertions resulted in deletions over 100 base pairs, suggesting potential DNase-mediated degradation post-linearization. These deletions could impair F8 expression, primarily through frameshifts or incomplete cDNA/protein sequences. This highlights the need for more precise gene editing strategies. However, these unexpected outcomes didn’t seem to negatively impact the host, as evidenced by stable unintended insertion ratios in mice sacrificed at different post-editing intervals.

Previous research has shown that transfected plasmid DNA can patch I-SceI-induced DNA DSBs, demonstrating that DSBs can be repaired through DNA sequence insertion [42–44]. Our extensive analysis indicates that NHEJ is the predominant mechanism for DSB repair. NHEJ favors nearby DNA fragments for insertion, with more abundant pieces preferentially integrated. In our study, F8 and backbone fragments showed the highest insertion rates among the introduced plasmid vectors in hepatocytes, followed by genomic fragments and circular CRISPR plasmid elements. We also noted sporadic insertions of two or three segments, suggesting these are rare events. This indicates that repairing one DSB end might allow for the connection with various DNA fragments over time.

In some therapeutic scenarios, replacing editing plasmids with CRISPR ribonucleoproteins (RNPs) could reduce unintended plasmid integrations. CRISPR RNPs offer greater precision, fewer off-target effects, and improved safety, making them appealing for diverse therapeutic applications. Nonetheless, the choice between RNPs and plasmids should be tailored to the specific therapeutic requirements.

DSB repair has played a crucial role in the evolution of eukaryotic genomes by facilitating the capture and integration of foreign DNA elements like retrotransposons, plasmid segments, and moderately repetitive sequences [24]. Our research supports this, revealing frequent, random insertions of DNA fragments, from single to multiple pieces, at the CRISPR target site [45]. Our study in hemophilia A mice underscores the necessity of in vivo assessment of gene editing results, with almost 99% of insertions stemming from the introduced double-cut donor template. The rest included genomic insertions, LINE-1 elements at 0.02%, and circular CRISPR plasmid segments at 0.1%. As previously documented, various sequences, including those from the target and non-target chromosomes and plasmid DNA, can integrate into target sites [23], presenting potential safety concerns despite their low frequency.

The CRISPR system activates two primary cellular repair pathways: NHEJ and HDR. However, HDR events are usually less common than NHEJ [46]. Our study found that varying homology arm lengths (HA600-600, HA190-130, HA190-0, HA85-130, HA85-0) didn’t significantly affect HDR efficiency. This insight is crucial for optimizing homology arm lengths to enhance in vivo HDR editing. For the forward insertions of five double-cut donors, NHEJ and HDR were equally involved in repairing both junctions, with HDR contributing to about 25% of all events. In comparison, our previous investigation showed that NHEJ accounted for 40 ~ 70% of all knock-in events [11].

CRISPR-induced DSBs can lead to indels and large deletions. In our study, regular PCR followed by NGS assessed gene editing efficiency, which ranged between 6 and 11% in vivo. We classified large deletions as those exceeding 100 bp (D100) [47], finding them relatively infrequent. Large deletions were less common in hepatocytes than in hematopoietic cells, especially during in vivo liver editing, as most hepatocytes were not actively dividing. This suggests that large deletions may vary significantly across different cell types and stages of the cell cycle.

## Conclusions

Our research underscores that genotoxic integration events are a significant safety concern in CRISPR-mediated gene therapy, particularly with techniques involving dsDNA cleavage and donor integration. We presented a novel methodology to assess the occurrence of extended insertions and unintended genomic integrations. Although our gene-edited hemophilia A mice model showed no immediate adverse effects, the discovery of diverse and complex integration patterns underscores the critical need for ongoing research. Investigating the long-term safety implications of these unintentional integrations is paramount for the responsible advancement of CRISPR-mediated therapies. It is imperative to continue research to fully understand the functional impacts of these inadvertent genomic alterations. Such knowledge is essential for evaluating the safety of in vivo gene editing techniques and developing more secure gene therapy approaches.

## Methods

### Cas9 and sgRNA plasmid construction

The crRNA and sgRNA sequences were designed using the CHOPCHOP website (https://chopchop.rc.fas.harvard.edu/). The sequences of all the sgRNAs utilized in this study can be found in Supplementary Table S1. The U6 promoter drove the sgRNA targeting the Alb locus and the double-cut donor, while the EF1 promoter controlled Cas9 protein expression. The Cas9 and sgRNA plasmids were constructed following established protocols using the NEBuilder HiFi DNA Assembly Kit (New England Biolabs) [11]. Endonuclease digestion and Sanger sequencing (Tsingke Biotechnology) were performed to verify the constructed vectors’ accuracy.

### Hydrodynamic injection of editing plasmids

The current study employed a well-established hemophilia A mouse model, previously described in the literature [11]. The mice were maintained at the State Key Laboratory of Experimental Hematology (SKLEH) in Tianjin, China. All animal experiments adhered to the Institutional Animal Care and Use Committee of SKLEH and the Institute of Hematology guidelines. For the experimental groups, eight hemophilia A mice at 6–10 weeks of age received a tail vein injection of CRISPR plasmid (pEF1-Cas9 and pU6-sgAlb), sgDocut plasmid (pU6-sgDocut), and donor plasmids with varying lengths of homology arms (pD-BDDF8-sg with HA600-600, HA190-130, HA190-0, HA85-130, HA85-0) at 10 µg each, dissolved in sodium lactate Ringer’s solution (China Otsuka Pharmaceutica) with a volume equivalent to 10% of their body weight. The hydrodynamic delivery was completed within 5–6 s. In our experimental groups, two mice succumbed following the hydrodynamic injection of editing plasmids. Such mortality is not unusual in hydrodynamic injection procedures due to the transient physiological stress they can induce. Diverging from previous methodologies that utilized a single sgRNA for targeting both the Alb locus and the double-cut donor [11], our study implemented distinct sgRNAs for targeting the Alb (sgAlb) and F8 donor (sgDocut) loci, as detailed in Supplementary Table S1.

### Peripheral blood collection and plasma isolation

As detailed in a previous study [11], blood collection was performed by obtaining 100 µl of venous blood from the lateral tail vein clip into a 1.5 ml centrifuge tube, with 10 µl of 3.2% sodium citrate added as an anticoagulant. The bleeding was promptly stopped using styptic powder (Miracle Corp). The blood samples were centrifuged at 2000 ×g for 15 min at 25 °C, allowing plasma separation from blood cells. The plasma supernatant was transferred to a new tube and stored at -80 °C for future analysis. To measure F8 bioactivity, plasma samples were rapidly thawed at 37 °C to prevent coagulation factor degradation.

### F8 coagulant factor activity assay

Factor VIII coagulation activity (FVIII:C) was assessed using a one-stage clotting assay on a Sysmex CA1500 system analyzer (Sysmex, Kobe, Japan). First, plasma samples were diluted fourfold and tested with the aPTT reagent (Dade Actin activated cephaloplastin reagent; Siemens; B4218-1) and factor VIII deficient plasma (Siemens; OTXW17) from Siemens (Siemens; Marburg, Germany), with a normal range of 74–112%. During the measurement, 5ul of diluted mouse plasma sample was mixed with 45 µl of Dade Owren’s Veronal Buffer (Siemens; B4234-25), 50 µl of factor VIII deficient plasma, and 50 µl of aPTT reagent. The mixture was incubated at 37 °C for 120 s to generate factor XIa. Subsequently, 50 µl of 25 mM calcium chloride was added for factor IXa and thrombin generation, leading to clot formation. The Sysmex CA1500 system recorded the clot formation time, which was compared to a standard curve obtained by diluting human calibration plasma (Siemens).

### Cell culture

Hepa 1–6 cells, obtained from Procell (Wuhan Procell Biotechnology Co., Ltd.), were cultured in Dulbecco’s Modified Eagle Medium (DMEM, Gibco) supplemented with 10% fetal bovine serum (FBS, Gibco) and 1% penicillin-streptomycin (Gibco) at 37 °C in a 5% CO_2_ humidified incubator. Cells were passaged every 2–3 days using 0.25% trypsin-EDTA (Gibco) and were utilized for experiments between passages 5–10.

### RNP formation and DNA in vitro cleavage

The synthetic crRNA targeting the Alb gene (crAlb), trans-activating crRNA (tracrRNA), and SpCas9 nuclease (Alt-R S.p.Cas9 Nuclease V3) were obtained from Integrated DNA Technologies (IDT). To form sgRNA (sgAlb), crAlb and tracrRNA were mixed in equal molar amounts and diluted in 5X Annealing Buffer (Synthego). The mixture was subjected to a temperature profile of 78 °C for 15 min, 37 °C for 30 min, followed by cooling to room temperature for 15 min. Ribonucleoprotein (RNP) complexes targeting Alb (RNP-sgAlb) were generated by incubating Cas9 protein with sgAlb at a 1:3 molar ratio for 10 min at room temperature. RNP complexes targeting plasmid backbone (RNP-sgBB) were composed similarly when indicated. These RNP complexes were used for in vitro cleavage experiments and Hepa 1–6 cell editing.

### Transient transfection for genome editing

Hepa 1–6 cells were transfected with RNP-sgAlb using nucleofection. The cells were harvested, and 1 × 10^6 cells were electroporated with the Amaxa 4D Nucleofector (program CM138) and the P3 Primary Cell Nucleofector Kit (V4XP-3032). The transfected cells were immediately transferred to a pre-warmed complete growth medium and incubated at 37 °C with 5% CO2. Three days later, the cells were harvested for further analysis.

### Small indel analyses by next-generation sequencing (NGS)

Primers designed with Primer3Plus were used to amplify ~ 240 bp fragments surrounding the on-target sequences for Illumina paired-end 150 bp sequencing (Supplementary Table S2). PCR was conducted using KAPA HiFi polymerase with the following cycling conditions: 98 °C for 1 min, followed by 25 cycles of 98 °C for 5 s, 64 °C for 10 s, and 72 °C for 10 s. Barcoded PCR amplicons were pooled equimolarly and sequenced using Illumina’s NovaSeq6000 System (Novogene). Novogene performed library construction and raw data acquisition. The acquired data was merged using Flash [48], demultiplexed with Barcode-splitter (https://pypi.org/project/barcodesplitter/), and subsequently analyzed for indel efficiencies using CRISPResso2 [49].

### Long-range PCR of genomic DNA

Long-range PCR was performed on genomic DNA extracted from liver samples using the Puregene Cell and Tissue Kit (Qiagen) with PrimeSTAR GXL DNA polymerase (Takara Bio). To ensure the representativeness of results for each mouse, 2–3 replicates of 20 µl PCR reactions were used, with each reaction containing 400 ng of genomic DNA and 0.5 µM primers. The first long-range PCR was conducted with cycling conditions of 98 °C for 1 min, followed by 25 cycles of 98 °C for 10 s, 64 °C for 15 s, and 68 °C for 6.5 min. Barcode-containing primers were used to amplify DNA from size-selected first PCR products for nanopore sequencing. In addition, indel-correcting 11-nt DNA barcodes were also used to prevent sample misalignment during demultiplexing of pooled nanopore sequencing reads [50]. The second PCR thermal cycler program was as follows: 98 °C for 1 min, followed by 20 cycles of 98 °C for 10 s, 64 °C for 15 s, and 68 °C for 6.5 min. All PCR products were visualized by electrophoresis on 1% agarose gel.

### Gel extraction for DNA fragments with insertions

We performed gel extraction on 50–100 µl PCR products containing representative DNA alleles to isolate DNA fragments with insertions. The PCR products were separated on a 1% agarose gel, and the gel region predicted to have pieces with insertions was excised. Next, purification was performed using a gel extraction kit (TransGen Biotech), and the DNA fragments of interest were dissolved in 50 µl elution buffer. Purified products were collected by centrifugation and were ready for sequencing.

### Depleting background Alb alleles by in vitro RNP cleavage

For in vitro cleavage, a reaction mixture was prepared to contain either genomic DNA or purified 1st PCR products, Cas9-sgAlb, and 10X NEB3.1 buffer (New England Biolabs). The mixture was incubated at 37 °C for 1 h in a thermocycler to allow efficient cleavage of target DNA sequences by the RNP complex. After cleavage, the reaction mixture was subjected to magnetic bead-based size selection to enrich for long fragments (> 3 kb) and deplete small pieces and cleaved background alleles. The purified products were further amplified by PCR using barcode-containing primers for sequencing.

### Magnetic bead enrichment for purification of genomic DNA and PCR amplicons

We employed the Select-a-Size DNA Clean & Concentrator MagBead Kit (ZYMO Research) to purify genomic DNA and PCR amplicons according to the manufacturer’s instructions. Briefly, the appropriate volume of magnetic bead buffer was added to the PCR amplicon reaction and mixed thoroughly by vortexing until homogenous. Following a 2-min incubation, the sample was placed on a magnetic rack for 5 min. Once the beads were cleared from the solution, the supernatant was discarded. Next, the beads were washed twice with 200 µl of DNA wash buffer. Then, DNA elution buffer was added to the beads, and the sample was incubated at room temperature for 2 min. After a 5-minute incubation on the magnetic rack, the supernatant was transferred to a clean microcentrifuge tube, yielding DNA or PCR products ready for downstream applications.

### qPCR analysis

For qPCR, we used KAPA SYBR® Fast qPCR reagent (Sigma-Aldrich) to prepare reactions in a 96-well optical plate (Life Technologies). We added 0.5 µM primers and 1% of the first PCR product to a total reaction volume of 10 µl. The qPCR program consisted of 40 cycles, starting with 1 cycle at 98 °C for 2 min, followed by 98 °C for 5 s, 60 °C for 15 s, and 72 °C for 15 s. To identify specific products, melting curve analysis was performed with a 0.5 °C increment every 5 s from 60 to 95 °C.To assess F8 enrichment, we designed qPCR primers to amplify and quantitate both Alb and F8 amplicons. We used the ΔΔCt calculation relative to the background Alb amplicon copies to determine the relative F8 copy numbers.

### Long-range junction PCR

Four pairs of primers were designed, with one primer situated at the F8 cassette and the other beyond the homology arm, enabling the detection of both forward and reverse F8 insertions. Each PCR incorporated 400 ng of hepatic genomic DNA derived from mice administered CRISPR plasmids and double-cut donors with distinct homology arms. Four pairs of primers were applied to every mouse specimen. The PCR was conducted with cycling conditions of 98 °C for 1 min, followed by 30 cycles of 98 °C for 10 s, 62 °C for 15 s, and 68 °C for 7.5 min. The resulting amplicons were confirmed by 1% agarose gel electrophoresis and subsequently subjected to nanopore sequencing for further analysis.

### Nanopore sequencing

Long-range PCR amplicons were sequenced on an ONT MinION device using R9.4.1 chemistry (FLO-MIN106) and the 1D ligation sequencing kit (SQK-LSK110, ONT). First, the amplicons were purified using the Select-a-Size DNA Clean & Concentrator MagBead Kit (Zymo Research). Subsequently, the purified products were subjected to DNA damage repair and end repair using the NEBNext FFPE Repair Mix and NEBNext Ultra II End Repair/dA Tail Addition Module. Sequencing adapters were then ligated according to the manufacturer’s recommendations. The SpotON sequencing chip was pre-processed, and the libraries were sequenced for 12–36 h using the MinKNOW software on the R9.4.1 flow cell (GenoStarBio, China). The FAST5 data files were base-called and converted into FASTQ format using Guppy (ONT’s base-caller software) with default parameters. The resulting FASTQ files were then analyzed using various bioinformatic tools to assess the quality of the sequencing data, including read length distribution, per-base quality scores, and overall sequencing accuracy. The reads were aligned to the reference sequences using minimap2, followed by bioinformatics analysis detailed in the next section.

### Large deletion analysis - deletion indexes

To precisely quantify deletions, we employed deletion indexes. The ‘deletion index’ is defined as the discrepancy between the percentage of deletions in edited alleles and that in unedited alleles, which serve as the background reference. Here, ‘deletion in unedited alleles’ signifies the baseline level of small deletions detectable via Nanopore sequencing, occurring independently of gene editing. We determined the proportions of deletion by the formula: (read depth − mean depth) / read depth. This was executed using the “Samtools coverage file.bam” command from Samtools [30, 51]. Consistent with previous research, we classified deletions larger than 100 base pairs (bp) as substantial deletions, referring to them as ‘D100’ [12, 47].

### Length distribution analysis

We analyzed the length of nanopore sequencing reads by Seqkit bioinformatics packages [52] and depicted the length distribution using the command “seqkit watch --fields ReadLen file.fq.gz -O file.pdf”.

### Analysis of F8 or BB insertion patterns in NP reads

#### Data processing using the GREPore-seq workflow

To analyze the complex inserted sequences at the on-target edited locus, we employed the GREPore-seq pipeline that we recently reported [30]. Briefly, we began by demultiplexing the pooled nanopore sequencing data using stretches of barcoded sequences (BCseqs). These BCseqs consisted of 11-mer fragments generated with a step size of 1 nt from the 11 nt barcode sequence and the first 6 nt of the primers. We expected the inserted sequences to be flanked by 146 bp of Alb genomic DNA (gDNA) on the left and 186 bp on the right. Consequently, we employed similarly generated grepseqs to retrieve specific Alb amplicons with or without inserts.

#### Grouping data based on read length

Following the linearization of the double-cut donor, two distinct fragments were released: the F8 cassette (F) and the plasmid backbone (B). As multiple pieces can be captured at the Cas9-sgAlb target site, we investigated all possibilities involving integrating up to three components. Based on this assumption, we calculated the precise theoretical length of nine potential second PCR products. Subsequently, we grouped the demultiplexed data into nine files representing insertions of B (2506 bp), F (5858 bp), BF (8032 bp), BB (4680 bp), FF (11,384 bp), BBF (10,206 bp), BFF (13,558 bp), BBB (6854 bp), and FFF (16,910 bp), respectively.

Considering the ~ 5% sequencing errors (primarily indels) of nanopore sequencing and the partial degradation of donor fragments before integration, we expanded the interval by ± 20% based on the expected perfect insertion size when grouping reads. For instance, the perfect size of B inserts was 2506 bp; thus, we grouped reads ranging from 2006 to 3006 bp in length to analyze the insertion of a single backbone (Alb-Backbone-Alb).

#### Filtering data with grepseqs within restricted length ranges of datasets

We employed 20 17-nt grepseqs for backbone forward insertion (BBf), backbone reverse insertion (BBr), F8 cassette forward insertion (F8f), and F8 cassette reverse insertion (F8r) to filter reads within specific range intervals. Due to the ~ 5% sequencing errors, we arbitrarily narrowed the range intervals by 20% to reduce the retrieval of erroneous data. For instance, the reads with perfect insertion of B spanned from 147 to 2320 bp; thus, we searched for Bf or Br grepseqs at positions ranging from 447 to 2020 bp within the sequencing reads.

#### Calculate the proportion of reads with different insertion patterns

We divided the number of grepped reads by the total demultiplexed reads carrying both the left and right Alb background gDNA sequences to determine the proportion of specific insertion patterns. This approach allowed for an accurate assessment of the prevalence of each insertion pattern within the dataset. The grouped reads were then aligned to the predicted reference sequences, which consisted of Alb-left, 1–3 pieces of BB or F8 in forward or reverse orientations, and Alb-right. The data were sorted using the widely used Samtools software package for processing and analyzing high-throughput sequencing data [51]. Finally, we used the Integrative Genomics Viewer (IGV) to visualize the aligned reads and reference sequences, enabling a comprehensive examination of the insertion patterns identified in our study [34, 35].

### Analysis of insertion patterns of mice at 3 w vs. 3 m after in vivo editing

First, we harvested livers of mice at three weeks and three months after in vivo editing and extracted genomic DNA using the Puregene Cell and Tissue Kit (Qiagen). Then, we performed the first long-range PCR with the conditions described above and purified the products, followed by the cleavage with RNP-sgAlb and RNP-sgBB. After that, the secondary size selection and PCR were carried out to amplify the F8 inserts further. The subsequent amplicons were subjected to nanopore sequencing for comprehensive analysis. As detailed above, we analyzed the F8 and BB insertion patterns and summarized the single BB inserts, single F8 inserts, tandem F8 and BB inserts, and other patterns.

### Analysis procedure to identify reads with CRISPR plasmid inserts, short backbone inserts, and short F8 inserts

As previously described, we began by demultiplexing the amplicons using 11-mer BCseqs. Subsequently, we filtered the reads based on stretches of Alb k-mers generated with a window size of 17 nt and a step size of 20 nt, ensuring that the insertions occurred at the target site. Using the unique sequence of the Cas9 expression cassette, we created stretches of Cas9 k-mers using a window size of 17 nt and a step size of 100 nt. Similarly, we developed unique stretches of U6 k-mers for pU6-sgAlb plasmids with a window size of 17 nt and a step size of 40 nt. To determine the insertion of partially degraded BB and F8 (less than 80% of the full length), we searched for the BB k-mers in all reads shorter than 2 kb and the F8 k-mers in all reads shorter than 5 kb, using Seqkit bioinformatics packages [51]. Since the read length after the BB and F8 insertion of a full size is expected to be 2.5 kb and 5.7 kb, respectively, reads shorter than 2 kb and 5 kb could represent insertions of partially degraded BB and F8, respectively. By identifying these shorter reads, we could assess the presence of truncated BB and F8 insertions in our dataset.

### Analysis procedure to identify NHEJ or HDR-mediated donor insertions

We designed code to search for the corresponding homologous arm sequences using the Basic Local Alignment Search Tool (BLAST) and quantified the ratio of both repair pathways. Five pD-BDDF8-sg vectors with homology arms (HA600-600, HA190-130, HA190-0, HA85-130, HA85-0) flanked by Cas9-sgDocut recognition sequences were incorporated. First, the homologous arm sequences were aligned with the sequencing file, and reads with an identity score of 80 or more were selected. Next, the range of alignment was restricted to ensure that the homologous arms were located near the target site. Then, the length of the screened reads was limited to exclude artifacts caused by incomplete sequencing of Nanopore sequences. Finally, reads that matched up to two copies of homologous arms were considered “NHEJ,” while reads that matched up with only one homologous arm were considered “HDR.” Reads that did not meet either condition were considered “Others.” The reference sequence was generated by directly inserting the donor with homologous arms into the cleaved genomic sequence.

### Analysis procedure to identify reads with endogenous DNA or LINE-1 inserts

To determine the trace amounts of mouse endogenous DNA sequences captured at the Cas9-sgAlb target site, we aligned the sequencing dataset with the 332 bp Alb short amplicon sequence, mouse genomic database, or LINE-1 sequence using the Basic Local Alignment Search Tool (BLAST). Considering the high similarity between the human BDDF8 sequence and the mouse F8 gene located on chrX, and the ~ 150 bp sequences flanking the inserts amplified from the Alb site on chr5, we removed data within the Alb gene range on chr5 and F8 gene range on chrX. We defined the upstream of the sgAlb cut site as Alb-L and the downstream as Alb-R. The rightmost matched area of Alb-L and the leftmost matched site of Alb-R were abbreviated as Alb-L-r and Alb-R-l, respectively. To identify endogenous DNA sequences captured at the target site, we ensured that the matched sequences exceeded 50 nt and were flanked by Alb-L-r and Alb-R-l. This analysis allowed us to identify and characterize endogenous DNA or LINE-1 insertions within the CRISPR-edited genomic regions.

### Statistical analysis

GraphPad Prism 8.0.1 (GraphPad Software, San Diego, CA) was used to analyze experimental data. The mean ± standard error of the mean (SEM) was determined for each treatment group in individual experiments. Paired and unpaired two-sided Student’s t-tests were used to assess the statistical significance between the treatment and control groups. One-way analysis of variance (ANOVA) facilitated the comparison among multiple groups. The designation “ns” represents no statistical significance (*P* > 0.05).

### Electronic supplementary material

Below is the link to the electronic supplementary material.

Supplementary Material 1

Supplementary Material 2

## Data Availability

The sequencing data have been deposited in the NCBI under project accession number PRJNA956661 (https://www.ncbi.nlm.nih.gov/bioproject/?term=PRJNA956661) [53]. Any other materials supporting this paper’s findings can be obtained from the corresponding author upon reasonable request. The consensus sequences of L1MdA_I in the LINE-1 family are accessible via Repbase (http://www.girinst.org/repbase/). The code in this article has been deposited to Zenodo at 10.5281/zenodo.7852223 [54].
