# Supplementary material for: Decoding the complexity of on-target integration: characterizing DNA insertions at the CRISPR-Cas9 targeted locus using nanopore sequencing
Source: BMC Genomics. 2024 Feb 17;25:189. doi: 10.1186/s12864-024-10050-6 (PMC10874558; doi:10.1186/s12864-024-10050-6)
Supplement: Supplementary file 1 — Supplementary Material 1 [file 12864_2024_10050_MOESM1_ESM.pdf]

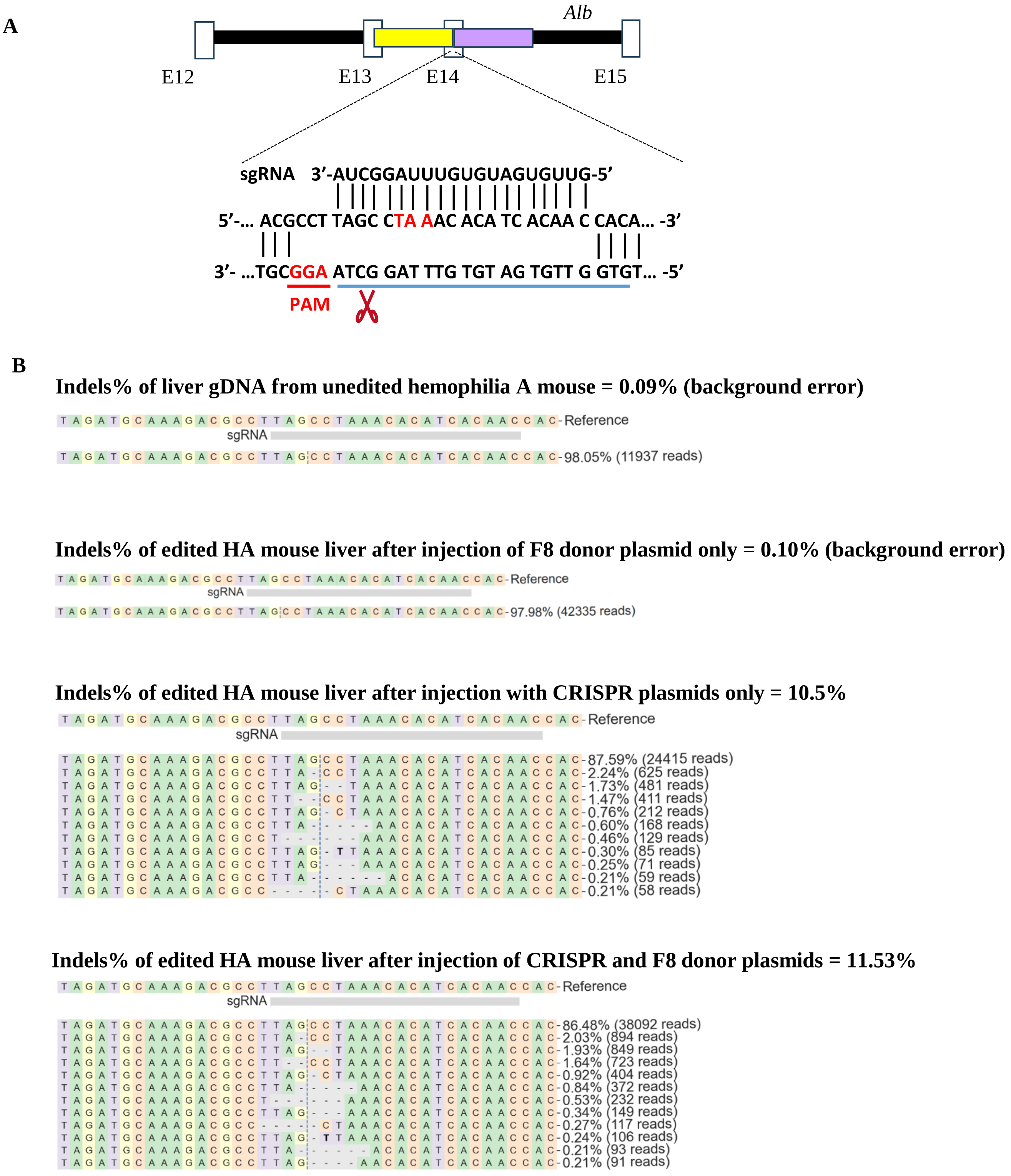

Figure S1. sgRNA Design and Indel Frequencies at the Alb Locus. (A) An sgRNA (sgAlb-E14) was designed to target the sequence surrounding the stop codon TAA (shown in red, reverse complement) in Exon 14 of the Alb gene. The red scissor icon indicates the anticipated Cas9 cleavage site, with "PAM" denoting the protospacer-adjacent motif (PAM) for SpCas9, recognized as NGG. (B) Frequencies of indels observed at the Alb locus following in vivo editing in the liver of hemophilia A mice. One week after the injection of editing plasmids, the sequence adjacent to the editing site was amplified from liver genomic DNA and subsequently analyzed using next-generation sequencing (NGS) and CRISPResso2.

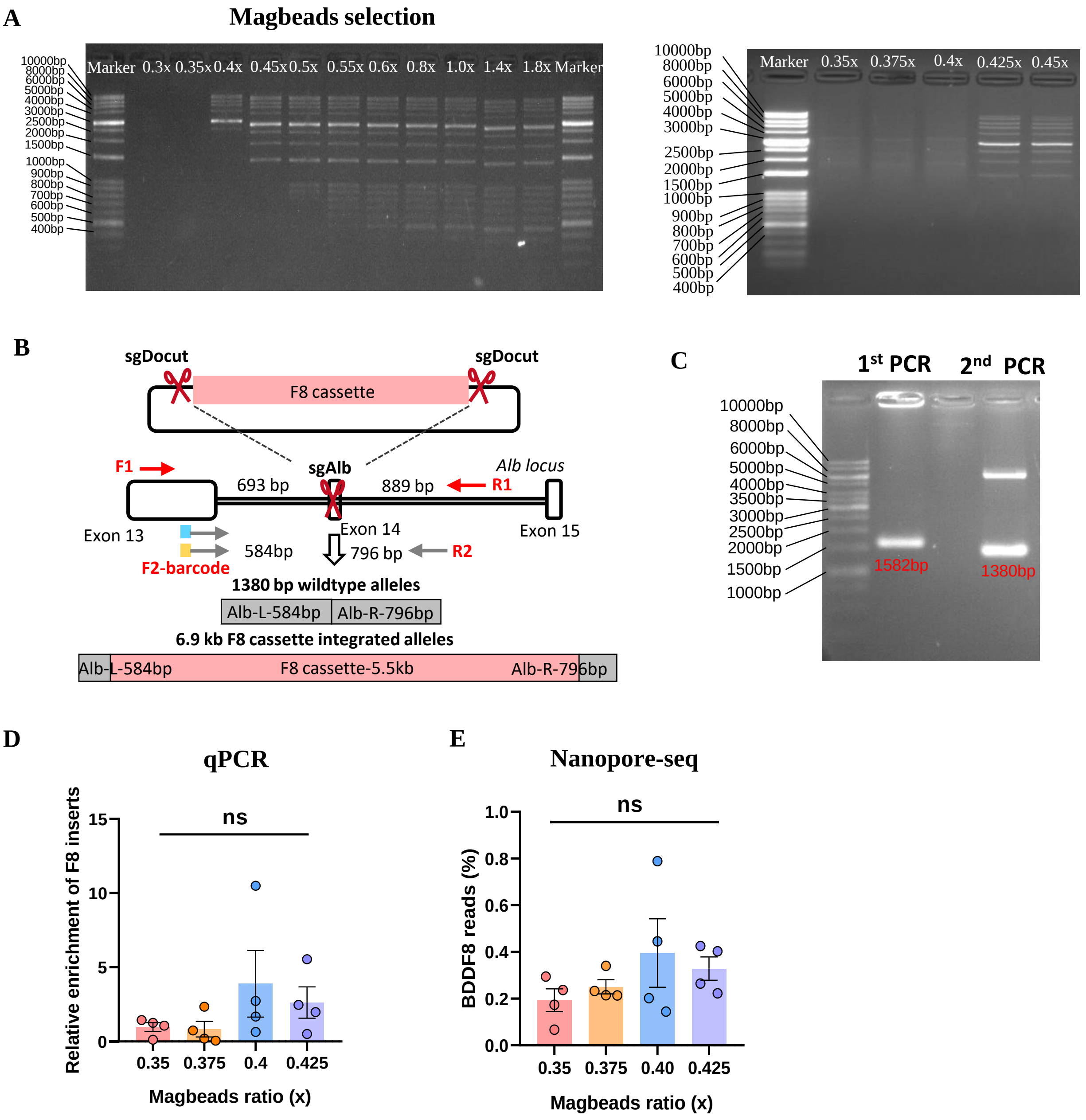

Figure S2. Optimizing Magnetic Beads for the Enrichment of F8-Integrated Alleles. (A) Evaluation of size selection efficiency using various ratios of magnetic beads (0.3x-1.8x). (B) Primer design for nested PCR, depicting expected PCR products, including the 1380bp wildtype alleles and the approximately 6.9kb F8 cassette-integrated alleles. (C) Representative results of gel electrophoresis for the 1st PCR products and the 2nd PCR products, which were amplified from the 1st PCR products after size selection using a beads ratio of 0.4x. The bands corresponding to alleles lacking donor insertion in the 1st and 2nd PCR products were 1586bp and 1380bp, respectively. (D) Relative enrichment of F8 inserts determined by qPCR after size selection using magnetic beads at various ratios. Error bars represent the mean ± SEM, n = 4, and paired two-sided Student’s t-tests were performed. (E) Determination of the percentage of both forward and reverse F8 inserts by nanopore sequencing (F8 reads %) in the 2nd PCR products following size selection of the purified 1st PCR products using different ratios of magnetic beads. Error bars represent the mean ± SEM, n = 4, and paired two-sided Student’s t-tests were conducted.

Size selection-None

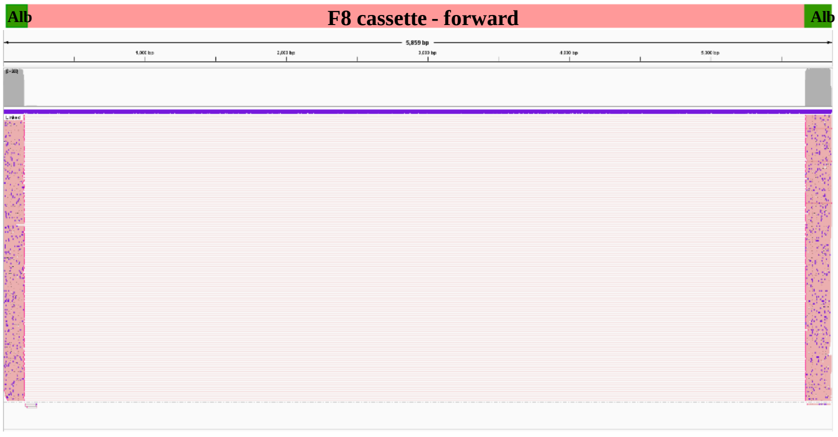

Size selection-Once

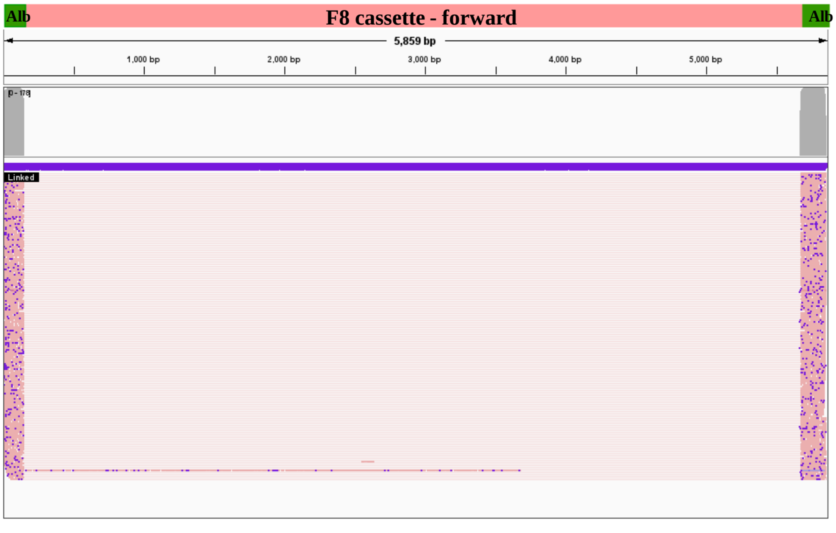

Size selection-Twice

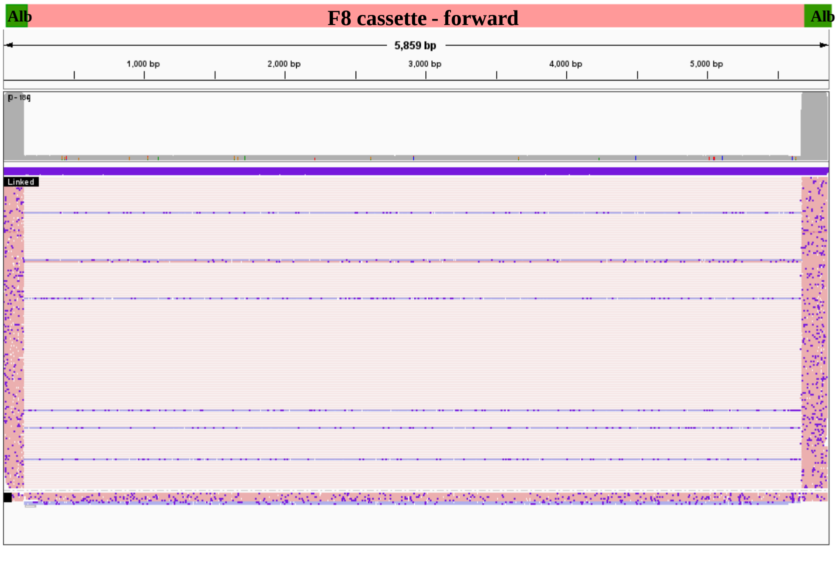

Figure S3. Visualization of Sequencing Results for 2nd PCR Amplicons after Magnetic Beads Size Selection. The reference sequence of the F8 cassette is flanked by 332bp of the Alb genomic sequence. Representative Integrated Genome Viewer (IGV) images display nanopore sequencing results after size selection of the 1st PCR products using magnetic beads 0 (above), 1 (middle), or 2 (bottom) times.

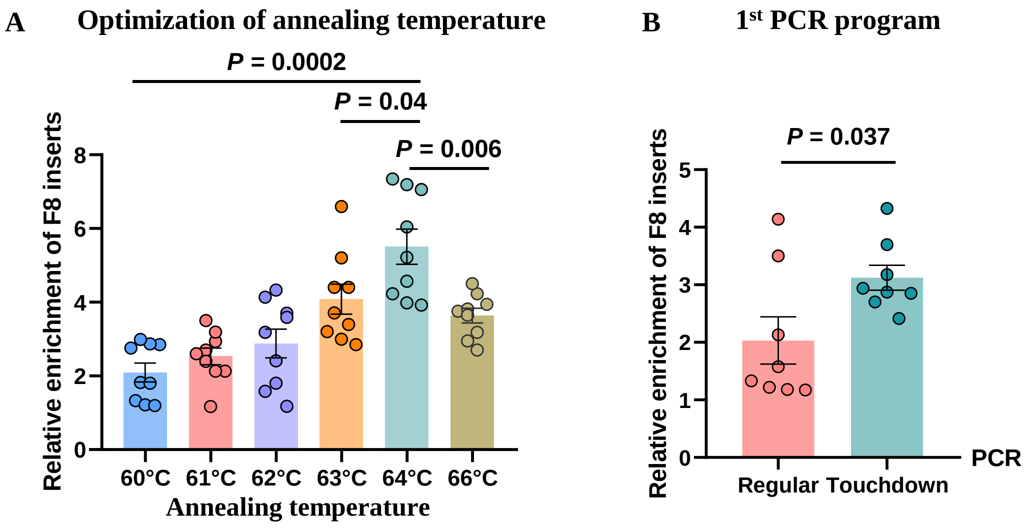

Figure S4. PCR Conditions Optimization for Enhanced Amplification of F8 Inserts. (A) Optimization of annealing temperature in the 1st PCR to improve the amplification of F8 inserts. The relative enrichment of amplicons with F8 inserts was determined using qPCR. Error bars represent the mean ± SEM, n = 9, and paired two-sided Student's t-tests were performed. (B) Employment of a touchdown PCR program in the 1st PCR to increase the amplification of F8 inserts. The touchdown PCR program used was 6 cycles of [98℃ for 10s, 68℃ (-1℃/cycle) for 15s, 68℃ for 6.5min]; followed by 25 cycles of [98℃ for 10s, 62℃ for 15s, 68℃ for 6.5min]. Error bars indicate the mean ± SEM, n = 8, and paired two-sided Student's t-tests were conducted.

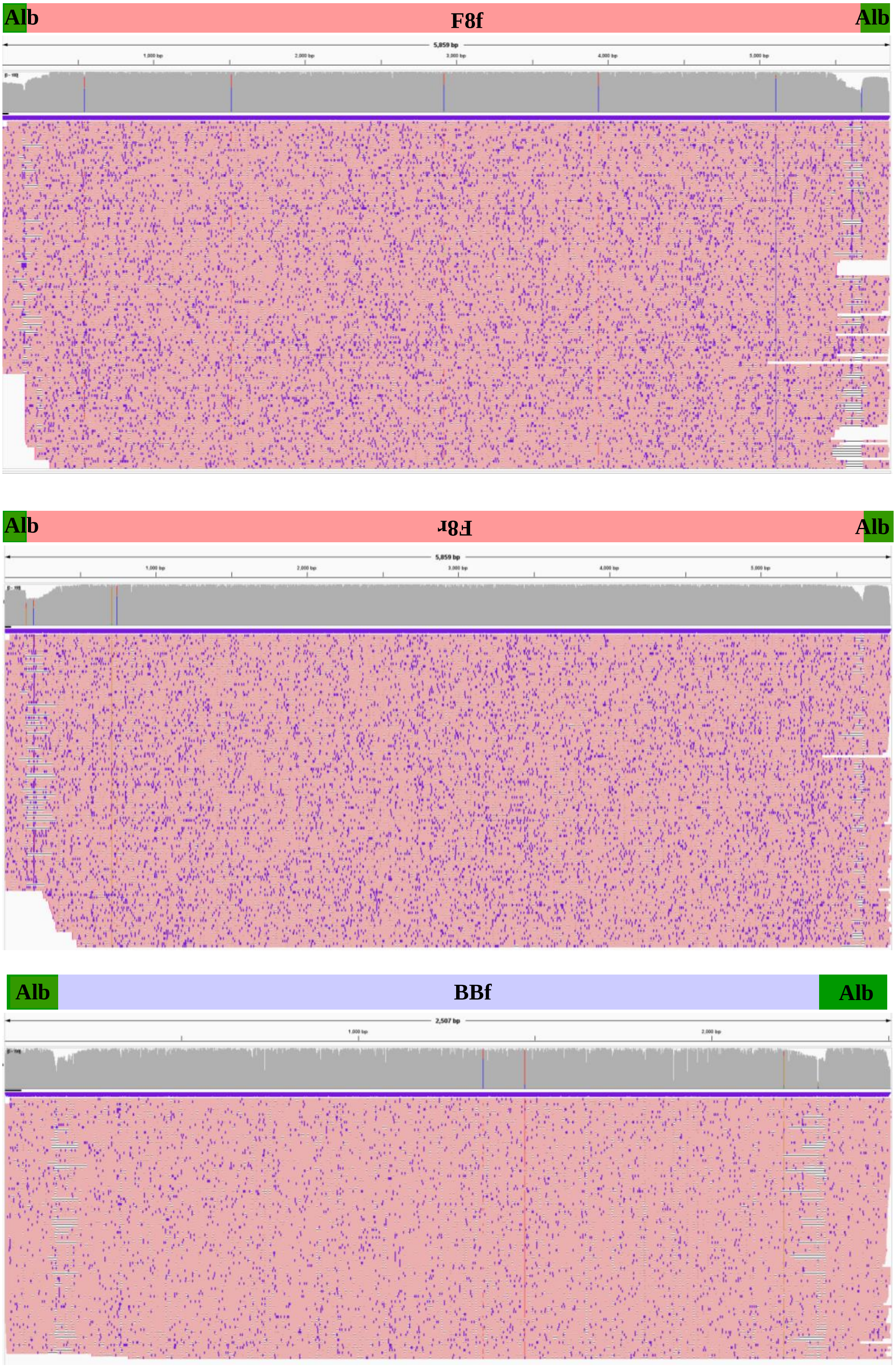

Figure S5. Random 200 reads in the visualization of eight representative insertion patterns of the donor template in Figure 5E. The F8 cassette is abbreviated as F8, and the plasmid backbone is abbreviated as BB. Forward insertions are abbreviated as f, and reverse insertions are abbreviated as r. (continued).

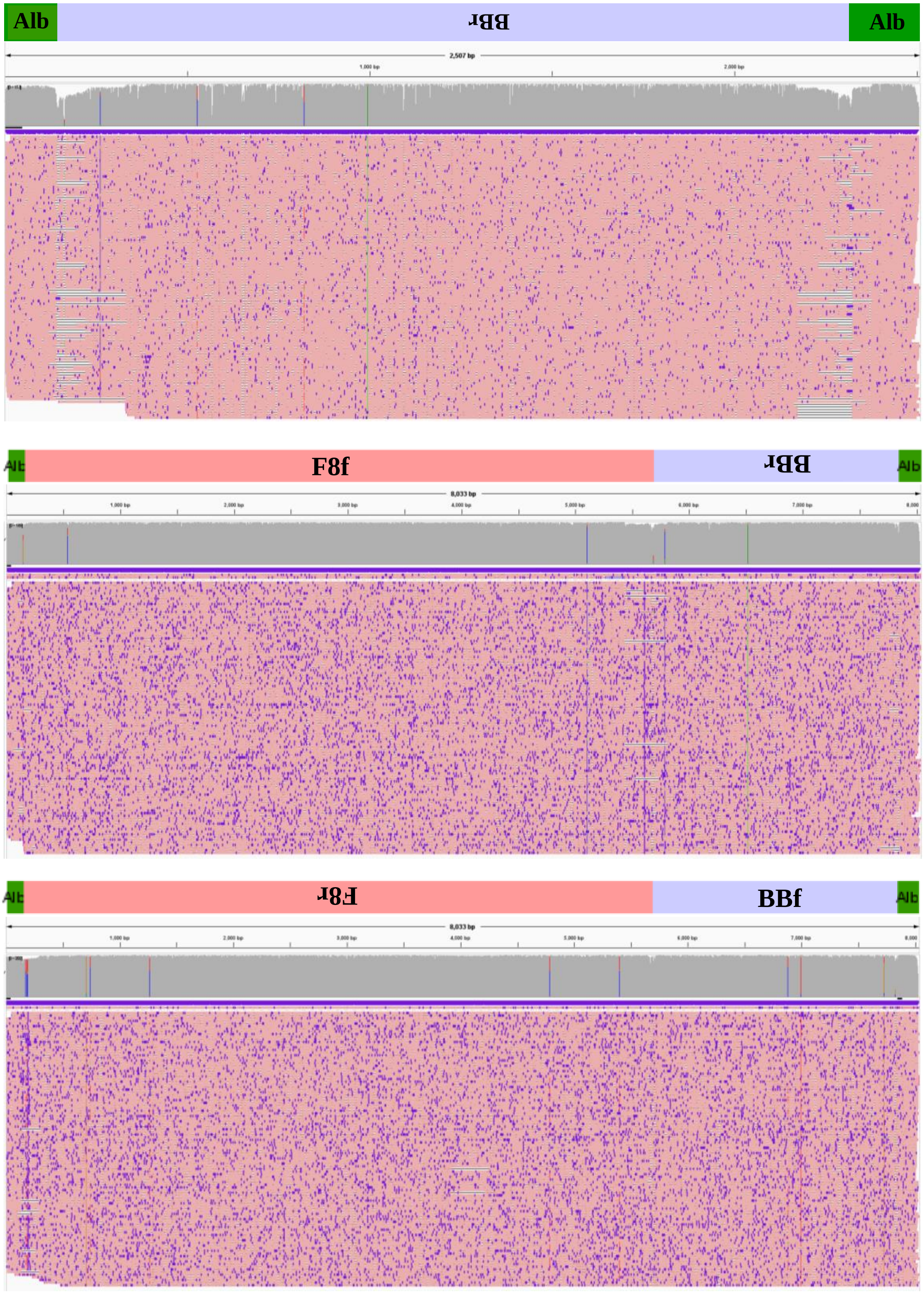

Figure S5. Visualization of Insertion Patterns: This figure presents the visualization of 200 randomly selected reads showcasing eight representative insertion patterns of the donor template, as depicted in Figure 5E. The insertions include components from the F8 cassette (abbreviated as F8) and the plasmid backbone (abbreviated as BB). The orientation of these insertions is also indicated, with forward insertions abbreviated as 'f' and reverse insertions as 'r'. This visualization aids in understanding the diversity and orientation of the insertions at the targeted genomic site. (Continued)

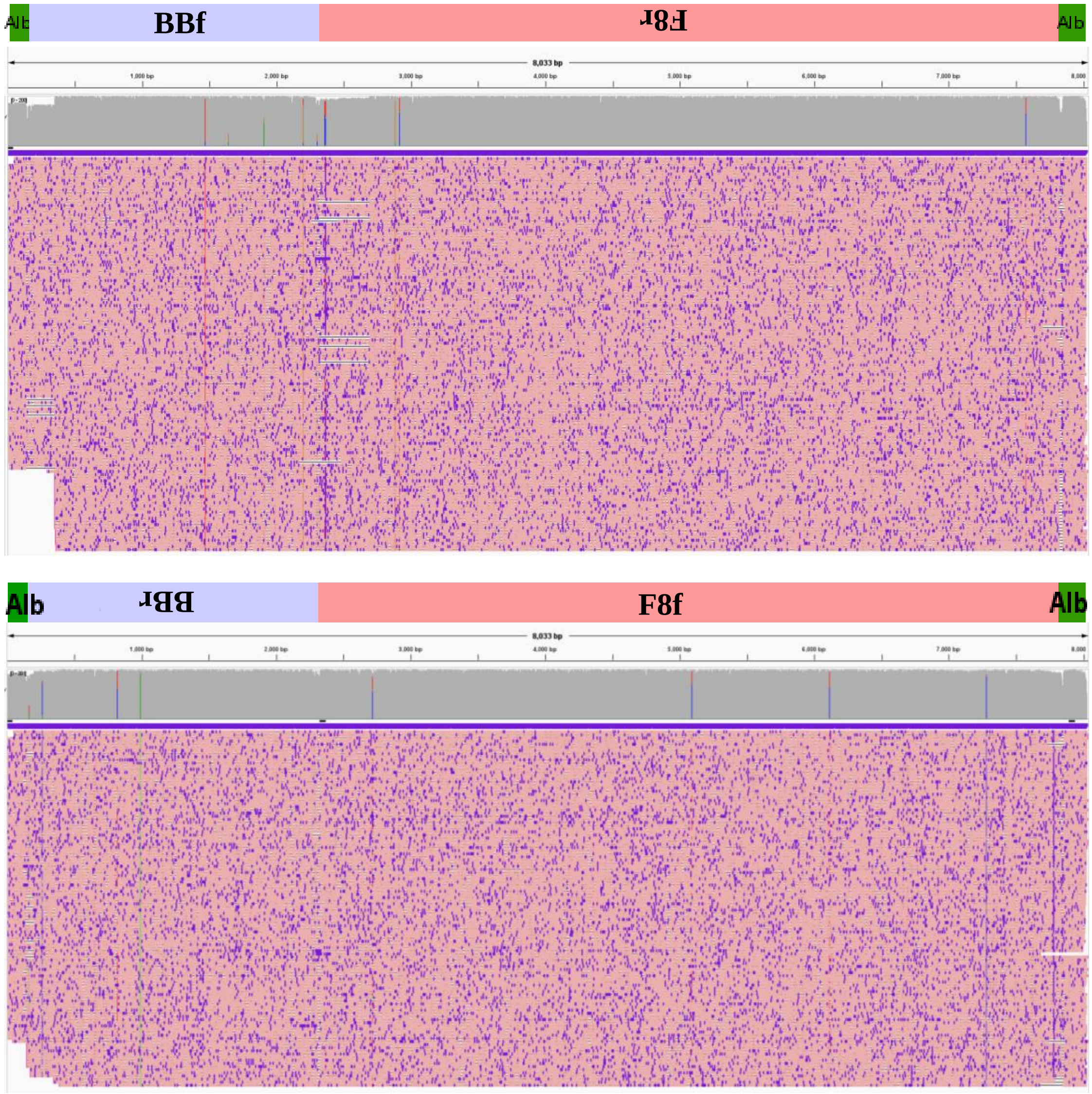

Figure S5. Visualization of Insertion Patterns: This figure presents the visualization of 200 randomly selected reads showcasing eight representative insertion patterns of the donor template, as depicted in Figure 5E. The insertions include components from the F8 cassette (abbreviated as F8) and the plasmid backbone (abbreviated as BB). The orientation of these insertions is also indicated, with forward insertions abbreviated as 'f' and reverse insertions as 'r'. This visualization aids in understanding the diversity and orientation of the insertions at the targeted genomic site.

A Schematic of insertions of multiple double-cut donor fragments

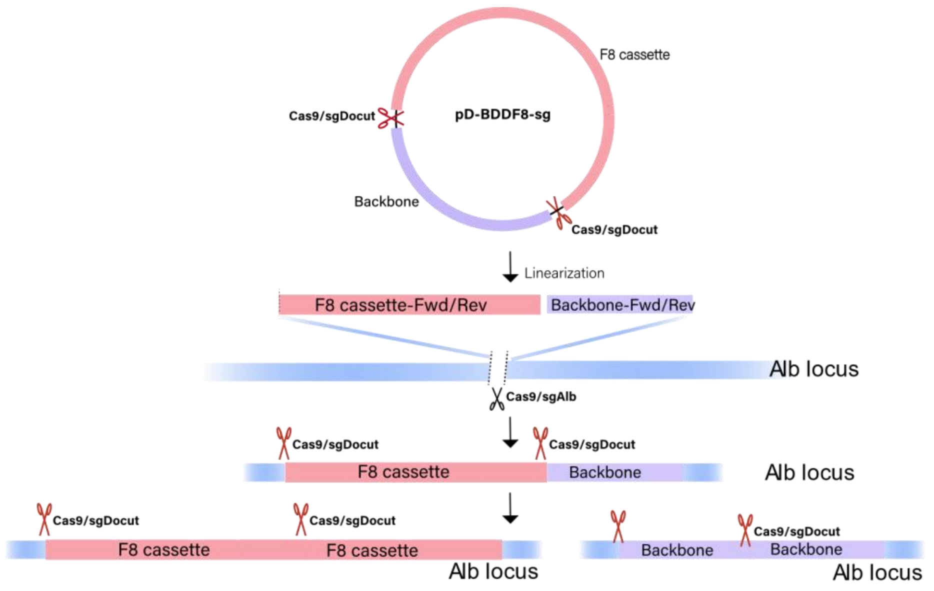

B Additional two-fragment integrations

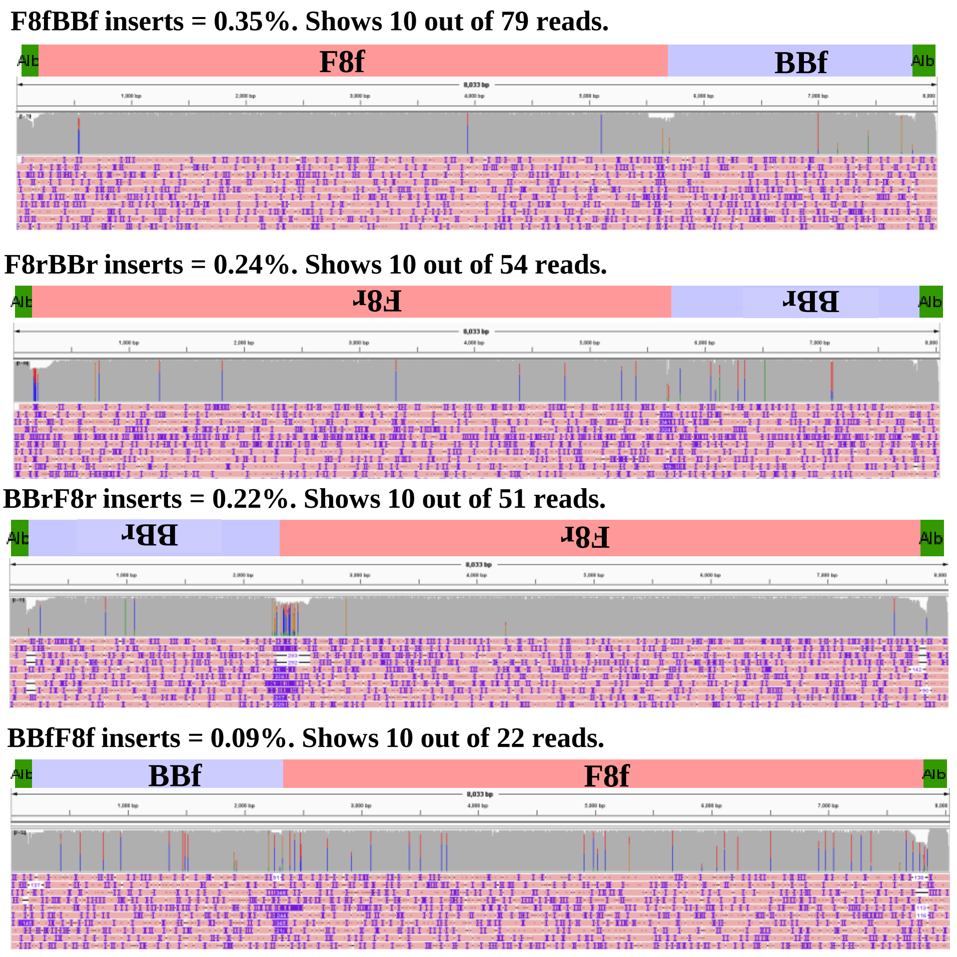

Figure S6. Characterization of Multi-Fragment Insertions from Double-Cut Donor Plasmid Following Cas9-sgRNA Linearization. The orientation of these insertions is indicated, with forward insertions abbreviated as 'f' and reverse insertions as 'r'. (A) Schematic representation of multi-fragment insertions resulting from the linearization of the double-cut donor plasmid. Following linearization, multiple fragments can integrate, albeit at lower frequencies. (B) Visualization of additional two-fragment integrations not shown in Figure 5, including four patterns of F8 and BB insertions in both orientations.

BBrBBr inserts = 0.22%. Shows 10 out of 49 reads.

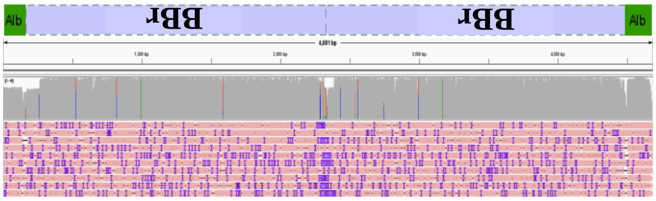

BBfBBf inserts = 0.20%. Shows 10 out of 46 reads.

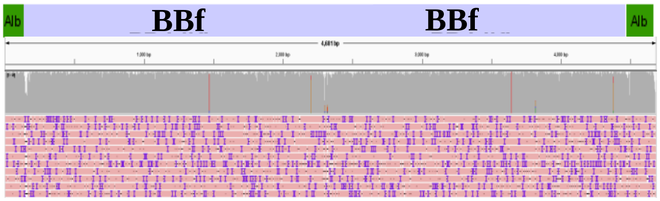

BBfBBr inserts = 0.04%. Shows 9 out of 9 reads.

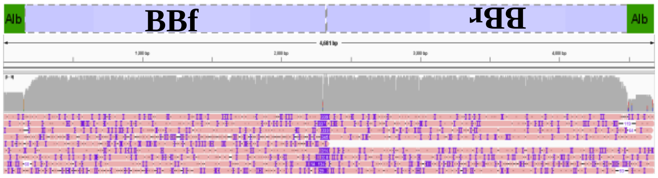

BBrBBf inserts = 0.04%. Shows 8 out of 8 reads.

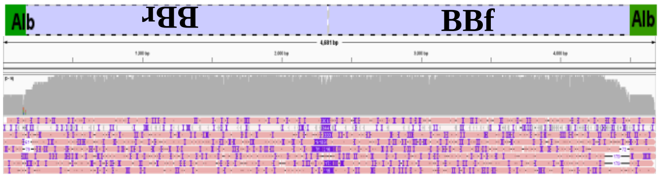

F8rF8r inserts = 0.08%. Shows 10 out of 19 reads.

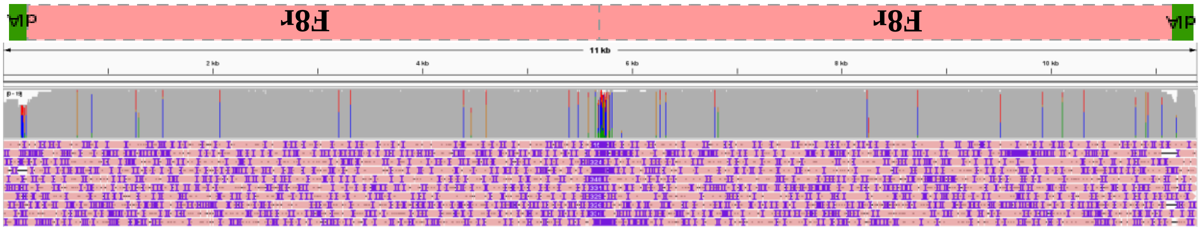

F8fF8f inserts = 0.08%. Shows 10 out of 17 reads.

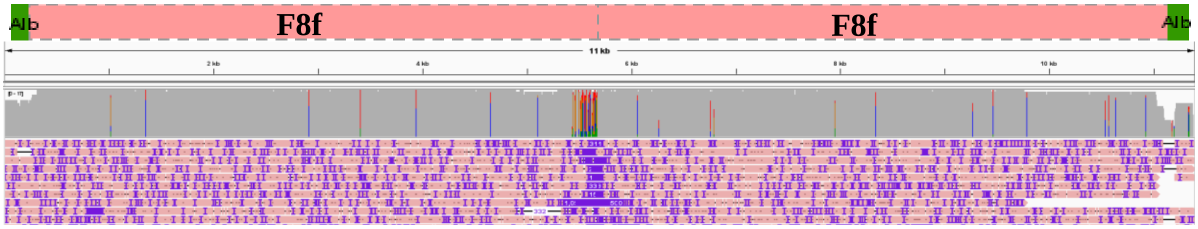

F8fF8r inserts = 0.05%. Shows 10 out of 11 reads.

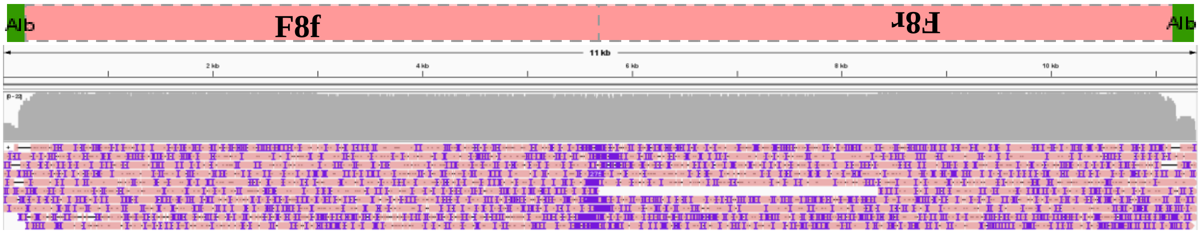

F8rF8f inserts = 0.03%. Shows 8 out of 8 reads.

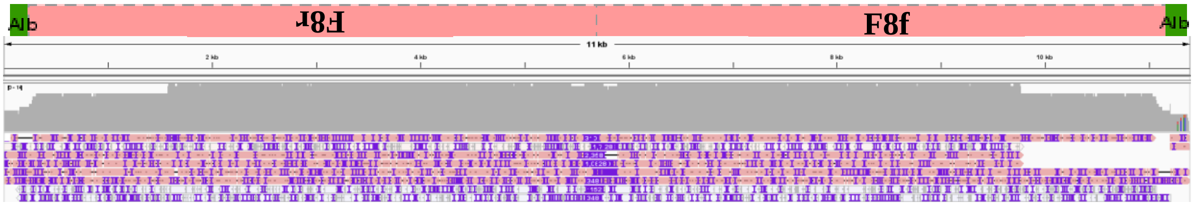

Figure S6B. Visualization of additional two-fragment integrations not shown in Figure 5, including four patterns of F8 and BB insertions in both orientations (continued).

C Three-fragment insertions: BB+BB+F8

F8fBBrBBr inserts = 0.02%. Shows 5 out of 5 reads.

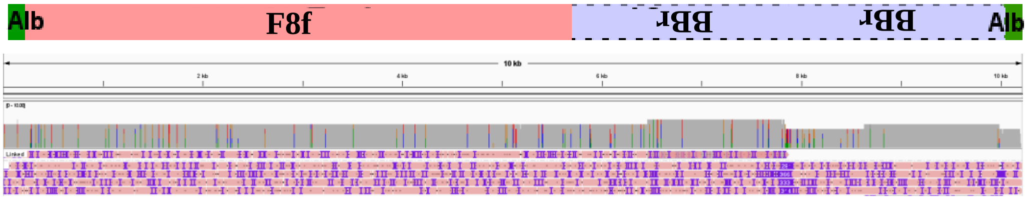

F8rBBfBBr inserts = 0.01%. Shows 3 out of 3 reads.

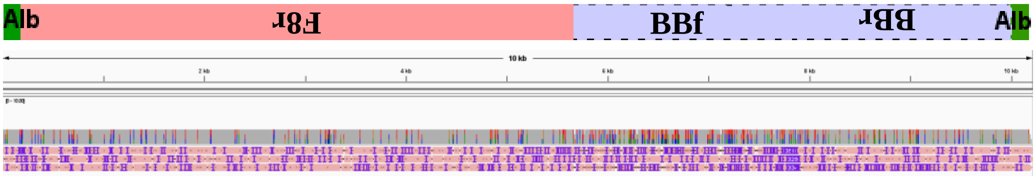

F8fBBfBBf = 0.004%. Shows 1 out of 1 read.

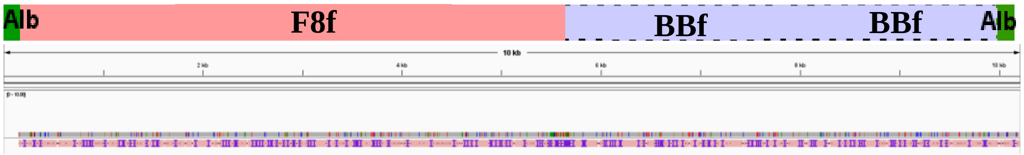

F8fBBfBBr = 0.004%. Shows 1 out of 1 read.

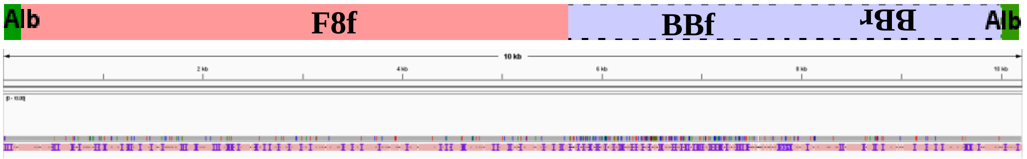

F8fBBrBBf = 0.004%. Shows 1 out of 1 read.

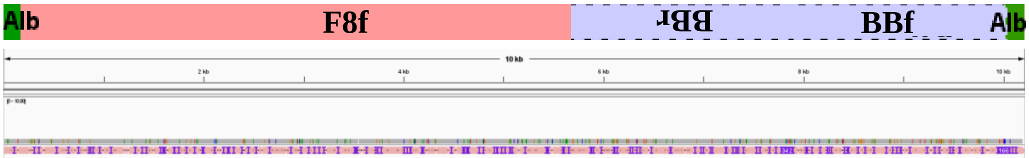

F8rBBfBBf = 0.004%. Shows 1 out of 1 read.

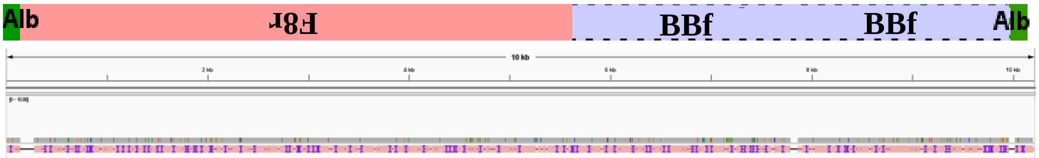

BBfF8rBBr inserts = 0.01%. Shows 3 out of 3 reads.

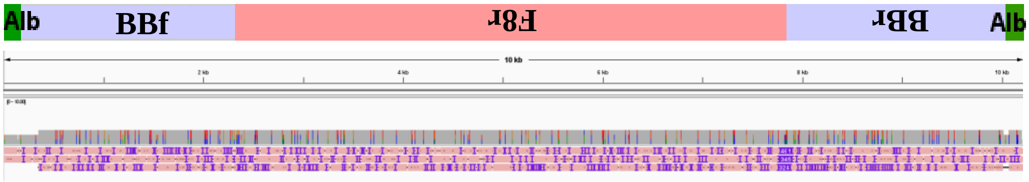

BBrF8fBBf = 0.004%. Shows 1 out of 1 read.

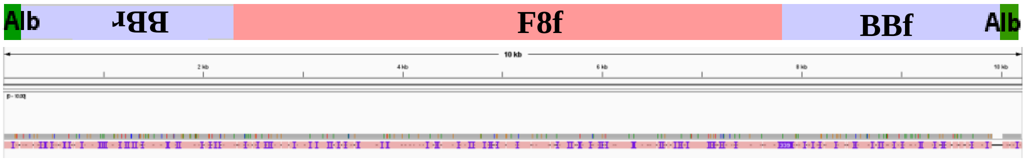

Figure S6C. Visualization of three-fragment insertions, specifically the insertion of two plasmid backbone fragments and one F8 fragment.

BBrF8rBBf = 0.004%. Shows 1 out of 1 reads.

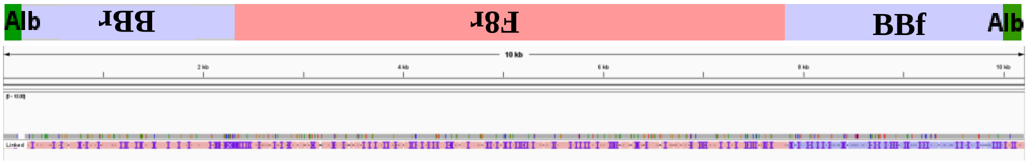

BBfBBfF8r inserts = 0.03%. Shows 6 out of 6 reads.

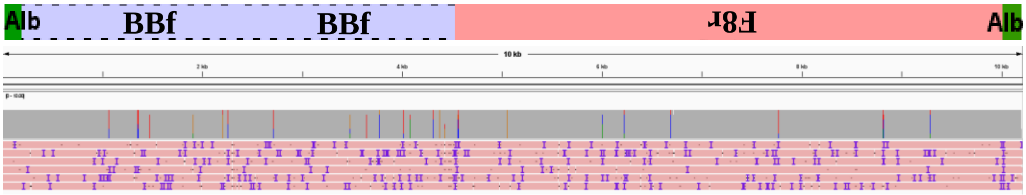

BBrBBrF8f inserts = 0.02%. Shows 4 out of 4 reads.

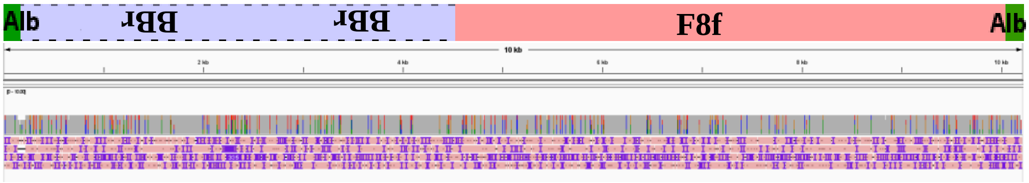

BBrBBfF8r inserts = 0.01%. Shows 3 out of 3 reads.

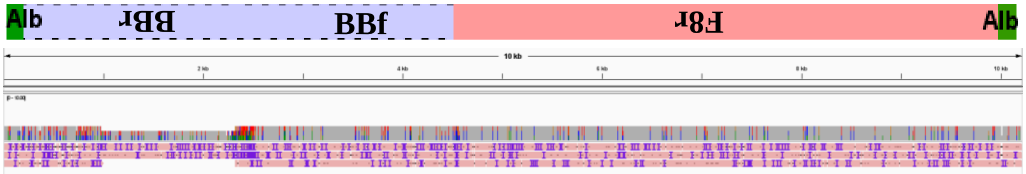

BBfBBrF8r inserts = 0.004%. Shows 1 out of 1 reads.

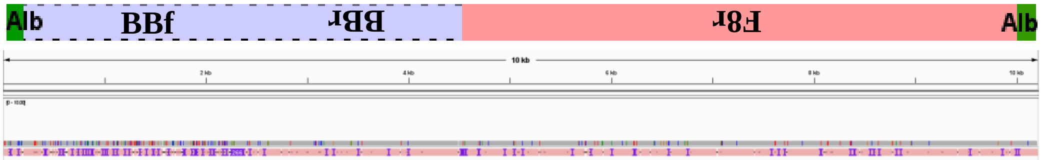

BBfBBrF8f inserts = 0.004%. Shows 1 out of 1 reads.

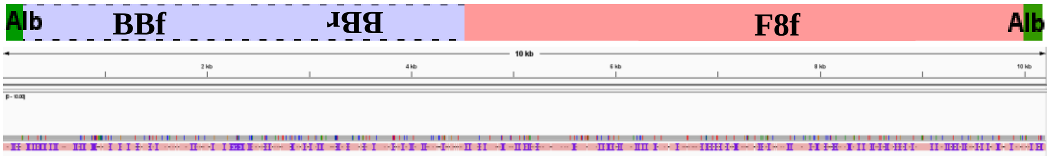

Figure S6C. Visualization of three-fragment insertions, specifically the insertion of two plasmid backbone fragments and one F8 fragment (continued).

D Three-fragment insertions: BB+F8+F8

BBrF8fF8f inserts = 0.01%. Shows 3 out of 3 reads.

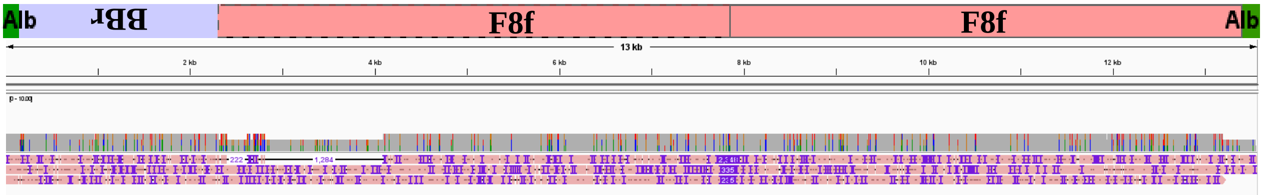

BBfF8rF8f inserts = 0.01%. Shows 2 out of 2 reads.

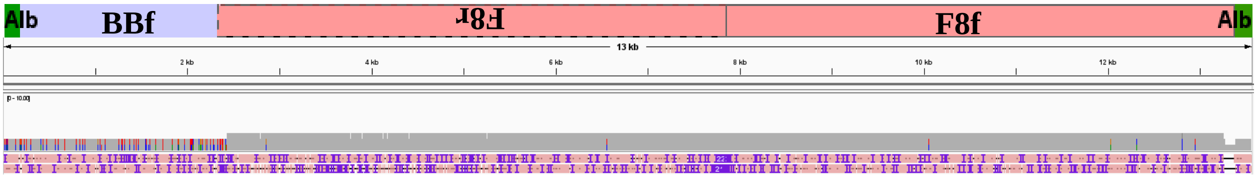

BBrF8rF8f inserts = 0.01%. Shows 2 out of 2 reads.

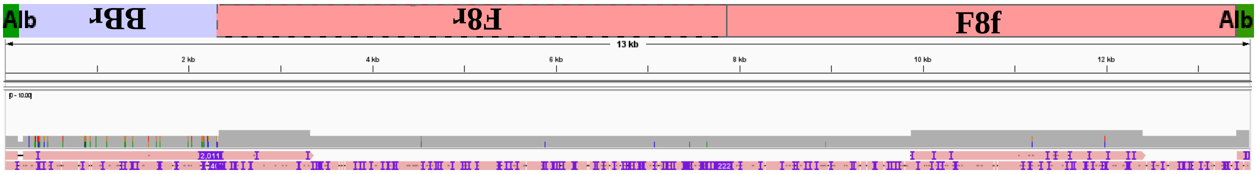

BBfF8fF8f inserts = 0.004%. Shows 1 out of 1 reads.

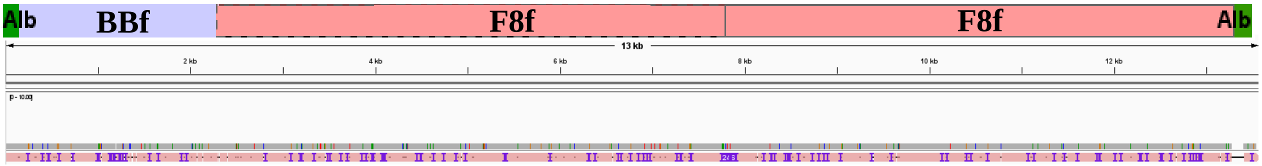

BBfF8rF8r inserts = 0.004%. Shows 1 out of 1 read.

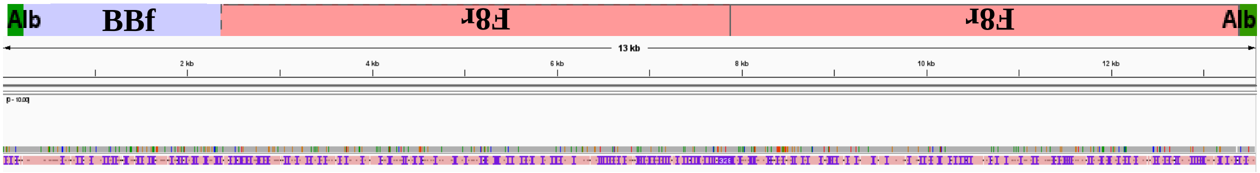

BBrF8fF8r inserts = 0.004%. Shows 1 out of 1 read.

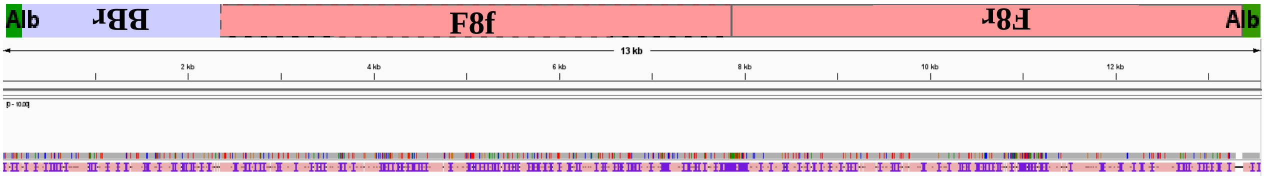

F8fBBrF8f inserts = 0.03%. Shows 7 out of 7 reads.

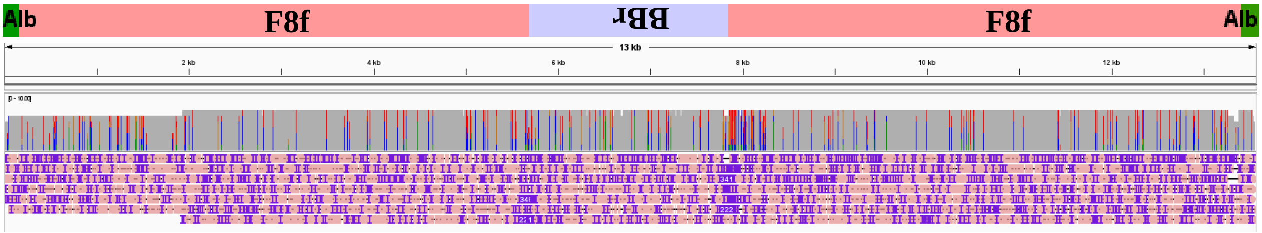

F8fBBrF8r inserts = 0.03%. Shows 6 out of 6 reads.

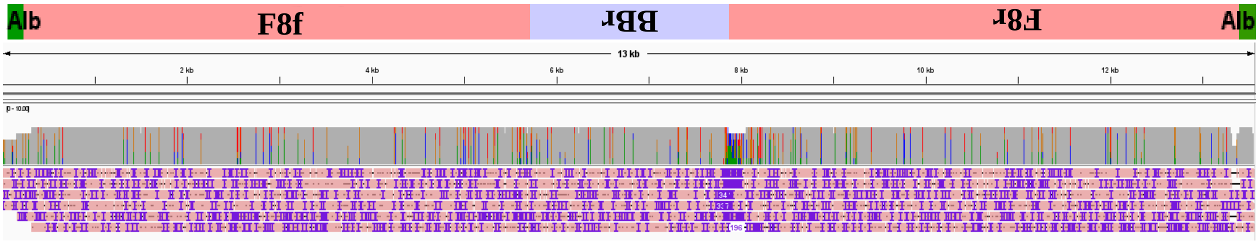

Figure S6D. Visualization of three-fragment insertions, specifically the insertion of one plasmid backbone fragment and two F8 fragments.

F8fBBfF8r inserts = 0.02%. Shows 4 out of 4 reads.

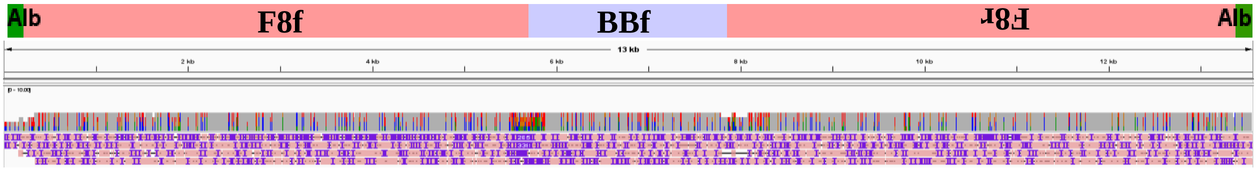

F8rBBfF8r inserts = 0.02%. Shows 4 out of 4 reads.

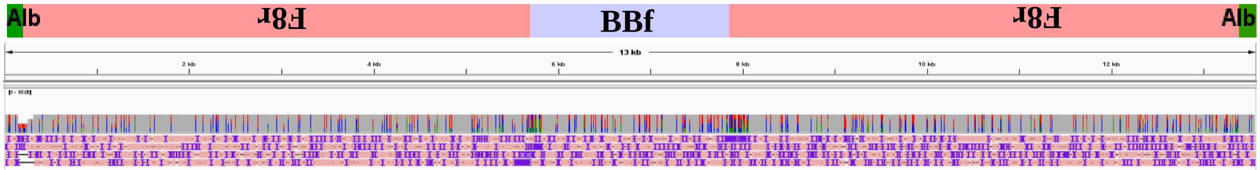

F8rBBfF8f inserts = 0.01%. Shows 3 out of 3 reads.

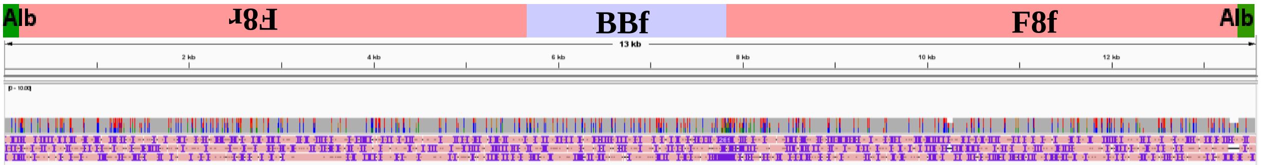

F8fBBfF8f inserts = 0.004%. Shows 1 out of 1 read.

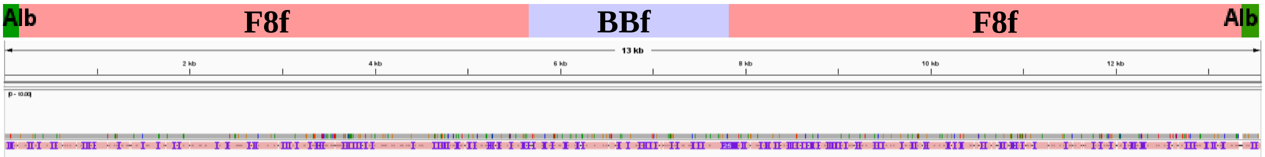

F8rBBrF8f inserts = 0.004%. Shows 1 out of 1 read.

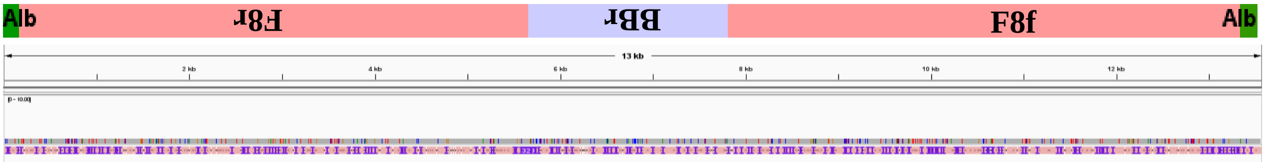

F8fF8fBBr inserts = 0.03%. Shows 7 out of 7 reads.

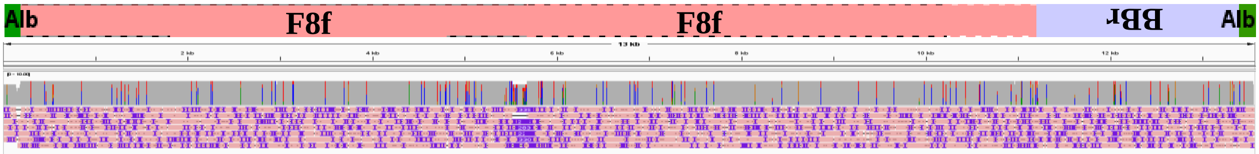

F8rF8rBBf inserts = 0.01%. Shows 3 out of 3 reads.

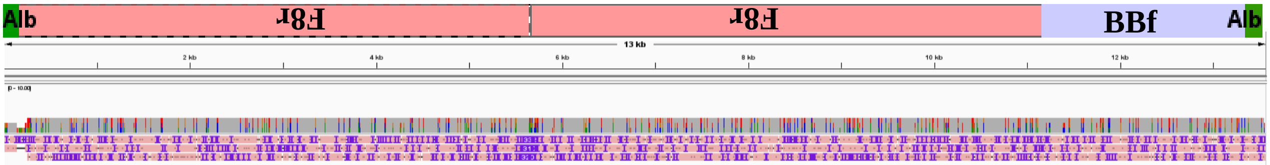

F8fF8rBBf inserts = 0.01%. Shows 2 out of 2 reads.

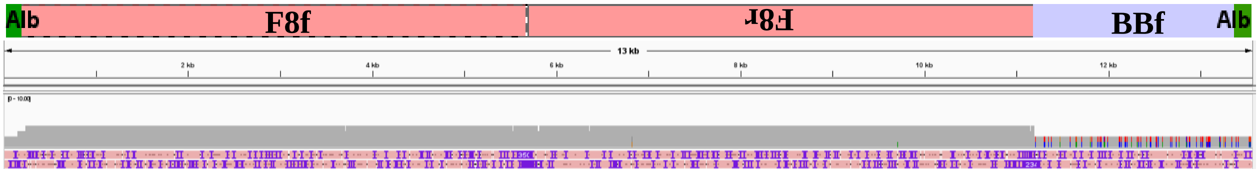

F8rF8fBBr inserts = 0.004%. Shows 1 out of 1 read.

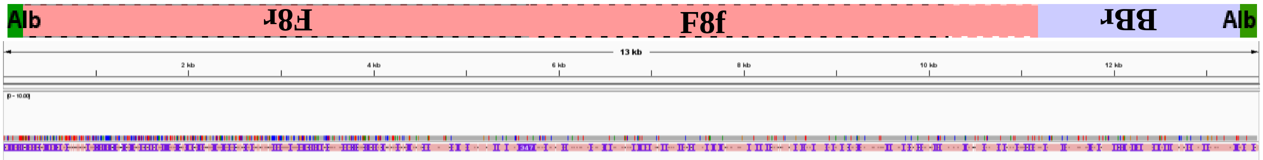

Figure S6D. Visualization of three-fragment insertions, specifically the insertion of one plasmid backbone fragment and two F8 fragments (continued).

E Three-fragment insertions: BB+BB+BB

BBrBBfBBr inserts = 0.01%. Shows 2 out of 2 reads.

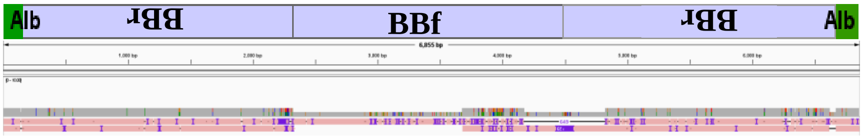

BBrBBfBBf inserts = 0. 004%. Shows 1 out of 1 read.

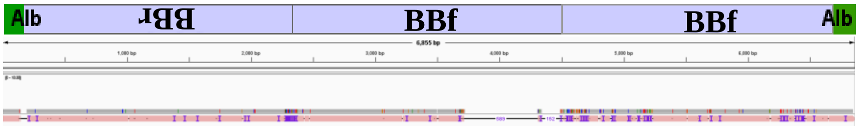

BBrBBrBBr inserts = 0. 004%. Shows 1 out of 1 read.

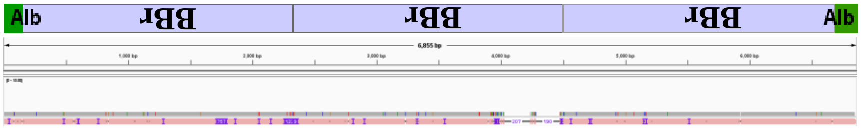

BfBBfBBf inserts = 0. 004%. Shows 1 out of 1 read.

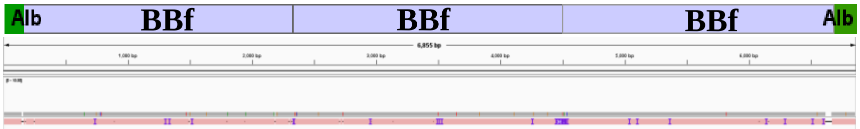

BBfBBfBBr inserts = 0. 004%. Shows 1 out of 1 reads.

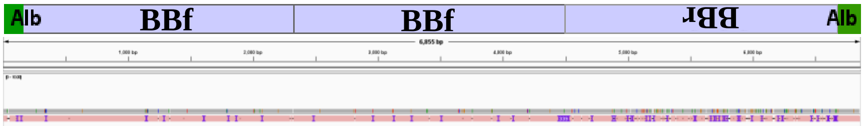

F Three-fragment insertions: F8+F8+F8

F8rF8rF8r inserts = 0. 004%. Shows 1 out of 1 read.

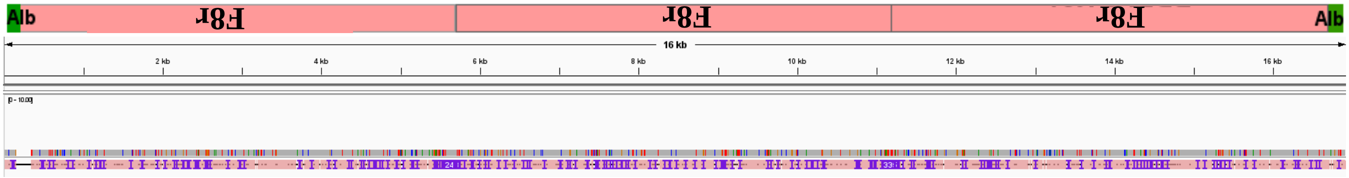

Figure S6EF. The visualization of three-fragment insertions, specifically the insertion of three plasmid backbone fragments (E), and three F8 fragments (F).

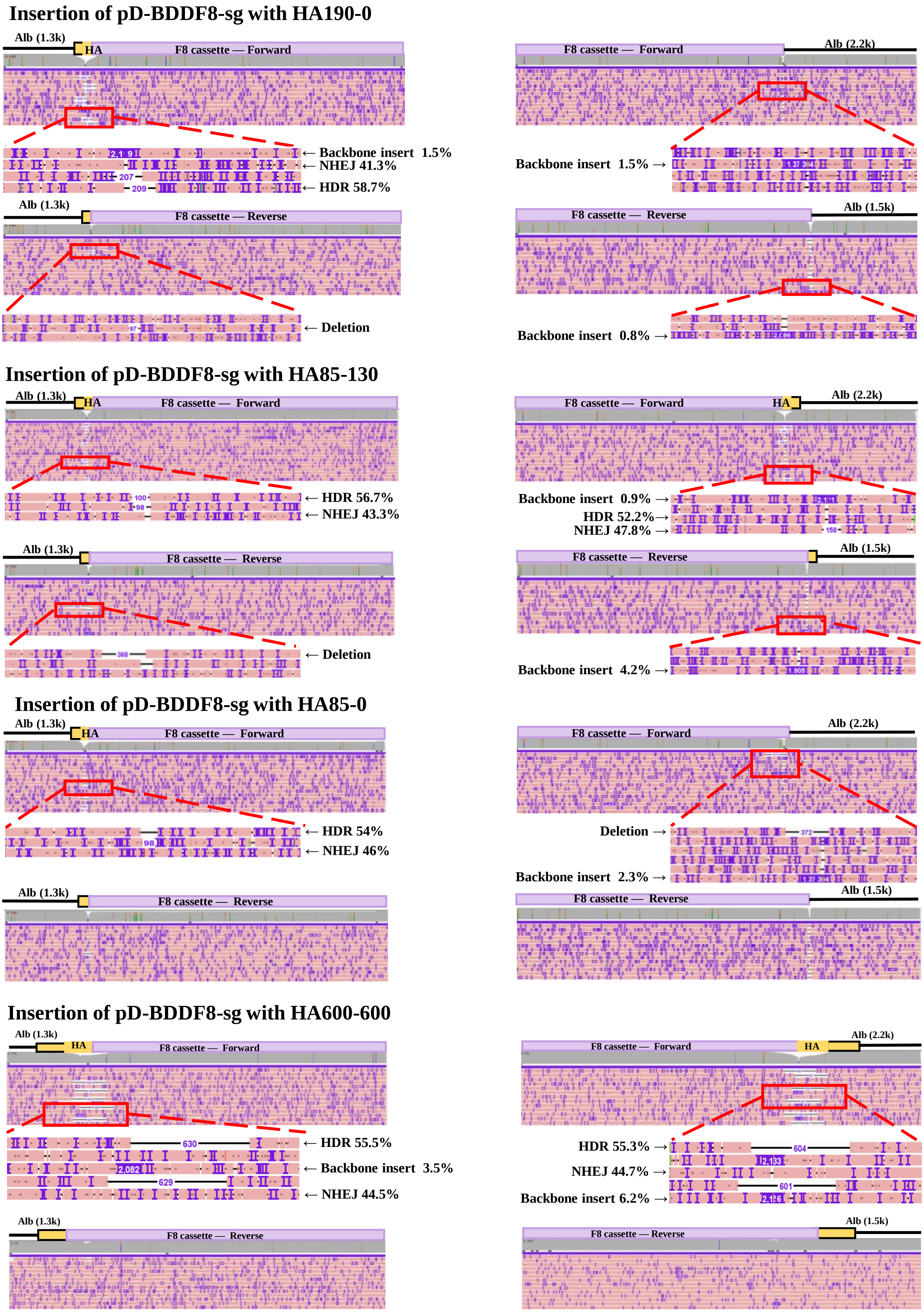

Figure S7. Visualization of PCR Products from Mouse Liver Edited with Donor Plasmids Featuring Various Homology Arm Lengths. Integrated Genomics Viewer (IGV) was employed to display the PCR products derived from mouse liver that had been edited using donor plasmids with different lengths of homology arms, specifically HA190-0, HA85-130, HA85-0, and HA600-600. The figure presents alignments of 15 randomly selected reads for each homology arm length.

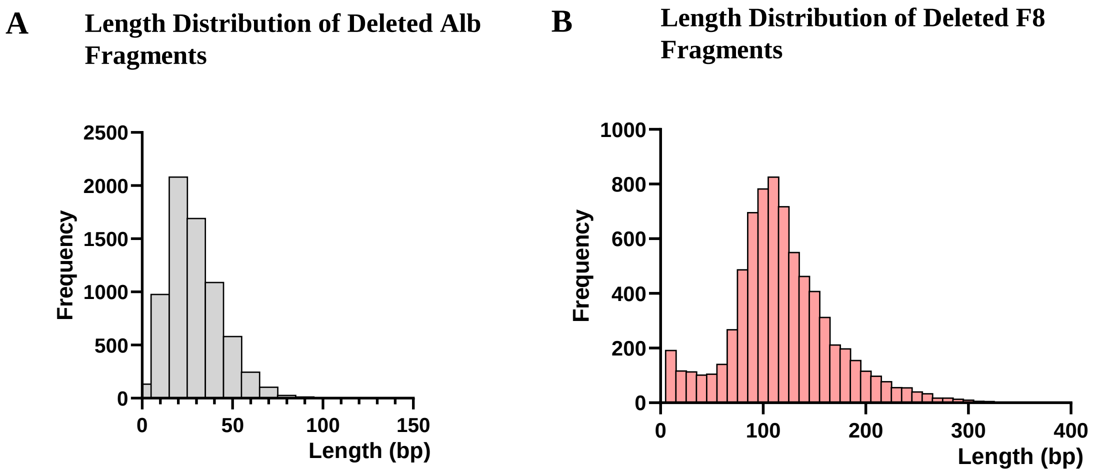

Figure S8. Analysis of Deletion Length Distribution in Junction PCR of pD-BDDF8-sg Inserts with Homology Arm HA85-0. (A) Length Distribution of Deleted Alb Fragments: This section presents the distribution of deletion lengths observed in the Alb fragments within the junction PCR of F8 donor inserts that utilized the HA85-0 homology arm. (B) Length Distribution of Deleted F8 Fragments: Here, the focus is on the distribution of deletion lengths found in the F8 fragments within the same junction PCR of F8 donor inserts, again using the HA85-0 homology arm.

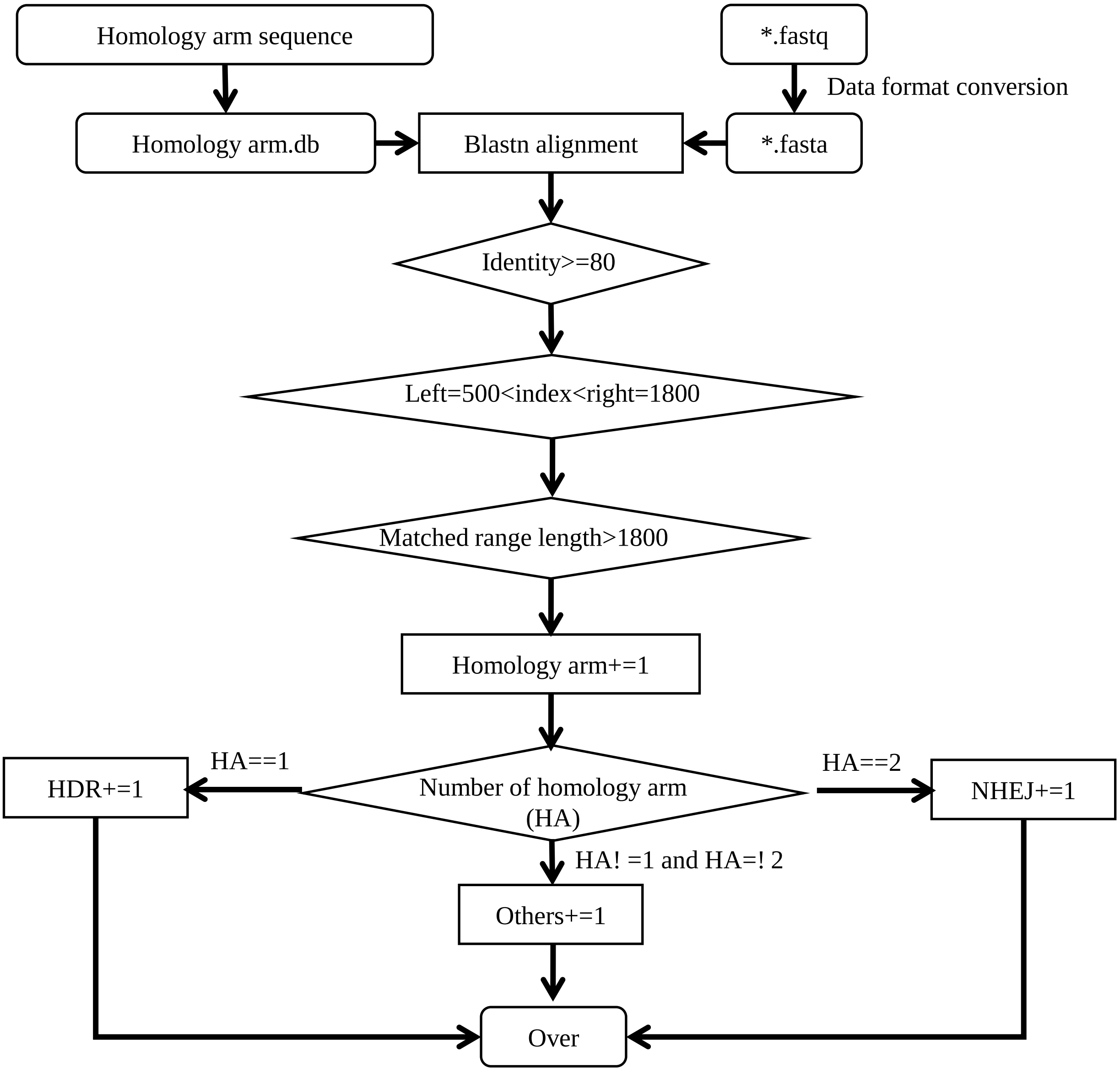

Figure S9. Workflow for Distinguishing Non-Homologous End Joining (NHEJ) and Homology-Directed Repair (HDR) in Donor Insert Analysis. This flow chart outlines the data processing steps used to differentiate between NHEJ and HDR events, with a focus on a donor with 600bp homology arms as a case example. The analysis employs the Basic Local Alignment Search Tool (BLAST) to search for corresponding arm sequences and determine the proportion of inserts mediated by each repair pathway. The steps include:

1. Aligning the homology arm sequences with the sequencing file and selecting reads with an identity score of 80 or more.
2. Restricting the range of alignment to ensure the homology arms are located near the target site.
3. Limiting the length of screened reads to exclude artifacts caused by incomplete Nanopore sequencing.
4. Classifying reads that match up to two copies of homology arms as 'NHEJ' and those matching only one homologous arm as 'HDR'. Reads not meeting either condition are categorized as 'Others’.

The reference sequence used in this analysis was generated by directly inserting the donor with homology arms into the cleaved genomic sequence.

A. Schematic definition of terms used in the data processing

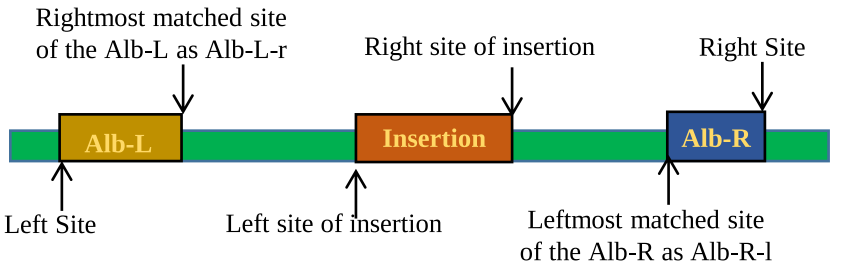

B. The flow chart for processing data to analyze endogenous genomic DNA insertion

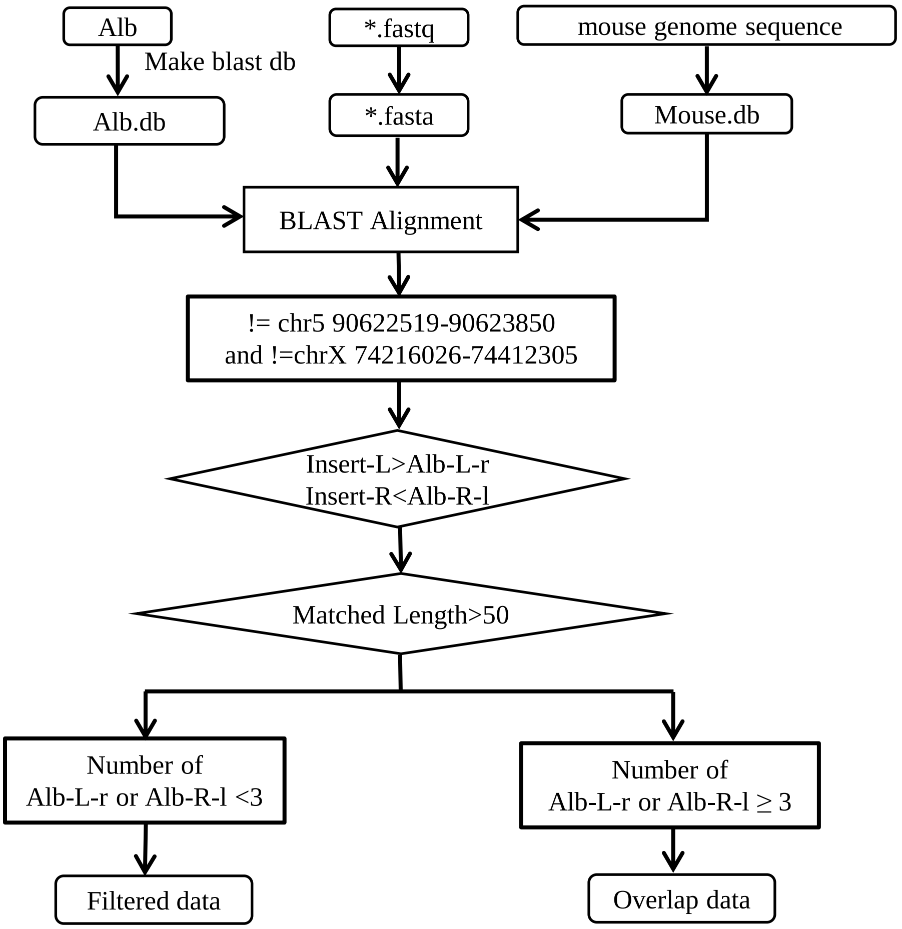

Figure S10. Data Processing Workflow for Analyzing Endogenous DNA Insertion. This flow chart describes the steps for processing data to analyze the insertion of endogenous DNA. It includes the following key components:

A. Definition of Terms:

Alb-L: The upstream homology arm near the cut site.

Alb-R: The downstream homology arm.

Alb-L-r: The rightmost matched site of Alb-L.

Alb-R-l: The leftmost matched site of Alb-R.

B. Steps for Data Processing:

Generation of database files containing the 332bp homology arm sequence (denoted as Alb), the mouse genome sequence, and the LINE-1 sequence.

Conversion of sequencing data format from *.fastq to *.fasta.

Alignment of the sequencing file with the homologous arm sequences and the mouse genome sequence (or the LINE-1 sequence).

Elimination of sequences matching the Alb gene range on chromosome 5 and the F8 gene range on chromosome X.

Limitation of insertions between Alb-L-r and Alb-R-l to ensure endogenous DNA fragments integrate at the target site.

Removal of indexes matched with a length of less than 50bp.

Consideration of occurrences where the number of Alb-L-r or Alb-R-l is greater than three as derived from the same amplicon, counting them as the same type of insertion.

Read #1 Alb-chr1-Alb

CCGTCACAAGCAATGCAAGGCACGTACGTTTACTTATGTCATTGCCTATGGCTAGAAGTGCAAATCCAACAGTCCTGCTAATACTTTTCTAACATCCATCATTTCTTTGTTTTCAGGGTCCAAACCTTGTCACTAGATGCAAAGACGCCTTT
AAAAGACTCTAGGTGGGGATTCAGCCCGGCTGTTGGACTCTCTAAAATACCAGGACCCTGACTGATGCCCAGCACTCATCACTGGTCAGATGGTTCATTGTGGTGTGGCTGGGACAATATGACAGGAAGGTGATATGCTCCTAAACACATCA
CAACCACAACCTTCTCAGGTAACTATACTTGGGACTTAAAAACATAATCATAATCATTTTTCCTAAAACGATCAAGACTGATAACCATTTGACAAGAGCCATACAGACAAGCACTAGCTGGCACTCTTAGGTCTTCACGTATGGTCATCAGT
TTGGGTTCCATTTGTAGATAAGAAAC

Read #2 Alb-chr1-Alb

GTGACCAGAGCAATGCAAGGCACGTACGTTTACTATGTCATTGCCTATGGCTATGAAGTGCAAATCCTAACAGTCCTGCTAATACTTTTTCTAACATCCATCATTTCTTTGTTTCAAGTTCAAACCTCAGCACTTGGGAGGCAGAAGGCAGA
TGGGTCTCTCAGTTCCAGGCCAGCCTGGTCTACAGAGTGAATTTCAGAACAACCAGGCCTACCCAAAACAGTGTCTCACAAAACCAAAAAAAAAAAAAAGAAAAAAGAAAAGAAAGAGAAGAAAAAGAAAGAGAGAGAGAGAGAGGGAGGGTG
GCAGGCTGCTAAAACAGCTAGATTTTATAGCAGGCAAAAAAAACAGTCCTTCAAAATTTTTTTTTAACCTCTGGAAACTTTCTTAAATTATAAGTAATTAAAATTACATGACACTCATGAGTCCCTGGCCTGGCCTGGGGGAAATGTTGAAA
AGACCTAAACACATCACAACCACAACCTTCTCAGGTAACTATACTTGGGACTTAAAAAACATAATCATAATCATTTTTTCCTAAAACGATCAAGACTGATAACCATTTGACAAGAGCCATACAGACAAGCACCAGCTGGCACTCTTAGGTCT
TCACGTATGGTCATCAGTTTGGGTTCCATTTAGCCAGATAAGAAAC

Read #3 Alb-chr2-Alb

GCACCTAGACACGGTAGCAATGCAAGGCACGTACGTTTACTAGTGTCATTGCCTATGGCTATGAAGTGCAAATCCCAACAGTCCTGCTAATACTTTCTAACATCCATCATTTCTTTTTTTTTTTTTTTTTTTTTTTTTGGTTTTTCGAGACA
GGGTTTCTCTGTATATCCCTGGCTGTCCTGGAACTCACTCTGCAGACCAGGTTGGCCTCGAACTCAGAAGTCCGCCTGCCTCTCTGCCTCCCGAGTGCTGGGATTAAAGGCGTGCGCCACCACGCCCGGCTTAGATCCTTATTTCATGGAGA
GTCACTGGTACTTAAAGGATCAGAATAAACTGTTTGCACAGAATTGTTGCTTTTGGAAGAAAGCACTTTTTAAGGGTCCTTTATCAACTTAATGTATCATTGCTAATTAAAAGTCCTTCCTCCCAAGTGTGATGACTGGAAGTTGAATTCTT
AGAAACTATGAATGCCAGCTGTGTAATCTCAGCACTGGAAAGGAGCCTAAACACATCACAACCACAACCTTCTCAGGTAACTATACTTGGGACTTTAAAAACATAAATCATAATCATTTTTCCTAAAACGATCAAGACTGATAACCATTTGA
CAAGAGCCATACAGACAAGCACCAGCTGGCACTCTTAGGTCTTCACGTAGCGGTCATCAGTTTGAAGTTCCATTTTGTAGATAAGAAACCGAGGTGC

Read #4 Alb-chr2-Alb

AATGCAAGGCACGTACGTTTACTATGTCACGTTTTATGGCCATGAAGTGCAAATCCTAACAGTCCTGCTAATACTTTTCTAACATCCATCATTTCTTGTTTCAGGGTCCAAACCTTGTCAATGATGCAAAGACGCCTTCAGTTTTCTCGCCA
TATTTCGCATCCATTATATTTCTCATTTTCCGTGATTTTCAGTTTTCTCGCCATAATCCAGGTCCTTCAGTGTGCATTTCTCATTTTCTTTACGTTTTTTTAGTGATTTCCTGTCATTTTCAAGTCTTCAAGTGGATGTTTCTCATTTTCCA
TTATTTTCAGTTTTCTTGCACATCACAACCACAACTCTCTCAGGTAACTATATTTGGGACTTAAAAAACATAATCATAATCATTTTTCCTAAAACGATCAAGACTGATAACCATTTGACAAGAGCTATACAGATGCCACCAGCTGGCACTCT
TAGGTCTTCACGTATGGTCATCAGTTTGGGTTCCATTTGTAGAT

Read #5 Alb-chr2-Alb

CGACCAGCCAGCAATGCAAGGCACGTACGTTTACTATGTCATTGCCTATGGCTATGAAGTGCAAATCCTAACAGTCCTGCTAGCAATACTTTTCTAACATCCATCATTTCTTTGTTTTCAGGGTCCAAACCTTGTCACTAATATGCAAAGAC
GCCTACGTCCTAAAGTGTGAATTTCTCATTTTTCGTGATTTTCAGTTTTCTCGCCATATTCCAGGTCCTTCAGTGTGCATTTCTCATTTTTCACGTATTTTAGTGATTTGGGATTTTTCAAGTTGTCAAGTGGATGTTTCTCATTTTCCATG
ATTTTCAGTTTTCTTGCCATATTCCACGTCCTACAGTGGGCATTTAAATTTTCCACCTTTTCCTAACACATCACAACCACAACCTTCTCAGGTAACTATACTTGGGACTAAAAACATAATCATAATCATTTTTCCTAAACCATCAAGACTCA
TAACCATTTGAGTAGAGCCATACAGACAAGCACCAGCTGGCACTCTTAGGCTTCACGTATGGTCATCAGTTTGGGTTCCATTTGTAGATAAAGAATCAT

Read #6 Alb-chr2-Alb

GAGCAATGCAAGGCACGTACGTTTACTATGTCATTGCCTATGGCTATGAAGTGCAAATCCTAACAGTCCTGCTAATACTTTTCTAACATCCATCATTTCTTTGTTTCAGGGTCCAAACCTTGTCACTAGATGCAAAACTACTTATCCTCTTC
CAACAATATGTTGTTTCAGTGTAACTCACTCATCTAATCTGTTCTACAGTGTGGTTTTTATCATTTTCCATGTTTCTCATTGTAACTCATTGATATACACTGTTCTACAGAATCCATTTTCCAAAGAATGTGTTTTTCAGTGTAACTCACTC
GCCTAAACACATCACAACCACAACCTTCTCAGGTAACTATACTTGGGACTTAAAAAACATAATCATAATCATTTTTCCTAAAACGATCAAGACTGATAACCATTTGACAAGAGCCATACAGACAAGCACCAGCTGGCACTCTTAGGTCTTCA
CGTATGGTCATCAGTTTGGATTTCATTTGTC

Read #7 Alb-chr2-Alb

GGAGCAATGCAAGGCACGTACGTTTACTATGTCATTGCCTATGGCTATGAAGTGCAAATCCTAACAGTCCTGCTAATACTTTTCTAACATCCATCATTTCTTTGTTTTCAGGGTCCAAACCTTGTCACTAGATGCAAAGACGCCTTGACAGG
CCTATGAGCAAGACTTTGGTCTCCTGTAATGCAGAGCCATCCATAGTGCAGAGCAACAGGCCTATGAGGCATTAAGACAGACTATACTGAATACTGCATCTTACATGCCCAAGTCAGGTTCACAGAGCTCGAGATGAGATTAAGAGAACTCA
TGGAGGAACTCCAGACAACAAGATGCTAGAAGGTAAGACAGTCCAGGGAGTTCAAAGAGTGCAGGAAGCCGTACCCAATTTAAGGGAAAGAGAATCATTGTTGTTAGGTGCCATCTCAGTTGAAACACATCACAACCACAACCTTCTCAGGT
AACTATACTTGGGACTTAAAAAACATAATCATAATCATTTTTCCTCAAAACGATAGTTTCAAGACTGATAACCATTTGACAAGAGCCATACAGACAAGCACAGCTGGCACTCTTAGGTCTTCACGTATGGTCATCAGTTTTCAGTACCATTT
GTAGAT

Read #8 Alb-chr2-Alb

AGCACCTACGCCACGTTAGCAATGCAAGGCACGTACGTTTACTATGTCATTGCCTATGGCTATGAAATTACCAAATCCTAACAGTCCTGCTAATACTTTTCTAACATCCATCATTTCTTTGTTTTCAGGGTCCAAACCTTGTCACTAGATGC
AAAGACGCCTTAGTTTTTCCTCGCCATATTTCACGTCCTAAAGTGTGTATTTCCTCCATTTTCTGTGATTTTCAGCGGATTTTTTCTCAACCATATTCCAGTCCTTCAGTGTGCATTTCTCATTTTTCACGTTTTTAGTGATTTCGTCATTT
TCCTAAACACATCTTCCACAACCACAACCTTCTCAGGTAACTATACTTGGGACTTAAAAAACATAATCATAATCATTTTTCCTAAAACGATCAAGACTGATAACCATTTGACAAAGAGCCATACAGACAAGCACCAGCTGGCACTCTTAGGT
CTTCACGTATGGTCATCAGTTTTGGAGTTTCATTTGT

Read #9 Alb-chr2-Alb

GCACTCCTCGACTAGACCAGTAATGCAAGGCACGTACGTTTACTTGCGTCATTGCTCTGCGGCTATGAAGTGTGTAATCCTAACAGTCCTGCTAATACTTTTCTAACATCCATCATTTCTTTGTTTTCAGGTCCAAACCTGTCACTAGATGC
AAAGACGCCTTAAGGTGGAAAATTTAGAAATGTTCACTGTAGGACGGTGAAATATGGCAAGAAAACTGAAAATCATGGAAAATGAGAACCATCCACTTGACGACTTGAAAATGACGAAATCACTAAAAACGTGAAACGAGAAATGCAACACC
GAAGGACCTGGAATATGGCGCCTAAACACATCACAACCACAACCCTCTCAGGTAACTATACTTGGGACTTAAAACATAATCATAATCATTTTTCCTAAAACGATCAAGACTGATAAACCATTTGACAAGGCTTCATAAGATGGCACCAGCTG
GGAAAAAAATTTTAGGGTCTCACGTATGGTCACCAGTTTGGGTTCCATTTGTAGATAAGAAAC

Read #10 Alb-chr2-Alb

AGGACACGGTAGCAAATGCAAGGTACTTCACGTTTACTATGTCATTGCCTATGGCTAAGTGCAAATCCTAACAGTCCTGCTATAATACTTTTCTAACATCCACTGCTTCCTTTGTTTTCAGGGTCCAAACCTTGTCACTAGATGCAAAGACG
CCTCTCTGGCTCTGAGGCCATCTAGTCTACAGAGTGAGTTCCCAAACAACCAGGGCTACACAGAGAAACCTGTCTCAAATGAACAAAAACAACAGAGTAACAAAACAAAAGGTTAGAAAGAAAGCTAATGCCTAAACACATCACAACCACAA
CCTTCTCAGGTAACTATACTCTGGGACTTAAAAACATAATCATAATCATTTTTCCTAAAACGATCAAGACTGATAACCATTTGACAAGAGCCATACAGACAAGCACCAGCTGGCACTCTTAGGGCTTCTCCTCATGTATGGTCATCAGTTTG
GGTTCCATTTGTAGATAAAGAAAC

Read #11 Alb-chr2-Alb

GAGCAATGCAAGGCACGTACGTTTACTATGTCATTGCCTATGGCTATGAAGTGCAAATCCTAACAGTCCTGCTAATACTTTTCTAACATCCATCATTTCTTTGTTTTCCAGGGTCCAAACCTTGTGCCTACGAAATCACTAAAAAAACGTGAG
AAAAATGAAATGCACACTGAAGGACCTGGAATATGGCGAGAAAACGGAAAATCACGGAAAATGAAAATACACACTTTAGGACGTGAAATATAGCGAAGATGCCCAGAAAAAGGTGGAAAATTTAGAAATGTCCACTGTAGGACGTGGAATAT
GGCAGAAAACTGAAAATCACGGAAAATGAGAAATACACACTTTAGGACGTGAAATATGGCAGGAAAACAGAAAAGGTGGAAAATTTAGAAATGTCCACTGTAGGACTTTGAATAGGGAGCAAAAAAGTGAAAATCATAGAAAATGAAACATT
CACTTGACGACTTGAAAAATGAAATCACTAAAATACGTGAAAAATGAAATGCACACACTGAAGGACCTGGAATATGGCGAAAACTGAAAATCATGGAAAATAGAGGAAATACACACTTTAGGAACCTGACATCACAACCACAACCTTCTCGG
AAGTAACTACTTGGGACTTAAAACATAATCACTAATCGTTTCCTAAAACGATCAAGACTGATAACCATTGACAAGAGCCATACAGACAAGCACCAGCTGGCACTCTTAGGTCTTCACGGCCTTGGTCATCAGTTGGGTTCCATTTGTATT

Read #12 Alb-chr3-Alb

AATGCGAATAGCAATGCAAGGCACGCATGTTTACTATGTCATTGCCTATGGCTATGAAGTGCAAATCCTAACAGTCCTGCTAATACTTTCTAACATCCATCATTTCTTTGTTTTCAGGTCCAAACCTTTGTCACTAGATGCAAAGACGCCCA
GAGATACTCAGTGAAAATCTATAGAGTTCACTTCAGAGAATATGTAGTTTGGTAAGTGAGCCTTAAAAATAATAAAATAATACCTACTGCTTTAAAAGCTATCATCTTGCACCTAAACAATACGTAACCACAACCTCTCAGGTAACTATACT
TGGGACTTAAAAAACATAATCATAATCATTTTTCCTAAAACGATCAAGACTGATAACCATTTGACAAGAGCCATACAGATGCTAAATTCAGCTGGCAATTTTAGGTTCCTCACGTATGGTCATCAGTTTGGGTTCCATTTGTAGAT

Read #13 Alb-chr3-Alb

GCATATCCAGCAATGCAAGGCACATACATTTTACTATGCCATTGCCTATGGCTATGAAGTGCAAATCCTAACAGTCCTGCTAATACTTTCTAACATCCATCATTTCTTTGTTTTCAGGGTCAAAACCTCTGTCACTAGATGCAAAGACGCC
TGCTGTGAGAGATGCTGCATTTGGCTTCCTGGTTAGAAGACATGAGCTGAAAGCTTTCCAGTCCACACGCTGACACAAAGACTGCGCCTGTTTACAATAGCTTTACAAGCACAGACTTCCAGCCAGCTGTACTTGCTCTTCCTTCTTTTGGC
TACCACAAGCTTTTGCTTGAACTCCTGCTCAGTGTTGAACCTGCACCGGATGCTGGCAACAAAAGAATGTGCATAAGCCCAAGGCTCTGGCCCTCAGGAACACGAAACCAAGCAGAGAGCGCCACCAAGTTTTTACGCGAAGGATACAGTTC
TGCAAACATGCCTCAAACCTCTGTATCCCACTGGGAATGATTAAATGATACGGTGTGTTTGATGTGATGTTCTCCATCCACATATTTGCACATATCACATATATCATCCACCTGGCAGATTTCACAAAAGAGCAACAGCCATGTGGGATGGA
TATTTTGGTTTCCCAACTTGACTACACCTGGAATTCACCAAAACCCAAGCATCTGGATACACCTGTGAGATTATTAAACACATCACAACCACAACCTTCTTAGGTAACTATACTTGGACTTAAAAAACATAATCATAATCATTTCCTAAAAC
GATCAAGACTGATAACCATTTGACAAGAGCCATACAACAAGCACCAGCTGGCACTCTTAGGTCTTCACGTATGGTCATGAGTTGGGTTCCATTTGTAGATAGAAAC

Figure S11. Representative Sequences Illustrating Various Insertion Patterns at the Alb Site. (A) Single Genomic DNA Fragment Insertion: The displayed read sequences show the insertion of a single piece of genomic DNA fragment at the Alb site.

Read #14 Alb-chr3-Alb

TCAATTCAGAGCAATGCAAGGTAATGTACGTTTACTTGTCATTGCCTATGGCTATGAAGGCAAATCCTAACAGTCCTTGCTAATACTTTCTAACATCCATCATTTCTTTGTTTTCAGGGTCCAAACCCCAGCCCAGCCTTGTTTTTATTTTA
GGGGTGGGTAGGGGAGGGGGGTGGGTGGGTATGGGGACTTTTGGGATAGCATTGAAATGTAAATGGAGCTAAATACCTAATAAAAATGGAAAAAAAAAAATTGCAGAAAATCACACTATAATAGAAATGGTTCCTAAACACATCACAACCA
CAACCTTCTCAGGTAACTATACTTGGGACTTTAAAAAACATAATCATAATCATTTTTCCTAAAACGATCAAGACCGATAACCATTTGACAAGAGCCATACAGACAAGCAATTAGCTGGTACTTAGGTCTTCACGTATGGTCATCAGTTTGCT
CCATTTGTAGAT

Read #15 Alb-chr4-Alb

ACACCACAAGCAATGCAAGGCACTGTACGTTTACTATGTCATTGCCTATGGCTATGAAGTGCAAATCCTAACAGTCCTGCTAATACTTTTTAACATCCATCATTTCTTTGTTTTCAGGGTCCAAACCTTGTCACTAGATGCAGATGCCTTAG
AGACAGAGACAGAGAGAAGGGAAGTAGAGGCCAGTCGTGGACACATGGAGTGGAGAGAGAAGGGAAGACAGAGAAAGGGGAGCAAGGGTAAGCACAAGAAAATAAGAGGTTAGAGAGTGGGGAGGGCCTAAACACATCACAACCACAACCTT
CTCAGGTAACTATACTTGGGACTTAAAAAACATAATCATAATCATTTTTCCTAAAACGATCAAGACTGATAACCATTTGACAGAGCCATACAGACAGTACTAGCTGGCACTCTAGGCTTCACGTATGGTCATCAGTTTGGGTTCCATTTGTA
GATAAAGAAACT

Read #16 Alb-chr4-Alb

AATGCAAGGCACGTACGTTTACTATGTCATTGCCTATGGCTATGAAGTGCAAATCCTAACAGTCCTGCTAATACTCTTCTAACATCCATCATTTCTTTGTTTTCAGGGTCCAAACCTTGTCACTAGATGCAAAGACGCCAGTGTGCATTTCA
TTTTCCACGTTTTTGAGAAACATGCATTTCATTTTCACCTTTTGAGTGTTTCTCGCCATATTCCATGTCAAACCGTGTTTGTGTCTCTATTTTCCATTGTTTTTCTGTTTTCTCACCTTATTCCAGGTCCTAAAATTTCCATTTCATTTTTC
ACTTTTTTTCAAGGTTTCTAGCCACCTAAACACATCACAACCACAACCTTCAGGTAAACTATACTTGGGACTTAAAAAACATAATCATAATCATTTTTCCTAAAACGATCAAGACTGATAAACCATTTGACAAGAGCCATACATGACAAGCA
CCAGCTGGCACTCTTAGGTCTTTTTTTACGTATGGTCATCAGTTTGGGCTCCATTTGTAGAT

Read #17 Alb-chr5-Alb

CAGCAATGCAAGGCACGTATTAACATTATGTCATTGCCTGCGCTATGAAGTGCAATCCTAACAGTCCCAAGTACTTTTCTAACATCCATCATTTCTTTGTTTTCAGGGTCCAAACCTTGTCACTAGATGCACAATCTCTTTCCACAATCCAT
TCTACAAAGGATAATAGATGGAAAATACAACACAAGGAGGGAAACTACATCCTAGAAAAAGCAAGAAAGTAATCTTTCAACAAGCCCAAAAGAAGATAGCCACACAATCACACACCCATCTCTAACATGAAAAGTAACAGGAAGCAACACTC
AGTTTTCCTTAATATCTTCTTATCAGTGGACCCATTTCCTAGTAAAAAGACATAGACTAACAGACTGGATAAGTAAACCGTACCTAGAATTTTAGTTGCATACAGGAAACATACCTTAGTCAGCAAAGACAGATACTACCTCAGAGTAAAGT
CTGGAAAAAGTTATCCAAGCACATGGTACCAAGAAGCAAGCTGAGTTGCCATTCTAATATCAAATAAAATCAACTTTATAACCTTTGGTATCATCAGAACCCATTTCACCATAATAACAGAAAGGCAAGATAGATCTAAAATCACTTCATGA
TGATGACAGAGGACATTAAGAAGGACATAAATAACTCCCTTAGAAATACAGGAAACACAGATACTACCTCAGAGTAAAAGTGCTGGAAAAAAATTCAAGCAAATAGTCCCAAGAAACAAGCTGGAGTTGCTATTCTAATATTGAATAAATCA
ACTTGTAACCTTTGGCATCACTGAACCAGTTCTCCCAGCATAACATGTCCATAACCAAAAGTTATAAAGAAAAGATAAGGAAGGACACTTCATACTCATCAAAGGAAAAAAATATACCAAGATGAACTCTCAATTCTGAACATCTATGCTCC
AAATGCACAGGTACCATCACTCATTAAAGAAACTTTACTAAAGCTCAAAGCACACACTGTACCTCATACAGTAATAGTGGAAGATTTCAACACCCCACTCTCAGCAATGGACAGATCATGGAAATAGAAAATAAACAGACACACAGTGAAAC
TTTCAGAAGTTATGAACCAAATGGATTTAACAGATACTTACAGAACATTTCATCCTAAAACAAAAGAATATACCTTCTTCTCAGCACCTCATGGTACCTTCTCCAAAACTGACCACATAATTGGTCACAAACAGGCCTCAACAGATACAAGA
AGACTGAAATAATCCATGCATCCCATCAGATCACCACAGACTAAATCTTGTCTTCAATAATACAACAAAAACAACAGAAAGCCCACATACACATGGAAGCTGAACAATGCTCTACTTCCAATGATAACTTGGTCAAGGAAGAAAGGAAGAAA
ATTAAAGAATTTTTAGAACTTAATGAAAATGAAGGCATATCATACCCAAACAATGAAAGCAGTGCTAAGAGGAAACTCATAGCCTGAGTGCCTCCTAAATAGAAACTGAAGAGAGCATACACTACCAACTCGACAGCACACCTGGAAGCTCT
AGAGCAAACAGAAGAATACACCCAAAAAAGGAGTAGACGGCAGGAAATAATCAAACTCAGAGCTAAAATTAACTAAGTAAAAACAAAGAAAACTATACAAAGAACTTCAAACAAACTCAGGAGCTGGTTCTTTGAGAAAGTCAACAAGGTAG
ATAAACCTCTAGCCGGACTAATCCAAAGGGTCCAGAGCCAGCATCCAAACAATAAAATCAGAATGAAAAGGGAGACATAACAACGGAAACTTATGAAATTAAAAATCCATCAGATCCTACTAAGAAAAGTCTATACTCAACAAAATTGGAAA
ATGTGTGTGTAATGGACAATTTTCTAGCCAGAGACCAGGTGCCAGTTAAATCAGGATCTGATAGATTATCTAACAGTCCCATAATCCCTAAAGAAATAGAAGAAGTCATTAAAAGTCTCCCCAGCCAGCTGTGCAGTAGTGGCACACACCTT
TAGCTGCTGAAAATCATTTACCAAAATTCAACAGCCCTTCATGGTAAAAGTCTTGGATAGATCAGGAATTCAAGGTCCATACCTAAACATACTAAAAGCAAAATACAGCAAACTAATTGCCAACATCAAACTAAATGGAGAGAAACTTGAAG
CAATCCCACTAAAATCAGGACTAGATAACAAGGCTGCCCACTCTCTCCCTATCTATTCAAATATAGTACTTGAAATTTTAGCTTTAGCAAGTAGAGACAACAAAAGGAGGCAAGGAGATATGAATCTGGAAAAAGAAGGAGTTAAAGTATCA
CTATTTGCAGAGAGGATATGATGGTACACTCTAAGTGACCCCAAACATTCTACCAGAGAACTCCTAAACCTGGATAAACAACTTCAGAGTGGTTGGATATACAATTAATCAAACTGCAGCCTTCCCATAATTGGGAAAAGGATAAACAGGCT
GAGAAAGAAACTAGGAAATGATACCTCTACAGTAGTCACAAATAACATTACATACCTTGGTGTGTGACAACCAAGCAACTGAAAGATTTGTATGAGAAGAACTTCAAGTCTCTGAAGAAACAAATCGAAGAAGATCTCAGAAGATGGAAAGA
GATCGCCAATGCTCATGGACTGGCAGGATTAATATAGTAAAAGGCCATCTTGTCAAAAGCAATCTATAGAGTCAATGCAATCCTATCAAAATTCCAACTCAATTTTCATAGACTTAGAGCAATTTGGCAAATTTATCTGGAATAACAAAGAA
TCCAGAAGATCAAAAACTATTTTCAGTGATAAAAGAACTTGGGAACCAGCATCCCTGACCTCAAGCTGTACTCAGAGCAATAGGGATAAAAACCAAAACAAAACAAAAAAAACAAAAACAACAAACAAAAAAACCATATGGTATTGGTACAGA
GACAGTAGGAAGATCAATGGACTAGAATTAAAGACCCAGAATGAACCCACATGCTCATGGTCACTTGATCTTGATAGAGGAGCTAAAAACCATCCAGTGGAAAAAAGACAGCATTTTCAACAAATGGTGCTGGTTCAACTGGAGGTCAGCAT
GTAGAAGAATGCAAATTGACCTATTCTTGTTTCCTTGTACAAAGCTCAAGTCCAGAAGCTGATAAAGGAATCTAGACATAAACCAAATATACTGGAACTTATAGAGTGAGAAATTGGGGAAAGTCTCAAGATATGGGCACTGTGGAAAATT
TCCTGAAGAGGACATCAATGGCTTATGTCTAAGATCAAGAATTGACAAATGGGACTCATAAAATTGCAAAGCTTTGATAAGGCAAAGAATACTGCTAATAGGACAAATGGCAACTAACAGATTGAGAAAAGATCTTTCCCAATCCTACATCT
GATAGAGGGCTAATATCTCATATATACAAAGAACTCAAAATTTTAGATTCCAGAGAATCAAATAAATTCTATTAAAATAAAAGGTAGCAAGCTAAACAAAGAAATTTCAACTGAGGAATATCAAATGGCCAAGAACCACCTATAGAAATGTT
CAACATCTTTAGTCATCAGGAAATGCAAATCAAAACAATCCTGTGATTCTACCTCACACTATTCAGAATGGCTAAGATCAAAAGCTCATGTGACAAAAGATACTGACAAGGATGTCCGGAAAGAGGAACACTCCATTGCTGGTGGACGCAAG
CCGTGACAAACACTCTGAAATCAATCTGTCGTTCCTCAGAAAATTGGTTATAGTATTACCTGAGTGACCTAGCTATACCAATTCTGGGCATATGCTCCAAAATATCTCTCTAACATATAACAAGGACACATGCTACACTCATGTTCATAGCA
GCCTTGTTTTTATAATAGCCAGGACCTGAAAGAACCTGTGATGTCCTTCATAGAGGAATGACAGTAAATGGTAAATTATACACTGAGAACTTCTCAGCTATTAAAAAAACAATGAATTCATGAAATTTTAGGTAAAATGGATCGAACTAGAA
AATATCATCCTGAGGAGGTAACCCAATCACAAAAAGAATACACATGGTATGCATTCACTGATAAGGAGGTTAGCCCATATGCTCAGAATAAATTCAAGATACAATTGACAGACCACATGAAGCTTATGAAGAAGGAAGACCAAAGTATGGAT
ACTTCTGTCTTTCTTAGAAAGGGGGAATAAAATACTTGTGGAACAAATACAGAGACAAAGTGTGGATAGAGACAGAAGGAAAGACCATCCAGAGACTGCCCCACCTGGAGATCCATACCATATACACAGTCACCAAACACAGACACTATTAT
TGATGTCAAGAAGTACTTGCTGAAAGCAGCCTGATAAGAAATGCCTCGAGAGGTTTCAGCAGAGCCTGACAATACAGAGGTGGATACTCTAAGCCAAATCCATTGGTCTGACCATAGGGTTCACAATGGAGGAGTTAGAGAAATAACTGAAG
GAGCTGAAGGGCTTTTGCAACCATAGGAAGGGACAACACAATAATCCAGCAGATTTCCCACCCCCACCCCAGAGCTCACAGGTGACTAAGCCACCAACCAAGTGAGTACACATAGAGGGACCTATAATTCCAGATGCACAAGTAGCAGAGGA
TGGCTTTGTCACGCATTAATGCAAGAAGAGCCCCTTGGTCCTATGAACTTCGATGGATGTCCCAGTGTAGGGTAATGCCAGGGTGGGGAGGTGGGAATGGATGGGTGGGTGGTGGAACACCTCATAGAAGCAGCGGAAGAGGAGATGGTTTA
AGGAGTTTCTTGGGGTGGTACGAAAGGGAATAACATTTGAAGTATAAATAAAGAAAAATAAATATACAATTAAAAAAAGAAACTGACTTTATTTGAGTGTTTCAAAGTGCTCTGTGATTCTCCTGACAGCTTATCTTAAAAAAATTTTATTA
GATATTTTCTTCATTTATATCAATGCTATCCCAAAGTCCCCTATTACCCCTCCTGCTCCCTACCCACCCACTCCCACCTCTTGGCCCTGGTGTTCCTCTGTACTGGGGCATATACAGTTTGCAAGACTAAGGGGCCTCTTCCCAATGATGGC
CGACTAGGCCATCTTCTGCTACATATTCAGCTAGAGACACGAGCTCTGGGGTACTGGTTAGATCATGCTGTTGTTCCACCTATAGGGCTGCAGACCCTTTCCAGCTCCCTTGGGTATTTTCTCTAGCTTCCCATTGGGGACTCTATGTTTCA
TCCAATAGATGACTGTGAGACCCATTTCTGTATTTGCCAGGCACTGGCAAAGCCCTCACAGGAGACAGCTATATCAGGGTCTTTCAGCAAAATCTTGCTGGCGTAAGTAACAGTGTCAGTCAGAATGGCTAGGATCAAAATTCAGGTGACAG
CAGATGTTATGAGATGTGGAGAAAGAGGAACACCTCCATTGTTGGTGGGACAAGGTAAGCTGGTACAACCACTTGAAATCAGTCTGGCGGTTCCTCAGAAAACTGGACATAGTACTATCGCTAGATCCGGCACAATTTCCTGGGCAACACCC
AGAAGATATTCCAACTGGTAATAAGGACACATGCTCCACTATGTTCATAGCAGCCCCATTTATAATAGCCAGAAGCTGGAAAGAATCCAGATGTCTTTCAACAGAGGAATGATACAAAATGTGGTACATTTACACAATGGAGTACTACTCAG
CAATTAAAAAACAATGAATTTATGAAATTCTTTGGCAAATGGATGTATCTGGAGTGATATTATCCTGAGGAGGTAACCCAATCACAAAAGAAGTCACTTGCTATGCACTCACTGATAAGTGGATATTAGCCCAGAAACCTAGAATACCCATG
ATACAACTTCCAACACACACAAAAAAATTCAAGAAGGAAGACCAATTTGATTACTTCATTCCTCCCTAGAATAGGAACAAAATATCCATGGAAGGAGTTGCAGAGACAAAGTTTGACCTAAGATGAAAGGATAGACCATCCAGAGACTGCTC
CACCCAGGGGGTCCATCCCATAATCAGGCCAATTTAAACGCAGACACTATTGCCTACAGCTTATCTAAACACATCACAACCACAACCTCTCAGGTAACTATACTTGGGACTTAAAAAACATAATCATAATCATTTTTCCTAAAACGATCAAG
ACTGATAACCATTTGACAAGAGCCATACAGACAAGCACCAGCTGGCACTCTAGGTCTTCATGTATGGTCACTGCCTGGGTTCCATTTGTAGAT

Read #18 Alb-chr6-Alb

AGGCTCTTAGCAATGCAAGGCACGTACGTCAGATTTGCCATTACGGGTTTTTATGGCTGCGAAGTGCAAATCCTAACAGTCCTGCTAATACTTTTCTAACATCCATCATTTCTTTGTTTCTCAGGGTCCAAACCTTGTCACTAGATGCAAAG
ACGCTTTCCTAGCTGTGATTCTTCATCAATCTAAGGTATAGTTAACTTAGTGTGAAGAAAGCCTACCTGACCTTCAAAGAGTAGGTTGTTCCTCAGTTTTGATCATCTATAGAGTTGGAAGACAGAGTGACTTGCATGCCTCAGTACGATAT
AGCACTATATTAGTGAAACAACAGTAAGACCAGATGCAAAAAATTAGACAAAGTCTAAACACATCACAAATCCACAACCTCTCAGGTAACTATACCTTGGGACTTAAAAATACAATCATAATCATTTTCCCAAAACGATCAAGGACTGAAAA
TCATTTGACAAGAGCCATACAGATGCTGTCCAGCTGGCACTTTAGGTCTTCACGTATGGTCATCAGTTTGGTTCCATTTGTAGATATGAAAC

Read #19 Alb-chr6-Alb

AGGCTCTTAGCAAGGCACATACATTTTACTATGTCATTGCCTATGGCTATAGAAGTACAAATCTAACAGTCCTGCTAATACTTTTCTAACATCCATGTTTCTTTTTTTTTTTTAGGAACACCAGCTACCCTTCTGACACTTGCCGTTCTGCC
CTTCTATCAATCTAGGCATAGTTAACTTAATTAATGAAAGCCTCACCTGACCTTCAAAGTGGGTTGTGTCTCTGTTTGATCTCATCTATAGGTTGGAAGACAGAAGTGACACAGCATACCTCCAAATGATAGTACTCATATTAGTGAAACAA
ATATAAGACCAGATGCAAAAATTAGATAAGTCTAAACACATCTCACAACACAACCTTCTCAGGTAACTATACTTAGGACTTAAAAAGCTTATAATCATAATCACATTTTTCCTAAACGTCAAGACTGATAACCATTTGACAAGAGCCATACA
GACAAGCACCAGCTGGCACTCTTAGGTCTTCACGTATGGTCATCAGTTTGGGTTCCCATTGTAGATAAGAAAC

Read #20 Alb-chr6-Alb

ATGCAAGGCACGGTAACGTTTACTATGTCATTGCCTATGGCTATGAAGTGAAATCCTAACAGTCCTGCGAGAACTTTCTAACATCCATCATTTCTTTGTTTTCCAGGGTCGGTACCTTGTCACTAGATGCAAAGACGCCTTAGCGTGCACAG
GGTCAAGTCAATTGGAGGTGAAGTGTCCATAAGGTCCAATCATTAACCAAAAATCTATCTCCAATTGATATAACCTTATAAAGAAAAATATTAGTTTACTCCAATGATTTCAGTCGAGTGAAAGGCCAAAACAATTTTCCAAGCAAATGGAC
TCAAGAAACAAGCTGGAGTAGCCATTTATATTGGATAAATCTACTTCCAACCCAAAGTTATCAAAAAAGACAAGGAGGGACACTTCATACTCATCAAAGGTAAACTCTCCAGAGGAAACTCTCAATTCCTAAACACATCACAACCACAACCT
TCTCAGGTAACTATACTTGGGACTTAAAAAACATAATCATAACTATTTTTCCTAAAAACCATCAAAGACTGATAACCATTTGACAAGAGCCATACAGACAGTACCAGCTGGTACTCTTAGGCTTCACGTATGGTCATCAGTTTGGGTTCCAT
TTGTAGATAAGAAACTG

Read #21 Alb-chr6-Alb

CAATGCAAGGCACGTACGTTTACTATGTCATTGCCTGCTGGCTATGGCGCAAATCCCAACAGTCCATAATACTTTTCTAACATCCATCATTTCTTTGTTTTCAGGTCCAAACCTTGTCACTAGATGCAAAGACGCCTTAGCTCCTCACAGGC
CTCAGCCACCTGAAGGCTAGCCTGGGCTGGCGAACAGTCTATCTCTTCTTAGGTGGCTCAGACAGTTGTGTAGGCTCTCTGCCACTGCCTCAGAGTACTTGTAAATCGCATTGCAAAGGAGGGCAAGGTTTATTTGGCCCAGTGTCAGAAGC
TGCACCTGGGTCAGTTGGTCTATTATTCTGAGTAGACATATTGAGTGAGCATAGATGTGGCAAGGAAGATGTGGCAGAGCCAAATATTAAGCTCCTCTTGGGTGACCAGGAACAAAGTTGAGGGCTAGGAAGGACCAGGATTCCATTGTCCC
CTCTGAGCCAAGCATGCTTCACCAGCCTCCTTCCACCAGGCCCTCCTCTTTCTCCATCCAGAGGCCTGACAATGCAAGGCAGGCTTACAACCAGGTCTTCAGCAATGGGCCTTTGAAGATCCAAACTACAACAACCCTACAACAGGCACTGG
CCAGCCATAGCAAGCTCAGTTCTTCAAACACATCACAACCACAACCTTCTCAGGTAACTATATTTGGGATTAAAAAAACATAATCATAACTATTTTTCTAACGATCAAGACTGATAACCATTTGACAAGAGCTATACAGACAAGCACCAGCT

GGTACTTTAGGTCTCACGGTCACTGTCGGCCCCATTTGTAGACAAGAAACTG

Figure S11A. Single Genomic DNA Fragment Insertion: The displayed read sequences show the insertion of a single piece of genomic DNA fragment at the Alb site (continued).

Read #22 Alb-chr6-Alb

AGCACCTCCAGGCTCTTAGCAATGCAAGGCACGTACGTTTACTATGTCATTGCCTATGGCTATGAAGTGCAAATCCTAACAGTCCCAAGTATAATACTTTTCTAACATCCATCATTTCTTTGTTTTCAGGGTCCAAACCTTGTCACTAGATG
GCAAAAGAGCCAAGGTACCACTCCTATCCCCAAACATTCTTGGGTATTTCTATCTAAGAGCTGTACAAATCGTCCCTAAAATTTTTGAGTATTCTAGTTAGAAGATGACAAACTGTGTACGTATGCCTAAAAACACATCACAACCACAACCT
TCTCAGGTAACCATACTTGGGACTTAAAAACATAATCATAATCATTTTTCCTAAAACGATCAAGACTGATAACCATTTTGACAAGAGCCATACAGACAAGCACCAGCTGGCACCTTCTTAGGTCTTCACGTATGGTCATCAGTTTGGTTCCA
TTTGTAGATAAGAAACTGAGGTGC

Read #23 Alb-chr7-Alb

GTGACCAGAGCAATGCAAGCACGTACGTTTACTTATGTCATTGTTTATGGCTATGAAGTGCAAATCCTAACAGTCCTGCTAATACTTTTCTAACATCTATCATTTCTTTGTTTTCAGGGTCCAAACCTTGTCACTAGATGCATTTTAACCAA
GAGATAGACTCAAGATGTTCAGAGGGAAGAGGGAAGAAGCTCCTAATTATAGAGCATCACTATGCCAGGTATAAAAGAACGTGCAAAGTAGGGCTAGGGGTGCAGCTCATAGAAGCCCTAAAACACATCACAAAATCCACAACCTTCTCAGG
TAACTACTCTGGGACTTAAAAACATAATCATAATCATTTTTCCTAAAACGATCAAGACTGATAACCATTTGACAAGAGCCATACAGACAAGTACCAGCTGGCACTTCCAGTCTTCTGTATGGTCACTGTCGGTTCCATTTGTAGATAAGAAA
C

Read #24 Alb-chr7-Alb

ACACCTCAGTGACCAGAGCAATGCACGAGAACACGTACGTTTACCTGTATGCATTGCCTATGGCTATAAGAGTGCAAATCTATGCTGATCTGCTAATACTTTTCTAACATCCATCATTTCTTTGTTTTCAGGGTCCAAACCTTGTCACTAGA
TGCAAAGACGCCTTAGCACAAGTGTTGTCTGGAATTAGGGAGAGAAATGCAGGAGCACTGCTGAAAGCACAGGCTCTTCTTAAAATCAGTATTTCCACCACTGTTTTTTTTTTTTTGAAGAGTCTTTCCATGTTGGTCTTGAGCTCATAATC
TACTGAGAGAAAGGGTCTCTCTCTCTCTCTCTCTCTCTCTCTCTCTCTCTATATATATATATGTGTGTGTGTGTGTGTGTGTGTGTGTGTGTGTGTGTGTGTAGTGGTGAGTGTGTGTGTGTGTGTGTGTGTGTGTGTGTGTGTGTGTGGTGTGTG
TGTGTGTTTGTTAGTTTGTTTGTTTGGTGGGTTTCAACTTTCTTTTGGGTTCCTAAACACATCACAACCACAACCTTCTCAGGTAACTATACTTGGGACTTAAAAAACATAATCATAATCATTTTTTCCTAAAAACGATCAAGACTGATAAC
CATTTGACAAGAGCCATGCAGACAAGCACCAGCTAACTCTTGGAGATAACTGCGTATGGTCATCAGCTTGGGTTCCATTTGAATGAAACTGGGGTGC

Read #25 Alb-chr7-Alb

AGTCAATGAGCAATGCAAGGCACTATGTTTACTAGGTCATTGCCTATGGCTATGAAGTGCAAATCCTAACAGTCCTGCTAATACTTTTAACATCCATTTATTTCTTTGTTTTCAGGGTCCAAACCTAGCTGGATATAAAATTAACTCAAACA
AGTCAATGGCCTTTCTCTACACAAAGAATAACAGGCTGACAAAGAAATTAGGAAACAATACCCTTCTCAATAGCTCACAAATAATATAAAATACCTTGCGTGACTCTAACTAAGACCTAAACACATCACAACCACAACCTTCTCAGGTAACT
ATACTTGGGACTTAAAAAACATAATCATACGATTTTTCCTAAAATGATCAAGAACTCATAACCATTCTACAAGAGCCATACAGACAAGCACCAGCTGGCAACTCAGGTTCTCATGCACTGGTCATCAGTTTGGGTTCCATTTGTAGAATAAG
AAAC

Read #26 Alb-chr8-Alb

CAGTGACCAGAGCAATGCAAGGCACGTACGTTTACTTCGGTGTCATTGCCTATGGCTATGAAGTGCAAATCAAAATAGTCCTGCCAGGTACTTTCTAACATCCATCATTTCTTTGTTTTCAGGGTTATTGTCTCAGCGAGCGGACATATATT
TGAATGTATTTAGAAAATAAACAAATAGGGGTTCCGCGCACAGGCATGGTGCCGGAGCAGGAGCTGAGAGCGTCACATCTCTAACCTGCATTGGCAGGTGAGAGAGCAGAACTGCACTTGGCATGGGCTTTCTAAACCTCAAAGCGCACTCA
CAGTGACACACCTTCAACAAGGCGAGGCCCTCAACAACCCAAACACATCAAAATCCACAACCTTCTCAGTAACTATACTTGGGACTTCAAAAACATAATCATAATCATTTTTCCTAAAACGATCAGACTACAACCATTTGACAAGAGCCATA
CAGACAAAGCACCAGTTGGCACTCTGTAGTTCTCACGTATGGTCATCAGTTTGGTTCCATTTGTAGATAAG

Read #27 Alb-chr8-Alb

AATGCAAGGCACTGTACGTTTACTATGTCATTGCCTATGGCTATGAAGTGCAAATCCCAACAGTCCTGCTAATACTTTTCTAACAGATCCATCATTTCTTTGTTTTCAGGGTCCAAACCTTGTCATCAGATGCAAAGACGCCTTTTAGTGTT
TGAAGATGGAAAGCCACATCAAGGCTAAAGGTAACATTGTTTGTTTGTCAAGTCACCAGAGTTGCCAGAAAATCTGGTAGATGAATCAACCCGAATTTTTTTCCCAAAGATATAAAAGGGACTTCTTAAGAGAGAAACTTCAATGTTGTAGA
TAATAAATCTCAAATTTCACTCTGTAGTTTAACTCAATGATAGATCTTAGGAGTACAAATCATGTTATCATTCTAAGTTGCTTCTGGAAAACAATAAATTTAACAAAAAGTACTTAAAAATACTGTTAAGCATAAACTGATGTAAAAATCTA
GTTGTCATCAGATCAAATATTAAAGACATTCACAACAGACAATGCTGTTATTTAAAATATATTAAAATCCATGAATTTAATTGTTTTGGAACCCATTTATAAATATATAAATAAATTGTCATATATTGCAATATTCAATGTATGTTAATCCA
GATCACTGCTTAAAGTAAAAGAAGAAATTCAATTATTATTTTTTTTATGTTTAGTTGTTGATGCTTTTGTTTGTTTTGCTCTGTTTCTGGGTTGACCTCCTCACTCAAAAACTTTTTTATCTAAATCTTCTGGAATAATGTGTCTGTGTGTG
TGTGCATGTGTGTGTGTGTGTGTGTGTGTGTGTGCCTAAACACATCACAACCACAACCTTCTTTGTGTAACTATACTTGGGACTTAAAAACATAATCATAATCATTTTTCCTAAAACGATCAAGACTGATAACCATTTGACAAGAGCCATAC
AGACAAGCACCAGCTGGCACTTTAGGGTCTTCATAATGTATGGTCACTGTCAGTTCCATTTGTAGAT

Read #28 Alb-chr8-Alb

GCACCTCCAGGCTCTTAGCAATGCAAGGCACGTACGTTTACTATGTCATTGCCTATGGCTATGAAGTGCAAATCCTAACAGTCCTGCTTTAATACTTTTCTAACATCCATCATTTCTTTGTTTTCAGGGTCCAAACCTTGTCACTAGATGCA
AAGACGCCTTAGTCTTATGTGTAGAAGGCAGATTCTTGTGCAAACAAGATTTGGGGACAAATTAGCTTATAATACAAGCAGCATTAGACCAAACCCACTACACTAACTTGTTAATCATTTGGGTCAATCTAATTTCCCTGTCTAATTAATGA
AATTTGTGATAAGCTCTGAGATAAGAGTACTTATTATTTCTAAAAAAAAAACCCAAATATTTTGGTTTTTTTCACAATAAAATAAATATGTCTAGTAAATGAACAGTTGAAGGCATAGCAGTGAAGTCTGACAAATGAACATCTAAAAACTT
CTATCAAAAAATCATCAAAACTGTACTGTGGACTTAAATTACATACACAGATCATGTACAACATACATAGTTTCACTAAAGAGGCCCTTAATGGACAATTGACTCTGAGGGTGTGTGTGTCTGTCTGTGTGTGTGTGTGTGTGTGAAGAGCA
GGAGACAAGAGTAAGCCAGGCTTTCAACGTGTTTTGTTTCTATGAATACACCAGGTACCTTCACGTTTTTGCCTAAACACATCTATACAACCTTCTCAGGTAACTATACTTGGGACTTAAAAAACATAATCATAATCATTTTTTCCTAAAAC
GATCAAGACTGATAACCATTTGACAAGAGCCATACAGACAAGCACCAGCTGGCACTCTTAGGTCTTCACGTATGGTCATCAGTTTGGGTTCCATTGT

Read #29 Alb-chr9-Alb

CCTCCAGGCTCTTAGCAATGCAAGGCACGTACGTTTACTATGTCATTGCCTATGGCTATGAAGTAAATCCCAACAGTCCTGCTAATACTTTTCTAACATCCATCATTTCTTTGTTTTCAGGTCCAAACCTTGTCACTAGATGCAAAGACGCC
TTATAATAACACAGGGTAGCTCCAAGGGTTTGTGCTACCTCTAGATATTCTGCTGTCTCCTTGCTGCCTTTCTGATGATAATCCTGATTTTTTAAAAATTCAGTTTTTACCATTACATGTCGAAACATTTTTATGACATCTCTGTTTTGAAT
AGATTTGCTACTTTCTTGATGTCAAAGCCCTAAACACATCACAACCACAACCTTTCTCAGTAACTATACTTGGGACTTAAAAACATAATCATAATCATTTTTCCTAAACGATCAAGACTGATAACCATTTGACAAGAGCCATACAGACAAGC
ACCAGCTGGCACTCTTAGGTCTTCACGTATGGTCATCAGTTTGGGTTCCATTTGTAGATAAGAAAC

Read #30 Alb-chr9-Alb

GCAATGCAAGGCACGTACGTTTACTATGTCATTGCCTATGGCTGCGAAGTGCAAATCCTAACAGTCCTGCTAATACCTTCTAACATCCATCATTTCTTTGTTTTGGGTCCAAACCTTGTCACTAGATGCAAAAGATTTTAGAGGGCTGAATC
CAGGCACCTTGCATCAGGAAGTGGGATCATCATACCGCTCATCTCTTACAGCAGGGTTCAAACCTCTAATTCCGGGACCCTTTAATACAGTTCCCATATTGTGGTGACCCCCCAACCATAAAATTACTTCATTGCTACTTCATAACTTTAAT
TTTGCTACTGTTATGAATGATAATGTAAATATCTGATAGTAACATCTGTGGGGGTTATAACCACAGGTTGACAACCATTGCTCTTCACAACCACAACCTTCTCAGGTAACTATACTTGGGACTTAAAACATAATCTATAATCATTTTTCCTA
AAACGATCAAGACTGATAACCATTTGACAAGAGCTATACAGACAAGCAATTGCTGGTAATTTTAGGGTCTTCAATGTATGGGTCATCAGTTTGCCCATTTGTAGAC

Read #31 Alb-chr9-Alb

CCGTGTGAAGCAATGCAAGGCACGTACGTTTACTATGTCATTGCCTATGGCTATGAGTGCAAAAACCGAACAGTCCTGCTAATACTCTTGACACTCCATCATTTCGTTCAGGGTCCAAACCTTGTCAACTAGACGCAAAGACGCCTTAGAAA
TGAGAAACATCCACTTGACGACTTGAAACTTACAAAATTCCTGAATAACGTGAAAAATGAGAAATGCACACTGTAGGACCTGGAATATGGCGAGAAAACTGAAATCACGGAAAATGAGAAATACACACTTTAGGATGTGAAATATGGGGACA
GAAACCTCAAAAAGGCCTAGACACATCACAACCACAACCTTCTCAGGTAACTATACTTGGGACTTAAAAAACATAATCATAACATCTTTGAAAACGATCAAGACTGATAACCATTTGACAAGAGCCATACAGACAAGCACCAGCTGGCACTC
TTAGGTCTTCAATGTATGGTCATCAGTTTGGGTTCCATTTGTAGATAAGAAACTG

Read #32 Alb-chr9-Alb

GCACCTCGACTAGACCAGCAGTAAGGCACGTACGTTTACTATGCTCGTACCTACACCAGCTGTAAAATTCTGGAAATCCTAACAGTCCTGCTAATACTTTTCTAACATCCATCATTCTTTATTTCCAGGGTCCAAACCTTGTCACTAGATGC
AAGGCCAACAGCAAGGTGGAAAATTTAGAAATGTTCACTGTAGGACGAGAAATATAGCAAGAAAACTGAAAATCATGGAAAATGAGAACCATCCACTTGACGGCAGAAAATGACGAAATCACTAAAACATGAAAAATAGAAATACACACTGA
GACCTGGAATACCAAGCGCCTAAACACATCACAACCACAACCTTCTCAGGTAACTATACTTGGGACTTAAAACATAATCATAATCATTTTCCTAAAACGATCAAGACTGATAACCGGTGACAAAGACCATGCAGACGAAGCACCAGCTGCTC
TTGAGTCTTCGTATGGTCATCAGTTTGAGTTCCATTTGTAAGATAAGAAACTGAGGTGC

Read #33 Alb-chr9-Alb

GCACCTCCAGGCTCTTAGCAATGCAAGGCACGTACGTTTACTATGTCATTGCCTATGGCTATGAAGTGCAAATCCTAAATAACAGCGTCCTACACGCTCTCTTCTAACATCCATCATTTCTTTGTTTTCAGGGTCCAAACCTTGTCACTAGA
TGCAAAGATGCCTTAGCAACAGAAATGCACACTGAAGGACCTGGAATATGGCGAGAAAAAACTGAAAATCACGTAAATGAGAAATACACACTTTAGGACGTGAAATATGAAGAGGAAAAAACTGAAATGCTGGAAATTTAGAAATGTCCATC
AGTAGACATGGAATATGGTAAGAAAAACTAAAAAAATCATGGAAAATGAGAAACATCCACTTGACGACTTGAAAATGACAAAATCACTAAAACGTGAAAAACGAGAAAATGCCCACTGAAGACCTGGAATATGGCGAGAAAACTGAAAATAA
CAGAAAATGAGAAATACACACTTTAGAATCTAGGTAACTATACTTGGGACTTAAAAAACATAATCATAATCATTTTTCCTAAAACGATCAAGACTGATAACCATTTGACAAAGGAGCCGAAGTACAGACAAGCACCAGCTGGCACTCTTAGG
TCTTCGCCATAATACCAGTTTTAGTTCCGCATTTAGATGAAACTGAGGTGC

Read #34 Alb-chr9-Alb

GGCTCTTAGCAATGCAAGGCACGTACGTTTTACTATGTCATTGCCTATGGCTATGAAGTGCAAATCCTAACAGTCCTGCTAATACTTTTCACAACATCCATCATTTCTTTGTTTTCAGGGTCCAAACCTTGTCACTAGATGCAAAGACGCCT
TGATCTTAGAGTGGAGAACATGGATCTGGAGCCCACATGCCTGGGTTTGCATCTGAGCTCTGCCATGTATAGCAAGGTTATGACTTTGGGGTTCTCTGGCTTTCTACGACTCAGTCTTCTTTCTGGTGAAATACTGTAACTGAGAAATGCCT
CACAGCGTTTTTGTGAGGTTGGATAACTAGGTCTAGTCTGCAGCATCCACGCCAGTTATCACCTCCTAAACACATCACAACCACAACCTTCTCAGGTAACTATACTTGGGACTTAAAGCTTATAATCATAATCATTTTTCCTAAAACGATCA
AGACTGATAACCATTTGACAAAACCATACAGACAAACACCAGCTGGCACTCTTAGGTCTTCACGTATGGTCATCAGTTTGGGTTCCATTTGTAGATAAGAAAC

Figure S11A. Single Genomic DNA Fragment Insertion: The displayed read sequences show the insertion of a single piece of genomic DNA fragment at the Alb site (continued).

Read #35 Alb-chr11-Alb

GACACGGTAGCAATGCAAGGCACGTACGTTTACTTCGTGTCATTGCCTATGTATTATGCTGCAACTTCTTAACAGTCCTGCTAATACTTTTCTAACATCCATCATTTCTTTTGTTTTCAGGGTCCAAACCTTTGTCACTAGATGCAAGACGT
CTTAGCCCCAACATTGCACCAAGGTAGGGAGAACTCTCACTTTTTCAAATAAGGAGGCTCATTGAGTGATGTGGCACATACCTTTGTTCCCAGCACTCAGGAGGCAGAAGCAAGTGGATCTCTGAGTTTGAGACCAATCTGGTCTATTTAAC
AAGTTCCAGACCAGCCAAGTTACATAGTGAGCCTAAACACATCACAACCACAACCTTCTCAGGTAACTATACTTGGGACTTAAAAAACATAATCATAATCATTTTTCTCCTAAAACGATCAAGACTGATAACCATTTGACAAGAGCCATACA
GACAAGCACCAGCTGGCACTCTTAGGTCTTCACGTATGGTCATCAGTTTGGGTTCCATTTGTAGATAAGAAAC

Read #36 Alb-chr11-Alb

CCGTCACAAGCAATGCAAGGCACGTACGTAAGTTTTTGGGCATTATGTCATTGCCTATGGCTATGAAGTAAATCCCAACAGTCCTTGCTAATACTTTTCTAACATCCATCATTTCTTTGTTTTCAGGGTCCAAACCTTTGTCACTAGATGCA
AAGACGAGACCTGCCTGCTTCTGCCTCCCGAGTGCTGGGTTTATTTTATTTTTTTTATTTTTTTGGTTTTTCGAGACAGGGTTTCTCTGTGTAGCCCCTGAGTGCTGGGTTTAAAGGCATACCTAGTCCTGGTTCTGAACTCTTTTTTTTCT
TTTAAAACATGTCCTAAACACATCACAACCACAACCTTCTCAGGTAACTATACTTGGGACTTAAAAACATAATCATAATCATTTTTCCTAAAACGATCAAGACCGTGATAACCATTTGACAAGAGCCATACAGACAAGCACCAGCTGGCACT
CTTAGGTTCCTCACGTATGGTCATCAGTTTGGGTTCCATTTGTAGATAAGAAAC

Read #37 Alb-chr12-Alb

AGTCAATGAGCAATGCAAGGCACGTACATTTACTATGTCATTGCCTATGGCTATGAAGTACCAAATCCTAACAGTCCTGCTAATACTTTTCTAACATCCATCATTTCTTTGTTTTCAGGGTCCAAACCTTGTCTAGATGCAAAGACGCCTTC
CCTGCAATAAAGGCTGTTGAGAAGAATCTGACCATGTCGTGTCTTCCTTGCTGGCTGAGGTGGGCGCGACACCCCATGGCCCTTCAGCGAAACAGAGATTTTTTCTAAACACATCACAACCACAACCTTCTCCAGGTAACTATACTTGGGAC
TTAAAAACATAATCATAATCATTTTTCCTAAAACGATCAAGACTGATAACCATTTGACAAGAGCCATACAGACAAGCACCAGCTGTACTCTTAGGTCTTCACGTATGGTCATCAGTTTGGGTTCCCATTGTAGATAAGAAACT

Read #38 Alb-chr14-Alb

CAATGCAAGGCACGTATTGTTTACTATGTCATTGCCTATGGCTATGAAGTGCAAATCCTAACAGTCTCTGCTAATACTTTTCTAATATCCATCGTATTTCTTTGTTTTCAGGTCCAAACCTTGTCACTAGATGCAAAGACGCCTTAGCTGGC
AATCCACATAGCTCTCAGCCCCATCCAGACAGGTTAAAGGTAAGCAGAAGGTGGGGACAGCAGTGCAGTGTGACAGAAAGTAAGGATTCAGGCCACAGCCTCCCTGAAGGACTTACCCAGAAAGCCAAACTTGTGCCTAAACACATCACAAC
CACACCTTCTAGGTAACTATACTTGGGACTTAAACATAATCATAAATCATTTTTCCAAAACGATCAAGACTGATAACCATTTGAAGAGCCATACAGACAAGCACCAGCTGGCACTTCTTAGGGTCTCTCTGCCAGAGTTGTCATCAGTTTGG
GTTCCATTTGTAGAT

Read #39 Alb-chr15-Alb

GCACCTGCAACAGAGGAGCAATACGAAGGCACGTACGTTTACTATGTCAGAGCTGCTATATGAAACATGAAGTGCAAATCCTAACAGTCCTGCTAATACTTTTCTAGCATCCATCATTTCTTTGTTTTCAGGGTCCAAACCTTGTCACTAGA
TGCAAAGACGCCTGTCTACTTCCTTTTTAAGTCTAGTATGGTTTTGTTCATTTCCATCACCTGTTTGGATGTGCTTTCCTGTTTTTTCTTTAAGGACTTCTACCTGTTTGATTGTGTTTTCCTTTTTTCTTTAAGGACTTGTAACCTAAACA
CATCACAACCACAACTTCTCAGGTAACTATACTTGGGACTTAAAAAAACATAATCATAATCATTTTCCCTAAAACGATCAAGACTGATAACCATTTGACAAGAGCCATACAGACAAGCACCAGCTGGCACTCTTAGGTCTTCACATGTGGTC
ATCAGTTTGGGTTCCATTTGTAGATAAGAAACTGAGGTGC

Read #40 Alb-chr16-Alb

ACTAGACCAGCAATGCAAGGCACGTACGTTTACTATGTCATGCCTATGGCTATGAAGTGCAAATCCTAACAGTCCTGCTAATACTTTTCTAACATCCATCATTTCTTGTTTCAGGGTCCAAGCCTTGTCACTAGATGCAAAAAGACGCCTTG
GATCAAGTCTCTGCAGGATTAGGCTCATCCTCTCCATATTCCAGACAAGGCAGCCCTCTGGTGCATGTGTTGCGGTCCTTGGTCCAGCCTATATGCTCTTTGAGTGGTGGCTCATTCTCTTGAAATTCCCAGGAGTCCAGGTTAGTTGTCTA
AACACATCACAGCCACAACCTTCTCAGGTAACTATACTTGGGACTTAAAAAAACATAATCATAATCATTTTTCCTAAAACGATCAAGACTGATAACCATTTGACAAGAGCCATACAGACAAGCACCAGCTGGCACTCTTAGGTCTTCACGTA
TGGTCATCAGTTTGAGTTCCATTGTAGATAAGAAACTGACG

Read #41 Alb-chrX-Alb

GCACCTCCAGGCTCTTAGCAATGCAAGGCACGTACGTTTTACTATGTCATTGCCTATGGCTATGAAGTGCAAATCCTAACAGTCCTGCTAATACTTTCTAACATCCATCATTTCTTTGTTTTCAGGGTCCAAACCTTGTCACTAGATGCAAA
GACGCCTTAGGGGTTCTTGTGGCGGGGCTGAAAGGGCTTCTAACAGATGCCCCACCAATTTGGCTAAGGTAAGGGTTTTTTTTTTATGACCTGTTGAACCACCGTCTGTGTTTCTTTAATGTTTAATGGAGGTTTATTGGTGTTATACTCCC
TTTGGCCTAGCCTCTGAGGGTCAACAACTAACCCATCACAACCACAACCTTCTCCAACGAGTAACTATGCTTGGGACTTAAAAAACATAATCATAATCATTTTTCCTAAAACTGATCAAGACTGATAACCATTTGACAAGAGCCATACAGAC
AAGCACCAGCTGGCACTCTTAGGTCTTCACATGCTGGGTCATCAGTTTGGAGTTCCATTGTAGATAAGAAACTGAGGTGC

Figure S11A. Single Genomic DNA Fragment Insertion: The displayed read sequences show the insertion of a single piece of genomic DNA fragment at the Alb site (continued).

Read #42 Alb-chr10-chr3-Alb

CCAAATGCAAGCAATGCAAGGCACGTACGTTTACTATGTCATTGCCTGTGGCTGTGAAGTGCAAATCTAACAGTCCTGCTAATACTTTTCTAACATCCATCATTTCTTTGTTTTCAGGGTCCAAACCTTGTCACTAGATGCAAAGACGCCTT
AGCATCCGCTGACCATCTTTGAAACTCAGATAATCACAGCCACGATGACTCTCAGGGTGCTTTCTCTCCATCCAGTGTTGGCCAGAGTTGATGCAGAAAGGTGGAGTCAAAGCAAAGGGAGGATCATACCACTGAGTGCTTCCCAGGATTTT
AGGCTAGTAAAAAAAAAAAACACAATCAATTAGTAAATAACCTAATGGATGTTAGCAGAGGAGCTAGATGCACAGAGAGGGGAGAGTGTCTGGGGTCAGGGCATAATATCTCTAGATGAGATTTTACCTAGCAGAAGGAGTCCTAAACCATC
ACAACCACAACCTTCTCAGGTAACTATACTTGGGACTTAAAAAACATAATCATAATCATTTTTCCTAAAACGATCAAGACTGATAACCATTTGACAAAGAGCCATACAGACAAGCACCAGCTGGCACTCTTGGAGTCTTCACGTATGGTCAT
CAGTTTGGGTTCCATTTGTA

Read #43 Alb-chr2-chr9-chr13-Alb

CGACACTAGCAATGCAAGGCACGTACGTTTACTTGCGTCATTGCCTATGGCTATGAAGTGCAAAATCCTAACAGTCCTGCCAATACTTTTCTAACATCCATCATTTCTTTGTTTTCAGGGTCCAAACCTTGTCACTAGATGCAAAGACGCCT
TAGCTTGCTTGAGAAAAGGAAAGCAAGAAGGAGAGATGATGAATGCCATGGCATGAGTGTCAAGGTCACACACAAACACATTCTCTGCTCCTATTACATGGATCCCAATGTTGAACTTAGGTCCCTCAGGCTTGGCAGAAAACACAATTGCT
TCCTCTGGCATCTCACCAGCACCAACATTTAACATGTAAGAGAGGGATTGCCAAATATCAACAAGAAAGAGGAGGCTTCAAGGGAGGGGAAAGAGCCTGGGGCTCTCTGAAGGTCAAGTGCAATCTTTCAGCTTTGGAACCAATGGTCACAG
TGCCCTCTCTACCCACAATATTTCTTTCTGTTATGACCACGAATGTTCTTCAGGATGCTACCAAGCTTAAGGAGCTCTCTTTGTACAGACGACCAGAGCGGAATACTACGAGCTCTTTGCTTGTCTGGCTCTTTATCACACTACATGTTCTC
TACAAGCACAGAAATGTACTTTTCTTTTTTTTTTTTTTTTTTTTTTTTTTTTTAAACCTAAACACATCACAACCACAACCTTCAGGTAACTATACTCTGGGGGGGGGACTTTAAAAACATAATCATAATCATTTTTCCTAAAACGATCA
AGACTGATAACCATTTGACAAGAGCCATACAGACAAGCACCAGCTGGCACTCTTAGGTCTTCACGTATGGTCATCAGTTTGGGTTCCATTTGTAGATAAGAAACT

Read #44 Alb-chr5-chr1-chr2-chr8-Alb

GCACCTGTAGTCAATGAGCAAATGCAAGGCACGTATTGCTTTACTATGTCATTGCCTATGGCTATGAAGTGCAAATCCCAACAGTCCTGCTAATACTTTTCTAACATCCATCATTTCTTTGTTTTCAGGGTCCAAACCTTGTCACTAGATGC
AAAGACGCCTTTAAAACACGTGGATCTATGTCACAGTGTTGTGTTACTATTGCACTGAAGATTTCTGGCTTAGTACAAGCAGTAGAGGTAATACTAGAATTATAGATACTTTCTGGACAACCATACTAGGAACAGCGCAAAGTTAATTGCTA
TGCTATAAACATGCACCATTTAAATTCTGAGTTGGGAGCATCTGGCAAGGTACTTGACTTATAAGTTTATAAGGAGACTTGTTCACCTTGCCTCTAAGTTAGGTAAATGTATAATTAAATTTACAAAATATGACTTGGAGAAACCCAAAGCA
AAGGAATGTAATTAGTTTTATGAAAAAAATTAAACTCTAGAAGTATTTTAGCATGTTGCATGTCTCCAATAGGTCACCCATGTTTTACACATAAATGCAATAAAAATGAAAACATTCATGCTACTGTTTCCTAGTAATCAATAAAATGACTC
TAGAGATTTTAAACAATATTCAAGTTAACAAAAGTATAGGTGTATAGGTTTTCAAATTTGCCACGGAACATACTATGTAACATTTCTCTATGTAACTATACAGACATAAGACAGTGCATCTTGCTTGAGGCTCTTGCTTGAGTACTGCATAC
ATAATTGTGGCAGGCTGCTTTGCTCCAACTGACCGCAGTTGAGCCTGCAAATAGAAGGAAGAGTGCCCTGCAACAGGGGAAATGTATTGAGCAGTATTTAATAAAAAAGAAGGAAATTGGTTTAGGAGAAAATGTCAACATTCACTCTGGAC
TATGTTTGCAAACCTAGCTCTTCAAGTCAGTACCTAAACTCTGTTCTTATGCTTGTTCATCAAACCCTTTCCTGACTGAACTATCAACCTGGTCCTTGCCTCCTATTTTTAATGTTTCCTGAAAAAAATAGGACAGCAGTTTAATCTTAGCC
ACAAAAGTTGAAGCAACATTAAATTATTAAAAAAAAATGTTCACTAACAGAATGCACGGTGGGTTTCTCTTCACGACACGTACTTGTGTTTTTTCTGTATCTCCATAGTCTCTACACCTTACTCTAAGGCAGAGTCCAATGTCTGTGTCTGG
CCAGCCATGTGCCAAAGTTTTATACAGAATGATGCAATAACTGGAGTATGAATGACTAATTTAAACAGTTGGAAAAACCTCTAATAAAGAACCCAAATCATTGCAAGATTCTAAAGCACATAGGTTTTTCCTGTAAGAAAGAAAATTGAAAG
CTATGCTTTTGATATCCTAATGACTATTTTTTAAATAAAATAGGCTTTAGATTTTAAATTACTGAATAATTTAAATACAATCTTCTGCACCTCCTTCTTTCCCCTACTGTTTTCTATATTTCGATGTGCCATAAAGGCCTCAGCTCATATGT
CTACTTTATTTTGGGGTTTTTAGACTCTAGACCATGAACTATACATTTTTTCTTTATGAATTACTTACATTCTGGTTTTCGGTTATAACAGCAGAAAATTGAGTAAGACTGCCATAATTAGGTTTACTGCTGTGAACAGATACCACGACCAA
GGCAAGTCTTATGATGAAAACAAATATTTAATTGGGGCTAGCTCACAGGTTCAGAGGTTCAGTCCATTATCATCAAGGTGGGAACATGGCAGTATCCAGGCAGGCAGAGCTGAGAGTTCTATATCTTCATCCAAAGGCTGCTAGGGAGAAGA
CTGACTTCAAGGCACCAGCAGGGCGGCCAAGCCCCACACCCACAGTGACACACCTACTCCAACCATGTCACACCTATTCCAACAAGGCCACACCTCCGGGCAGCCACTCCCTGGGTCCAAGAATATCCCTTAAACCATCACAAAGACAAATC
TATTTTATGTTAGCATTCACCACATAGTATGTTTCTGAGGGAATAACATCTCTGGTGCTCAACATGTGCAAAGATGCTATATTAAAAACAGGTGTGACAGAGTCCATCTCTGATCAAGGGGGATACAAAATTGGAAAGGGAGTGGGAAGTCA
AAATATCACTTTTTGCAGATGATATGATAGTATATATAAGTGACCCTAAAAATTCACCAGAGAACTCTTAAAACCCATAAAACAGTTTGGGCAGTAGCTGGACATAAAATTAACTCAAACAAGTCCAGTCTTCTTTCTCTCTCTTTTTTTTT
TTTACAAAAGAATAAACAGGCTGAGAAAGAAATTAGGGAAACAACACCCTTCTCAATAGTCACAAATAATATGTACATTCTATGACTTCCTAACTACAAAGTGAAAGATCTGTATGATAATAAATCTCAAGTCTCTGAAGAAAGAAATTAAA
GAAGATCTCAGAAGGACACATCCTCCCATGCTCAAGTGATTGGCAGATCAATACGCAAAAATGGCTATCTTGCCAAAAGCAATCTACAGATTCAATGCACACCCCATCAAAATTCCAACTCAATTCTTCAACAATTAGAAGGAGCAATTTGC
AAATTCATCTAGAATAAACAAAAAACCTAGGATACAAAACTCTCTCTCTCAAGGATAAAAGAACCTCTGGTGGAATCACCATGCCTGACCTAAAGTTTATTACAGAGCAATTGTGGTAAAAACTGCATGGTACTGGTATAGAGACAGACAAG
TAGACCAATGGAATAGAATTGAAGACCCAGAAATGAACCCACACACCTATGGTCACTTGATCTTCAGACAAGGGAGCTAAACCATCCAATGAAGAAAGACAGCATTGCACAATTGGTGCTGGTAACAAACCTGGTTGTTATCATGCAGAAGA
ATGCGGATCGATCCATACCTATCTCCTTGTACTAAGGTCAAATCTAAATGGATCAAAGAACTTCACATAAAACCAGAGACACTGAAACTTATGAGAAAGTGGGGGAAAAAGCCTGAAGATATGGGCACATCAAGAAATCATGCAACCTCAGG
CAACTATACTGGGACTAAAAACATGAATCATAAATCATTTTCCTAAAACGATCAAGACTACAACCATTTGACAAGAGCCATACAGACAAGCACCAGTTGGCACTTCCTGAAGCCTCACGTATGGTCATCAGTTTGGGTTCCATTTGCAGATA
AGAAACTGAGGTGCTGC

Figure S11B. Multiple Genomic DNA Fragments Insertion: The illustrated read sequences demonstrate the insertion of multiple pieces of genomic DNA fragments at the Alb site.

Read #45 Alb-BB-chr9-Alb

ACTAGCAATGCAAGGCACGTACGTTTACTATGTCATTGCCTATGGCTATGAAGTGCAAATCCTAACAGTCCTGCTAATACTTTTCTAACATCCATCATTTCTTTGTTTTCAGGGTCCAAACCTTGTCACTAGATGCAAAGACGCCTTAAGA
TAACTACGATACGGGAGGGCTTACCATCTGGCCCCAGTGCTGCAATGATACCGCGAGACCCACGCTCACCGGCTCCAGATTTATCAGCAATAAACCAGCCAGCCGGAAGGGCCGAGCGCAGAAGTGGTCCTGCAACTTTATCCGCCTCCAT
CCAGTCTATTAATTGTTGCCGGGAAGCTAGAGTAAGTAGTTCGCCAGTTAATAGTTTGCGCAACGTTGTTGCCATTGCTACAGGCATCGTGGTGTCACGCTCGTCGTTTGGTATGGCTTCATTCAGCTCCGGTTCCCAACGATCAAGGCGA
GTTACATGATCCCCCATGTTGTGCAAAAAAGCGGTTAGCTCCTTCGGTCGTGAAAAATGAGAAATGCACACTGAAGGACCTGGAATATGGCGAGCAAACTGAAAATCACGGAAAATGAGAAATACAAACTTTAGGACTTGAAATATGGCGA
GGAAAACTGAAAAAGGTGGAAAATTTAGAAATGTCCACTGTAGGAGCTTGGAATATGGCAAGAAAACTGAAAATCATGGAAAATGAGAAACATCCATTTGACAACTTGAAAAATGACGAAATTACTAAAAAACGTGAAAGGAGAAATGCAC
ATTGAAGGACCTGGAATATGGCAAGAACTGAAAATCACCAAAAATGAGAAATACACACTTAAACACATCACAACCACAACCTTCTCAGGTAACTATACTTGGGACTTAAAAAACATAATCATAATCATTTTTCCTAAAACGATCAAGACTG
ATAACCATTTGACAAGAGCCATACAGACAAGCACCAGCTGGCACTCTTAGGTCTTCACGTATGGTCATCAGTTTGGGTTCCATTTGTAGATAAGCA

Read #46 Alb-chr1-BB-Alb

GCACCTCACTAGACCAGCAATGCAAGGCACGTACGTTTACTATGTCATTGCCTATGGCTATGAAGTGCAAATCCTAACAGTCCTGCTAATACTTTCTAACATCCATCATTTCTTTGTTTTCAGGGTCCAAACCTCCCGCACCTTGTGTTCC
AAATACCTAGGGACCTACGCTCACTCTGTCATTCATCATTCAGCTAGGAGGTAGAACTGCTCTGCAGTAGTTCACAGATCAACCATCAGGATGTTTAGCTGCATCTATCATTTTTATGCTACTTTAAAAATAAAAGAACTGCAAATTTCCC
ACCTCCCTGTTTCTAAAAACTGTATTCCTACAGCTGACTTAAACTCTTCTGCTTTAAGTAGAGAAGCAACACCCCCTTTACTGGGATTGGGGAGCTTTGCCTATTTCTACTGTCATTTTATTTCTGGAAAGAAATCCAGGTGGGAGGGTGA
GAGCAGAGTTAGGTCGGTGGAGCGAGAAATCAAATACTAACCTGTAGGCAGTTGAAGCCTAAGGGTAGAGGAGTAGGATATAAAAGGCACTGTTATAAAGGCAACAGGAGAGAAAGCCTCAGAAGTTAAGACCACAAGAAGAAGAAAATGT
CACTCACTAAGGATGCCTAAGCAGATGAGCAGTGTCAGAGAGAAGCATGATGTTAAGGGGAGAGTCTTTACAGGGAGGTCATCAGATGACTGCAGAAAATATTGCACAATAAAGAAACATCCATGGAACACTTTTCTCATGAAGGCATTAT
TATCTTGCTGCCTTTTAGCCAAAGAAGCTCTTAGGTCCAATTCCTGGTCAAACTCGAGATCATTCTGTCACTGAATGGAACACGATGCCATCAAAACACACTGAACAGCTACTGCATAGAAAGAGTTGAATCCTTGCCTCCCAGGAGCTTC
TGGATGGAAAGAGAGAAGAGGCAACCCCAAACAAATCTAATATTGACTCATTGGTTTGGCTACTGTGTGCTCAAATTATCCTGAGCTCAGTATCGGGCTGAACAGATGGACATATGAGCTAGCAACAACAGGACATTTCCTCAGCTCCTAG
AAAAACACACGATTTAGAGACGACAGTCAAACAAATAACCACACGTTAAATTGTAGAATTGTCAAGTATGGGCTATCAAGCTGGGTCTCAGAGAACTACAGAATCCTATCCTGTGGGATTTGATCTGCACCACCTGTGTGAAATTGTTATC
CGCTCACAATTCCACACAACATACGAGCCGGAAGCATAAAGTGTAAAGCCTGGGGTGCCTAATGAGTGAGCTAACTCACCCGACCAGGCAAGCGAGCTCACTGCCCGCTTTCCAGTCGGGAAACCTGTCGCCAGCTGCATTAATGAATCGG
CCAACGCGCGGGAGAGGCGGTTTGCGTATTAAGTGCGCTCTTCCGCTTCCTCAAGCTCACTGACTCGCTGCGCTCGGTCGCTCGGCCGGTGCAGCAGCGCATCAGCTCACTCAAAGGCTATGAGAAGGCTTTGCTATCCACAGAATCAGGA
TAACGCAGGAAAGAACATGTGAGCAAAAGGCCAGCAAAAGGCCAGGAACTGTAAAAAGGCCGCGTTGCTGGCGTTTTTCCACAGCCCCCGCTCCCCTGACGAGCATCACAAAAATCGACGCTCAAGTCAGAGGTATGAAACCTGACAGACT
ATAAAGATACCAGGCGTTTCCGAAGCCCTCGTGCGCTCTCCCCGTTCCGACCCTGCCGCTTACCGGATACCTGTCCGCCTTCTCCTTTCGGGAAGCGTGGCGCTTTCATAGCTCACGCTGTGGTGTATCTCAGTTCGGTGTAGGTCGTTCG
CTCCAAGCCGGGCTGTGTGCACGAACCCCCCGTTCAGCCCGACCGCTGCGCCTTATCCGGTAACTACCGTCTTGAGTCCAACCCGGTAAGACACGACTTATCGCCACTGGCAGCAGCCACTGGTAACAGGATTAGCAGAGCGAGGTATGTAG
GCGGTGCTACAGAGTTCTTGAAGTGTGGTGGCCTAACTACGGCTACACTAGAAGAACAGTATTTGGTATCTGCGCCTGCTGAAGCCAGTTACCTTCGGAAAAAGAGTTGGTAGCTCTTGATCCGGCAAAATTAAACCACCGCTGGTAGCGG
TGTTTTTTTGTTTGCAAGCAGTAGATTACGCGCAGAAAAAAAGGATCTCAAGAAGATCCTTTGATCTTTTCTACGGGGTCTGACGCTCAGTGGAAAACGAAAACTCACGTTAAGGGATTTTGGCCGCGAGATTATCAAAAGCGATCTTCACT
CTAGATCCTTTAAATTAAAAATGAAGTTTAAATCAATCTAAAGTATATATGAGTAAACTTGGTCTGACAGTTACTAATGCTTAATCAGTGAGGCACCTATCTCAGCGATCTGTCTATTTCGTTCATCCATAGTTGCTCTGACTCCCCCGTC
GTAGATAACTACGATACGGAGGGCTTACCATCTGGCCCCAGTGCTGCAAATGATACCGCGAGACCTGACGCTCACCGGCTCCAGATTTATCAGCAATAAACCAGCCAGCCGGAAGGGCCGAGCGCAGAAGTGGTCCTGCAACTTTATCCGC
CTTCCATCCAGTCTATTAATTGTTGCTGAAGCCGAGTAAGTAGTTCGCCAGTTAATAGTTTGCGCAACGTTGCTGGGCATTGCTACAGGCATCGTGGTGTCACGCTGTCGCTCTGGTATGGCTTCATTCAGCTCCGGTTCCCAACGATCAA
GGCGAGTTACATGATCTCATGTTGTGCAAAAAGCGGTTAGCTCCTTCGGTCCTCCGATCGTTGTCAGAAGTAAGTTGGCCGCAGTGTTATCACTCATGGTTATGGCAGCACTGCATAATTTCCTCTCTTACTGTCATGCCATCCGTAAGAT
GCTTCTGTGACTGGTGAGTACTCAACCAAGTCATTCTGAGAATAGTGTATGCGGCGACCGAGTTGCTCTTGCCCGGCGTCAATACGGGATAATACTCGCGCCACATAGCAGAACTTTAAAAGTGCTCATCATTGGAAAACGTTCTTCGGGG
CGAAAACCTCAAGGATCTTATCGCTGTTGAGATCCAGTTCGATGTAACCCACTCGTGCACCCAACTGATCTTCAGCATCTTTTACTTTCACCAGCGTTTCTGGGTGAGCAAAAACAGGAAGGCAAAATGCCGCAAAAAAGGGAATAAGGGCG
ACACGGAAATGCTGAATACTCATACTCTTCCTTTTTCAATATTATTGAAGCATTTATCAGGTTATTGTCTCATGAGCGGATACATATTTGAATGTATTTAAAAATAAACAAATAGGGGTTCCGCGCACATTTCCCCGAAAAGTGCCACCTG
GTGGTGCAGATGAACTCCTTTTAAAACACCATTATCACAACCACAACCTTCTCAGGTAACTATACTTGGGACTTAAAAAACATAATCATAATCATTTTTCCAAAACGATCAAGACTGATAACCATTTGACAAGAGCCATACAGACAAGCAC
CAGCTGGCAATTTTAGGTCTTCGCCGTATGGTCATCAGTTTGGGTTCCATTTGTAGATAAGAAACTGAGGTGC

Read #47 Alb-chr3-BB-Alb

GCAAGGCACGTACGTTTACTATGTCATTGCCTATGGCTATGAAGTGCAAATCCTAACAGTCCTTAAAGAAGAAACACACACACACACACACATACACATACACACATACACACACACAAAGAATTAACAACAACAACAACAAAAAAAAACC
CTTAAAGAATTACAAGAAAACACAAACAAACAGGGTAGTAGAATTGGAATAAAAACCATCCAAGACCTAAAAATGGAAGTAGAAAAAACAATAATGAAAACCCAAAGAGAGACAACTCTGGAGATAGAAAAACCCCAGGAAAGGAATCAGG
AACCATAGATGCAAGCATCAGCAACACAGAAAAACAAGAGCAGATAGAAGAGAGAATCTCAGGCAGACAGGGGGAATGCCAGGGCCAAGAAGTGCCTCTCCCCGCGTTGGCCGATTCACATGTACAATGCAGCTGGCACGACAGGTTTCCT
GACTGGAAAGCGGGCAGTGAGCGCAACGCAATTAAGGTGAGTTAGCTCAATTAATAGGGCAAGGCACCCCAGGCTTTACACTTTATGCTTCCGGTTTTTCCCCCGTATGTTGTGTGGAATTGTGAGCGGATAACAATTTCACACAGGTGGC
AGATGAACTCCAAACACATCACAAACTACACAACCTTCTCAGGTAACTATACTTTGACTTAAAAAAACATAATCATAATCATTTTTCCTAAAACGATCAAGACTGATAACCATTTGACAAGAGCCACAGACAAGCACCAGCTGGCACTCTT
AGGTCTTCTACGTATGGTCATCAGTTTGGGTTCCATTTGTAGAT

Read #48 Alb-chr13-BB-Alb

CTCTCAGGAGCAATGCAAGGCACGTACGTTTACTATGTCATTGGATGATAATGGACTAAATCACTAAATTGTAAGTCAGTCCCAATTAAATGTTTGCTTTATTGGAATTATGTTAGTTGGCGGTGTCTTTACAGGGAATGAAACTCCAACT
AAGATAATGAGAAACCTCTTAGGACTCATTATCGCAGCAACGCGGCCTTTTTACGGTTCCTGGCCTTTTGCTGGCCTTTGCTCACATGTTCTTTTCCTGCGTTATCCCCCTGATTCTGTGGATAACCGTATTACCGCCTTTGAGTGAGCT
GGATACCGCTCGCCGCAGCCGAACGACCGAGCGCAGCGAGTCAGTGAGCGAGGAAGCGGAAGAGCGCTCAATACGCAAACCGCCTCTCCCCGCGCGTTGGCCGATTCATTAATGCAGCTGGCACGACAGGTTTCGACTGGAAAGCGGGCA
GTGAGCGCAACGCAATTAAGGTGAGTTAGCTCACTCATTAGGCACCCCAGGCTTTACACTTTATGCTTCCGGTTCGTATGTTGTGGAATTGTGAGCGGATAACAATTTCACACAGGTGGTGCAGCCTAAACACATCACAACCACAACCTTC
TCAGGTAACTATACTTGGGACTTAAAAACATAATCATAATCATTTTTCCTAAAACGATCAAGACTGATAACGTATTTGACAAGAGCCATACAGACAAGCACCAGCTGGCACTCTTAGGGTCTTCACGTATGGTCATCAGTTTGGGTTCCAT
TTGTAGATAAGAAAC

Figure S11C. Plasmid Backbone and Genomic DNA Fragments Insertion: The presented read sequences reveal the insertion of both plasmid backbone and genomic DNA fragments at the Alb site.

Read #49 Alb-F8-chr7-Alb

TCAATGAGCAATGCAAGGCACGTACGTTCACGGATGTGTCATTGCCTATGGCTATGAAGTGCAAATCTGAGACAGTCCTGCTAAGACTTTTCTAACATCCATCATTTCTTTGTCTTCAGGGTCCAAACCTTGTCACTAGATAGGTTTCATC
TGTGTATGCCAGATAAATTGGTCTTTGTACTCCTACCAGTTGCGAGGGCCATTGTTCAAATATTGACTTTCATAAACTCTCGTCATCGGGGTGAGGCACTAAGGAGCATACCCAGTCCTCCTCTTCAGCAGCAATGTAATGTACCCAAGTT
TTAGGATGCTTCTTGGCAACTGAGCGAATTTGGATAAAGGAAGGAGAGTTGTCATCATCAAACCTGACCACATCCATTTCAGAATCAGTAAGATCATCATCATAGTCTTCCGCTTTTCATCATTCTTCATTCGTAGTTGGGGTTCCTTGGA
CAGCTGTTGACTTTGACATAACTTCCATGCTCATCATGTTGGTGGGAAGAATATATGACAAAACAGTAGAAACTGTCGAGGTCGATCAAGAGTGTTTGAGCAGTAAGGAAGTTATTGGCGAGATTTCCAAGGACGCCTGGCGATGGTCTCA
GCACAAGAAGTGTGCCACTCTTCCAGGAATATTGAGTGCACTTCAGGAAGCGGTGCCCATTCCATCACATGCCAATAGACTGATTTCCTGTGGCATCCAATCAGACCTGGCAGAGACCTGTTTACATAACCATTGATGCGTGGATTCAGGC
CAGGCCCCTGAGCAGATGCAGCATCCCATCCTGCATCAAGGAGTTCTTTGTTTCTGAGTGCCAACTTTTCCTTCATCAAATACAGCAAAAGTAGTATAAATTTGTGCAAAGGTCTGTGCCCTTTCCTTGGCCAACACTCCTCTCTCACATA
CGAATAGGGCTCCAACCAGCGAATTCAAGTCTTTTACCAGGTCCACATGAGAAAGATATGAAGTAGGAAGGCACAGTGGGTCAGAGGCCATTGGACCATTCTCTTCAGGACCTGCCAGACATATGTATGGCTTCCACCAGGGAAGACTTTA
TCATCTTCTTTCTCCCTTTGACTGGTCTGATCATCATATTCAGCTCCCTCAGAAGCTTTCCAGTAGGAACAGACACCAACAGCATGAAGACTGACAGGACGGGAAGCCATGTTCTTAGTGTAATTACCACTGTATCATAAACCAGCCTGGA
TGGTAGGACCTAGCAGACCCATCCAGGGTGGCCTTGGCTTAGCGATGTCTAAGGTGATCCGTAACCACAAACAGAGTCTTTTTGTACACGACTGAGGTGTTGAATGAAAAGATTTTGTACTTAGGAGGAAATCTTGCGTCCACAGGCAGCT
CACCGAGATCACTTTGACATAGTCCCACTACAGTTCCACTGCACCCAGGTAGTATTTAGACAAGGCCTGGCTATGTACAGAGCTACCCATTCACCCTTCCTCAGAGAGCTCAGGTTTGCGGTAACCCTCCTGGTTCACCTTTGTTTGATTT
GTTGTTTGTTAGACAGATCAAGCATTACGGTGTACTTCAGACCTCCCGCCTCAGGATCCTGGGTAGCTGGAATTATAAGTCTGCATTAGCAAGTCTATGTTTGCCATTATATTAAAGTAAAAATACAAATGGAAAATCAAAAAGGTGATTA
TGACTTGCCCAACACATTAGTCAATGCAAAGACAAGCCATCCTCAGGTTAGCAACTCCAAAATCCTGGCCCTCTGGGCACGCTCAGATGCCTCTTTGAGTCAGATGCTCCTGGGCAGGGTGGTACATTTCTCAGTATGAATGAATCTAATG
ATATTAATTTATATGTAGCCAATATTAACAAATGGGGTTAAGATGTACATAGTACACGTGCAAGTACACACACTTGAACACACATGCACATGCACACATACAAGGATACTTCAATAAAAACTGGAAAGTCACAAAACTTTTTTCACTGTTA
TCATCTACTTATATTCAGATAGGTAACTTATGGTATGTTCTAAACAGACACTTAGAATGGGTGTGCCTCAGATGCAGAACTGGAAGCTGAAAGCAATTATATAGACAATTCACATTTGAGAGTCATGAGGATGGTAACTCTTTACAATTAA
TAAATTGGATGAAACACAATCAGTAAATTGAACAAATCCTTTCTCCAGGTACTATACTTGGGACTTAAAAAACATAATCATAATCATTTTTCCTAAAACCATCAAAGACTGATAACCATTTGACAAGAGCCATACAGACAAGCACCAGCTG
TACTTTAGGCTTCCACGTATGGTCATCAGTTTGAATTTCATTTGTAGATAAGAAAC

Figure S11D. F8 and Genomic DNA Fragments Insertion: The depicted read sequence showcases the insertion of both F8 and genomic DNA fragments at the Alb site.

Read #50 Alb-F8-chr3-BB-Alb

GAATCAGGCAGCAATGCAAGGCACATGCGTTTTACTATGTCATTGCCTATGGCTATGATACAAATCCTAACAGTCCTGCTAATACTTTTCTAACATCCATCGTTTCTTTGTTTTCAGAGGGTCCAAACCTTGTCACTAGATGCAAAGACGC
CGGGGAGCTGCATGTGTCAGAGAGTTTTCTACGTCATCTTGAAACACTTGCCACGAGGCAATAGGATCATAATCAGCCATACCACATTTGTAGAGGTTTTGCCCCCTTTTAAAGCCTCCCACCTCCCCTGAACCTGGAAACATAAAATGAA
TGCAATTGTTATTTGTTAACTTGTTTGTGCAGCTTATAATGGTTACAAATAAAGCAATAGGCATGCAAATTTCACAAATAAAGCATTTTTCCTGCATTCTAGTTGTGGTTTGTCCAAACTCATCAATGTATCTTATCATGTCTGGATCCGC
CCAAAGGGAGATCCGACTCGTCTGAGGAGCGAAGGCGAGACGCGGAAGAGGCCGCAGAGCCGGCAGCAGGCCGCGGGAAGGAAAGGTCCGCTGGATTTGGGGGCCGAAGGGACGTAGCAGAAGGACGTCCACGCAGAATCCAGGTGGCAAC
ACGGGCGAGCAGCCATGGAAGGACGTCGGCTTCCCGACAACACCACGGTGTCAGTGCCCAACAGCTGAGCCCCTGTCCAGCAGCGGGCAAGGCGGAAAGCGATAGCGGAAGGTTCCGCCGTGGCAATAGGGAGGGGGAAAGCGAAAGTCCC
GGAAAGGAGCTGACAGGTGGTGGCAATGTCCCAACCAGTGGGGTTGCGTCAGCAAACACAGTGCACCACGCCGTTACCCTGACAACGGGCCTAGCTCCCTCATAAAGAGACAGCAACCAGGATTTATACAAGGAGGAGAAAATGAAAGCCA
TACAAGGAAGCAATAGCATGATACAAAGGCATTAGAAGCAGCGTATCCGCGTAAAAAGGAGCAACATAGTTAAGAATACCAGTCAATCTTTCACAAATTTTGTAATCAGAGGTTGATTGTCGACGTTTTAAACTCAGTAGAGTCCTGTACT
TGCCGGCCCAGAACCTCCATCCTCAGGGCAATCTGGTGCACCCAACTCTGGGAGGTGAATTCAGGTAGCAGGTCAGTAACGGTGGGTCAGAGAGTTCACCACAGGTGTGAAGGAGTCTTGATTTCCCTGAAAAACCTTTACTTTTGCCATT
CTGAAAAAGAGAGTCCACTGATAATACATCTTGACTGCTGGAGATGAGAACTCCTTCACATACATGCTGGTAAGCAGAGATTTTACTCCTAGTAGTTACTCCTGTGACTTTCATTGTCTTCTGGAAGTCCACTTGCAGCCACTCTTTTGGA
TTATTCACCTGGGAGTCTCAGGCATTTACTCCTCCCTTGGAGGTGAAAGTCAGGCTTTTGAAGGAGACCAGGTGGCAAACATGTGTGGTAAAATGGGGATGAAGCAGTAATCTGTGCATCTGATATTTGCTTTACTCTCCATTTCCCAATG
GCATGCTGCAGCTATTTAAATCACAGCCCATCAACTCCATGCGAAGAGTGCTGCAGATATATAATGAGTTAGGTGCAAACGGATGTATCGAGCAATAATTGGAGGGTTAAAAATATTGTGTTTTATCCCAGATGAATCCACATTGCCAAAG
AAGACCATTAAGGTTCCAGTGGAATTTCCTCGATAAGTCTGCCACTTCTTCCCATCAAGACTATACATGATAAACTGAGAGATGTAGAGGCTGGAGAACTTCTGACAGACACCCTGGGTCTTGATGCCGTGAATAATCATTTGGTGCCAAC
AAGATCCACCTTGATCCAAGAAAAGGGCTCCTTGGTACTCCAGGCATTGATTGATCCGGAATAATGAAGTCTGGCCAACTTTGGGGCCCACTGTCCATATTGTCCTGAAGCTGTAATCTGAGGAAATCTCATGTGTCAGAAGCCATTTCAG
GGGAGTCTGACACTTATTGCTGTACACCAGAAAAAGTGTACTCATCCCAGCATGTAGATGCTCGCCAATGAGCATTCCACCCATGGAAATTCCAGCTTTGGATGGTAACATTCCACTGTCTCAAAAACACCTGGATAGAGATTGTACAGTG
CCATTTTATACTCCTCTTTTTTTTCGTACAGTGAACACATGTCCACTGAAATGAATAGAATGGATGTTTTTCATTGCTGCCCATGCTGAGCAGATACCATCGAATCCTTTGATCCTGAGCCATTACTAAGCCAGGTAGTGTATCCATTATG
TAGCCATTGATTGCATGGAAGCGATAATTCTCTTTAAAAGTGGGATCTTCCATCTGGATATTGCAGGGAGAGTCCTTAAAGAAGAAACACACACACACATACACATACACACATACACACACACAAAGAACAACAACAACAACAAAAAAAA
ACCCTTAAAAATTGCAAGAAAACACAAACAAACAGGTAGTAAATTGAATAAAACCATCCAAGACCTAAAAATGGAAGTAGAAACAATAATGAAAACCCAAGAGAGACAACTCTGGAATGAAACCCCAGAGAGGAATCCCAGGAACCATAGT
GCAAGCATCAGCAACAGAAAACAAGAGATAGAAGAGAGAATCTCAGGTACAGGAGGAATGCCAGGGCCAAGAAGTGCCTCTCCCCGCGCGTTAGCCGATTCATTAATGCAGCTGGCACGACAGGTTCCCGACTGGAAAGCAGACGGTAGGC
ACGCAACGCAATTAAGGTGAGTTAGCTCACTCATTAGGCACCCCAGGCTTTACACTTTATGCTTCCGGCTCGTATGTTGTGGAGATTGTGAGCGGATAACAATTTCCACACAGGTGATGCAGGTATCGAACTTATTTCATCACAACCACAA
CCTTCTCAGGTAACTATACTTGGGACTTAAAACATAATCATAATCATTTTTCCCTAAAACGATCAGACTGATAACCATTTGACAAGAGCCATGAACCAAGCACCAGCTGGCACTCTTAGGTCTTCACGTATGGTCATCGGTTTGGGTTCCA
TTTGT

Figure S11E. F8, Plasmid Backbone, and Genomic DNA Fragments Insertion: The exhibited read sequence displays the insertion of F8, plasmid backbone, and genomic DNA fragments at the Alb site

cgactcgagactcgagccccgggctaccttgccagcagagtcttgcccaacacccgcaagggtccacacgggactccccacgggaccctaagacctctggtgagtggatcacagtgcc
tgccccaatccaatcgcgcggaacttgagactgcggtacatagggaagcaggctacccgggcctgatctggggcacaagtcccttccgctcgactcgwgactcgagccccgggctacc
ttgccagcagagtcttgcccaacacccgcaagggtccacacgggactccccacgggaccctaagacctctggtgagtggatcacagtgcctgccccaatccaatcgcgcggaactyga
gactgcggtacatagggaagcaggctacccgggcctgatctggggcacaagtcccttccgctcgactcgagactcgagccccgggctaccttgmcagcagagtcttgcccaacaccc
gcaagggcccacacgggactccccacgggaccctaagacctctggtgagtggaacacagcgcctaccccaatccaatcgcgtggaacttgagactgcggtacatagggaagcaggct
acccgggcttgatctggggcacaaaccccttccactccactcgagccccggctaccttgccagctgagtcgcctgacacccgcaagggcccacacaggattccacacgtgatcctaaga
cctctagtgagtggaacacaacttctgccaggagtctggttcgaacaccagatatctgggtacctgccttgcaagaagagagcttgcctgcagagaatactctgcccactgaaactaag
gagagtgctaccctccaggtctgctcatagaggctaacagagtcacctgaagaacaagctcttaacagtgacaactaaaacagctagcttcagagattaccagatggcgaaaggcaa
acgtaagaatcctactaacagaaatcaagaccactcaccatcatcagaacgcagcactcccaccccacctagtcctgggcaccccaacacaaccgaaaatctagacccagatttaaaa
acatttctcatgatgatgatagaggacatcaagaaggactttcataagtcacttaaagatttacaggagagcactgctaaagagttacaggctcttaaagaaaagcaggaaaacaca
gccaaacaggtgatggaaatgaacaaaaccatactagaactaaaggggaagtagacacaataaagaaaacccaaagcgaggcaacgctggagatagaaaccctaggaaagag
atctggaaccatagatgcgagcatcagcaacagaatacaagaaatggaagagagaatctcaggtgcagaagattccatagagaacatcgacacaacagtcaaagaaaatacaaaa
tgcaaaaggatcctaactcaaaacatccaggtaatccaggacacaatgagaagaccaaacctacggataataggaattgatgagaatgaagattttcaacttaaagggccagctaat
atcttcaacaaaataatagaagaaaacttcccaaacataaaaaaagagatgcccatgatcatacaagaagcatacagaactccaaatagactggaccagaaaagaaattcctcccg
acacataataatcagaacaacaaatgcactaaataaagatagaatattaaaagcagtaagggagaaaggtcaagtaacatataaaggaaggcctatcagaattacaccagactttt
caccagagactatgaaagccagaagagcctggacagatgttatacagacactaagagaacacaaatgccagcccaggctactatacccggccaaactctcaattaccatagatggag
aaaccaaagtattccacgacaaaaccaagttcacacaatatctttccacgaatccagcccttcaaaggataataacagaaaagaagcaatacaaggacggaaatcacgccctagaac
aaccaagaaagtaatcattcaacaaaccaaaaagaagacagccacaagaacagaatgccaactctaacaacaaaaataaaagggagcaacaattacttttccttaatatctcttaat
atcaatggactcaattccccaataaaaagacatagactaacagactggctacacaaacaggacccaacattctgctgcttacaggaaacccatctcagggaaaaagacagacactac
ctcagagtgaaaggctggaaaacaattttccaagcaaatggactgaagaaacaagctggagtagccattttaatatcggataaaatcgacttccaacccaaagttatcaaaaaagac
aaggagggacacttcatactcatcaaaggtaaaatcctccaagaggaactctcaattctgaatatctacgcaccaaatgcaagggcagccacattcattagagacactttagtaaagct
caaagcatacattgcacctcacacaataatagtgggagacttcaacacaccactttcttcaaaggacagatcgtggaaacagaaactaaacagggacacagtgaaactaacagaagt
tatgaaacaaatggacctgacagatatctacagaacattttatcctaaaacaaaaggatataccttcttctcagcacctcacgggaccttctccaaaattgaccatataattggtcacaa
aacaggcctcaatagatacaaaaatattgaaattgtcccatgtatcctatcagaccaccatggcctaagactgatcttcaataacaacataaataatggaaagccaacattcacgtgga
aactgaataacactcttctcaatgataccttggtcaaggaaggaataaagaaagaaattaaagactttttagagtttaatgaaaatgaagccacaacgtacccaaacctatgggacac
aatgaaagcatttctaagagggaaactcatagcgctgagtgcctccaagaagaaacgggagacagcacatactagcagcttgacaacacatctaaaagccctagaaaaaaaggaa
gcaaattcacccaagaggagtagacggcaggaaataatcaaactcaggggtgaaatcaaccaagtggaaacaagaagaactattcaaagaattaaccaaacgaggagttggttctt
tgagaaaatcaacaagatagataaacccttagctagactcactaaagggcacagggacaaaatcctaattaacaaaatcagaaatgaaaagggagacataacaacagatcctgaa
gaaatccaaaacaccatcagatccttctacaaaaggctatactcaacaaaactggaaaacctggacgaaatggacaaatttctggacagataccaggtaccaaagttgaatcaggat
caagttgaccatctaaacagtcccatatcacctaaagaaatagaagcagttattaatagtctcccaaccaaaaaaagcccaggaccagatgggtttagtgcagagttctatcagacctt
caaagaagatctaattccaattctgcacaaactatttcacaaaatagaagtagaaggtactctacccaactcattttatgaagccactattactctgatacctaaaccacagaaagatcc
aacaaagatagagaacttcagaccaatttctcttatgaatatcgatgcaaaaatcctcaataaaattctcgctaaccgaatccaagaacacattaaagcaatcatccatcctgaccaag
taggttttattccagggatgcagggatggtttaatatacgaaaatccatcaatgtaatccattatataaacaaactcaaagacaaaaaccacatgatcatctcgttagatgcagaaaaa
gcatttgacaagatccaacacccattcatgataaaagttttggaaagatcaggaattcaaggcccatacctaaacatgataaaagcaatctacagcaaaccagtagccaacatcaaag
taaatggagagaagctggaagcaatcccactaaaatcagggactagacaaggctgcccactttctccctaccttttcaacatagtacttgaagtattagccagagcaattcgacaacaa
aaggagatcaaggggatacaaattggaaaagaggaagtcaaaatatcactttttgcagatgatatgatagtatatataagtgaccctaaaaattctaccagagaactcctaaacctga
taaacagcttcggtgaagtagctggatataaaataaactcaaacaagtcaatggcctttctctatacaaagaataaacaggctgagaaagaaattagggaaacaacacccttctcaat
agtcacaaataatataaaatatcttggcgtgactctaactaaggaggtgaaagatctgtatgataaaaacttcaaatctctgaagaaagaaattaaagaagatctcagaagatggaa
agatctcccatgctcatggattggcaggatcaacattgtaaaaatggctatcttgccaaaagcaatctacagattcaatgcaatccccatcaaaattccaactcaattcttcaacgaattg
gaaggagcaatttgcaaatttgtctggaataacaaaaaacctaggatagcaaaaagtcttctcaaggataaaagaacttctggcggaatcaccatgccagacctaaagctttactaca
gagcaattgtgataaaaactgcatggtactggtatagagacagacaagtagaccaatggaatagaattgaagatccagaaatgaacccacacacctatggtcacttgatcttcgacaa
gggagctaaaaccatccagtggaagaaagacagcattttcaacaattggtgctggcacaactggttgttatcgtgtagaagaatgcgaatcgatccatacttatctccttgtactaaggt
caaatctaagtggatcaaggaacttcacataaaaccagagacactgaaacttatagaggagaaagtggggaaaagccttgaagatatgggcacaggggaaaaattcctgaacaga
acagcaatggcttgtgctgtaagatcgagaatcgacaaatgggacctaatgaaactccaaagtttctgcaaggcaaaagacaccgtcaataagacaaaaagaccaccaacagattg
ggaaaggatctttacctatcctaaatcagataggggactaatatccaacatatataaagaactcaagaaggtggacttcagaaaatcaaataaccccattaaaaaatggggctcaga
actgaacaaagaattctcacctgaggaataccgaatggcagagaagcacttgaaaaaatgttcaacatccttaatcatcagggaaatgcaaatcaaaacaaccctgagattccacctc
acaccagtcagaatggctaagatcaaaaattcaggtgacagcagatgctggcgaggatgtggagaaagaggaacactcctccattgttggtgggagtgcaggcttgtacaaccactc
tggaaatcagtctggcggttcctcagaaaactggacatagtactaccggaggatccagcaatacctctcctgggcatatatccagaagatgccccaacaggtaagaaggacacatgct
ccactatgttcatagcagccttatttataatagccagaagctggaaagaacctagatgcccctcaacagaggaatggatacagaaaatgtggtacatctacacaatggagtactactca
gctattaaaaagaatgaatttatgaaattcctagccaaatggatggacctggagggcatcatcctgagtgaggtaacacattcacaaagaaactcacacaatatgtattcactgataag
tggatattagccccaaacctaggatacccaagatataagatataatttgctaaacacatgaaactcaaggagaatgaagactgaagtgtggacactatgcccctccttagatttgggaa
caaaacacccatggaaggagttacagagacggagtttggagctgagatgaaaggatggaccatgtagagactgccatagccagggatccaccccataatcagcatccaaacgctga
caccattgcatacactagcaagattttattgaaaggacgcagatgtagctgtctcttgtgagactatgccggggcccagcaaacacagaagtggatgctcacagtcagctaatggatgg
atcatagggctcccaatggaggagctagagaaagtagccaaggagctaaagggatctgcaaccctataggtggaacaacattatgagctaaccagtaccccggagctcttgactcta
gctgcatatatatcaaaagatggcctagtcggccatcactggaaagagaggcccattggacttgcaaactttatatgccccagtacaggggaataccagggccaaaaagggggagtg
ggtgggcaggggagtgggggtgggtggatatgggggacttttggtatagcattggaaatgtaaatgagttaaatacctaataaaaaatggaaaaaaa

Figure S12. The consensus sequence of L1MdA_I in the LINE-1 family.

Read #1 Alb-L1-Alb @6054e536-fd9d-4ce6-bf33-b065bed95978

GCGCACCTCAACTCCTGAAGCAATGCAAGGCACGTACGTTTACTATGTCATTGCCTATGGCTATGAAGTGCAAATCTAACAGTCCTGCTAATACTTTTCTAACATCCATCATTTCTTTGTTTTCAGGGTCA
AACCTTGTCACTAGATGCAAAGACGCCTTAGTCACAATTTAGAATTTTTAGGGTCCTTATATATACTATCATATCATCTGCAAAAAGTGATATTTTGACTTCCTCTTTTCAATTTGTATCTCCTTGATCTC
CTTTTGTTGTCGAATTGCTCCTAAACACATCACAACCACAACCTTCTCAGGTAACTATACTTGGGACTTAAAAACATAATCATAATCATTTTTTCCTAAAACGATCAAGACTGATATACATTTGACAAGAG
CCATACAGACAAGCGCCAGCTGGCACTCTTAGGTCTTCACGTATGGTCATCAGTTTGGGTTCCATTTGTA

Read #2 Alb-L1-Alb @88a19c42-27f1-4911-b00a-1e132a1b9c61

ACATGAGGAGCAATGCAAGGCACACTTTTGTTTACTATGTCATTGCACTATGGCTATGAAGTGCAAATCCTAACAGTCCTGCTAATACTTTTCTAACATCCATCATTTCTTTGTTTCAGGGTCCAAACCTT
GTCCACTAGATGCAAAAAGACGCCTTTATAGCTCCTTTTCATTTTCTGGGATGCATTTAATTAGGATACTGTCTCTGTGCCCTCTAGTTAGTCTGGCTAAGGGTTTTATCTATCTTGTTGATTTTCTCAAA
GTACCACTAAACACATCACAACCACAACTTCTCAGGTAACTATACTTGGGACTTAAAAAACATAATCATAATCATTTTCCTAAAACGATCAAGACTGATAGCAAAATTTGACAAGAGCCATACAGACAAGC
ACCAGCTAACTCTTAGGTCTTCACGTATGGTCATCAGTTTTGGTGGGTTCCATTTGTAGATAAGAAAC

Read #3 Alb-L1-Alb @d5a82961-5d4f-4c2e-b8e2-232b26dbf4d8

TTACCTCGATTACCTCAGCAATGCAAGGCACGTACGTTTACTATGTCATTGCCTATGGCTATGAAGTGCAAATCCTAACAGTCCTGCTAATACTTTTCTAACATCCATCATTTCTTTGTTTTCCAGGGGTC
AAACCTTGTCACTAGATGCAAAGACGCCTTAGAAAATCTTGCTAGTGTATGCAATGGTGTCATCGTTTGGAAGCTGGTTATAGGATGGATCCCCAGATATAGCAGTCTCTAGATGGTCCATCCTTTCGTCA
CAGCTCAAACTTTGTCTCTATTAACTCTACAAACACATCACAACCACAACCTTCTCGAGTAACTATACTTGGGACTTAAAAACATAATCACCAATCGTTTTTCCTAAAACGATCAAGACTGATAACCATTT
GACAAAGGCCATACAGACAAAGCACCAGCTGGCATAACAGAGTCTTCACGTATGGTCAATGGTTTGGAGTTCCATTTGTAGATAAGAGCTGAGGTGC

Read #4 Alb-L1-Alb @0d0a64cb-40d1-46c7-bbae-5601d14b962e_R

CGTTACGTGCAAGCAAATGCAAGGCACGTACGTTTACTATGTCATTGCCTATGGCTAAGAAGTGCAAATCCCAGTCAGTCCTGCTAATACTTTTCTAACATCCATCATATTTCTTGTTTTCGTTAGGTCCA
AACCTTGTCAAATACAGAGAGCAATTGTGATAAAAACTGCATGGTACTGTATAGAGACAGACAAGTAGACCAATGGAAGTTAGAACGTCCAAGATCCAGAAATGAACCCACACACCTATGGTCACTTGATC
TTCGACACCTAAACACATCACAACCACAACCTTCTCCTTCTCAGGTAACTATACTTGGGACTTAAAAAACATAAATCATAATCATTTTTTCCTAAAACGATCAAGACTGATAACCATTTGACAGAAGCTTT
TTCATACAGAGAGACAAGCACCAGCTGGCACCTCTTAGGTCTTCACGTATGGTCATCAGTTTGGGTTCCATTTGTAGATAAGAAAC

Read #5 Alb-L1-Alb @845b268b-2288-4aa5-b915-53780c9fa804_R

AGTCAATGAGCAATGCAAGGCACTACGTTTACTAGGTCATTGCCTATGGCTATGAAGTGCAAATCCTAACAGTCCTGCTAATACTTTTAACATCCATTTATTTCTTTGTTTTCAGGGTCCAAACCT~~TGTCA~~
~~CTAGATGCAAAGACGCCTTAG~~AGCTGGATATAAAATTAACTCAAACAAGTCAATGGCCTTTCTCTACACAAAGAATAACAGGCTGACAAAGAAATTAGGAAACAATACCCTTCTCAATAGCTCACAAATAA
TATAAAATACCTTGCGTGACTCTAACTAAGACCTAAACACATCACAACCACAACCTTCTCAGGTAACTATACTTGGGACTTAAAAAACATAATCATACGATTTTTCCTAAAATGATCAAGAACTCATAACC
ATTCTACAAGAGCCATACAGACAAGCACCAGCTGGCAACTCAGGTTCTCATGCACTGGTCATCAGTTTGGGTTCCATTTGTAGAATAAGAAAC

Read #6 Alb-L1-Alb @9aca963b-8fb1-4709-905c-f0ba2759f766

GAGCCATAGCAATGCAAGGCGCGTACGTTTTACTATGTCATTGCCTATGGCTATGAAGTGCAAATCTAACAGTCCTGCTAATACTTTTCTAACATCAAAGCCATTTCTTTGTTTTCAGGGTCAAAACGTAC
TAGATGCAAGACGCCTGGGAAGTAGACACAATAAAGAAAACCAAAAGCATGAGCAACGCTGAAGATAGAAACCCTAGGAAAGAGATCTAGAACCATAGATGCGAGCATCAGCAACAGAATACAAGAAATGG
AAGAGAATCTCAAGGTGCAGAAGATTCCATAGAGAACATCCTAGCTTACATCACAACCACAACTTCTCCAGGTAACTATACTTGGGACTTAAAAAAGAAAACATAATCACTTCAATCATTTTTCCATAAAA
CTGATCAAGACTGATAACCATTTGACAAGAGCCATACAGCCAGACAAGCACCAGCTGGCACTCTTGGAGTCTTCACGTATGGTCATCAGTTTGGGTTCCATTTGTAGATAAGAAAC

Read #7 Alb-L1-Alb @92f1ed7a-d649-470f-bc21-3215f61678e1

GGCTGACAGAGCAATACAAAGGCACATTACAGTTTACTATATGTACCTATGGCTATGAAGTGCAAATCCTAACAGTCCTGCTAATACTTTTCTAACATCATCATTTCTTTGTTTTCAGGGTCCAAACCTTG
TCACTAGATGCAAAGGCATGTAGATATCTATTAAAAATGGACTTCCCAACCCAAAAACTATCAAAAAGACAAGGAAGGACACTTCATACTCATCCAAAGTTAAAATCTTCCAGAGGAACTCTCCAATTCTG
AATATCTATGCTCCAAATGCAAGGGCAGCCACATTCATTAAAGAGACTTTTAGTAAAGCTCAAAGCACATTACCTCATACACAATAATAAATAGGAGACTTTGCCTACTTTCATCAATGGATAGATCCTGG
CAGAAAACCTAAACACATCACAACCACAACCTTCTCAGGTAACTATGTAGGACTTAAAACATAATCATAATCATTTTTTCCTAAAACGATCAAGACTGATAACCATTTGACCAAGAGAGCCATACAGACAG
CACAACTAACACTCTGCAGGTCTTCACATGTGATCATCAGTTTGGGTTCCATTTGTAGATAAGAAAC

Read #8 Alb-L1-Alb @7a05acea-6e10-403a-bfd9-d05e5b25ef5c_R

ACCACTATCAGCAATGCAAGGCACGTACGTTTACTATGTCATTGCCTATGGCTATGAAGTGCAAATCCTAACAGTCCTGCTAATACTTTTCTAACATCCATCATTTCTTTGTTTTCAGGGTCCAAACCTTG
TCACTAGATGCAAAGACGCCTTAGTAAGTATGGATCGATTCACATTCTTCTACATGATAACAACCAGTTGTGCCAGCACCAATTGTTGAAAATGCTGTCTTTCTTCCACTGGATGGTTTTGGCTCCCTTGT
TGAAGATCAAGTGACCATAGGTGTGTGGGTTCATTTCTGGGTCTTCAATTCTATTCCATTGGTCCACTTGTGTGTCTCTATACCAGTACCATGCAGTTTTTATTACAATTGCTCTGTAGTAAAGCTTTAGG
TC~~GC~~TAAACACATCACAACCACAACCTTCTCAGGTAACTATACTTGGGACTTAAAAAACATAATCATAATCATTTTCCTAAAACGATCAAGACTGATAACCATTTGACAAGAGCCATCACGAGCACAAGC
ACCAGCTGGCACTCTTAATGTTCACGTATGGTCATCAGTTTGGGTTTCATTTGTAGA

Read #9 Alb-L1-Alb @05d5093e-f9d6-4514-8d26-c3a74881845f

ATTACCTCAGCAATGCAAGGCACATACGTTTACCTATGTCATTGCCTATGGCTATGAAGTGCAAATCTAACAGTCCTGCTAATACTTTTCTAACATCCATCATTTCTTTGTTTTCAGGGTCCAAACCTTGT
CACTAGATGCAAAGGCATTGCCTTGGAAAATGATCCCCCACAGTGGTCATTTTTGATGGTTGACAAAGGAACTAAAACCATCCAGTGTATAAAGACAGCATTTTCAGCAAATGGTGCTGACAGTTATCATG
TAGAAGAATGCAAGCTGATCCATTCTTATCTCCTTGTACAAAACAAGTCTAAGTGGATTAAGGAACTGCATAAAATAAGAGACACTGAAACTTATAGAGGGGAAAAGTGGGAAGACCCTCAAAGATGTGAG
GAAGGGGAAAAATTCCTTGTGCTGTAAGATTGAGAATGGACAAATGGGACCTCATAAAATTGCAAAGCATCTGTAAAAGACAAGGACACATTTATCAAAAAAGACAAAACTGCAACCAACAGATTGGGGAA
AGATCTTTACCAATCCTATCTCAGATGGGGGCTAATATCCAAGATATATAAAGAAGTCAAGGAAGTTGGTGTGTAGAAAACCAAGTAACACTATTAAAAATGGGGTACAGAAACTAAACAAAGAATTCTCA
AGGAATACCGACTGGCTGAGAAAGCACCTAACAAAATGTTCAACGTCCCTTAATCTTCAGGGAAATGAAAATCAAAACAACCCTAAGATTCCCACCTCACACCAGTCAGAATGGCTAACATCAAAAATTCA
GGTGACAGCAGATGCTGGTAGGGATGTGGAGAAGAGGAACACTCGTCCATTGCTAGTGGGATTATGGCTGGGACAGCCACTGTGGAAATCAGTTTGGCATTTCCTCAGAAAAATTGTTCATAGGTTCTACT
GGAAGGTCCAGCAGTACCACTCCTGGACATTTTACAGAAGATGCTCAACTTGTAATAAGCACACATACTCCACTATGTTCATAGCAGCCTTATTTATAAGAGCAGGAAGCTGGAAAGCAACCAGATGTCCC
TCAACAGAGAAATGGATACAGAAAAATGTGGTACATTTACACAACGGAGCTATTAAAAACTAATGAGTTCATGAAATTCTTAGACAAATGGATGGAACTATAAAATATCATTATGGTAGGGTAACCCAGTC
ACAAAAGAACACACATGATATGCACTCACTGATAAGTGGATATTAGCCAAGTTAAAATACCCACAGATACATTTTACAAAACACATGAAACTCAAGAAAAGGAAGACCAAAGTGGACACTTTGACCCCTTC
ATAGAAGGGAGATAAAAATACCCATGGAAGAAGTTACAGAGACAATAGAAGTTCAGAGCAGAGACTGAAGGAATAACCATCCAGAGAAACTGCCCTACCTGGGATCCACATCCCAAAAAACAACTAAACCC
AGACACTATTGTGGATGCCAACATTAACTTGTTGGCAGGAGCCTAACATAGCTGTCTCCTGAGAGGTTCTGACGGTTCCTGACTAATACAGGAAATGGATGCTCACAGTCATCCATTAGATGGAGCATAGG
GTCCCCAATGGAAAAGGGAGCTAGAGGAGTACCCAGGGAGCTGAAGGTGTATACAATCCCCTAGGAGGGAGGAACAATATAAACTAACCAGTAATCTGCACCACCAGGTGGCACTTTTCAGGGGAAATGAT
GGATGTGAAAGTATTAGCAAGGACTGTTAGGATTTGCACTTCATGGCCGCAGGCAGCTGACATAGTAAACGTACGTGCCTTGCATT

Figure S13. (A) The representative read sequences display the insertion of a single LINE-1 fragment at Alb.

Read #10 Alb-chr1-L1-Alb @450abd0c-3536-4496-b7d3-78d412798077_R

ATGCAAGGCACGGTAACGTTTACTATGTCATTGCCTATGGCTATGAAGTGAAATCCTAACAGTCCTGCGAGAACTTTCTAACATCCATCATTTCTTTGTTTTCCAGGGTCGGTACCTTGTCACTAGATGC
AAAGACGCCTTAGCGTGCACAGGGTCAAGTCAATTGGAGGTGAAGTGTCCATAAGGTCCAATCATTAACCAAAAATCTATCTCCAATTGATATAACCTTATAAAGAAAAATATTAGTTTACTCCAATGAT
TTCAGTCGAGTGAAAGGCCAAAACAATTTTCCAAGCAAATGGACTCAAGAAACAAGCTGGAGTAGCCATTTATATTGGATAAATCTACTTCCAACCCAAAGTTATCAAAAAAGACAAGGAGGGACACTTC
ATACTCATCAAAGGTAAACTCTCCAGAGGAAACTCTCAATTCCTAAACACATCACAACCACAACCTTCTCAGGTAACTATACTTGGGACTTAAAAAACATAATCATAACTATTTTTCCTAAAAACCATCA
AAGACTGATAACCATTTGACAAGAGCCATACAGACAGTACCAGCTGGTACTCTTAGGCTTCACGTATGGTCATCAGTTTGGGTTCCATTTGTAGATAAGAAACTG

Read #11 Alb-L1-chr15-Alb @c7c6b1ba-fd1e-4ca1-aded-92dd0734e698

GCACCTGCAACAGAGGAGCAATACGAAGGCACGTACGTTTACTATGTCAGAGCTGCTATATGAAACATGAAGTGCAAATCCTAACAGTCCTGCTAATACTTTTCTAGCATCCATCATTTCTTTGTTTTCA
GGGTCCAAACCTTGTCACTAGATGCAAAGACGCCTGTCTACTTCCTTTTTAAGTCTAGTATGGTTTTGTTCATTTCCATCACCTGTTTGGATGTGCTTTCCTGTTTTTTCTTTAAGGACTTCTACCTGTT
TGATTGTGTTTTCCTTTTTTCTTTAAGGACTTGTAACCTAAACACATCACAACCACAACTTCTCAGGTAACTATACTTGGGACTTAAAAAAACATAATCATAATCATTTTCCCTAAAACGATCAAGACTG
ATAACCATTTGACAAGAGCCATACAGACAAGCACCAGCTGGCACTCTTAGGTCTTCACATGTGGTCATCAGTTTGGGTTCCATTTGTAGATAAGAAACTGAGGTGC

Read #12 Alb-L1-chr17-Alb @9df79b4a-32fc-4a67-b4d7-d517944ee53e

CCGTCACAAGCAATGCAAGGCACGTACGTTTACTATGTCATTGCCTATGGCTATGAAGTGCAAATCCTAACAGTCCTGCTAATACTTTTCTAACATCCATCATTTCTTTGTTTTCCAGGGTCCAAACCTT
GTCACTAGATGCAAAGACGCCTTAGCTATACAGGTATGTGACAACATACCTGGCTTGTGTTCTTAAGAAAATAGTCCCAAAAAATCTCCTATAAACAATATAAAATTAAAACTGTCAGGCTCTGAGAGAT
GAAAATATTTCCTGTTCAGAAACCCATCTCAGGGAAAAAGACAGGGCCCTACCTCAGAGTGAAAGGCTGGGGAAAACAATTTTCCAAGCAAATGGACTGAAGAAACAAGCTGGAGTAGCCATTTTAATAT
GGATAAAATCGACTTCAACCCAAAGTTATCCAAAAAAGACAAGGAGGGACGCTTCATACTCCATCAAGGTAAAATCCTCCAAGAGAGGAACTCTCAATTCTGAATATCTACGCACCAAATGCAAGGGCAG
CCACATTCATTAGAGACACTTTAGTAAAACCAAAGCATACATTGCACCTCACACAATAATAGTGAGGAGGCTTCAACACACCACTTTCTTCAAAGGACAGATCGTGAAACAGAAACTAAACAGGGACACA
GTGAAACTAACAGAAGTTATGAACAAGAGCAGAAATCATAGGCATTTGGGCAAAACTTCTTCATGTGACTCACTTGTGCCATCAGGACCTGGTCAGGTTCAATCATGCATATACCACTTCCATTTTAAAA
GATTTATTTGTTTTGTGTATACGAGTATACTATAGCTGTCTTCAGACACATCAGAAATGGGCATCAGATCCATTGCAGATGGATTTGTGAGCCACCATGTGAATGCTGGGAATGGACTTAGGACCTCTGG
AAGAGCAGTCAGTGCTCTTAACTGCTGAGCTTTCTCTCCAGCTCTATACCACTTCCATTTGATAATGGATTGCTGCTGTGCATGTCCTACTTTATGATTTGTTGGTCCAATAATACTTAGCTCATGATGG
GCAGCTCTGGTCGCATGGTAACTAGCAGCTCATTGTCAAATGTTCAGTTTCCACTGAGGCCCAATAGCAGGCCAAGAGCTGTCTTTAAAGGGAGAATAGTTGTCTGCAGATGACGGTAGAGCCTTGTTCC
AAAATCTCAAAGGTCTTTTCTGTGATTCACCTATTGGTATGCCAAAGCTCCAAACAGCATTCCCCGTTCTGTCACTGACACCTCAAGTACCATTGGGTCTGCTGGATCATGTCATCCAAGTGGTAGAACA
GCCTGCACAGTAGCCTGGATCTGTTGAGGCCTTCTCCTGTCCCAGGCCCCACACAAAACTAACAGCTTTCCAAGTCATAGTATATGGCCTGGAGTAACAAACCCATGTGAGAGTTGTAATGCTTCCAGAA
GCCAAATAGACCCACTAAACATTATGCTTCTTTCTTGGTGGGGGGGGGTGGGGTCAACTGCAATTATTTATTCTTCACATACAAGGAATATCTCTGCATTCCCCACACCACTGGACTCCTAAGGGATTTC
TGCTGTTGTAGACAGTTCTTGAATTTTCCTATCTTCTGATTCTACATATCTGTTATTTACCAATGAGTAGCTACATCCAGTTCACTTGGTCCAGTCAGCATAATATCGTCAATATAATTGCACCAATGTG
ATATTTTGTGGAAGAGTCAGACAATCAAGTTCCTTCTAACTAAGTTATGACACAGAACAGGAGAGTCTATATATCCTTGAGGTAAAACTGTAAAGGTATCTAAACACATCACAGAAAAGCCACAACCTTC
TCAGGTAACTATGCAGGACTTAAAAAACATAATCATAATCATTTTCCTAAAACGATCAAGACTGATAACCATTTGACAAGAGCCATACAGACAAGCACCAGCTGGCACTCTTAGGTCTTCACGTATGGTC
ATCAGTTTGGGTTCCATTTGTAGATAAGAAAC

Read #13 Alb-L1-chr1-L1-Alb @d2d48a3f-5803-4c82-b0fc-7af9a078f5e7_R

CAACAACACTAGCAATGCAAGGCACGTACGTTTACTATGTCATTGCCTATGGCTATGAAGTGCAAATCCTAACAGTCCTGCTAATACTTTTCTAACATCCATCATTTCTTTGTTTTCAGGGTCCAAACCTTGTCACTAGATGCAAAGACGCC
TTAGATTCATTCTTATCTCCTTGTACAAAGCTCAAGACTAAGCAGATCAACGAACTCCACATAAAACAAGAGACACTGAAACTTATAGAGGAGAAAGTGGGAAAGAGTCTCGATGATATGGGCACAGAGGAAAAATTCCTGAACAGAACACT
AGTGGCTTGTGCTGTAAGAATTGACAAATGGGACCTCATAAAATTATAAAGCTTCAATAAAGAAAGGGACAGTGTCAATAAGACAAACTAGCAACCAACAGATTGGGAAAAATCTTTACCAATCCTAACCAATAGAGGGATAATATCCAATA
TATTCAAAGAACTCAAGAAGTTGGACTCCAGAAAACCAAATAACCCTATTTTTAAAATGGGATACAGAGCAAACAAAGAACTCTCAACTGAGGACTACCAAATGTGTGAGAAGCACCAAAAATGTTCAACATCCTTAGTCATCAGGGAAATG
CAATCAAAACAACCCTGAGATTCCACCTCACACCAGTCAGAATGGCTAAGATCAAAAACTCAGGTGACAGCAGACACTGGCAAGAATGTAGAGAGAGGAGTACTCCTCCATTGCTGGTGGGATTGCAAGCTGGTACAAACACTCTGGAAATC
TGTTTGGTGGTTGCTCAGAAAATTGGACATAGTACTATCAGAAGATCCAGCAATACCTCCTCCTGGGCATGTACTGAGAAGATGCTCCAACTGGTAATGACACGTGCCCACTATGTTCATAGCAGCCTTATTTATAAAAGCCAGGAGCTGGA
GAGAGAGCCCAGATGTCCCTCAACAGAGGGATGGATACAGAAAATGTGGCACATTTACACAATGGTGTACTACATAGCTATTAAAAACAATGAATTTATGAAGTTCTTAGACAAATGGATGGATCTGAGGATATCATCTCAAGTGAGGTAAC
TCAATCACAAAAGAACACTCATGATATGCATTCACTGATAAGTGGATATTAGCCCAGTAGCTCGGAATACCCAAGATACGATTTGCAAAATATATGAAATTCAAAAAGGAAGACCAAAAGTATGGATGCTTCTGATCCTTCATAGAAGGGGG
GAGCAAAATATCCATGGAAGGAGTTACAGAGACAAAGTTCAGAGCAGAGACTGAAGAAATGACCATCCAGAGATTGCCCCACCTGGGGATCCATCCCATAAACAACCACCAAAACCAGACACTATTGTAGACACCAACAAGAGCTTGCTGGC
AGGACCTGATATAGCTGTCTCCTGAGAGGCTCTGCCACTACCTGACAAATACAGAAGTGGATGCTCACAGCCATCCATTGGACAGGAGCTAGAGGAAGTATCCAAGGAGCTGAAGGGGTTAGTAGCCCCATAGAGGAACAAAAATATAAACT
AACCAGTACACTGAAGCTCCCTGGAAGTAAACTACCAATCAAAGAAACATATGGTAGGATTCATGTTCTAGCTGCATATGTAGCATAGGATGGCCTAGTCAGTCATTAATGGGAAGAGAGGCTCTTGGTCCTGTGATGGTTCTATGCCCCAG
TATAGAGAACTTCCAGGGCCAGGAATTGGGGAGTGGGTAGGTTGGGGAGCAGGGGATAGGGGGAAAGGGATAGGGGATTTTTGGTGAGGAAACTGGAAAGATAACATTTGAAATGTAAATAAAGAAAATATCTAATAAAAATGAAAAAAAAA
TGGGGTACAGAGCTAAACAAAGAATTCTCAATGGAGGAATATCAAATGCCCAAGAAGCACCTAAAGAAATGTTCAACATCCTTAGTTATCAGGGAAATGCAATCAAAACAACCCCTGAGATCCTACCTCACACCAGTCAGATGGATATGATC
AAAAACTCAGGTGACAGCAGATGCTGGCGAGGATGTGGAGAAAGGGGGAAGCTCCTCCATTTTTGGTGGGATTGTAAGCTAATACAACCACTTGGAAATCAGTTTGGTGGTTCTCAGAAAATTGGACATAGCACTACCAGAAGATCCAGCTA
TGATCCAGTTATACTACTCCTGGGCATATACCCAGAAGATGCTTCAACATGTAATAAGGACACATGCTCTACTATATTCATAGCAGCCTTATTTATAATAGCCAGAAGATGGAAAAGAACCCAGATGTCCCTCAACAGAGGAATGGATACAG
AAAATGTGGCACATCTACACAATAGAGTACTACTTCAGTTTTTTTAAATGATGAATTCATGAAATTCTTAGGCAAATGGAACTAGAAAAATATCATCCTGAGTGAGGTAACCCAATCACAGAAGAACACACATAGTATGCACTCACTGATA
AGTGGATACTATGGTTCAAAACCTCCAAATACCCAAGATACAATCTACAGACCACATGGAAGCCCTAAACACATCACAACCACAACCTTCTCAGGTAACTATACTTGGGACTTAAAAAACATAATCATAATCATTTTTCCTAAAAACGGAT
CAAGACTGATAACCATTTGACAAGAGCCATACAGACAAGCACCAGCTGGCACTCTTAGGTCTTCACGTATGGTCATCAGTTTGGGTTCCATTTGTAGATAAGAAAC

Read #14 Alb-chr5-ch1-chr2-L1-Alb @6cbc53de-41a9-4812-9844-df95ef5c762b

GCACTTCGACTAGACCAGCAATGCAAGGCACGTACGTTTACTATGTCATTGCCTATGGCTATGAAGTGCAAATCCTAACAGTCCTTGCTACTACTTTCTAACATCCATCATTTCTTTGTTTTCAGGGTCCAAACCTTGTCACTAGATGCAAA
GACGCCTTTAAAACACGTGGATCTATGTCACAGTGTTGTGTTACTATTGCACTGAAGATTTCTTATTTAGTACAAGTAGAGGTAATAATTAGAATATAGATAATTTCTGGACAACTACATATTTAGAACAGCGCAAAGTTAATTGCTATGCT
ATAAACATGCACCATTTAAATTCTGAGCTGGGACATCTGGCAAGGTACTTGACTTATAAGTTTATAAGGAGAGACTCTTGTTCACCTTGCCTCTAAGTTAGGTAAATGTATAATTAAATTTTGGCAAATATGACTTGGAGAAACCCAAAGCA
AAGGAATGTAATTAGTTTTATGAAAAAAAATTAAACTCTAGAAGTATTTTAGCAAGCATGTTGTATGTCCCAATAGGTCACCCATGTTTTTACACATAAATGCAATAAAAAATGAAAAAAAAAAACATTCATGCTTACTGCTTCCTAGCAAT
CAATAAAATGACTCTAGAGATTTTAAACAATATTCAAGTTAACAAAAGTATAGGTATAGGTTAAATTTGGCTAATGGAACATACTATGTAACATTTCTCTATGTAACTATACAGACATAATATAGTATCTTGCTTGGAGGCTCTTGCTTGAG
TACTGCATACATAATTGTGGCAGGTTGCTTTGCTCCAACTGACCGGCAGTTGAGCCTGCAAATAGAAGGAAGAGTGCTCCAAACAGGGGAAATGTGTATTGAGCAGATGCATAAAAGAAGGAAATTGGCTTTAGTGGGAGAAATGTCTAACA
TTCACTCTGGACTATGTTTGCAAACCTAGCTCTTCAAGTCAGTACCTAAACTCTGTTCTTATGCTTGTTCATCAAACCTTTTCCTGACTGAACTATCAACCTGGTCTCACTTGCCTCCTATTTTTAATGTTTCCTGAAAAAATAGGACACAG
GGCAGTTTAATCTTTTAGCCAAAAGTTGAAGCAACATTAAATTATTAAAAAAAATGTTCACTAATGAATGCAATGGTGAGCTTTCTTCACGACACGTACTTAAAGTTTGCATTCCATAGTCTCTACACCTTACTCTAAGGCAGAGTCCAATG
TCTGTACCACAGCTATGTGCCAAAGTTTTATACAGAATGATAATAACTGAGTATGAATGACTAATTTAAACAGCTGAAAACTCACAAAGAACCCAAATCATTGCAAGATTCTAGCACATAGGTTTTTCCTGTAAGAAAGAAAATTGAAAAGC
TATGCTTTGATATCCTAACACTATTTTTTAAATAAAATAGGCTTTAGATTTTAAATTACTGAATAATTTAAATACAATCTTCTGCACCTCTCCTTTCCCTCTACTGTTTTCTATATTTCCGATGTGGCCATAAAGGCCTCAGCTCATATGTC
TACTTCTTTATTTTGGGTTTTTAGACTCTAGATCCATGAACTATACATTTTTCTTTTATGAATTACTTACATTCTGGTTTTCGGGTTATAACAGCAGAAAATTGAGTAAGACTGCTATAATTAGGTTTTAATGTGTTGTGAACAGATACTAC
GACTAAGGCAAAGTCTTTATGAAACAATATTTAATTGGGGCTGGCTCACAGGTTCAGAGGTTCAGTCCATTATCATCAAGGTGGGAACATGGCAGTATCCAGGCAGGCAGAGCTGAGAGTTCTATATCTTCATCCAAAGGCTGCTAGGAGAA
GACTGACTTCCAGGCAACTAGGGGTGAGAGCATTAAGCCTCCACACCCACAGTGACACACCTACTCCAACCATGTCACACCTTATTCCAACAAGGCCACACTTCCAAGTGGTGCCACTCCTGGTCCAAGAATATCCAAACCATCACAAAGAC
AAACGTATTTTATGCCAGCATTCACTACATAGTATGTTTCTGAGGGACACATCTCTGAGTGCTCACAACATGTGGCAAAAGATGCTATATTAAAAATAGGTGTGACAGAGTCCATCTGATCAAGGGGATACAAATTGAAAGGAGGGAAGTCA
AAATATCACTTCTTTGCAGATGATATGATAGTATATATAAGGACCCTAAAAATTCCACCAGAGAACTCCTAACCTGATAAACAGCTTCGGTGAAGTAGCTGGACATAAAATTAACTCAAACAAGTCAATGGCCTTTTCTACACAAAGAATAA
ACAGGCTGAGAAAGAAATTAGGGAAACAACACCCTTCTCAATAGTCACAAATAATATAAAATATCTCTATGACTTAACTAAGGAAGTGAAAGATCTGTATGATAATAACTTCAAGTCTCTGAAGAAAAGAAATTAAAGAAGATCTCAGAAGA
TGGGACGATCTCCATGCTCATGGGATTGGCAGGATCAACATTGTAAAAAATGTCATCTTGCCAAAAGCAATCTACAGATTCAATGCACCCCCATCAAAATTCCAACTCAATTCTTCAAATGAATTAGAAGGAGCAATTTGCAAATTCATCTA
TGTTAGAATAACAAAAACCTAGGATAGCAAAAAAAAAAACTCTTCTCAAGGATAAAAGAACCTCTGGTGGAATCACCATGCCTGGGACCTAAAGCTTTACTACAGAGCAATTGTGGTAAAAACTGCATGGTACTGGTATAGAGACAGACAAG
TAGACCAATGGAATAGAATTGAAGACCCAGAAATGAACCTAATAATACCTATGGTCACTTGATCTTCGACAAGGAGCTAAAACCATCCAATGGAAGAAAGACAGCATTTTCAACAATTGGTGCTGGCACAACTGGTTGTTATCATGTAGAAG
AATGCGGATCGATCCATACTTATCTCCTTGTACTAAGGTCAAATCTAAATGGATCAAAGAACTTCACATAAAACCAGAGACACTGAAACTTGAGTGGCGGCGCGTGGGGGGAAAAGCCTGAAGATATGGGCACATCATGTCTCACAACTCTT
CTCAGGTAACTATACTTGGGACTTAAAAAACATAATCATAATCATTTTTCCTAAAACGATCAAGACTGATAACCATTTGACAAGAGCCATACAGACAAGCACCAGCTGGCACTCTTAGGTCTTCACGTATGGTCATCAGTTTGGGTTCCATT
TGTAGATAAGAAACTGAGGTGCCGC

Figure S13. (B) The representative read sequences display the insertion of LINE-1 and genomic DNA fragments at Alb.

Read #15 Alb-chr5-L1-chr5-L1-Alb @f4993ccf-89c0-4e58-85c8-03f07f708a2b_R

CAGCAATGCAAGGCACGTATTAACATTATGTCATTGCCTGCGCTATGAAGTGCAATCCTAACAGTCCCAAGTACTTTTCTAACATCCATCATTTCTTTGTTTTCAGGGTCCAAACCTTGTCACTAGATGCACAATCTCTTTCCACAATCCATT
CTACAAAGGATAATAGATGGAAAATACAACACAAGGAGGGAAACTACATCCTAGAAAAAGCAAGAAAGTAATCTTTCAACAAGCCCAAAAGAAGATAGCCACACAATCACACACCCATCTCTAACATGAAAAGTAACAGGAAGCAACACTCAG
TTTTCCTTAATATCTTCTTATCAGTGGACCCATTTCCTAGTAAAAAGACATAGACTAACAGACTGGATAAGTAAACCGTACCTAGAATTTTAGTTGCATACAGGAAACATACCTTAGTCAGCAAAGACAGATACTACCTCAGAGTAAAGTCTG
GAAAAAGTTATCCAAGCACATGGTACCAAGAAGCAAGCTGAGTTGCCATTCTAATATCAAATAAAATCAACTTTATAACCTTTGGTATCATCAGAACCCATTTCACCATAATAACAGAAAGGCAAGATAGATCTAAAATCACTTCATGATGAT
GACAGAGGACATTAAGAAGGACATAAATAACTCCCTTAGAAATACAGGAAACACAGATACTACCTCAGAGTAAAAGTGCTGGAAAAAAATTCAAGCAAATAGTCCCAAGAAACAAGCTGGAGTTGCTATTCTAATATTGAATAAATCAACTTG
TAACCTTTGGCATCACTGAACCAGTTCTCCCAGCATAACATGTCCATAACCAAAAGTTATAAAGAAAAGATAAGGAAGGACACTTCATACTCATCAAAGGAAAAAAATATACCAAGATGAACTCTCAATTCTGAACATCTATGCTCCAAATGC
ACAGGTACCATCACTCATTAAAGAAACTTTACTAAAGCTCAAAGCACACACTGTACCTCATACAGTAATAGTGGAAGATTTCAACACCCCACTCTCAGCAATGGACAGATCATGGAAATAGAAAATAAACAGACACACAGTGAAACTTTCAGA
AGTTATGAACCAAATGGATTTAACAGATACTTACAGAACATTTCATCCTAAAACAAAAGAATATACCTTCTTCTCAGCACCTCATGGTACCTTCTCCAAAACTGACCACATAATTGGTCACAAACAGGCCTCAACAGATACAAGAAGACTGAA
ATAATCCATGCATCCCATCAGATCACCACAGACTAAATCTTGTCTTCAATAATACAACAAAAACAACAGAAAGCCCACATACACATGGAAGCTGAACAATGCTCTACTTCCAATGATAACTTGGTCAAGGAAGAAAGGAAGAAAATTAAAGAA
TTTTTAGAACTTAATGAAAATGAAGGCATATCATACCCAAACAATGAAAGCAGTGCTAAGAGGAAACTCATAGCCTGAGTGCCTCCTAAATAGAAACTGAAGAGAGCATACACTACCAACTCGACAGCACACCTGGAAGCTCTAGAGCAAACA
GAAGAATACACCCAAAAAAGGAGTAGACGGCAGGAAATAATCAAACTCAGAGCTAAAATTAACTAAGTAAAAACAAAGAAAACTATACAAAGAACTTCAAACAAACTCAGGAGCTGGTTCTTTGAGAAAGTCAACAAGGTAGATAAACCTCTA
GCCGGACTAATCCAAAGGGTCCAGAGCCAGCATCCAAACAATAAAATCAGAATGAAAAGGGAGACATAACAACGGAAACTTATGAAATTAAAAATCCATCAGATCCTACTAAGAAAAGTCTATACTCAACAAAATTGGAAAATGTGTGTGTAA
TGGACAATTTTCTAGCCAGAGACCAGGTGCCAGTTAAATCAGGATCTGATAGATTATCTAACAGTCCCATAATCCCTAAAGAAATAGAAGAAGTCATTAAAAGTCTCCCCAGCCAGCTGTGCAGTAGTGGCACACACCTTTAGCTGCTGAAAA
TCATTTACCAAAATTCAACAGCCCTTCATGGTAAAAGTCTTGGATAGATCAGGAATTCAAGGTCCATACCTAAACATACTAAAAGCAAAATACAGCAAACTAATTGCCAACATCAAACTAAATGGAGAGAAACTTGAAGCAATCCCACTAAAA
TCAGGACTAGATAACAAGGCTGCCCACTCTCTCCCTATCTATTCAAATATAGTACTTGAAATTTTAGCTTTAGCAAGTAGAGACAACAAAAGGAGGCAAGGAGATATGAATCTGGAAAAAGAAGGAGTTAAAGTATCACTATTTGCAGAGAGG
ATATGATGGTACACTCTAAGTGACCCCAAACATTCTACCAGAGAACTCCTAAACCTGGATAAACAACTTCAGAGTGGTTGGATATACAATTAATCAAACTGCAGCCTTCCCATAATTGGGAAAAGGATAAACAGGCTGAGAAAGAAACTAGGA
AATGATACCTCTACAGTAGTCACAAATAACATTACATACCTTGGTGTGTGACAACCAAGCAACTGAAAGATTTGTATGAGAAGAACTTCAAGTCTCTGAAGAAACAAATCGAAGAAGATCTCAGAAGATGGAAAGAGATCGCCAATGCTCATG
GACTGGCAGGATTAATATAGTAAAAGGCCATCTTGTCAAAAGCAATCTATAGAGTCAATGCAATCCTATCAAAATTCCAACTCAATTTTCATAGACTTAGAGCAATTTGGCAAATTTATCTGGAATAACAAAGAATCCAGAAGATCAAAAACT
ATTTTCAGTGATAAAAGAACTTGGGAACCAGCATCCCTGACCTCAAGCTGTACTCAGAGCAATAGGGATAAAAACCAAAACAAAACAAAAAAAACAAAAACAACAAACAAAAAACCATATGGTATTGGTACAGAGACAGTAGGAAGATCAATG
GACTAGAATTAAAGACCCAGAATGAACCCACATGCTCATGGTCACTTGATCTTGATAGAGGAGCTAAAAACCATCCAGTGGAAAAAAGACAGCATTTTCAACAAATGGTGCTGGTTCAACTGGAGGTCAGCATGTAGAAGAATGCAAATTGAC
CTATTCTTGTTTCCTTGTACAAAGCTCAAGTCCAGAAGCTGATAAAGGAATCTAGACATAAACCAAATATACTGGAACTTATAGAGTGAGAAATTGGGGGAAAGTCTCAAGATATGGGCACTGTGGAAAATTTCCTGAAGAGGACATCAATGG
CTTATGTCTAAGATCAAGAATTGACAAATGGGACTCATAAAATTGCAAAGCTTTGATAAGGCAAAGAATACTGCTAATAGGACAAATGGCAACTAACAGATTGAGAAAAGATCTTTCCCAATCCTACATCTGATAGAGGGCTAATATCTCATA
TATACAAAGAACTCAAAATTTTAGATTCCAGAGAATCAAATAAATTCTATTAAAATAAAAGGTAGCAAGCTAAACAAAGAAATTTCAACTGAGGAATATCAAATGGCCAAGAACCACCTATAGAAATGTTCAACATCTTTAGTCATCAGGAAA
TGCAAATCAAAACAATCCTGTGATTCTACCTCACACTATTCAGAATGGCTAAGATCAAAAGCTCATGTGACAAAAGATACTGACAAGGATGTCCGGAAAGAGGAACACTCCATTGCTGGTGGACGCAAGCCGTGACAAACACTCTGAAATCAA
TCTGTCGTTCCTCAGAAAATTGGTTATAGTATTACCTGAGTGACCTAGCTATACCAATTCTGGGCATATGCTCCAAAATATCTCTCTAACATATAACAAGGACACATGCTACACTCATGTTCATAGCAGCCTTGTTTTTATAATAGCCAGGAC
CTGAAAGAACCTGTGATGTCCTTCATAGAGGAATGACAGTAAATGGTAAATTATACACTGAGAACTTCTCAGCTATTAAAAAAACAATGAATTCATGAAATTTTAGGTAAAATGGATCGAACTAGAAAATATCATCCTGAGGAGGTAACCCAA
TCACAAAAAGAATACACATGGTATGCATTCACTGATAAGGAGGTTAGCCCATATGCTCAGAATAAATTCAAGATACAATTGACAGACCACATGAAGCTTATGAAGAAGGAAGACCAAAGTATGGATACTTCTGTCTTTCTTAGAAAGGGGGAA
TAAAATACTTGTGGAACAAATACAGAGACAAAGTGTGGATAGAGACAGAAGGAAAGACCATCCAGAGACTGCCCCACCTGGAGATCCATACCATATACACAGTCACCAAACACAGACACTATTATTGATGTCAAGAAGTACTTGCTGAAAGCA
GCCTGATAAGAAATGCCTCGAGAGGTTTCAGCAGAGCCTGACAATACAGAGGTGGATACTCTAAGCCAAATCCATTGGTCTGACCATAGGGTTCACAATGGAGGAGTTAGAGAAATAACTGAAGGAGCTGAAGGGCTTTTGCAACCATAGGAA
GGGACAACACAATAATCCAGCAGATTTCCCACCCCCACCCCAGAGCTCACAGGTGACTAAGCCACCAACCAAGTGAGTACACATAGAGGGACCTATAATTCCAGATGCACAAGTAGCAGAGGATGGCTTTGTCACGCATTAATGCAAGAAGAG
CCCCTTGGTCCTATGAACTTCGATGGATGTCCCAGTGTAGGGTAATGCCAGGGTGGGGAGGTGGGAATGGATGGGTGGGTGGTGGAACACCTCATAGAAGCAGCGGAAGAGGAGATGGTTTAAGGAGTTTCTTGGGGTGGTACGAAAGGGAAT
AACATTTGAAGTATAAATAAAGAAAAATAAATATACAATTAAAAAAAGAAACTGACTTTATTTGAGTGTTTCAAAGTGCTCTGTGATTCTCCTGACAGCTTATCTTAAAAAAATTTTATTAGATATTTTCTTCATTTATATCAATGCTATCCC
AAAGTCCCCTATTACCCCTCCTGCTCCCTACCCACCCACTCCCACCTCTTGGCCCTGGTGTTCCTCTGTACTGGGGCATATACAGTTTGCAAGACTAAGGGGCCTCTTCCCAATGATGGCCGACTAGGCCATCTTCTGCTACATATTCAGCTA
GAGACACGAGCTCTGGGGTACTGGTTAGATCATGCTGTTGTTCCACCTATAGGGCTGCAGACCCTTTCCAGCTCCCTTGGGTATTTTCTCTAGCTTCCCATTGGGGACTCTATGTTTCATCCAATAGATGACTGTGAGACCCATTTCTGTATT
TGCCAGGCACTGGCAAAGCCCTCACAGGAGACAGCTATATCAGGGTCTTTCAGCAAAATCTTGCTGGCGTAAGTAACAGTGTCAGTCAGAATGGCTAGGATCAAAATTCAGGTGACAGCAGATGTTATGAGATGTGGAGAAAGAGGAACACCT
CCATTGTTGGTGGGACAAGGTAAGCTGGTACAACCACTTGAAATCAGTCTGGCGGTTCCTCAGAAAACTGGACATAGTACTATCGCTAGATCCGGCACAATTTCCTGGGCAACACCCAGAAGATATTCCAACTGGTAATAAGGACACATGCTC
CACTATGTTCATAGCAGCCCCATTTATAATAGCCAGAAGCTGGAAAGAATCCAGATGTCTTTCAACAGAGGAATGATACAAAATGTGGTACATTTACACAATGGAGTACTACTCAGCAATTAAAAAACAATGAATTTATGAAATTCTTTGGCA
AATGGATGTATCTGGAGTGATATTATCCTGAGGAGGTAACCCAATCACAAAAGAAGTCACTTGCTATGCACTCACTGATAAGTGGATATTAGCCCAGAAACCTAGAATACCCATGATACAACTTCCAACACACACAAAAAAATTCAAGAAGGA
AGACCAATTTGATTACTTCATTCCTCCCTAGAATAGGAACAAAATATCCATGGAAGGAGTTGCAGAGACAAAGTTTGACCTAAGATGAAAGGATAGACCATCCAGAGACTGCTCCACCCAGGGGGTCCATCCCATAATCAGGCCAATTTAAAC
GCAGACACTATTGCCTACAGCTTATCTAAACACATCACAACCACAACCTCTCAGGTAACTATACTTGGGACTTAAAAACATAATCATAATCATTTTTCCTAAAACGATCAAGACTGATAACCATTTGACAAGAGCCATACAGACAAGCACCA
GCTGGCACTCTAGGTCTTCATGTATGGTCACTGCCTGGGTTCCATTTGTAGAT

Figure S13. (B) The representative read sequences display the insertion of LINE-1 and genomic DNA fragments at Alb (continued).

Read #16 Alb-L1-BB-Alb @1716251b-f892-4f78-a6d6-bac4f37df9f3_R

GCACCTGATATGCGTTAGTAATGCAAGGCACGTACGTTTACTTGCGTCATTGCCTAAGTGGCTATGAAGTGCAAATCCTAACAGGTCCTGCTAATACTTTTCTAACATCCATCATTTCTTTGTTTTCAGGGTCCAAACCTTGTCACTAGATGC
AAAGACGCCTTAGCCACCAGAGGTTCTTTTATCCTTGAGAAGAGCTATCCTCGGTTTTTTTGTTATTCCAGATGAATTTGCAAATTGCTCCTTCTAATTCGTTGAAGAATTGAGTTGGAATTTTAATGGGGATTGCATTGAATCTGTAGATTG
CTTCGGGAAATGTGCGCGGAACCCCTATTTGTTTATTTTTCTAAATACATTCAAATATGTATCCGCTCATGAGACAATAACCCTGATAAATGCTTCATTAATATTGGAAAAGGAAGAGTATGAGTATTCAACATTTCCGTGTCGCCCTTATTC
CCTTTTTTGCGGCATTTTGCCTTCCTGTTTCTTGCTCACCCAGAAACGCTGGTGAAAGTAAAGATGCTGAAGATCAGTTGGGTGCATCGAGTGGGTTACATCGAACTGGATCTCAACAGCGGTAAGATCCTTGAGAGTTTTCGCCCCGAAG
AACGTTTCAAATGATGAGCACTTTTAAAGTTCTGCTATGTGGCGCGGTATTATCCCGTATTGACGCCGGGCAAGAGCAACTCGGTCGCCGTATTGATCGAGTTCAGAATGACTTGCTGAGTACTCACCAGTCACAGAAAAGCACTACGGATGG
CATGACAGTAAGAGAATTATGCAGTGCTGCCATAACCATGAGTGATAACACTGCGGCCAACTTACTTCTGACAACGATCGGAGGACCGAAGGAGCTAACCGCTTTTTTGCACAACATGGGGATCATGTCCTGCCTTGATCGTTGGGAACCTGA
GCTGAATGAAGCCATACCAAACGACGAGCGTGACACCACGATGCCTGTAGCAATGTGGCAACAACGTTGCGCAAACTATTAACTGGCGAACTACTTACTCTAGCTTCCCGGCAACAATTAATAGACTGGATGGAGGCGGATAGTTGCAGGAC
CACTTCTGCGCTCGGTCCTTCCGGCTGGCTGGTTTATTGCTGATAAATCTGGAGCCGGTGAGCGTGGGTCCTGCGGTATCATTGCAGCACTGGGGCCAGATGGTAAGCCCTCCTGCACGTAGTTATCTACACGACGGGAGTCAGGCAACTATG
GATGAACGAAATAGACAGATCGCTGAGATAGGCGCCCTACTGATTAAGCATTGCAACTGTCAAACCAAGTTTACTCAGCGTTATACTTTAGATTGATTTAAAACTCATTTTTAATTTAAAAGGATCTAGGTGAAGATCCTTTTGATAATCTCA
GCGACCAAAATCCCTTAACGTGAGTTTTCGTTCCACTGAGCGTCAGACCCCTTGTAGAAAAGATCAAAGGATCTTCTTGAGATCCTTTTTTCTGCGCGTAATCTGCTGCTTGCAAACAAAAAACCACCGCTACCAGCGGTGGTTTGTTTGCCG
GATCAAGAGAGCTACCAACTCTTCCGAAGGTAACTGGCTTCAGCAGAAAGTAGATACCAAATACTGTTCTTCTAGTGTAGCCGTAGTTAGGCCACCACTTCAAGAACTCTGTAGCACCGCCTACATACCTGGCTCTACAACCCTGTTACCAGT
GGCTGCCAGTGGCGATAAGTCGTGGCCCTACCGGGGCTGGACCTCAAGACGATAGTTACCGGATAAGGCGCAGCGGTCGGGCTGGAACGGGGGTTCGTGCACACAGCCCAGCTTGGAGCGAACGACCTACACCGAACTGGAGATACCCACAGC
GTGAGCTATGAGAAAGCGCCACGCTTCCCGAAGGGGAGAAAGGCGGACAGGTATCCGGTAAGCGGCAGGGTCGGAACAGGAGAGCGCACGAGGAGCTTCCAGGGGGAAACGTCTGGTATCTTTATAGTCCTGTCGGGTTTCGCCACCTCTGAC
TTGAGCGTCGATTTTTGTGATGCTCGTCAGGGGCGGCGCCTATGGAAAACGCCAGCAACGCGGCCTTTTTACGGTTCCTGGCCTTTGCTGGCCTTTGCTCACATGTTCTTTTCCTGCGTTATCCCCTGATTCTGTGGATAACCGTATTACCG
CCTTTGAGTGAGCTGGATACCGCTCGCTGCAGCCGAACGACCGAGCAGCGCAGCTCAGTGAGCGAGGAAGCGAAGAGCGCCCAATACGCAAACCGCCTCTCCCTCCCGCGCGTTCGGCCGATTCATTAATGCAGCTGGCACGACAGGTTTCCT
GACTGGAAAGCCGGCAGTGAGCGCAACGCACCAAGGTGAGTTAGCTCACCACAGGCACCCCAGGCTTTACACTTTATGCTTCGGTTTCCGTATGTTGTGTGGAATTGTGAGCGGATAACAATTTCATTACCAGGTGGTGCAGATGCCTAAACA
CATCACAACCACAACCTTCTCAGGTAACTATACTTGGGACTTAAAAAACATAATCATAATCATTTTCCTAAAACGATCAAGACTGATAACCATTTGACAAGAGCCATACAGACAAGCACCAGCTGGCACTCTTAGGTCTTCACGTATGGTCAT
CAGTTTGCCCATTTGTAGATAAGAAACTGAGGTGC

Read #17 Alb-BB-L1-Alb @f17a093d-ed82-46f9-95e2-ffbfd705893a

GTTTCTTATCTACAAATGGAACCCAAGGCGGTGACCATACGTGAAGACCTAAAATTTTAGCTGGTGCTTTATCTGTATGGCTCTTGTCAAATGGTTATCAGTCTTGATCGTTTTAGGAAAAATGATTATGATTATGTTTTTTAAGTCCCAAGT
ATAGTTACCTGAGAAGGTTGTGGTTGTGATGTGTGTTCATCTGCACCACCTGTGTGAAATTGTTATCCGCTCACAATTCCACACAACATACGAACCGGAAGCATAAAGTGTAAAGCCTGGGGTGCTAATGGGTAGGCTAACTCACCTTAATTT
GCGTTGCGCTCACTGCCCGCTTTCCAGTCAGGGAAACCTGTCGTGCCAGCTGCATTAATGAATCGGCCAACGCGCGGGAGAGGCGGTTTGCGTATTGAGCGCTCTTCCGCTTCCTCGCTCACTGACTCGCTGCGCTCGGTCGTTCGGCTGCAG
CGACGGCCATCGGCTCCTCAAAGCGGTAATACGGTTATCCACAGAATCAGGGGATAACGCAGGAAAGAACATGTGAGCAAAGGCCAGCAAAAAGGCCAGGAACCGTAAAAGGCCGCGTGCTGGCGTTTTTCCATAGGCTCCGCCCCCCTGA
CGAGCATCACCAAAAATCGACGCTCCAAGTCAGAATTGGCGAAACCCGACAGGACTATAAAGATACCAGGCGTTTCCCCTGCAGAGCTCCCTCGTGCGCTCTCCTGTTCCGACCCTGCCGCTTACCGGATACCTGTCCGCCTTTCTCCCCTTC
GGGAAGCGTGGCGCTTTCTCATAGCTCACGCTGTAGGTATCTCAGTTCGGTGTAGGTCGTTCGCTCCAAGCTGGGCTGTGTGCACGAACCCCCCACGTTCAGCCCGGCCGCTGCGCCTTATCCGGTAACTATCGTCTGCCCAGAGTCAACCCGG
TAAGACACGACTTATCGCCACTGTAGCAACCACTGGTAACAGGATTAGCAGAGCGAGGTATGTAGGCGGTGCTACAGAGTTCTTGAAGTGGTGGCCTAACTACGGCTGCACTTTGAAGAACAGTATTTGGTATCTGCGCTCTGCTGAAGCCAG
TTACCTTCCCCGGAAAAAGAGTTGGTAGCTCTTGATCCGGCAAACAAACCACCGCTGGTAGCAATTGGTTTTTGTTTTGCAAGCAGCAGATTACGCGCAGAAAAAAGGATCTCAAGAAGATCGCTTTGATCTTTTCTACGGGGTCTGACGCTC
AGTGTTGGAACGAGAAACTCACGTTAAGGGATTTTGGTCATGAGATTATCAAAAGGATCTTCACCTAGATCCTTTTAAATTAAAAATGAGTTTTAGAAAATCAATCTGATATATATGAAGTAAGCTTGGTCTGACAGTTACCAATGCTTAATC
AGTAGGCACCTATCTCAGCGATCTGTCTGTTTCGTTCATCCTGGTTGCCTGACTCCCCGTCGTGTAGATAACTACGATACGGGAGGGCTTACCATCTGGCCCCAGTGCTGCAATGATACCGCAGAGACCCACGCTCACCGGCTCCAGATTTAT
CAGCAATAAACCAGCCAGCCGGAAGGGCCGGAAAAGCGCAAGTGGTCCTGCAACTTTATCCGCCTCCATCCAGTCTATTGGTATTTGCCGGGAAGCTAGAGTAAGTAGTTCGCCAGTTAATAGTTTGCGCAACGTTGTTGCCATTGCTACAGG
CATCGTGGTGTCACGCTCGTCGTTTGGTATGGCTTCATTCAGCTCCGGTTCCCAACGATCAAGGCGAGTTACATGATCCCCCATGTTGTGCAAAAAAGCGGTTAGCTCCTTCGGTCCTCGATCGTTGTCAGAAGTAAGTTGGCCGCTAGTGTT
TATCACTCATAGTTATGGCAGCACTGCATAATTCTCTTACTGTCATGCCATCCGTAAGATGCTTTTCTGTGACTGGTGAGTGCTCAACCAAGTCATTCTGAGAATAGTGTATGCGGCATTCCGGTTGCTCTTGCCGGCGCGTCAATACGGGA
TAATACCGCGCCACATAGCAGAACTTTAAAGTGCTCATCATTGGAAAACGTTCTTCGGGGCGAAAACTCTCAAGGATCTTACCGCTGTTGATCCAGTTCGATGTAACCCACTCGTGCACCCAACTGATCTTCAGCATCTTTTACTTTCACCCA
ACGTTCTGGGTGAGCAAAAACAGGAAGGCAAATGCCGCAAAAAAGGGAATAAGGGCGACACGGAAATGTTGAATACTCATACTCTTCCTTTTCAATATTATTGACATTTATCAGTTATTGTCTCATGAGCGGATACATATTTAGATATCATT
AGAAAAATAAACAAATAGGGGTTCGCATTTTCCCCGAAAAGTGCCACCTGGTGGTGCAGATTACTGGTTAGTTTATATTGTTCCTCCTCCTAGGGGATTGCATACACCTTCAGCTCCTGGGTACTTTCTCTAGCTCCTTCATTGGGGACCTA
TGCTCCATCTAATGGATGACTGTGAGCATCCATTTCTGTATTAGTCAGGAACTGTCAGAACCTCTCAGAGGTAGCTATGTTAGGCTCCTGCCAACAAGCTAATGTTGGCATCCACAATAGTGTCTGGGTTTGGTTAAAATTTTTTTTTTTGGG
ATGGTCCCCAGGTAGGGCAGTTTCTGGATGGTTATTTTCCTTCAGTCTCTGCTCTGAACTTTGTCTCTGTAACTTCTTCCATGGGTATTTTGTTCCCCCTTCTATGAAGGGTCAAAGTGTCCACTTTGGTCTTCCTTTCTCTTGAGTTTCATG
TGTTTTGTAAAATGTATCACAGGGTATTTTGAACTTCTGGGCTAATATCCACTTATCAGTGAGTGCATATCATGTGTGTTCTTTTTGTGACTGGGTTACCCTACTCATAATGATATTTTATAGTTCCATCCATTTGTCTAAAATTTCATGAAC
TCATTGCTTTTAATAGCTCCGTTGTGTAAATGTACCACATTTTCTGTATCCATTTCTCTGTTGTTGAGGGACATCTGGTTGCTTTCCAGCTTCCTGCTCTTATAAATAAGGCTGCTATGAACATAGTGGAGTATGTGTACTTAGCCACAAGTT
GGAGCATCTTCTGGGTAAATGTCCAGAGTGGTATCCTTTGGACCTTCAGTAGAACTATGAACAATTTTCTGAGGAAATACCAAACTGATTTCCACAGTGGTTGTCCCAGCTTACAATCCACTAGCAATGGACGAGTGTTCCTCTTTTCTCCAC
ATCCTTACCAACATCTGCTGTCACTGAATTTTTGATGTTAGCCATTCTGACTGGTGTGAGGTGAATCTGGGGTTGTTTTTTGATTTCATTTCCCTGAAGATTAAGGATGTTGAACATTTTTGTTAGGTGCTTCTCAATAGTCAATTATTCATA
AGAGAATTCTTTGTTTAGCTCTGTACCCCATTTTTAATAGTGTTACTTGGTTTTCTACACCAACTTCTTGACTTCTTTATATATCTTGGATGTGTAGCCCCCTATCTGAGATGGGATTGGTAAAGATCTTTCCCCAATCTGTTGGTTGCAGTT
TTGTCTTTGACAATGTCCTTTGCTGCCACAGATGCTTTGCAATTTTATGAGGTCCCATTTGTCCATTCTCAATCTTACAGCACACAAGGAAATTTTTCCCCTTCACTCACATCTTTGAGGTCTTCCCCACTTTTCCTCTATAAGTTTCAGTGT
CTCTTATTTTATGTGCAGTTCCTTAATCCACTTAGACTTGAGCTTTGTACAAGGGGAGATGAATGGATCAGCTTGCATTCTTCTACATGATAACTGTCAGCACCATTTGTTGAAAATGCTGTCTTTTTTATACACTGGATGGTTTTAGTTCCT
TTGTCAACCATCAAATGACCACTCGTGTGGGGATCATCTAAGGCGTCTTTGCATCTAGTGACAAGGTTTGGACCCTGAAAACAAAGAAATGATGGATGTTAGAAAAGTATTAGCAGGACTGTAGGTTGCACTTCATGGCCATAGGCAATGACA
TAGTAAACGTACGTGCCTTGCATT

Read #18 Alb-BB-L1-Alb @3e9c16a4-5357-40ab-a0e4-94b9550244a9

ACTCCTGAAGCAATGCAAGGCACGTACGTTTACTATGTCATTGCCTATGGCTATGAGTGCAAATCCTAACAGTCCTGCTAATACTTTTCTAACATCCATCATTTCTTTGTTTTCCAGGGTCCAAACCTTGTCACTAGATGCAAGACGCCTTGG
ATTCATCTGCCACCAGGTGGCACTTTTCGGGAAACTGTGCGCGGAACCCCTATTTGTTTATTTTTCTAAATACATTCAAATATGTATCCGCTCATGAGACAATAACCTGATAAATGCTTCAATAATATTGAAAAAGGAAAGTATGAGTATTCA
ACATTTCCGTGTCGCCCTTATTCCCTTTTTTGCGGCATTTTGCCTTCCTGTTTACTCACCCAGAAACGCTGGTGAAAACAAAAGACGAAGATCAGTTGGGTGCACGAGTAGATTTTATCGAACTGGATCTCAACAGCGGTAAGATCCTTGAGA
GTTTTCGCCCCGAAGAACGTTTTCCAATGATGAGCACTTTTAAAGTTCTGCTATGTGGCGCGGTATTATCCGTATTGACGCCGGGCAAAGGCAACTCGGTCGCCGCATACACTATTCTCAGAATGACTTGGTTGGTACTCACCCAGTCACAGA
GAAGAAAGCATCTTACGGATGGCATGACAGTAAGAGAATTATGCAGTGCTGCCATAACCATGAGTGATAACACTGCGGCCAACTTACTTCTGACAACGATCGGAGGACCGAAGGAGCTAACCGCTTTTTTGCACAACATGGGGGATCATGCCA
ACTCGCCTTGATCGTTGGGAACCGGAGCTGAATGAAGCCATACCAAACGACGAGCGTGACACCACGATGCCTGTAGCAATGGCAACAACGTTGCGCAAATATTAACTGGCGAACTTCACTTACTCTAGCTTCCCGGCAACAATTAATAGACTG
GATGGAGGCGGATAAAGTTGCAGGACCACTTCTGCGCTCGGCCCTTCCGGCTGGCTGGTTTATTGCTGATAAATCTGGAGCCGGTGAGCGTGGGTCTCGCGGTATCATTGCAGCACTGGGGCCAGATGGTAAAGCCCTCCCGTATCGTAGTTA
TCTACACGACGGGGAATCCGAGCAACTATGGATGAACGAAAAATAGACAGATCGCTGAGATAGGTGCCTCACTGATTAAGCATTGGTAACTGTCAGACCAAGTTTACTCATATATACTTTAGATTGATTTAAAACTTCATTTTTAATTTAAAA
GGATCTAGGTGAAGATCGCTTTTTGATAATCTCATGACCAAAATCCCTTAACGCGTGAGTTTTCGTTCCACTGGGCGTCAGACCCCGTAAAGAAAAAGATCAAAAGGATCTTCTCCCGAGATCCTTTTTTTCTATGCGCGTAATCTCTGCTGC
TTGCAAACAAAAAAACCACCGCTACCAGCGGTGGTTTGTTTACCAGATCCAAAAACTACCAACTCTTTTTTCCGAAGGTAACGCTGGCTTCAGCAGAGCGCAGATACCAAATACTGTTCTTCTAGTGTAGCCGTAGTTAGGCCACCGCTTCAA
GAACTCTGTAGCACCGCCTACATACCTCGCTCTGCTAATCCTGTTACCAGTGGCTGCTGCCAGTGGCGATAAGTCGTGTCTTACCGGGTTGGACTCAAGACGATAGTTACCGGATAAGGCGCAGCGGTCGGGCTGAACGGGGGTTCGTGCACA
CAGCCCAGCTTGGAGCGAACGACCTACACCGAACTGAATACCACAGCGTGAGCTATAGAAGCGCCACGCTTCCCGAAGGGAGGAGGAGAGAGAGAGAGAAAAGGCGGACGCGTTATCCGGTAAGCGGCAGGGTCGGAACAGGAGAGCGCACGAGG
GAACTTCCAGGGGGAAACGCCTGGTATCTTTATAGTCCTGTCGGGTTTCGCCACCTCCTGACTTGAGCGTCGATTTTTGTGATGCTCGTCAGGGGCGGAGCCTATGGAAAAACGCCAGCAACGCGGCCTTTTTACAGTTCCTGGCCTTTTGCT
GGCCTTTTGCTCACATGTTCTTTCCTGCGTTATCCCCTGATTCTGTGGATAACCGTATTACCGCCTTTGAGTGAGCTGATACCCGCTCGCCGCAGCCGAACGACCGAGCGCGGCGAGTCAGTGAGCCGAGGAAGCGGAGGGAAAAGAGCATAA
TACGCAAACCGCCTCTCCCCGCGCGTTGGCCGATTCATTAATGCAGCTGGCACGACAGGTTTCCCGGCTGGAAAGCGGGCAGTGAGCGCAACGCCGTTAAGGTGAGTTAGCTCACTCATTAGGCACCCCAGGCTTTACACTTTATGCTTCCGG
CTCGTATGTTGTGTGGAATTGTGAGCGGATAACAATTTCACACTAAAGGGAGCTAAGGGACAAAATCCTAATTAACAAAAATCGAAATATGAAAAGGAACATAACAACAGATCCTGAAGAAATCCAAAACACCATCAGATCCTTCTACAAAAG
GCTATACTCAACAAAACTCCTAAACACATCACACAACCACAACCTTCTCAGGTAACTTATACTTGGGACTTAAAAACATAATCATAATCATTTTTCCTAAAACGATCAAGACTGATAACCATTTGACAAGAGCCATACAGACAAGCACCAGCT
GGCACTCTTAGGTCTTCACGTATGGTCATCAGTTTGGGTTCCATTTGTAGATAAAC

Figure S13. (C) The representative read sequences illustrate the insertion of both the plasmid backbone and LINE-1 fragment at Alb.

Read #19 Alb-Unknown sequence-L1-BB-Alb @7e188ad4-dbbd-4017-aadd-9336b25e1012

GATTGCCTCAGTAGAAGGCACGTATACGTTTACTATGTCATTGCCTGTAAACATAGAAGTGCAAATCCTAACAGTCCTGCTAATACTTTTCTAACATCCATCATTTTCTTTGTTTCGGGGTCAAACCTTGTCACTAGATGCAAAATTCAGCA
AATGGGTGCCAATGACCCAGTTATCCGTATGTGAAGAATGCAATGATCCGGCACGCCAGATGATCCCCCACAGGCGATTCATTTGATGGTGACAAGATTCATGACATCCGGTGTATAAAAGACAGCATTAGAACATGCTTCCTTGCAAAGCT
CAAGTCTACAAGTGGATTAAGGAGCTGCATAAAATAAGAGACACTGGAAACTTATGGGAAAGTGGGGAAGACCATAAAGTGAGGAAGGGAAAATTCCTTGTGCTGTAAGATTGAGAATGGACAAATGGACCTGTAAATTGCCACAAAGCAT
CTACCATTGTGAAGGCAAAGGACATTGTCAAAGACAAAACTGCAACCAACAGATTTGGGAGAGAGATCTTTACCAATCCTATCTCCAGATAGGGGCTAATATCCGAGATATATATAGAAGTCAAGAAGTTATGGTGTGAAAACTAAGTAACA
CTATTAAAAATGGGGTACAGGAGCTAAACTCTGAGAATTCTCAAGGAATACCGACTGGCTTGACTTCTAACAAAATGTTCCAACATCCTTAATCTTAGAGGGAAGTAAATCAAAACAGCCTAAGATTCCACCTCACCAGTCAGAATGGCTAA
CATCAAAAATTCAGGTGACAGCAGATGCTGGTAGGGATGTGGAGAAGAGGAACACTCGTCCATTGCTAGTGGGATTGTAAGCTGGGACCACTGGCAAATCTGATTTGGCATTTCCTCAGAGAAGGTGTTCATAGTTCTACTGGAAGGTCCAG
CGATACCACTCCTGGACATTTGCCCCAGAAAGATGCTCCAACTTTATGTCTTGCATCCTCCCACTGTTCCTGTAGCCTTATTTATAAGAGCAAGGCTGGAAAGCAACCAGATGTCCCTCAACAGAAATGGATACAGAAAATGTGGTACATTT
TACACAACGGAGCTAATAAAAGCAATGAGTTCATGAAATTCTTAGACAAATGGATGGAACTATAAAATATCATTATGATGAGGTAACCCAGTCACAAAAGAACTTTTTTTATGATGCACTCACTGATAAGTGGATATTAGCCCAAGTTCAAA
ATACCCCAGATACATTTTACAAACATGAAACTCAAGAGGAAGGGCCAAAGTGGACACTTTGACCCTTCATAGAAGGGAACAAAATACCATGGAAGGATTACAGAGACAAAGTTCCAGAGCAGAGACTGGAAGGAATAACCATCAGAAACTGC
CTACCTGAGATCCATCCCAAAAAACAACCAAACCCAGACACTATTGTGGATGCCAGCAGTAGCTTGTTGGCGGGGAGCCTAACACGCTGTCTCCTGAGAGGTTCTGACAGTTCTGACTAATACCCAGAAATGGATGCTCACAGTCATCCATT
AGATGGAGCATAAGTTCCACAATGGAAGGAGCTGGAGAGAAATTGCCCAGGGGAGCTGGAAGGTGTATGCAATCCCCTAGGAGGAGGAACAATACTCAGCAAGCTAACCAGTAATCCCTGCCACCAGGTGGCACTTTTCGGGAAATGTGCAC
TTGGAACCCCTATTTGTTTATTTTTCTAAATACATTCAAATATGTATCCGCTCATGAGACAATAACCCTGATAAATGCTTCAATAATATTGAAAAGGAAATATGAAATATTCAACATTTCGTGTCGTAGCACTTATTCCTTTTTTGGCATTT
TTACCTTCCTGTTTTTGCTCACCCAGAAACGCTGGTGAGAAAGTAAAAGATGCTGAAGATCAGTTGGGTGCCTGGTGGTTACATCGAACTGGATCTCGCCAGCGGTAGAATCCTTGAGTTTTCGCCCGAAGAACGTTTTCCAATGATGAGCA
CTTTTTAAAGTTCTGCTATCATGGCGCGGTATTATCCCGTATTGACGCCGGGCAAGAGCAACTCGGTCGCCGCATACACTATTCTCAGAATGACTTGGTTGAGTACTCACCAGTCACGAAGAAAAAGCATCTTACGGATGGCATGACAGTAA
GAGAATTATACGGTACTGCCATAACCATGAGTGATAACACTGCGGCCAACTTCACTTCTGATGACAACGATCGGAGGACGAAGGAGCTAACCGCTTTTTTACTGCTTATGGGGGATCATGTAACTCCTTGTGATGTTTGAGGAACCGGAGCT
GAATGAAGCCATACCAAACGACGAGCGTGACACCACGATGCCTGCTAACCATGGCAACAACGTTTAGCACAAACTATTAACTGGCGAACTACGCCTCCTGGCCCCGGCAACAGTGTAGACTGGATGGAGGCGGATAAAGTTGCAGGACCACT
TATGCCTCGGCCTTTCGGCTGGCTACGTGGTTTATTGCTGATAAATCTGGAGCCGGTGAGCGTAGGTCTCGCGGTATCATTGCAGCACTGGGGCCCGAGTAGGTAAAGGCCCTCCCATCGTGATTATCTACACGACAGGAGTCGAGCAACCT
GGATGGTAGAAGGCAGACCCAGATCGCTGAGATAGGTGCCTCACTTTGATTCAGCATTGGTAACTGTCAGACCAAGTTTACTCATATATACTTTAGATTGATTTAAAACTTCTGTTTTAATTTAAAAGGATCTGGGTGAAGATCCTTTTTGA
TAATCATGACCAAAAATCCCTTAACGTGAGTTTTCGTTCCACTGAGCGTCGGACCCACGCCAAAGAAAGATCAAAGGAATCTTCTTGAGATCCTTTTTTTCTACTGCGCGTAATCTGCTGCTTGCAAACAAAAAAACCACCGCTACAGCGGT
GAATGGTTTGTTTGCCGGATCAAACTACCAACAACAGGGTAACTGGCTTCAGCGGTACCAGATACCAAAATACTAACTGTTCTAGTGTAGCCGTAGTTAGGCCACCACTTCAAGAACTCTATACTTCCTACATACCTCGCTCTGCTAATCCT
GTTACCAGTGAAAAACGCTGCCAGTGGCGATAAGTCGTGTCTTGCCGGGTTGGACTCAAGACGATAGTTACCGGATAAGGCGCAGCGGTCGGGCTGAACAGGGGAGTTCGTCGCACACAGCCCAGCTTGGAGCGAGGCGACTACCGAGCAGA
TACCTGCAGGCGCTGAGCTATGAAAGCGCCACGCTTCCCGAAGGGAGAAAGGCGGACAGGTATCCGGTAAGCGGCAGAGTCCAGACAGGAGAGCGCACGGGGGCTTTCAGGAAACGCCTGGTATCTTTATAGTCCTGTCGGGTTCGCCTCGC
GGCAGGCGTCGATTTTTGCCGGTGCTCGATGGGGGCGAACCATGGAAAAACGCCAGCAACGCGGCCTTTTACAGTTCCTGGCCTTTTTGCCCGTGTGCCTTCACATGTTCTTTCCTGCGTTACATCCCCTGATTCTGTGGATAACCGTATTA
CCGCCTTTGGTGGCAGTACCGCTCGCCGCAGCCGGGTGACCGGGCGCAGCAGGTCAGTGAAACGAGGAAGCGGAAGAGCGCCCAATACGCAAACCGCCTCTCCCCGCTTGCGTTGGCTGAGTTAAGAACAATACTGCTTTTTAGCTGGCACG
ACAGGTTTCCCAGGCCGGAAAGCGGGCAGTGAGCGCGCAAGTCGGGTGAAGTTAGCTCGCTCATTAGGCACCCAGGCTTACACTTTATGGCTGACTCGTATGTTGTGTGGAATTGTGAGCGGATAACAATTTCCACTGGGGTGTTTCGAGAT
GGAGCATCACAACTACAACCTTCTCGGAAGTAACTATACTTGGGACTTAAAAACATAATCATAATCATTTTTCTAAAACGATCAAGACTGATAACCATTTGACAAGAGCCATACAGACAAGCACCAACTGGCACTCTTAGGTCTTCACGTAT
GGTCATCGGTTTGGGTTCCATTTAT

Read #20 Alb-BB-L1-chr5-BB-Alb @1955fd7b-f139-4c6d-9951-ffebbbf333b9_R

CAATGCAAGGCACGTACGTTTACTATGTCATTGCCTATGGCTATGAAGTAAATCCTAACAGTCCCATAATACTTTTCTAACATCCATCATTTCTTTGTTTTCAGGGTCCAAACTCTTGTCACTAGATGCAAAGACGCCTTAGCATCTGCACC
ACCTGTGTGAAATTGTTATCCGCCTCACAATTCCACACAACATACGAGCCGGAAGCATAAAGTGTAAAGCCTGGGGTGCCTAATGAGTGAGCTAACTCACCTTAATTGCGTTGTGCTCACTGCCCGCTTTCCAGTCGGGAAACCTGTCGCCA
GCTGCATTAACAGAATCGGCCAACGCGCGGGAGAGGCGGTTTGCGTATTGGCGCCCTTCCGCTTCCTCGCTCACTGACTCGCTGCGCTCGGTCGTCTGGCCAGCAGATGGCACTCAGCTCACCCGTGCGGTAATACGGTTATCCACAGAATC
AGGGATAACGCAGGAAAGAACATGTGAGCAAGCAAAAGGCCAGGAACTGTAAAAGCGTCGCGTTGCTGGCGTTTTCCATAGTTCTGCCCTGACGAGCATCACAAAAATCGACGCTCAAGTCAGAGGTGGCGAAACCTGACAGGACTATAAAG
ATACCAGGCGTTTCCCTGAAGCTCCTCGTGCGCTCTCCTGTTCCGACCCTGCCGCCACCGATACCTGTCCGCCTTTCTCCTTCGGGAAGCGTGGCGCTTTCATAGTCGCTACGCTGGTGGCATCTTTGTGTTCGGTGTAGGTCGTTCGCTCC
AAGCTGGGCTGTGTGCACGAACCCCCTGTTCACGATCACGTACTATCCGGTAACTATCGTCTTGAGTCCAACCCGGTAAGACACACGACTTATCGCCACTGGCAGCAGCCACTGGTAACAGGATTACAGAGCGAGGTATGTAGGCGGTGCTA
CAGAGTTCTTGAAGTGGTGGCCTAACTACGGCTAAGCACTAGAAGAACAGTATTTGCATCTGCGCTCTGGCTGAAGCCAGTTACCTCGGAAAAAAGAGTTGGTATTTTGATCCGTGAACTACCGCTGGTAGCGGTGGGTTTTTGTTTGCAAG
CAGTAGATTACGCGCAGAAAAAAAAGGATCTCTGCACATTTTGATCTTTCTACGGGTCTGACGCTCAGTGGAACGAAAACTCACGTGTTGGCGATTTTGGTCATGATTATCAAAAGGATCTTCACTCTAGATCCTTTTAAATTAAAAATGAA
GTTTTAAATCAGCATATATGAGTAAACTTGGTCTGACAGGGCTACTAATGCTTAATCAGGAGGCACCTATCTCAAAGGATCTGTCTATTTCGTTCATCCATAGTTGCCTGACTCCCGTCGTGCAGATAACTACGATACGGGAGGGCTTACCA
TCTGGCCCCAGTGCTGCAATGATACCGGCGAGACCCACGCTCACCGGCTCCAGATTTATCAGCAATAGTTCAGCTAGTCAGGTCGCCGAGCAGAAGTGGTCCTGCAACTTTATCCGCCTCCATCCAGTCTATTAATTGTTGCCGGAAGCCAT
GCGCAGTTCGCCAGTTAATAGTTTGCGCAACGTTGCTGATATTGCTACAGGCATCGTGGTGTCACGCTCTGTCGTTTGGCATGGCTTCATCTTAGCCCGGCTCCCAACGATCAAGGCGAGTTACATGGATCCCCATGTTGTGCAAAGCGGTT
AGCTCCTCTCGGTCCTCCGACTTCGTCAGAAGTAAGTTGCCGCAGTGTTATCACTCATGGTTATGGCAGCACTGCATAATTCTCTTACTGTCATGCCATCCGTAAGATGCTTGTGACTGGTGAGTACTCAAACTGTCATTCTGAGAATAGCG
TGGCGGCGACCGAGCTGCTCTGCCCGGCGTCAATACGGGATAATACTCGCGCCACATAGCAGAACTTTAAAAGTGCTCATCATTGGAAAACGTTCTTCGGGGCGAAAACTCGCGATCTTACCGCTGTTGAGATCCAGTTCGATGTAACCCAC
TCGTGCACTCCAACTGATCTTCAGCATCTTACTTTCACCAGCGTTTCTGGGTGAGCAAAACAGGGAAGGCAAAATGCCGCAAAAAGCAAAGGGCACACACGGAAATGTTGAATACTCATACTTTCCTTTTCAATATTATTGAAGTATTTATC
AGGTTATTGTCCTCATGAGCGGATACATATTTGAATGTATTTAGGAAAATAAACAAATAGGTTCCAGCACATTTCCCCGAAAAGTGCCACCTGTGCAGCTACAAGTGTCTTGCATCTAGTGACAAGGTTTGGACCCTGAAAACAAAGAAATG
ATGGATGTTAGAAAAGTATTGAATAGACTGTTAGGATTTGCACTTCATAGCCTCATAGGCAATGACATAGTAAACGTACGTGCCTTTGCATTGTTAGATCCTAGCTAAGCTGCAAGTGAGACCTTCACCTTCCACTCTGATATCTGCACACT
TCCAGGAGAAGGAGAAGCAGATTAAGAAACAAACGTGAGCAATATTTTTATTGCATCTATTTTTCATACTGCATATGTATTGATAGTGAACTGACAATTTATATTTTTTTGTTTTGATGCAGTGATTATATATTCCTTAGAGAGAAGTGTTT
AGATGTTGGCACTGAGTTGAAAGAGTGTCAAAATTTTGTCTCAGAAAAAAATTTCACAAAATCTTCATTCAACTTATCTCTTTATTACTGTGGGCAAAACTACAGTAGAATAAAATTCTAGTGAGGCCCATTGAAGACACTAAATACCAAAGT
TAGTGGATATAGATAGAAGAACTGGTCATGAAATCCATACAAGTATTTATTGCAAGGTAGGAATAGTTGATTGAAATGTTCCACGTACATCTACACAATGAGTACTACTCAGCTATTAAAAAGAATGAATTTATGAAATTCCTAGCCAACAT
CATCCTGAGTGAGGTAACACATTCACAAAGAAACTCACACAACATGTATTCACTGATAAGTGGCCATTAGCCCCAAACCTAGGATCTAAGATATAAGATATAATTTGCTAAACACATGAAACTCAAGATAGGGGTGAAGACTGAAGTGTGGA
CACTATGCCCCTCCTTAGATTTGGGGGAACAAAACACCCATGGAAGGAGTTACAGAGACGGAGTTTGGAGCTGAGATGAAAGGATGGACCATGTAGAGACTGCCATAGCCAGATCCACCCCATAATCAGCATCCAAACGCTGACACCATTGC
ATCACTTGTGTTGTTGGGCCCCGGCATTCTCACAAGAGACAGCTACATCTGCGTCCTTTCAATAAAATCTTGGCTAGTGTATGCAATGGTGTCATGTTTGGATGCTGATTATGGGGTGGATCCCCGGCTGGGTGGCAGTCTCTACATGGTCC
ATCCTTTCATCTCAGCTCCAAACTCCGTCTCTGTAACTCCTTCCATGGGTGTTTTGTTCCCAAATCTAAGGAGGGGCATAGTGTCCACACTTCAGTCTTCATTCTCCTTGAGTTTCATGTGTTTAGCAAATTATATCTTATATCTTGGGTAT
CCTAGGTTTGGGGCTAATATCCACTTATCAGCAGAATACATATTGTGTGAGTTTCTTGTAGAATGTGTTACTCACTCAGGATGATGCCCTCCAGGTCCATCCATTTGGCTAGGAATTTATAAATTCATTCTTTTAATAGCTGAGTAGTAATT
TTACTGTGTAGATGTACGTGGAACATTTTCAATCAACTATTCCTACCTTTGCAATAAATACTTGTATGATTTCCATGACCAGTTCTTCTACTCCATATCCACTAACTGGTATTTAGTGTCTTCATTGGGCCTCCACTAGAATTTTATTCTAC
TGTGGTTTTGCCCACAGTAATAAAAAGATAAGTTGAATGAAAAGCATTTTCCATTAGAAAATTTTTTCACAGAGACAAAAAATTTTGATTAATTTTTTCAACTCAGTGATGCCAAATATCTAAACACTTCTCTTCTAAGGAATATATAATCA
CTGCATCAAAACAAAAATGTCCAGTTGTCAGTTCTCACTATCAATACATATGCAGTATGAAAATAGATGCAATAAAAATATTGCTCACGTTTGTTTTTAATCTGCTTCTCTCTGGAAGTGTGCAGATATCAGAGTGGAAGGGGTGAAGGTCT
CACTTGCAGCTTAGCTTGGGATCTAGCAATGCAAGGTACGCAGTACGTTTACTATGTCATTGCCTATGGCTATGAAGTGCAAATCCTAACAGTCCTGCTAATACTTTTCTAACATCCATCATTTCTCTCTCTCTCTCTCTCTCTCTCTCTCT
CTCTCTTTTTATATATATATATATATATATGCAGCATATATATTTCATGTAACTAAACTCGTCACTAGATGCAAAGACGCCTTAGACCAGGTGGCACTTTGGGAAATGTGCGCGGAACCCCTATTTGTTTATTTTTCTAAATACATTCAAATAT
GTATCCGCTCATGAGACAATAACCTCTGATAAATGCTTCAATAATATTAGAAAAAGGAAGAGTATGAGCATTCAACATTTCCGTGTCGCTCCTTTTATTCCTTTTTTATGGCATTTTGCCTTCCTTGTTTTTGGCTCACCCAGAAACGCTGG
TGAAAGTAAAAGATGCTGAAGATCGCTGGTGCACGAGGGGTTATACTGAACTGGATCTCAACAGCGGTAAGATCCTTGAGAGTTTTCGCCCCGAAGAACGTTTTCCAATGATGAGCACTCCTGAAGTTCACAGGTGCGGTATTATCCCGTAT
TGACGCCGGGCAAGAGCAACTCGGTCGCCGCATACACTATTTCTCAGAATGACTTGGCTGAGTACTCACCAGTCACAGAAAAGCATCTACGGATGGCATGACAGTAAGAGAATTATGGAGTGCTGCCATAACCATGAGTGATAACACTGCGG
CCACTTCATTTTGACAACGATCGGAGGACCGGAAGGATTAACCGCTTTTTTGCACAACATGGGGATCATGTAACTCCGCCTTGATCGTTGTGGGGAACCGGAGCTGAATGAAGTCATACCAAACGACGAGCGTGACACCACGATGCTGTAGC
AATGGCAACAACGTTGCGCAAATTATTAACTGGCGAACTACTACTCTAGCTTCCCGGCAACAATTAATAGACTGGATGGAGGCGGACAGTTGCAGGACCACTTGCAGCTCGGCCCTTCCGCTGGCTGGCGACAGATAAATCTGAGCCGGTGA
GCGTGGGTCCGCGAACATTGCAGCACTGGGGCCAGATGGTAAGCCCTCCCGTATCGTAGTGTTATCTACACGACGGGGAGTCAGGCAACTATGGATGAACGAAATAGACAGATCGCTGAGATAGGTGCCCCACTGATTAGCATTGGTAACTG
TCAGACCAAGTTTACTCATATATACTCTTTAGATTGATTTAAAACTTCATTTTTAATTTAAAAGGATCTAGGTGAAGATCCTTTTTGATAATCTCATGACCAAAAAATCCCTTAACGTGAGTTTTCGTTCCACTGAGCGTCAGACCCCGTAGAA
AAGATCAAAGGATCTTCCTGAGATCCTTTTTTCTGCGCGCGTAATCTGCTGCTTGCAAACAAAAAAAACCACCGCTACCAGCGGTTTGTCGGCCGGATCAAGAGCTACCAACTCTTTTTCCGAAGGTAACTGGCTTCAGCAGCGCAGATATC
AAATACCGTTCTTCTAGTGTAGCCGTAGTTAGCCACCACTTCAAGAACTCTAGCACCGCCTACATACCTCGCTCTGCTAATCCTGTTACCAGTGGCTGCTGCCAGTGGCGATAAGTCGTGTCTTACCGGGTTGGACTCAAGACGATAGTTAC
CGGATAAGGCGCAGCGGTCGGGCTGAACGGGGTTCGTGCACACAGCCCAGCTTGGAGCGAACGACCTACACCGAACTGAGATAATTTACAGCGTGAGCTATGAGAAAGCGCCACGCTTCCCGAAGGGAGAAAGGCGACAGGTATCCGGTAAG
CGGCAGGGGGTCGGAACAGGAGAGCGCACGAGGAGCTTCCAGGGGAAACGCCTGGTAATCTTTATAGTCCTGTCGGTTTCCGCACTCTGACTTGAGCGTCGATTTTGTGATGCTCGTCAGGGGCGGAGCCTATGAAAACGCCAGCAACGCGG
CCTTTTACGGTTCCTGGCCTTTGGCTGGCCTTTTGCTCACATGTTCTTCCTGCGTTATCCCCTGATTCTGTGGATAACCGTATTACCGCCTTTGAGTGAGCTTTCGATACTGCTCGCCGCAGCCGAACGACCGAGCGCAGCGAGTCAGTGAG
CGAGGAAGCGGAAGAGCGCCCAATACGCAAACCGCCTCCCCGCGCGTTGGCCGATTCATTAATGCAGCTGTTCGACAGGTTTCCTGACTGGAAAGCGCGGGCAGTGAGCGCAACGCAATTAAGGTGAGCAGCTCACCCATTAGGCACCCCAG
GCTTTACACTTTATGCTTCCGGTTGTATGTTGTGTGGAATTGTGAGCGGATAACAATTTCTACACAGGTGGTGCAGATGAACTTATACACACACAAATCCAACCTTCTCAGGTAACTATACTTGGGACTTAAAAACATAATCATAATCATTT
TTCCTAAAACGATCAAGACTGATAACCATTTGACAAGAGCCATACAGGCCGGGCACCAGCTGGCACTCTTAGGTCTTCACGTATGGTCATCAGTTTGGGTCTCCATTTGTAGAT

Figure S13. (D) The representative read sequences show the insertion of the plasmid backbone, LINE-1, and genomic DNA fragment at Alb.

pD-sgDocut-LA190-E2A-BDDF8-Wpre-PolyA-RA130-sgDocut-Backbone（sgBB）

GGTGGTGCAGATGAACTCCATGGGGTCAGAGAGCCTGCTTTAGGAATTCTAAGTAGAACTGTAATTAAGCAATGCAAGGCACGTACGTTTACTATGTCATTGCCTATGGCT
ATGAAGTGCAAATCCTAACAGTCCTGCTAATACTTTTCTAACATCCATCATTTCTTTGTTTTCAGGGTCCAAACCTTGTCACTAGATGCAAAGACGCCTTAGCCCAGTGT
ACTAATTATGCTCTCTTGAAATTGGCTGGAGATGTTGAGAGCAACCCAGGTCCCATGCAAATAGAGCTCTCCACCTGCTTCTTTCTGTGCCTTTTGCGATTCTGCTTTAG
TGCCACCAGAAGATACTACCTGGGTGCAGTGGAACTGTCATGGGACTATATGCAAAGTGATCTCGGTGAGCTGCCTGTGGACGCAAGATTTCCTCCTAGAGTGCCAA
AATCTTTTCCATTCAACACCTCAGTCGTGTACAAAAAGACTCTGTTTGTAGAATTCACGGATCACCTTTTCAACATCGCTAAGCCAAGGCCACCCTGGATGGGTCTGCT
AGGTCCTACCATCCAGGCTGAGGTTTATGATACAGTGGTCATTACACTTAAGAACATGGCTTCCCATCCTGTCAGTCTTCATGCTGTTGGTGTATCCTACTGGAAAGCT
TCTGAGGGAGCTGAATATGATGATCAGACCAGTCAAAGGGAGAAAGAAGATGATAAAGTCTTCCCTGGTGGAAGCCATACATATGTCTGGCAGGTCCTGAAAGAGAA
TGGTCCAATGGCCTCTGACCCACTGTGCCTTACCTACTCATATCTTTCTCATGTGGACCTGGTAAAAGACTTGAATTCAGGCCTCATTGGAGCCCTACTAGTATGTAGA
GAAGGGAGTCTGGCCAAGGAAAAGACACAGACCTTGCACAAATTTATACTACTTTTTGCTGTATTTGATGAAGGGAAAAGTTGGCACTCAGAAACAAAGAACTCCTTG
ATGCAGGATAGGGATGCTGCATCTGCTCGGGCCTGGCCTAAAATGCACACAGTCAATGGTTATGTAAACAGGTCTCTGCCAGGTCTGATTGGATGCCACAGGAAATC
AGTCTATTGGCATGTGATTGGAATGGGCACCACTCCTGAAGTGCACTCAATATTCCTCGAAGGTCACACATTTCTTGTGAGGAACCATCGCCAGGCGTCCTTGGAAAT
CTCGCCAATAACTTTCCTTACTGCTCAAACACTCTTGATGGACCTTGGACAGTTTCTACTGTTTTGTCATATCTCTTCCCACCAACATGATGGCATGGAAGCTTATGTCA
AAGTAGACAGCTGTCCAGAGGAACCCCAACTACGAATGAAAAATAATGAAGAAGCGGAAGACTATGATGATGATCTTACTGATTCTGAAATGGATGTGGTCAGGTTT
GATGATGACAACTCTCCTTCCTTTATCCAAATTCGCTCAGTTGCCAAGAAGCATCCTAAAACTTGGGTACATTACATTGCTGCTGAAGAGGAGGACTGGGACTATGCT
CCCTTAGTCCTCGCCCCCGATGACAGAAGTTATAAAAGTCAATATTTGAACAATGGCCCTCAGCGGATTGGTAGGAAGTACAAAAAAGTCCGATTTATGGCATACACA
GATGAAACCTTTAAGACTCGTGAAGCTATTCAGCATGAATCAGGAATCTTGGGACCTTTACTTTATGGGGAAGTTGGAGACACACTGTTGATTATATTTAAGAATCAAG
CAAGCAGACCATATAACATCTACCCTCACGGAATCACTGATGTCCGTCCTTTGTATTCAAGGAGATTACCAAAAGGTGTAAAACATTTGAAGGATTTTCCAATTCTGCC
AGGAGAAATATTCAAATATAAATGGACAGTGACTGTAGAAGATGGGCCAACTAAATCAGATCCTCGGTGCCTGACCCGCTATTACTCTAGTTTCGTTAATATGGAGAG
AGATCTAGCTTCAGGACTCATTGGCCCTCTCCTCATCTGCTACAAAGAATCTGTAGATCAAAGAGGAAACCAGATAATGTCAGACAAGAGGAATGTCATCCTGTTTTC
TGTATTTGATGAGAACCGAAGCTGGTACCTCACAGAGAATATACAACGCTTTCTCCCCAATCCAGCTGGAGTGCAGCTTGAGGATCCAGAGTTCCAAGCCTCCAACA
TCATGCACAGCATCAATGGCTATGTTTTTGATAGTTTGCAGTTGTCAGTTTGTTTGCATGAGGTGGCATACTGGTACATTCTAAGCATTGGAGCACAGACTGACTTCCTT
TCTGTCTTCTTCTCTGGATATACCTTCAAACACAAAATGGTCTATGAAGACACACTCACCCTATTCCCATTCTCAGGAGAAACTGTCTTCATGTCGATGGAAAACCCAG
GTCTATGGATTCTGGGGTGCCACAACTCAGACTTTCGGAACAGAGGCATGACCGCCTTACTGAAGGTTTCTAGTTGTGACAAGAACACTGGTGATTATTACGAGGACA
GTTATGAAGATATTTCAGCATACTTGCTGAGTAAAAACAATGCCATTGAACCAAGAAGCTTCTCTCAAAACCCACCAGTCTTGAAACGCCATCAACGGGAAATAACTC
GTACTACTCTTCAGTCAGATCAAGAGGAAATTGACTATGATGATACCATATCAGTTGAAATGAAGAAGGAAGATTTTGACATTTATGATGAGGATGAAAATCAGAGCC
CCCGCAGCTTTCAAAAGAAAACACGACACTATTTTATTGCTGCAGTGGAGAGGCTCTGGGATTATGGGATGAGTAGCTCCCCACATGTTCTAAGAAACAGGGCTCAG
AGTGGCAGTGTCCCTCAGTTCAAGAAAGTTGTTTTCCAGGAATTTACTGATGGCTCCTTTACTCAGCCCTTATACCGTGGAGAACTAAATGAACATTTGGGACTCCTGG
GGCCATATATAAGAGCAGAAGTTGAAGATAATATCATGGTAACTTTCAGAAATCAGGCCTCTCGTCCCTATTCCTTCTATTCTAGCCTTATTTCTTATGAGGAAGATCA
GAGGCAAGGAGCAGAACCTAGAAAAAACTTTGTCAAGCCTAATGAAACCAAAACTTACTTTTGGAAAGTGCAACATCATATGGCACCCACTAAAGATGAGTTTGACT
GCAAAGCCTGGGCTTATTTCTCTGATGTTGACCTGGAAAAAGATGTGCACTCAGGCCTGATTGGACCCCTTCTGGTCTGCCACACTAACACACTGAACCCTGCTCATG
GGAGACAAGTGACAGTACAGGAATTTGCTCTGTTTTTCACCATCTTTGATGAGACCAAAAGCTGGTACTTCACTGAAAATATGGAAAGAAACTGCAGGGCTCCCTGCA
ATATCCAGATGGAAGATCCCACTTTTAAAGAGAATTATCGCTTCCATGCAATCAATGGCTACATAATGGATACACTACCTGGCTTAGTAATGGCTCAGGATCAAAGGA
TTCGATGGTATCTGCTCAGCATGGGCAGCAATGAAAACATCCATTCTATTCATTTCAGTGGACATGTGTTCACTGTACGAAAAAAAGAGGAGTATAAAATGGCACTGT
ACAATCTCTATCCAGGTGTTTTTGAGACAGTGGAAATGTTACCATCCAAAGCTGGAATTTGGCGGGTGGAATGCCTTATTGGCGAGCATCTACATGCTGGGATGAGCA
CACTTTTTCTGGTGTACAGCAATAAGTGTCAGACTCCCCTGGGAATGGCTTCTGGACACATTAGAGATTTTCAGATTACAGCTTCAGGACAATATGGACAGTGGGCCC
CAAAGCTGGCCAGACTTCATTATTCCGGATCAATCAATGCCTGGAGCACCAAGGAGCCCTTTTCTTGGATCAAGGTGGATCTGTTGGCACCAATGATTATTCACGGCA
TCAAGACCCAGGGTGCCCGTCAGAAGTTCTCCAGCCTCTACATCTCTCAGTTTATCATCATGTATAGTCTTGATGGGAAGAAGTGGCAGACTTATCGAGGAAATTCCA
CTGGAACCTTAATGGTCTTCTTTGGCAATGTGGATTCATCTGGGATAAAACACAATATTTTTAACCCTCCAATTATTGCTCGATACATCCGTTTGCACCCAACTCATTAT
AGCATTCGCAGCACTCTTCGCATGGAGTTGATGGGCTGTGATTTAAATAGTTGCAGCATGCCATTGGGAATGGAGAGTAAAGCAATATCAGATGCACAGATTACTGCT
TCATCCTACTTTACCAATATGTTTGCCACCTGGTCTCCTTCAAAAGCTCGACTTCACCTCCAAGGGAGGAGTAATGCCTGGAGACCTCAGGTGAATAATCCAAAAGAG
TGGCTGCAAGTGGACTTCCAGAAGACAATGAAAGTCACAGGAGTAACTACTCAGGGAGTAAAATCTCTGCTTACCAGCATGTATGTGAAGGAGTTCCTCATCTCCAG
CAGTCAAGATGGCCATCAGTGGACTCTCTTTTTTCAGAATGGCAAAGTAAAGGTTTTTCAGGGAAATCAAGACTCCTTCACACCTGTGGTGAACTCTCTAGACCCACC
GTTACTGACTCGCTACCTTCGAATTCACCCCCAGAGTTGGGTGCACCAGATTGCCCTGAGGATGGAGGTTCTGGGCTGCGAGGCACAGGACCTCTACTGAGTTTAAA
CGTCGACAATCAACCTCTGGATTACAAAATTTGTGAAAGATTGACTGGTATTCTTAACTATGTTGCTCCTTTTACGCTATGTGGATACGCTGCTTTAATGCCTTTGTATC
ATGCTATTGCTTCCCGTATGGCTTTCATTTTCTCCTCCTTGTATAAATCCTGGTTGCTGTCTCTTTATGAGGAGTTGTGGCCCGTTGTCAGGCAACGTGGCGTGGTGTGC
ACTGTGTTTGCTGACGCAACCCCCACTGGTTGGGGCATTGCCACCACCTGTCAGCTCCTTTCCGGGACTTTCGCTTTCCCCCTCCCTATTGCCACGGCGGAACTCATC
GCCGCCTGCCTTGCCCGCTGCTGGACAGGGGCTCGGCTGTTGGGCACTGACAATTCCGTGGTGTTGTCGGGGAAGCTGACGTCCTTTCCATGGCTGCTCGCCTGTGT
TGCCACCTGGATTCTGCGCGGGACGTCCTTCTGCTACGTCCCTTCGGCCCTCAATCCAGCGGACCTTCCTTCCCGCGGCCTGCTGCCGGCTCTGCGGCCTCTTCCGC
GTCTCGCCTTCGCCCTCAGACGAGTCGGATCTCCCTTTGGGCGGATCCAGACATGATAAGATACATTGATGAGTTTGGACAAACCACAACTAGAATGCAGTGAAAAA
AATGCTTTATTTGTGAAATTTGTGATGCTATTGCTTTATTTGTAACCATTATAAGCTGCAATAAACAAGTTAACAACAACAATTGCATTCATTTTATGTTTCAGGTTCAGG
GGGAGGTGTGGGAGGTTTTTTAAAGCAAGTAAAACCTCTACAAATGTGGTATGGCTGATTATGATCCGGCTGCCTCGCGCGTTTCGGTGATGACGGTGAAAACCTCTG
ACACATGCAGCTCCCGGAGACGGTCACAGCTTGTCTGTAAGCGGATGCCGGGAGCAGACAAGCCCGTCAGGCGTCAGCGGGTGTTGGCGGGTGTCGGGGCGCAGC
CATGAGGTTTAAACTAAACACATCACAACCACAACCTTCTCAGGTAACTATACTTGGGACTTAAAAAACATAATCATAATCATTTTTCCTAAAACGATCAAGACTGATA
ACCATTTGACAAGAGCCATACAGACAAGCACCCCATGGAGTTCATCTGCACCACCAGGTGGCACTTTTCGGGGAAATTGCGCGGAACCCCTATTTGTTTATTTTTCTAAA
TACATTCAAATATGTATCCGCTCATGAGACAATAACCCTGATAAATGCTTCAATAATATTGAAAAAGGAAGAGTATGAGTATTCAACATTTCCGTGTCGCCCTTATTCC
CTTTTTTGCGGCATTTTGCCTTCCTGTTTTTGCTCACCCAGAAACGCTGGTGAAAGTAAAAGATGCTGAAGATCAGTTGGGTGCACGAGTGGGTTACATCGAACTGGAT
CTCAACAGCGGTAAGATCCTTGAGAGTTTTCGCCCCGAAGAACGTTTTCCAATGATGAGCACTTTTAAAGTTCTGCTATGTGGCGCGGTATTATCCCGTATTGACGCCG
GGCAAGAGCAACTCGGTCGCCGCATACACTATTCTCAGAATGACTTGGTTGAGTACTCACCAGTCACAGAAAAGCATCTTACGGATGGCATGACAGTAAGAGAATTA
TGCAGTGCTGCCATAACCATGAGTGATAACACTGCGGCCAACTTACTTCTGACAACGATCGGAGGACCGAAGGAGCTAACCGCTTTTTTGCACAACATGGGGGATCA
TGTAACTCGCCTTGATCGTTGGGAACCGGAGCTGAATGAAGCCATACCAAACGACGAGCGTGACACCACGATGCCTGTAGCAATGGCAACAACGTTGCGCAAACTA
TTAACTGGCGAACTACTTACTCTAGCTTCCCGGCAACAATTAATAGACTGGATGGAGGCGGATAAAGTTGCAGGACCACTTCTGCGCTCGGCCCTTCCGGCTGGCTG
GTTTATTGCTGATAAATCTGGAGCCGGTGAGCGTGGGTCTCGCGGTATCATTGCAGCACTGGGGCCAGATGGTAAGCCCTCCCGTATCGTAGTTATCTACACGACGG
GGAGTCAGGCAACTATGGATGAACGAAATAGACAGATCGCTGAGATAGGTGCCTCACTGATTAAGCATTGGTAACTGTCAGACCAAGTTTACTCATATATACTTTAGA
TTGATTTAAAACTTCATTTTTAATTTAAAAGGATCTAGGTGAAGATCCTTTTTGATAATCTCATGACCAAAATCCCTTAACGTGAGTTTTCGTTCCACTGAGCGTCAGAC
CCCGTAGAAAAGATCAAAGGATCTTCTTGAGATCCTTTTTTTCTGCGCGTAATCTGCTGCTTGCAAACAAAAAAACCACCGCTACCAGCGGTGGTTTGTTTGCCGGATC
AAGAGCTACCAACTCTTTTTCCGAAGGTAACTGGCTTCAGCAGAGCGCAGATACCAAATACTGTCCTTCTAGTGTAGCCGTAGTTAGGCCACCACTTCAAGAACTCTG
TAGCACCGCCTACATACCTCGCTCTGCTAATCCTGTTACCAGTGGCTGCTGCCAGTGGCGATAAGTCGTGTCTTACCGGGTTGGACTCAAGACGATAGTTACCGGATA
AGGCGCAGCGGTCGGGCTGAACGGGGGGTTCGTGCACACAGCCCAGCTTGGAGCGAACGACCTACACCGAACTGAGATACCTACAGCGTGAGCTATGAGAAAGCG
CCACGCTTCCCGAAGGGAGAAAGGCGGACAGGTATCCGGTAAGCGGCAGGGTCGGAACAGGAGAGCGCACGAGGGAGCTTCCAGGGGGAAACGCCTGGTATCTTT
ATAGTCCTGTCGGGTTTCGCCACCTCTGACTTGAGCGTCGATTTTTGTGATGCTCGTCAGGGGGGCGGAGCCTATGGAAAAACGCCAGCAACGCGGCCTTTTTACGGT
TCCTGGCCTTTTGCTGGCCTTTTGCTCACATGTTCTTTCCTGCGTTATCCCCTGATTCTGTGGATAACCGTATTACCGCCTTTGAGTGAGCTGATACCGCTCGCCGCAGC
CGAACGACCGAGCGCAGCGAGTCAGTGAGCGAGGAAGCGGAAGAGCGCCCAATACGCAAACCGCCTCTCCCCGCGCGTTGGCCGATTCATTAATGCAGCTGGCAC
GACAGGTTTCCCGACTGGAAAGCGGGCAGTGAGCGCAACGCAATTAATGTGAGTTAGCTCACTCATTAGGCACCCCAGGCTTTACACTTTATGCTTCCGGCTCGTATG
TTGTGTGGAATTGT GAG CGGATAACAATTTCACACAGGA

Figure S14. The sequence of pD-BDDF8-sg vectors with HA190-130. The backbone used for the plasmids in our study is derived from pBR322, a well-known bacterial cloning vector.

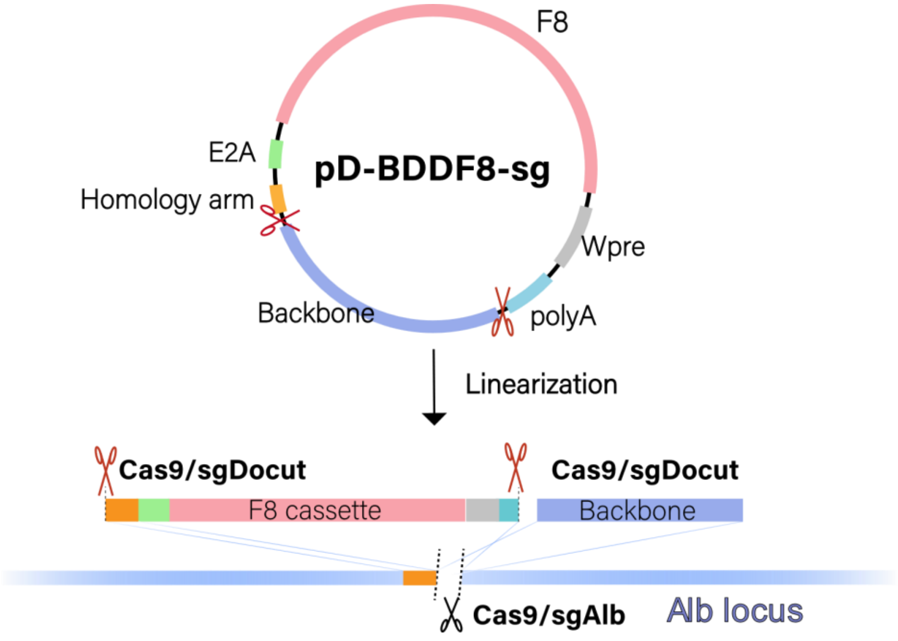

Figure S15. Linearization and Integration of Double-Cut Donor Plasmid: This schematic illustrates the process where a double-cut donor plasmid is linearized into two distinct fragments within hepatocytes. Subsequently, these fragments integrate at the genomic site cleaved by sgAlb. The diagram provides a visual representation of the linearization and integration steps, highlighting the two resulting fragments and their integration at the targeted genomic location.

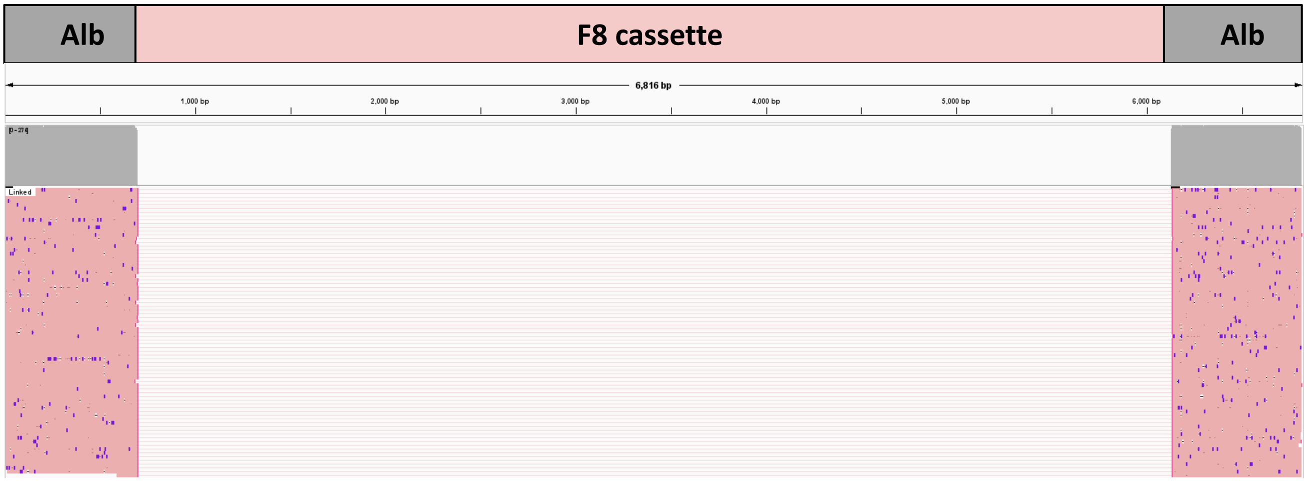

Figure S16. Analysis of Circular Donor Plasmid Integration: This figure analyzes the integration of a circular donor plasmid with HA600-600 homology arms, which lacks the sgDocut, into the target genomic site. The reference sequence used for alignment is based on the expected outcome of homology-directed repair (HDR) editing. This panel shows random 200 reads out of over 6000 aligned with the reference sequence. Nanopore sequencing of over 6,000 reads failed to reveal any F8 insertions. This lack of detection likely resulted from the preferential amplification of shorter background sequences (1.3 kb) without insertions, overshadowing potential longer insertions.

Target site	sgRNA Sequence
sgAlb-E14	GTTGTGATGTGTTTAGGCTA
sgDocut	GGTGGTGCAGATGAACTCCA
sgBB	AATAAACCAGCCAGCCGGAA

Table S1. The target sequences of sgRNAs utilized in this study.

Primer	Sequence	Length
Alb-PE150-F-BC1	aacaaggtATTGCCTATGGCTATGAAGTGC	242 bp
Alb-PE150-F-BC2	aacattccATTGCCTATGGCTATGAAGTGC	
Alb-PE150-F-BC3	aaccgtaaATTGCCTATGGCTATGAAGTGC	
Alb-PE150-F-BC4	aacgagaaATTGCCTATGGCTATGAAGTGC	
Alb-PE150-F-BC5	aacttggaATTGCCTATGGCTATGAAGTGC	
Alb-PE150-F-BC6	aagaccaaATTGCCTATGGCTATGAAGTGC	
Alb-PE150-F-BC7	aaggtcatATTGCCTATGGCTATGAAGTGC	
Alb-PE150-F-BC8	aatagtggATTGCCTATGGCTATGAAGTGC	
Alb-PE150-F-BC9	aatgccttATTGCCTATGGCTATGAAGTGC	
Alb-PE150-F-BC10	acaatagcATTGCCTATGGCTATGAAGTGC	
Alb-PE150-F-BC11	acaggattATTGCCTATGGCTATGAAGTGC	
Alb-PE150-F-BC12	accgacctATTGCCTATGGCTATGAAGTGC	
Alb-PE150-F-BC13	acgagtccATTGCCTATGGCTATGAAGTGC	
Alb-PE150-F-BC14	acgcataaATTGCCTATGGCTATGAAGTGC	
Alb-PE150-F-BC15	acggacgaATTGCCTATGGCTATGAAGTGC	
Alb-PE150-F-BC16	acgtatggATTGCCTATGGCTATGAAGTGC	
Alb-PE150-F-BC17	actaaccaATTGCCTATGGCTATGAAGTGC	
Alb-PE150-F-BC18	actcaggtATTGCCTATGGCTATGAAGTGC	
Alb-PE150-F-BC19	acttgttgATTGCCTATGGCTATGAAGTGC	
Alb-PE150-F-BC20	agaagtacATTGCCTATGGCTATGAAGTGC	
Alb-PE150-F-BC21	agccactcATTGCCTATGGCTATGAAGTGC	
Alb-PE150-F-BC22	agctctaaATTGCCTATGGCTATGAAGTGC	
Alb-PE150-F-BC23	aggtgtctATTGCCTATGGCTATGAAGTGC	
Alb-PE150-F-BC24	agtccgttATTGCCTATGGCTATGAAGTGC	
Alb-PE150-R	TGCTTGTCTGTATGGCTCTTGT	

Table S2. The primers employed to determine the cleavage efficiencies of Cas9-sgRNA that targets Alb.

Primer	Sequence	Length
Alb-4.9kb-F-BC1	gccggttggaaTGAGACCTTCACCTTCCACTCTG	4886 bp
Alb-4.9kb-F-BC2	ggtatagatggTGAGACCTTCACCTTCCACTCTG	
Alb-4.9kb-F-BC3	gtacttgttagTGAGACCTTCACCTTCCACTCTG	
Alb-4.9kb-F-BC4	taagctgcaagTGAGACCTTCACCTTCCACTCTG	
Alb-4.9kb-F-BC5	tcgcctccatcTGAGACCTTCACCTTCCACTCTG	
Alb-4.9kb-F-BC6	ttagaatcacgTGAGACCTTCACCTTCCACTCTG	
Alb-4.9kb-F-BC7	ttcaggtgttcTGAGACCTTCACCTTCCACTCTG	
Alb-4.9kb-F-BC8	ttctgcaccgcTGAGACCTTCACCTTCCACTCTG	
Alb-4.9kb-F-BC9	caatcttacagTGAGACCTTCACCTTCCACTCTG	
Alb-4.9kb-F-BC10	ccaggaggtgtTGAGACCTTCACCTTCCACTCTG	
Alb-4.9kb-F-BC11	cgagactattcTGAGACCTTCACCTTCCACTCTG	
Alb-4.9kb-F-BC12	gacctgacatcTGAGACCTTCACCTTCCACTCTG	
Alb-4.9kb-F-BC13	aacaacaacacTGAGACCTTCACCTTCCACTCTG	
Alb-4.9kb-F-BC14	aacattgagccTGAGACCTTCACCTTCCACTCTG	
Alb-4.9kb-F-BC15	aagaccataggTGAGACCTTCACCTTCCACTCTG	
Alb-4.9kb-F-BC16	aatagtgtcggTGAGACCTTCACCTTCCACTCTG	
Alb-4.9kb-F-BC17	acaatgcgaatTGAGACCTTCACCTTCCACTCTG	
Alb-4.9kb-F-BC18	accgctatgccTGAGACCTTCACCTTCCACTCTG	
Alb-4.9kb-F-BC19	acgtaggataaTGAGACCTTCACCTTCCACTCTG	
Alb-4.9kb-F-BC20	actattactccTGAGACCTTCACCTTCCACTCTG	
Alb-4.9kb-F-BC21	agctcggacctTGAGACCTTCACCTTCCACTCTG	
Alb-4.9kb-F-BC22	agtctagcgttTGAGACCTTCACCTTCCACTCTG	
Alb-4.9kb-F-BC23	atggaggacggTGAGACCTTCACCTTCCACTCTG	
Alb-4.9kb-F-BC24	attggtgcataTGAGACCTTCACCTTCCACTCTG	
Alb-4.9kb-R	TGGCTCGTTATTAGCCCAGTTGATAGC	

Table S3. The primers utilized to detect large deletions in Figure 1C.

Primer	Sequence	Length
Alb-4.7kb-F-BC1	aacaccttcttTGTGGGCAAAAACCACAGTA	4727 bp
Alb-4.7kb-F-BC2	aaccgtccaccTGTGGGCAAAAACCACAGTA	
Alb-4.7kb-F-BC3	aagcggttataTGTGGGCAAAAACCACAGTA	
Alb-4.7kb-F-BC4	aattccgcgctTGTGGGCAAAAACCACAGTA	
Alb-4.7kb-F-BC5	acctcctgtaaTGTGGGCAAAAACCACAGTA	
Alb-4.7kb-F-BC6	acggaaccagcTGTGGGCAAAAACCACAGTA	
Alb-4.7kb-F-BC7	agaagaagagaTGTGGGCAAAAACCACAGTA	
Alb-4.7kb-F-BC8	aggtgagtcttTGTGGGCAAAAACCACAGTA	
Alb-4.7kb-R	ACCTGGCTCGTTATTAGCCCAGTTGATAG	

Table S4. The primers utilized for long-range PCR amplification in Figure 2A.

Primer	Sequence	Length
Alb-HA600-1.5kb-F1	GGAGCAACTGAAGACTGTCATGGA	1586 bp
Alb-HA600-1.5kb-R1	AGTGTCAAAGTCAGGGGTCTCACC	
Alb-HA600-1.4kb-F2-BC1	aaacactttctcCGGAGCAACTGAAGACTGTCA	1380 bp
Alb-HA600-1.4kb-F2-BC2	ttaacgaaaccgCGGAGCAACTGAAGACTGTCA	
Alb-HA600-1.4kb-F2-BC3	aaactctgagaaCGGAGCAACTGAAGACTGTCA	
Alb-HA600-1.4kb-F2-BC4	tgtatcaaagacCGGAGCAACTGAAGACTGTCA	
Alb-HA600-1.4kb-F2-BC5	aacgtctccataCGGAGCAACTGAAGACTGTCA	
Alb-HA600-1.4kb-F2-BC6	tcgattaactgaCGGAGCAACTGAAGACTGTCA	
Alb-HA600-1.4kb-F2-BC7	acaaagtaacccCGGAGCAACTGAAGACTGTCA	
Alb-HA600-1.4kb-F2-BC8	tagcgaacaaccCGGAGCAACTGAAGACTGTCA	
Alb-HA600-1.4kb-F2-BC9	acagcagtttggCGGAGCAACTGAAGACTGTCA	
Alb-HA600-1.4kb-F2-BC10	gtagagacatgtCGGAGCAACTGAAGACTGTCA	
Alb-HA600-1.4kb-F2-BC11	accatagctaagCGGAGCAACTGAAGACTGTCA	
Alb-HA600-1.4kb-F2-BC12	gcactaacctttCGGAGCAACTGAAGACTGTCA	
Alb-HA600-1.4kb-F2-BC13	atgcacgaacagCGGAGCAACTGAAGACTGTCA	
Alb-HA600-1.4kb-F2-BC14	gatatgatgttgCGGAGCAACTGAAGACTGTCA	
Alb-HA600-1.4kb-F2-BC15	caaacacttccaCGGAGCAACTGAAGACTGTCA	
Alb-HA600-1.4kb-F2-BC16	cgtttccaatacCGGAGCAACTGAAGACTGTCA	
Alb-HA600-1.4kb-R2	AGCCTTCCATTATTGCCCTATT	

Table S5. The primers employed for nested PCR amplification of long genomic flanking regions in Supplementary Figure S2.

Primer	Sequence	Length
Alb-HA85-0.5kb-F1	Acaacagatgtcagagagcctgc	499 bp
Alb-HA85-0.5kb-R1	Cagggctaactcctttgctctgg	
Alb-HA85-332bp-F2-BC1	acgccacgttAGCAATGCAAGGCACGTACG	332 bp
Alb-HA85-332bp-F2-BC2	cgactagaccAGCAATGCAAGGCACGTACG	
Alb-HA85-332bp-F2-BC3	cagtgaccagAGCAATGCAAGGCACGTACG	
Alb-HA85-332bp-F2-BC4	ccaggctcttAGCAATGCAAGGCACGTACG	
Alb-HA85-332bp-F2-BC5	tcgcgacactAGCAATGCAAGGCACGTACG	
Alb-HA85-332bp-F2-BC6	agccgtcacaAGCAATGCAAGGCACGTACG	
Alb-HA85-332bp-F2-BC7	gtgcatatccAGCAATGCAAGGCACGTACG	
Alb-HA85-332bp-F2-BC8	accgagccatAGCAATGCAAGGCACGTACG	
Alb-HA85-332bp-F2-BC9	cactctcaggAGCAATGCAAGGCACGTACG	
Alb-HA85-332bp-F2-BC10	gtagtcaatgAGCAATGCAAGGCACGTACG	
Alb-HA85-332bp-F2-BC11	ccgtgtggtaAGCAATGCAAGGCACGTACG	
Alb-HA85-332bp-F2-BC12	gaatcgagacAGCAATGCAAGGCACGTACG	
Alb-HA85-332bp-F2-BC13	gacgcggtaaAGCAATGCAAGGCACGTACG	
Alb-HA85-332bp-F2-BC14	gaacaccacaAGCAATGCAAGGCACGTACG	
Alb-HA85-332bp-F2-BC15	caactcctgaAGCAATGCAAGGCACGTACG	
Alb-HA85-332bp-F2-BC16	cgattacctcAGCAATGCAAGGCACGTACG	
Alb-HA85-332bp-F2-BC17	gaccactatcAGCAATGCAAGGCACGTACG	
Alb-HA85-332bp-F2-BC18	gagacacggtAGCAATGCAAGGCACGTACG	
Alb-HA85-332bp-F2-BC19	gatatgcgttAGCAATGCAAGGCACGTACG	
Alb-HA85-332bp-F2-BC20	gcaacagaggAGCAATGCAAGGCACGTACG	
Alb-HA85-332bp-F2-BC21	gccagacaagAGCAATGCAAGGCACGTACG	
Alb-HA85-332bp-F2-BC22	gctaggatctAGCAATGCAAGGCACGTACG	
Alb-HA85-332bp-F2-BC23	ggagttaccgAGCAATGCAAGGCACGTACG	
Alb-HA85-332bp-F2-BC24	ggtacgtgcaAGCAATGCAAGGCACGTACG	
Alb-HA85-332bp-F2-BC25	gtactgacaaAGCAATGCAAGGCACGTACG	
Alb-HA85-332bp-F2-BC26	taacatgaggAGCAATGCAAGGCACGTACG	
Alb-HA85-332bp-F2-BC27	tacaacggatAGCAATGCAAGGCACGTACG	
Alb-HA85-332bp-R2	TATCTACAAATGGAACCCAAACTGATGACC	

Table S6. The primers utilized for nested PCR amplification of short genomic flanking regions in Figure 3.

Primer	Sequence	Length
qPCR-BDDF8-FL-F	CACGTACGTTTACTATGTCA	261bp
qPCR-BDDF8-FL-R	AGTATCTTCTGGTGGCACTA	
qPCR-BDDF8-FR-F	CTGTAAGCGGATGCCGGGAG	249bp
qPCR-BDDF8-FR-R	GCTGGTGCTTGTCTGTATGG	
qPCR-BDDF8-RL-F	GCACGTACGTTTACTATGTC	283bp
qPCR-BDDF8-RL-R	AAACCTCTGACACATGCAGC	
qPCR-BDDF8-RR-F	CCCAGGTAGTATCTTCTGGT	281bp
qPCR-BDDF8-RR-R	CCAGCTGGTGCTTGTCTGTA	
qPCR-BB-FL-F	CCTATGGCTATGAAGTGCAA	248bp
qPCR-BB-FL-R	GTTGAATACTCATACTCTTC	
qPCR-BB-FR-F	AAGCGGGCAGTGAGCGCAAC	248bp
qPCR-BB-FR-R	GGCTCTTGTCAAATGGTTAT	
qPCR-BB-RL-F	CACGTACGTTTACTATGTCA	277bp
qPCR-BB-RL-R	ACGACAGGTTTCCCGACTGG	
qPCR-BB-RR-F	CGCACATTTCCCCGAAAAGT	285bp
qPCR-BB-RR-R	TCTCCCTGGTTTGGTCTCCT	
qPCR-F8fBBr-junction-F	GCTTGTCTGTAAGCGGATGC	203bp
qPCR-F8fBBr-junction-R	CACTCATTAGGCACCCCAGG	
qPCR-F8rBBf-junction-F	GCACAGAAAGAAGCAGGTGGA	227bp
qPCR-F8rBBf-junction-R	CATTTCCCCGAAAAGTGCCA	

Table S7. The primers employed for qPCR to correlate with the proportion of different insertion outcomes analyzed based on Nanopore sequencing data in Figure 5.

Primer	Sequence	Length
**Forward left junction**		
Alb-S516-FL-F-BC1	aacattgagccCGTCATGGGTGTGACTTTTG	
Alb-S516-FL-F-BC2	aagttcattccCGTCATGGGTGTGACTTTTG	
Alb-S516-FL-F-BC3	accacagattaCGTCATGGGTGTGACTTTTG	
Alb-S516-FL-F-BC4	aacaccgattagCGTCATGGGTGTGACTTTTG	
Alb-S516-FL-F-BC5	aaccactctaagCGTCATGGGTGTGACTTTTG	
Alb-S516-FL-F-BC6	aaccgtatccgtCGTCATGGGTGTGACTTTTG	
Alb-S516-FL-F-BC7	aaccttggatggCGTCATGGGTGTGACTTTTG	
Alb-S516-FL-F-BC8	aacttctcttccCGTCATGGGTGTGACTTTTG	
Alb-S516-FL-F-BC9	aagaaggttacgCGTCATGGGTGTGACTTTTG	
Alb-S516-FL-F-BC10	aagagtggaagtCGTCATGGGTGTGACTTTTG	
Alb-S516-FL-F-BC11	aaggatactctcCGTCATGGGTGTGACTTTTG	
Alb-S516-FL-F-BC12	aagtcacacgccCGTCATGGGTGTGACTTTTG	
Alb-S516-FL-HA85-190-R	AAACTCATCAATGTATCTTATCATGTCTG	5.3kb
Alb-S516-FL-HA600-R	CCCAGGCTTTGCAGTCAAAC	4.9kb
**Forward right junction**		
Alb-S516-FR-F-BC1	acgtaggataaCCTTGGAAATCTCGCCAATA	
Alb-S516-FR-F-BC2	attaacatgccCCTTGGAAATCTCGCCAATA	
Alb-S516-FR-F-BC3	catacgaccgcCCTTGGAAATCTCGCCAATA	
Alb-S516-FR-F-BC4	aatagcaatcggCCTTGGAAATCTCGCCAATA	
Alb-S516-FR-F-BC5	aattacgagaggCCTTGGAAATCTCGCCAATA	
Alb-S516-FR-F-BC6	acaaccttcagcCCTTGGAAATCTCGCCAATA	
Alb-S516-FR-F-BC7	acaatgacaaggCCTTGGAAATCTCGCCAATA	
Alb-S516-FR-F-BC8	acaggtaataggCCTTGGAAATCTCGCCAATA	
Alb-S516-FR-F-BC9	accacaatgatgCCTTGGAAATCTCGCCAATA	
Alb-S516-FR-F-BC10	accgaacgccatCCTTGGAAATCTCGCCAATA	
Alb-S516-FR-F-BC11	acctcctgcgaaCCTTGGAAATCTCGCCAATA	
Alb-S516-FR-F-BC12	acgccatacataCCTTGGAAATCTCGCCAATA	
Alb-S516-FR-HA85-190-R	actcgtgcatgtgtgatataagttgtg	
Alb-S516-FR-HA600-F-BC1	aagaggaggtttgactgcaaagcctggg	
Alb-S516-FR-HA600-F-BC2	ctggaaatgtttgactgcaaagcctggg	
Alb-S516-FR-HA600-F-BC3	cttgttgggtttgactgcaaagcctggg	
Alb-S516-FR-HA600-F-BC4	tatgcgttgtttgactgcaaagcctggg	
Alb-S516-FR-HA600-R	GTGGGCAGATGACACACTGA	4.1kb
**Reverse left junction**		
Alb-S516-RL-F-BC1	ccagagcacacCGTCATGGGTGTGACTTTTG	
Alb-S516-RL-F-BC2	cgagactattcCGTCATGGGTGTGACTTTTG	
Alb-S516-RL-F-BC3	cttgacgttaaCGTCATGGGTGTGACTTTTG	
Alb-S516-RL-F-BC4	acggttctaatcCGTCATGGGTGTGACTTTTG	
Alb-S516-RL-F-BC5	actcagccggtaCGTCATGGGTGTGACTTTTG	
Alb-S516-RL-F-BC6	actgaggtgagcCGTCATGGGTGTGACTTTTG	
Alb-S516-RL-F-BC7	agagcataaggaCGTCATGGGTGTGACTTTTG	
Alb-S516-RL-F-BC8	aggaagagagagCGTCATGGGTGTGACTTTTG	
Alb-S516-RL-F-BC9	aggtaagcgtccCGTCATGGGTGTGACTTTTG	
Alb-S516-RL-F-BC10	agtatctggtgaCGTCATGGGTGTGACTTTTG	
Alb-S516-RL-F-BC11	agtccttctatcCGTCATGGGTGTGACTTTTG	
Alb-S516-RL-F-BC12	ataagactcaccCGTCATGGGTGTGACTTTTG	
Alb-S516-RL-R	ACACAGACCTTGCACAAATTTATACTACT	6.1kb
**Reverse right junction**		
Alb-S516-RR-F-BC1	gagtgcgtggtAATCACAGCCCATCAACTCC	
Alb-S516-RR-F-BC2	ggacaccgtaaAATCACAGCCCATCAACTCC	
Alb-S516-RR-F-BC3	gtcgtcatgtcAATCACAGCCCATCAACTCC	
Alb-S516-RR-F-BC4	atctccaaggtaAATCACAGCCCATCAACTCC	
Alb-S516-RR-F-BC5	atggagattggtAATCACAGCCCATCAACTCC	
Alb-S516-RR-F-BC6	attgagtcttccAATCACAGCCCATCAACTCC	
Alb-S516-RR-F-BC7	caacgtagtaagAATCACAGCCCATCAACTCC	
Alb-S516-RR-F-BC8	cacattcattggAATCACAGCCCATCAACTCC	
Alb-S516-RR-F-BC9	cacttccgagctAATCACAGCCCATCAACTCC	
Alb-S516-RR-F-BC10	cagagcaaccatAATCACAGCCCATCAACTCC	
Alb-S516-RR-F-BC11	caggttgatccgAATCACAGCCCATCAACTCC	
Alb-S516-RR-F-BC12	catcaagaacggAATCACAGCCCATCAACTCC	
Alb-S516-RR-R	ATTCAGAATTCTCGTAATGTTGAAGTATT	

Table S8. The primers utilized to distinguish NHEJ and HDR in Figure 6.

Primer	Sequence	Length
Alb-HA600-1.8kb-F1	CCTTAAACTGTGGCCTCACATTCC	1832 bp
Alb-HA600-1.8kb-R1	TACTTGGTGGGCAGATGACACACT	
Alb-HA600-1.4kb-F2	GGAGCAACTGAAGACTGTCATGG	1384bp
Alb-HA600-1.4kb-R2	AAGCCTTCCATTATTGCCCTATTTT	

Table S9. The primers utilized to detect the insertion of donor plasmid at a circular stage (with no sgDocut) .
