# Supplementary material for: Decoding the complexity of on-target integration: characterizing DNA insertions at the CRISPR-Cas9 targeted locus using nanopore sequencing
Source: BMC Genomics. 2024 Feb 17;25:189. doi: 10.1186/s12864-024-10050-6 (PMC10874558; doi:10.1186/s12864-024-10050-6)

# The Original Images of Electrophoretic Gels Showing Full-Length Membranes

The original image of Figure 2B

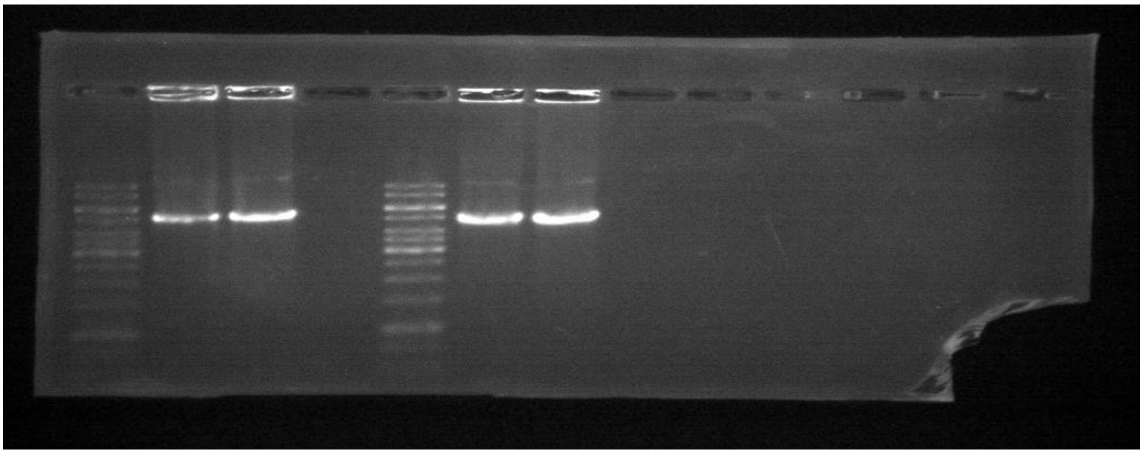

The following red-framed image is shown in Figure2B.

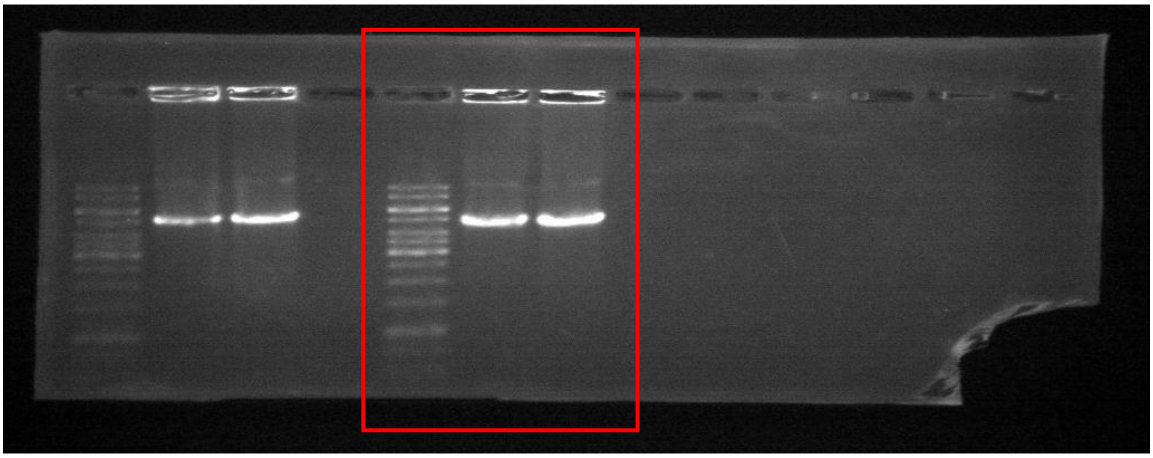

The original image of Figure 3E

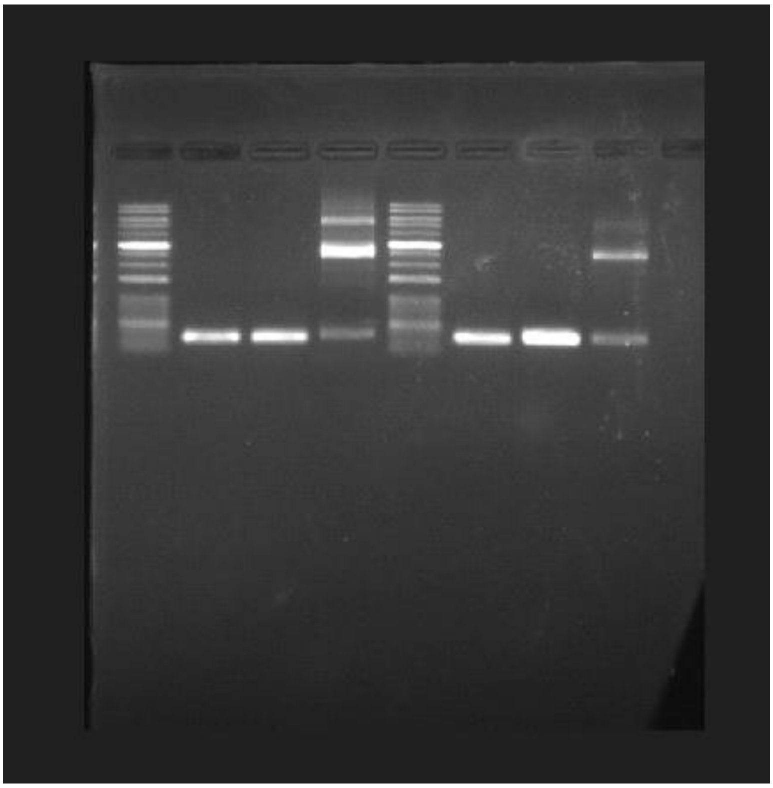

The following red-framed image is shown in Figure3E.

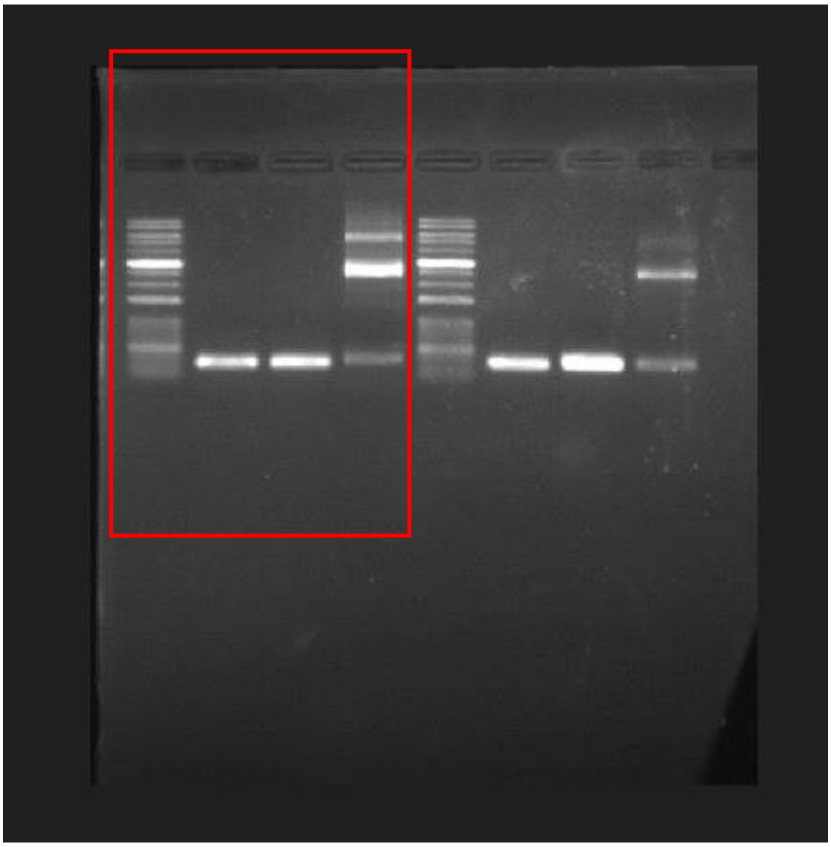

The original image of Figure 6B

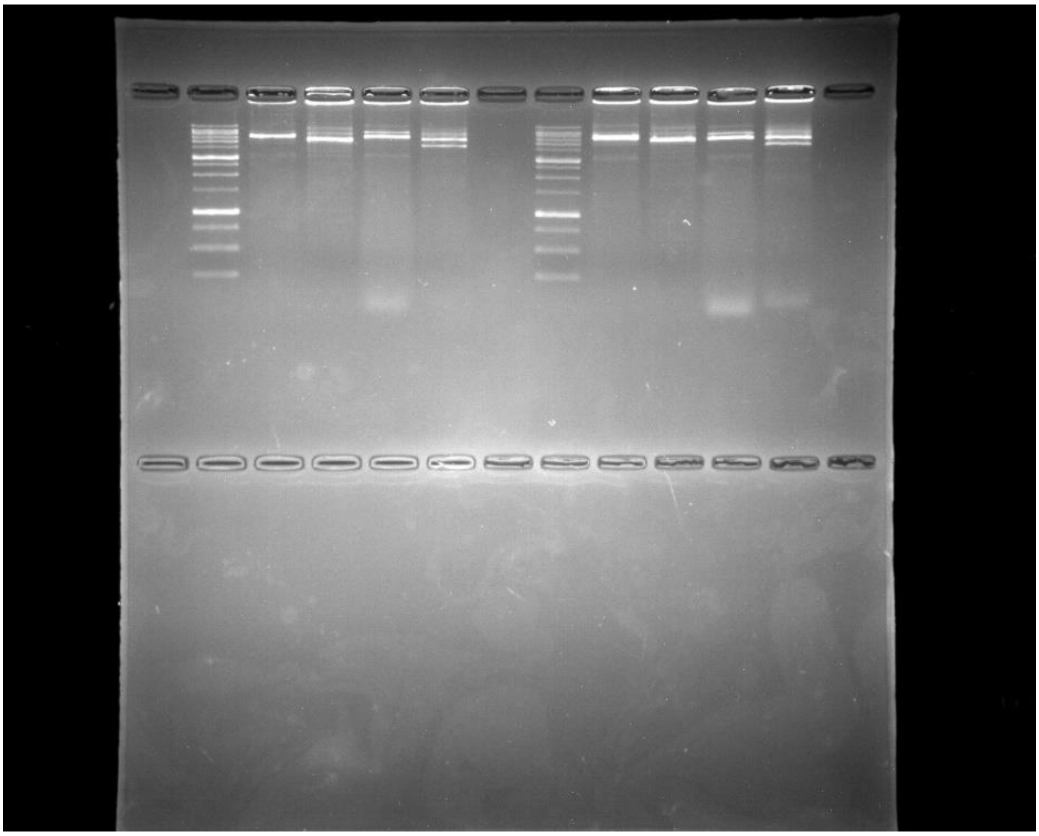

The following red-framed image is shown in Figure6B

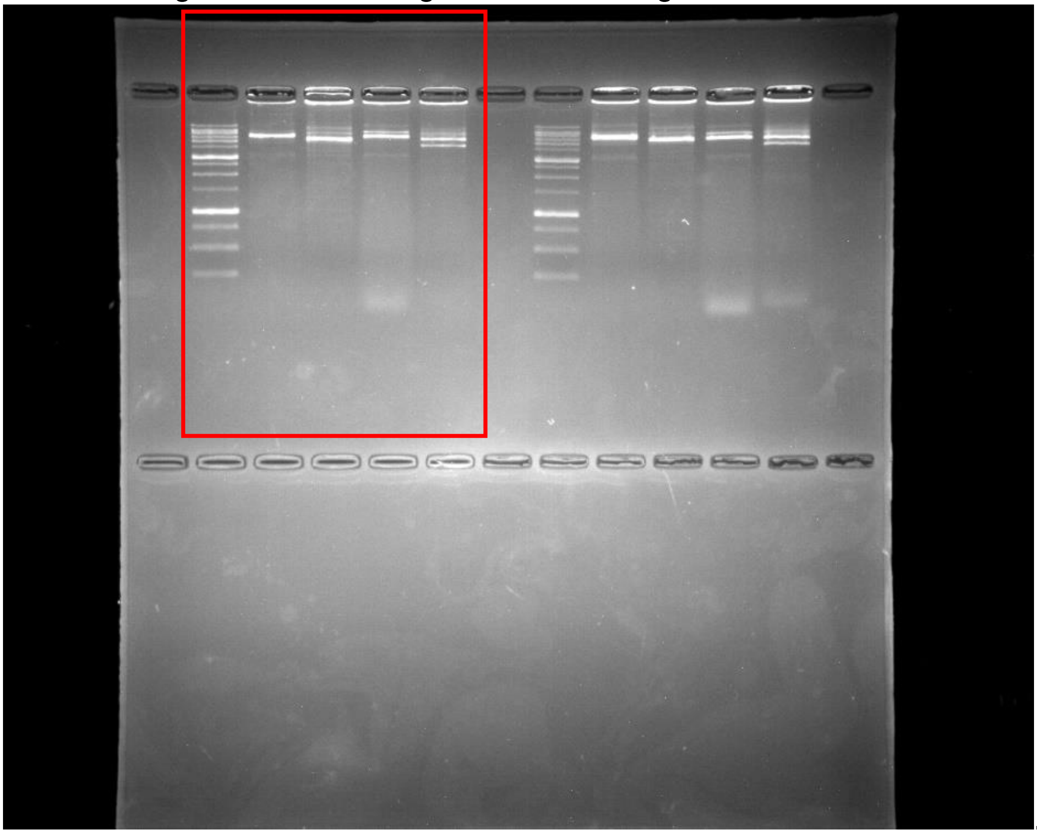

.

The original image of Supplementary Figure S2A

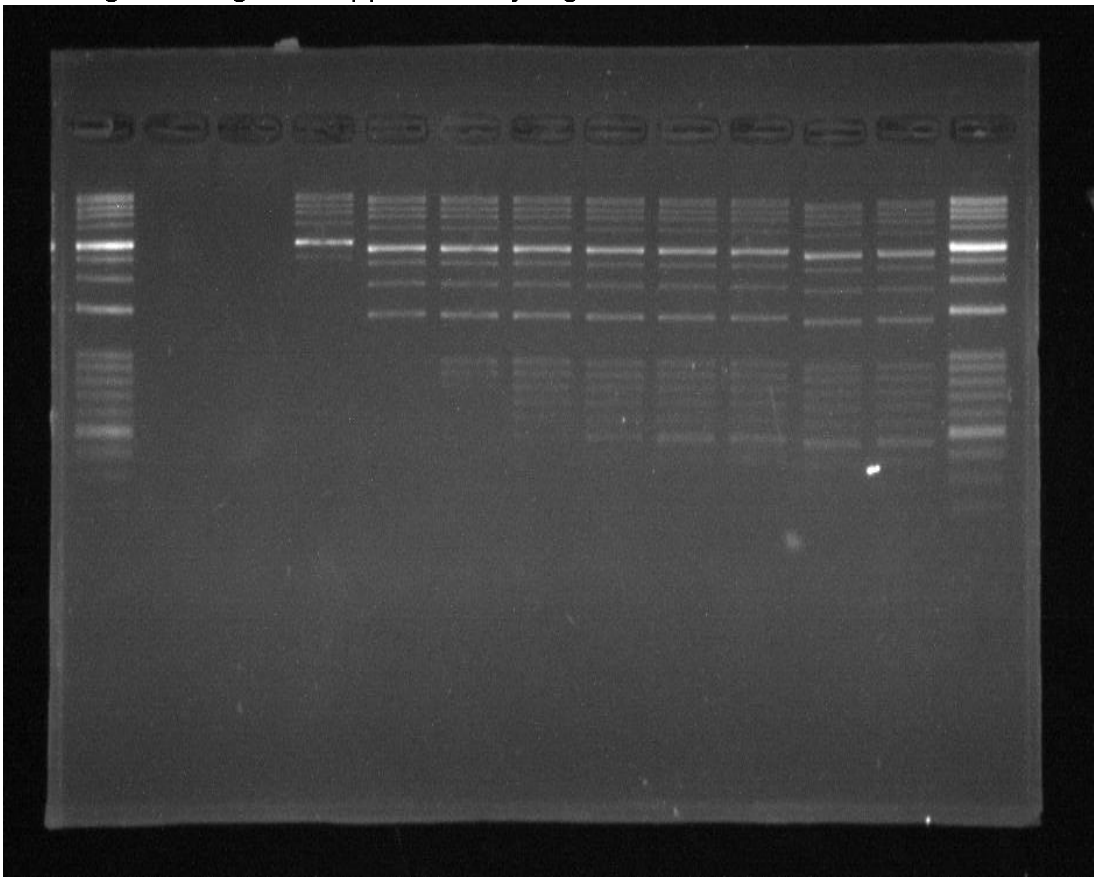

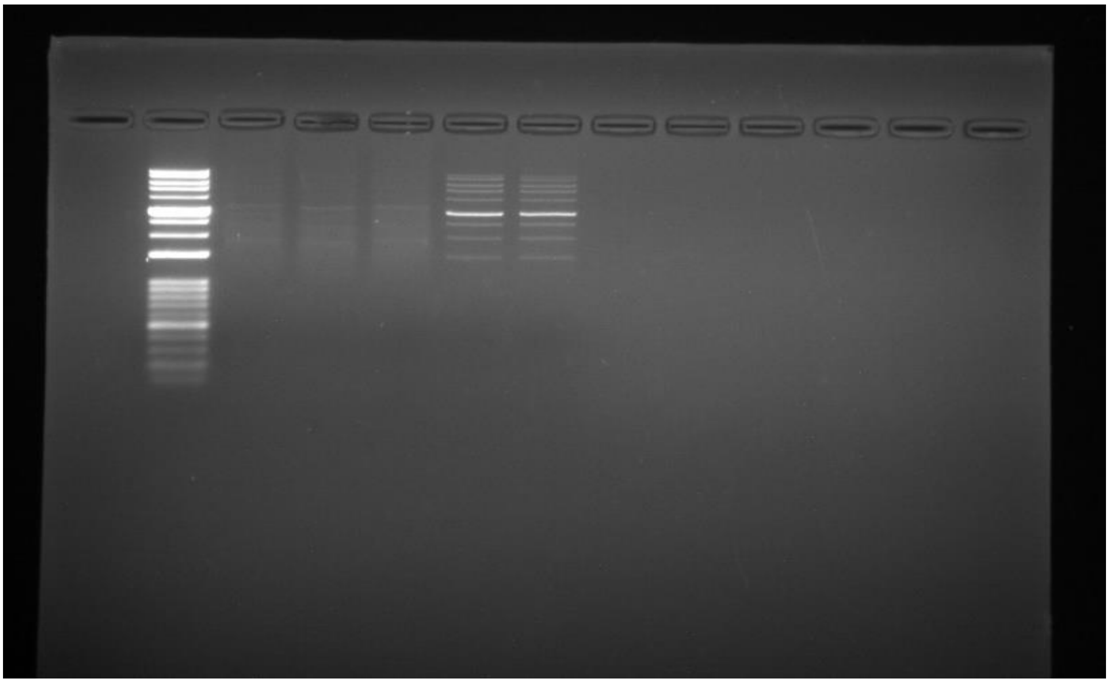

The following red-framed image is shown in Supplementary Figure S2A.

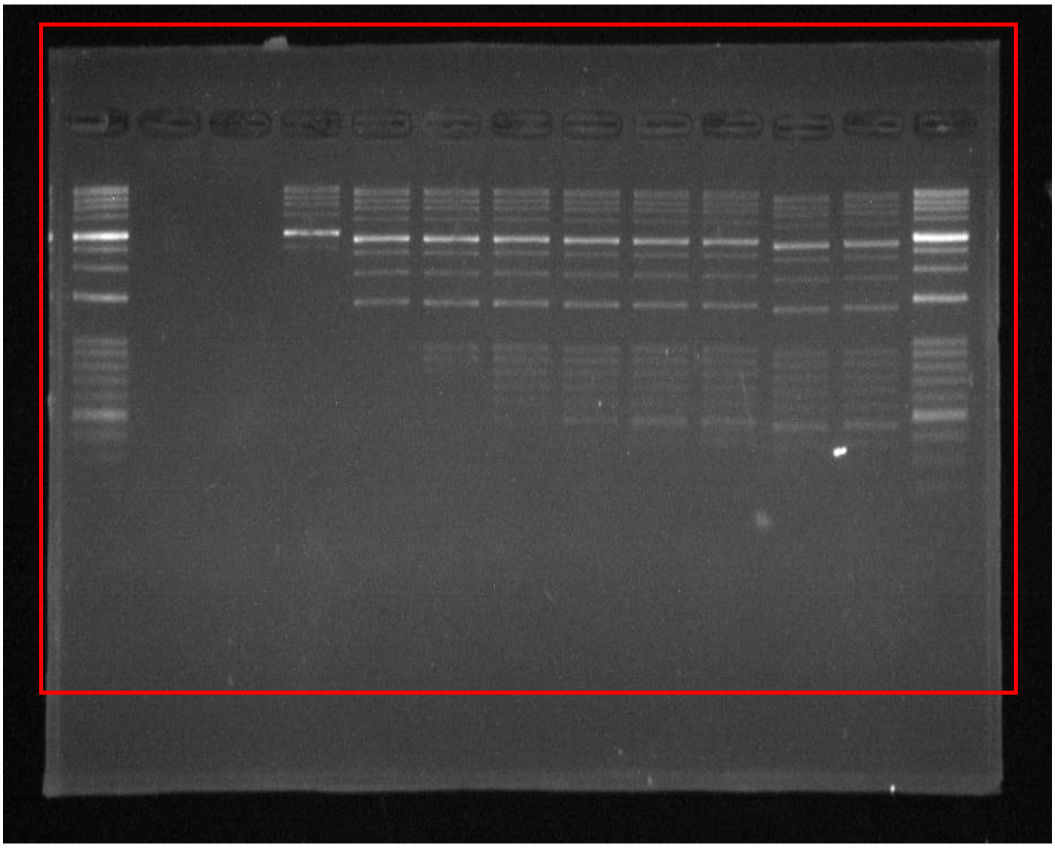

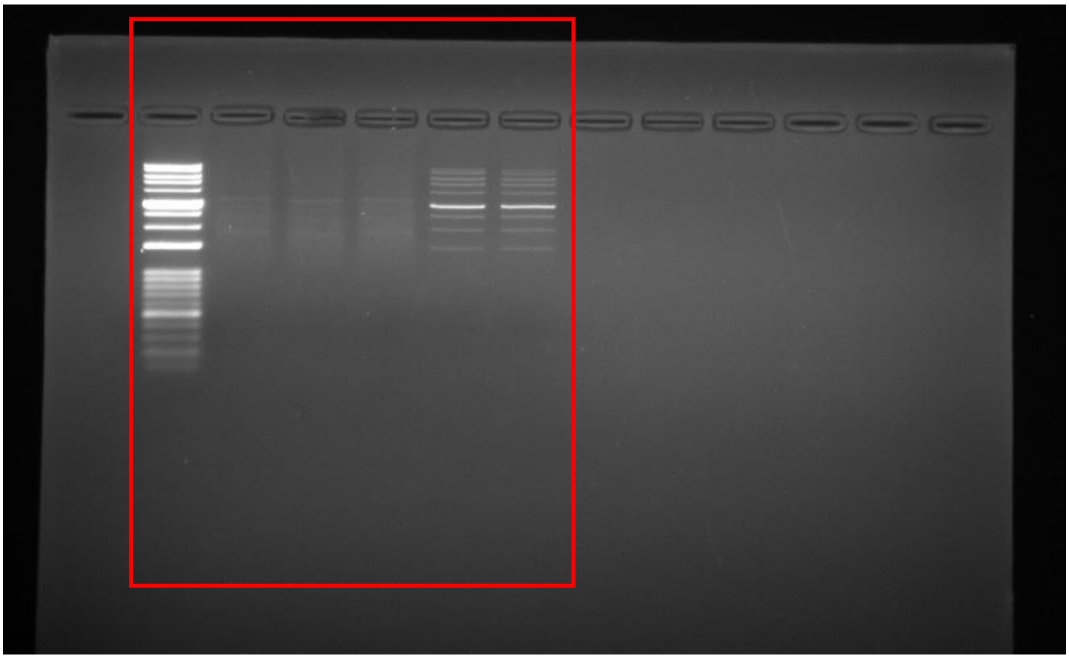

The original image of Supplementary Figure S2C

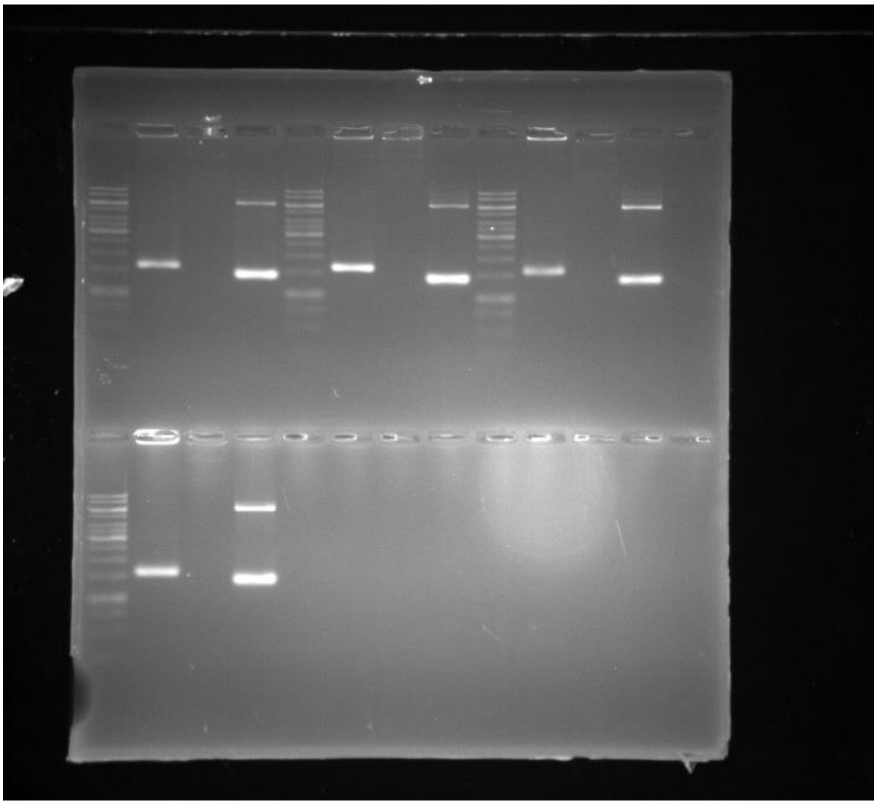

The following red-framed image is shown in Supplementary Figure S2C.

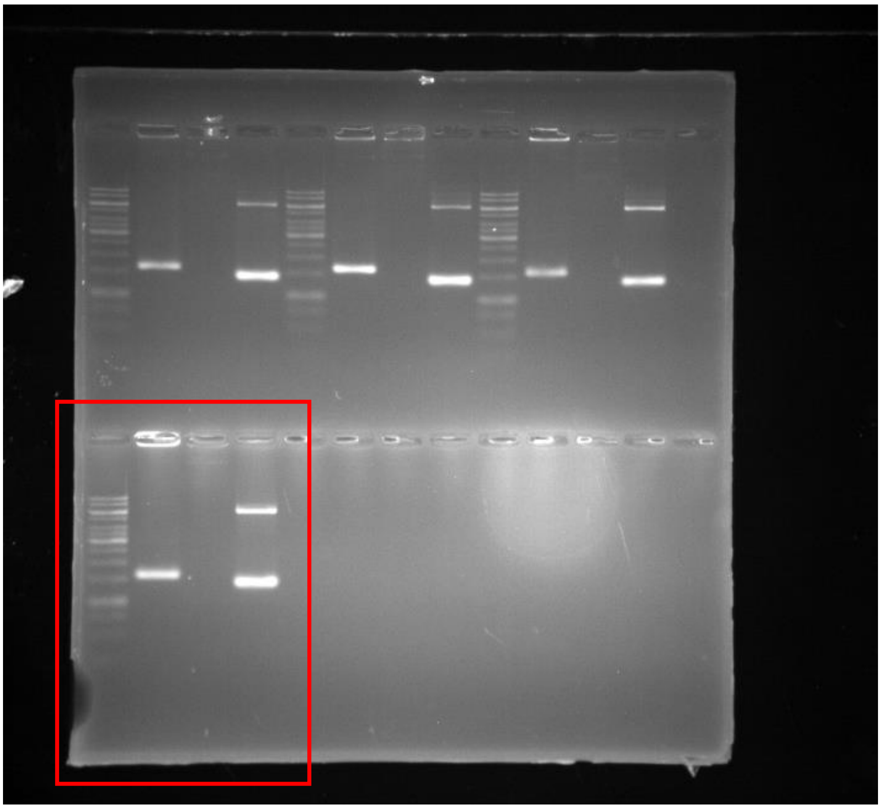

Supplement: Supplementary file 2 — Supplementary Material 2 [file 12864_2024_10050_MOESM2_ESM.pdf]
